# Baseline Characteristics of Mitochondrial DNA and Mutations Associated With Short-Term Posttreatment CD4+T-Cell Recovery in Chinese People With HIV

**DOI:** 10.3389/fimmu.2021.793375

**Published:** 2021-12-14

**Authors:** Anni Liu, Qian Wei, Haijiang Lin, Yingying Ding, Yan V. Sun, Dan Zhao, Jiayu He, Zhonghui Ma, Feihu Li, Sujuan Zhou, Xiaoxiao Chen, Weiwei Shen, Meiyang Gao, Na He

**Affiliations:** ^1^ Department of Epidemiology, School of Public Health, Fudan University, Shanghai, China; ^2^ Key Laboratory of Public Health Safety of Ministry of Education, Fudan University, Shanghai, China; ^3^ Weill Cornell Graduate School of Medical Sciences, Cornell University, New York, NY, United States; ^4^ Department of AIDS/STD Control and Prevention, Taizhou City Center for Disease Control and Prevention, Taizhou, China; ^5^ Department of Epidemiology, Rollins School of Public Health, Emory University, Atlanta, GA, United States; ^6^ Department of Biomedical Informatics, School of Medicine, Emory University, Atlanta, GA, United States; ^7^ School of Mathematical Sciences, Fudan University, Shanghai, China; ^8^ Key Laboratory of Health Technology Assessment, National Commission of Health, Fudan University, Shanghai, China

**Keywords:** mitochondrial DNA, HIV, PLWH, CD4+T cell, ART

## Abstract

**Background:**

Mitochondrial DNA (mtDNA) profiles and contributions of mtDNA variants to CD4+T-cell recovery in Euramerican people living with HIV (PLWH) may not be transferred to East-Asian PLWH, highlighting the need to consider more regional studies. We aimed to identify mtDNA characteristics and mutations that explain the variability of short-term CD4+T-cell recovery in East-Asian PLWH.

**Method:**

Eight hundred fifty-six newly reported antiretroviral therapy (ART)-naïve Chinese PLWH from the Comparative HIV and Aging Research in Taizhou (CHART) cohort (Zhejiang Province, Eastern China) were enrolled. MtDNA was extracted from peripheral whole blood of those PLWH at HIV diagnosis, amplified, and sequenced using polymerase chain reaction and gene array. Characterization metrics such as mutational diversity and momentum were developed to delineate baseline mtDNA mutational patterns in ART-naïve PLWH. The associations between mtDNA genome-wide single nucleotide variants and CD4+T-cell recovery after short-term (within ~48 weeks) ART in 724 PLWH were examined using bootstrapping median regressions.

**Results:**

Of 856 participants, 74.18% and 25.82% were male and female, respectively. The median age was 37 years; 94.51% were of the major Han ethnicity, and 69.04% and 28.62% were of the heterosexual and homosexual transmission, respectively. We identified 2,352 types of mtDNA mutations and mtDNA regions *D-loop*, *ND5*, *CYB*, or *RNR1* with highest mutational diversity or volume. Female PLWH rather than male PLWH at the baseline showed remarkable age-related uptrends of momentum and mutational diversity as well as correlations between CD4+T <200 (cells/μl) and age-related uptrends of mutational diversity in many mtDNA regions. After adjustments of important sociodemographic and clinical variables, m.1005T>C, m.1824T>C, m.3394T>C, m.4491G>A, m.7828A>G, m.9814T>C, m.10586G>A, m.12338T>C, m.13708G>A, and m.14308T>C (at the Bonferroni-corrected significance) were negatively associated with short-term CD4+T-cell recovery whereas m.93A>G, m.15218A>G, and m.16399A>G were positively associated with short-term CD4+T-cell recovery.

**Conclusion:**

Our baseline mtDNA characterization stresses the attention to East-Asian female PLWH at risk of CD4+T-cell loss-related aging and noncommunicable chronic diseases. Furthermore, mtDNA variants identified in regression analyses account for heterogeneity in short-term CD4+T-cell recovery of East-Asian PLWH. These results may help individualize the East-Asian immune recovery strategies under complicated HIV management caused by CD4+T-cell loss.

## Introduction

The central issue for people living with HIV (PLWH) is the CD4+T-lymphocyte loss, and a progressive depletion of CD4+T cells presages the acquired immunodeficiency syndrome (AIDS) (1) accompanied with opportunistic infection, cancer, cardiovascular and bone diseases, renal and hepatic disruption and other complications (2). As AIDS and non-AIDS morbidity and mortality are determined by the state of CD4+T-cell loss (3, 4), a fast restoration on CD4+T-cell levels during antiretroviral therapy (ART) is especially important (5, 6). Although combination ART enhances the CD4+T cell levels of PLWH by HIV replication suppression, 15%–50% of PLWH do not achieve satisfied CD4+T-cell levels within 48-week treatment (7–10). Those PLWH with inadequate CD4+T-cell gains are at risk of continued immunity disruption and complications. Host genetic architecture that helps individualize the short-term CD4+T-cell recovery is an unmet clinical need in PLWH.

Mitochondria, as powerhouses of immunity (11), have their own genomes named mitochondrial DNA (mtDNA), which provides the molecular basis of a plethora of pathological conditions from aging, diabetes, cancer, myopathy, deafness, Alzheimer’s disease, Parkinson’s disease, to immunodeficiency (12–14). MtDNA aberrations regulate apoptosis signaling (15–17), one of the critical mechanisms that are intrinsically linked to the pool size, development, and function of T lymphocytes (18–23).

Over the past two decades, characterization of mtDNA mutations in PLWH has been constructed in participants of European and American ancestries, with foci on the antiretroviral toxicities on mtDNA maintenance in PLWH. These earlier studies usually delineated one specific dimension of the mtDNA genome based on commonly used metrics, such as the frequencies of specific nucleotide variants, within a relatively small sample size (ranging from 14 to 87 HIV-infected participants) (24–27). Nevertheless, partially due to absence of more diverse characterization metrics and larger samples, it has been difficult to elucidate gender-, age-, and immune level-specific mtDNA patterns that can point out risky PLWH susceptible to aberrant mtDNA mutagenesis. Growing evidence shows that CD4+T-cell loss-related noncommunicable chronic diseases (NCDs) emerge as pervasive health concerns in HIV-infected populations (28–30) and expansion of mitochondrial DNA mutations linked to increased apoptosis drives aging and age-related diseases (31, 32). Design and applications of mtDNA screening and intervention to HIV-infected patients who are burdened with mtDNA mutations may personalize the complicated management of CD4+T-cell loss and relevant chronic diseases in HIV patient care. Identification of risky PLWH through a more comprehensive molecular portrait of host mtDNA in PLWH is an important part of these design and applications.

Two larger Euromerican cohort studies (633 and 423 participants, respectively) studied the contributions of mtDNA mutations at a resolution of the composite haplogroup and/or individual variant to AIDS progression/CD4+T-cell recovery, both using CD4+T-cell counts as outcomes (14, 33). Similarly, two smaller European studies (469 participants with cross-sectional design and 275 participants with cohort design, respectively) studied the associations between mtDNA haplogroups and AIDS progression/CD4+T cell recovery (34, 35). However, as mtDNA sequences are remarkably diverse in different regions (36, 37), it is difficult to transfer previous results directly to East-Asian PLWH. A mtDNA genome-wide study targeting East-Asian regions is necessary. Furthermore, most of the previous studies did not study how mtDNA correlates with CD4+T cell recovery within ~48-week antiretroviral treatment, and they usually selected mtDNA variants composing specific mtDNA haplogroups. It remains unclear as to whether mtDNA variants within a specific haplogroup and other mtDNA variants not attached to specific haplogroups could inform personalized diagnosis and treatment for short-term CD4+T-cell recovery.

In this paper, we first delineated the baseline mutational profiles of mtDNA including mutational diversity, volume, and prevalence, bias and physicochemical properties of amino acid changes in 856 ART-naïve Chinese PLWH, then investigated gender-, age-, and immune level-related mutational patterns at the subpopulation level, and finally systematically assessed the contributions of single nucleotide variants across the complete mtDNA genome to short-term CD4+T-cell recovery.

## Materials and Methods

### Study Participants

We profiled the complete mtDNA in ART-naïve PLWH registered with the Comparative HIV and Aging Research in Taizhou (CHART) cohort (38) and China National HIV/AIDS Comprehensive Response Information Management System (CRIMS) (39). Eight hundred fifty-six newly diagnosed HIV-infected individuals in Taizhou prefecture of Zhejiang province in east China from 2003 to 2017 were inform consented to participate in this study. The baseline and follow-up demographic, clinical, and laboratory information of participants at the pre-ART visits (refer to the visits at HIV diagnosis) and at the post-ART visits was retrieved from CRIMS (39).

To investigate patterns of mtDNA mutations under different gender, age, and pre-ART immune states, we performed ethnicity-stratified (33, 40) analyses in 806 ART-naïve Chinese PLWH with the dominant Han ethnic background (occupying 94% of all participants), and ensured at least five participants with the identical ethnicity in each of 16 subpopulations classified by gender (male; female), age [17–29; 30–44; 45–59; ≥60 (in years)], and immune levels at the pre-ART visit [severe immunodeficiency: CD4 <200; mild immunodeficiency: CD4 ≥200 (in cells/μl) (41)] (**Supplementary Table S1**). The studies involving human participants were reviewed and approved by the Institutional Review Board of Fudan University. The participants provided their written informed consent to participate in this study.

### Mitochondrial DNA Sequencing, Nucleotide Variant Calling, and Haplogroup Classification

MtDNA was extracted from whole blood for each individual at the pre-ART visit using QIAamp DNA blood mini Kit (Qiagen, Hilden, Germany). For 679 untreated PLWH, four overlapping segments of mtDNA were amplified by polymerase chain reaction (PCR) using four standard pairs of primers (**Supplementary Table S2A**). Each segment was ~4,800 bp in length and overlapped with the neighboring fragments by over 500 bp, to prevent the amplification of nuclear mitochondrial pseudogenes (nuMTs) (42, 43). LA Taq Version 2.0 plus dye Kits (Sangon Biotechnologies, Inc., Shanghai, China) were used for PCR assay with the following procedures: an initial 94°C for 1 min, followed by 30 cycles of 94°C for 30 s, 66.5°C for 5 min, and 72°C extending for 10 min. The PCR products were directly sequenced by 47 internal primers (**Supplementary Table S2B**) in Sangon Biotechnologies, Inc (42, 44, 45). and were assembled using the Sequencher5.4 software (Gene Codes Corporation, Ann Arbor, MI, USA) relative to the Revised Cambridge Reference Sequence (rCRS) (46, 47). Sequence variants were determined by the pairwise sequence alignment relative to the rCRS on the EMBOSS Needle Platform (48). For the other 177 untreated PLWH, the standard procedure of GeneChip Human Mitochondrial Resequencing Array v2.0 (Affymetrix, Santa Clara, CA, USA) was employed to sequence the entire mtDNA. Array intensity data and base variant calling were processed through the standard MitoChip Filtering Protocol which outputted the average call rate of 99.75% (49). Welch *t*-test was performed to compare the distributions of mtDNA substitutions in participants using two different sequencing platforms, and no statistically significant differences were found (*p* = 0.875). As Affymetrix GeneChip was unable to identify insertions and deletions (indels) accurately, we only described the distributions of indels in 679 participants, without any downstream analyses on indels across 16 subpopulations and mtDNA genome-wide association study that needed pooled mtDNA information from two sequencing platforms. For all 856 participants, haplogroups were assigned using HaploGrep 2 and Phylotree Build 17 (50, 51).

### Metrics to Characterize Mitochondrial DNA

Mutational diversity was defined as the number of distinct types of nucleotide variants (**Supplementary Table S1**) across the mtDNA, and mutational volume was defined as the number of counts of nucleotide variants across the mtDNA. At the same population scale, mutational diversity is lower bound on mutational volume, as duplicate types of nucleotide variants occur at the same nucleotide site. However, at the individual scale, mutational diversity equals mutational volume. These two metrics have several variations, as nucleotide variants can be further subdivided into synonymous and nonsynonymous substitutions, and indels. For instance, diversity of nonsynonymous substitutions was defined as the number of distinct types of nonsynonymous substitutions. Diversity density was defined as the diversity of substitutions divided by the sample size per subpopulation and kilobase pair size per macrodivision across the mtDNA. Relative diversity was defined as the observed number of distinct types of definite substitutions relative to all possible substitutions deduced from the human mitochondrial genetic code and rCRS, across the mitochondrial coding regions. Both two metrics also have several variations, as substitutions can be further subdivided into synonymous substitutions, nonsynonymous substitutions, transitions, and transversions. A definite substitution refers to a base mutation into a specified purine or pyrimidine (such as A>T), whereas an ambiguous substitution refers to a base mutation into a mixture of bases [such as A>R (A or G)] (52). 96% (2,137) of the total of 2,221 distinct types of substitutions were definite in this study. Relative diversity density was defined as the observed number of distinct types of definite substitutions divided by the sample size of each subpopulation relative to all possible substitutions, across the mitochondrial coding regions. This metric has several variations, as substitutions can be further subdivided into synonymous substitutions, nonsynonymous substitutions, transitions, and transversions. Momentum was defined as the average increase in the observed number of distinct types of definite substitutions divided by the sample size of each subpopulation per unit increase in the maximum number of synonymous/nonsynonymous substitutions, across the mitochondrial coding regions. This index is measured by the linear slope (β) of relative diversity density of synonymous/nonsynonymous substitutions. The lower the β is, the higher the momentum is, or vice versa. Bias of amino acid changes was defined as the proportion of the observed number of changes to a different amino acid which are deduced from distinct types of definitive nonsynonymous substitutions, relative to the maximum number of changes to this amino acid. Bias density of amino acid changes was defined as the proportion of the observed number of changes to a different amino acid divided by the sample size of each subpopulation relative to the maximum number of changes to this amino acid. Physicochemical property change of amino acid changes was defined as the prevalence of each type of physicochemical alterations brought by amino acid changes which are deduced from distinct types of definitive non-synonymous substitutions. Physicochemical property change density of amino acid changes was defined as the prevalence of each type of physicochemical alterations brought by amino acid changes which are deduced from distinct types of definite nonsynonymous substitutions, divided by the sample size of each subpopulation. When assessing the mutational patterns related to different levels of gender, age, and immunity across suppopulations, we combined the control region (D-loop), 24 RNAs and 13 protein-coding genes into seven macrodivisions (D-loop; RNRs1-2(RNRs); 22tRNAs(tRNAs); NDs1-6, 4L(NDs); COs1-3(COs); ATPs6,8(ATPs); CYB), given biological significance (53), and zero or too low-level mutation in some mtDNA genes.

### Mitochondrial DNA Genome-Wide Association Study

As indel information of 177 (21%) participants taking the Affymetrix gene chip sequencing was lost, only substitutions were studied in association analyses. To investigate the robust and independent associations between mtDNA substitutions and CD4+T-cell recovery, the least absolute deviation-based bootstrapping median regression with 1,000 replications (54) was performed when residuals’ homoscedasticity and normality required by the ordinary least squares regression was violated (55). Two hundred eighty-seven substitutions with prevalence between 1% (8 counts) and 99% (716 counts) in 724 participants were used. In the regression models using the pre-ART dataset, CD4+T-cell count at the pre-ART visit (continuous) was the dependent variable, 287 substitutions were the independent variables, and gender (male, female), age at the pre-ART visit (continuous), ethnicity (Han, other minorities), as well as HIV transmission mode [heterosexual, homosexual (14, 56), others] were the covariates. In the regression models using the post-ART dataset, CD4+T-cell count at the post-ART visit (continuous) was the dependent variable, 287 substitutions were the independent variables, and aforementioned covariates as well as CD4+T-cell count at the pre-ART visit (continuous), ART regimens (efavirenz/nevirapine+lamivudine+zidovudine (57, 58), efavirenz/nevirapine+lamivudine+stavudine (59), others), and ART treatment duration [<3, ≥3 (month)] were the covariates. As 574 multivariate regressions were performed using the same dataset, the Bonferroni-corrected threshold for *p*-value was set as 8.71 × 10^−5^ (0.05/574 tests).

### Other Statistical Analyses

Linear regressions were performed to test the relationships between the observed number of definite synonymous/nonsynonymous/transitional/transversional substitutions and the maximum number of synonymous/nonsynonymous/transitional/transversional substitutions as well as to test age-related trends of mutational diversity and volume when age was ordinally categorized into four groups (60). Wilcoxon rank sum test was used to compare the overall distributions of bias densities of amino acid changes between subpopulations of pre-ART CD4 <200 and gender- and age-matched counterparts of pre-ART CD4 ≥200.

All statistical tests were two-tailed. All statistical analyses were performed using the Excel 2016 (Microsoft, Redmond, WA, USA), R version 4.0.2 (R Foundation for Statistical Computing, Vienna, Austria), Stata 15 (StataCorp, College Station, TX, USA), or OriginPro 2020 (OriginLab, Northampton, MA, USA) whichever was appropriate.

## Results

### Participant Characteristics

**Figure 1:**
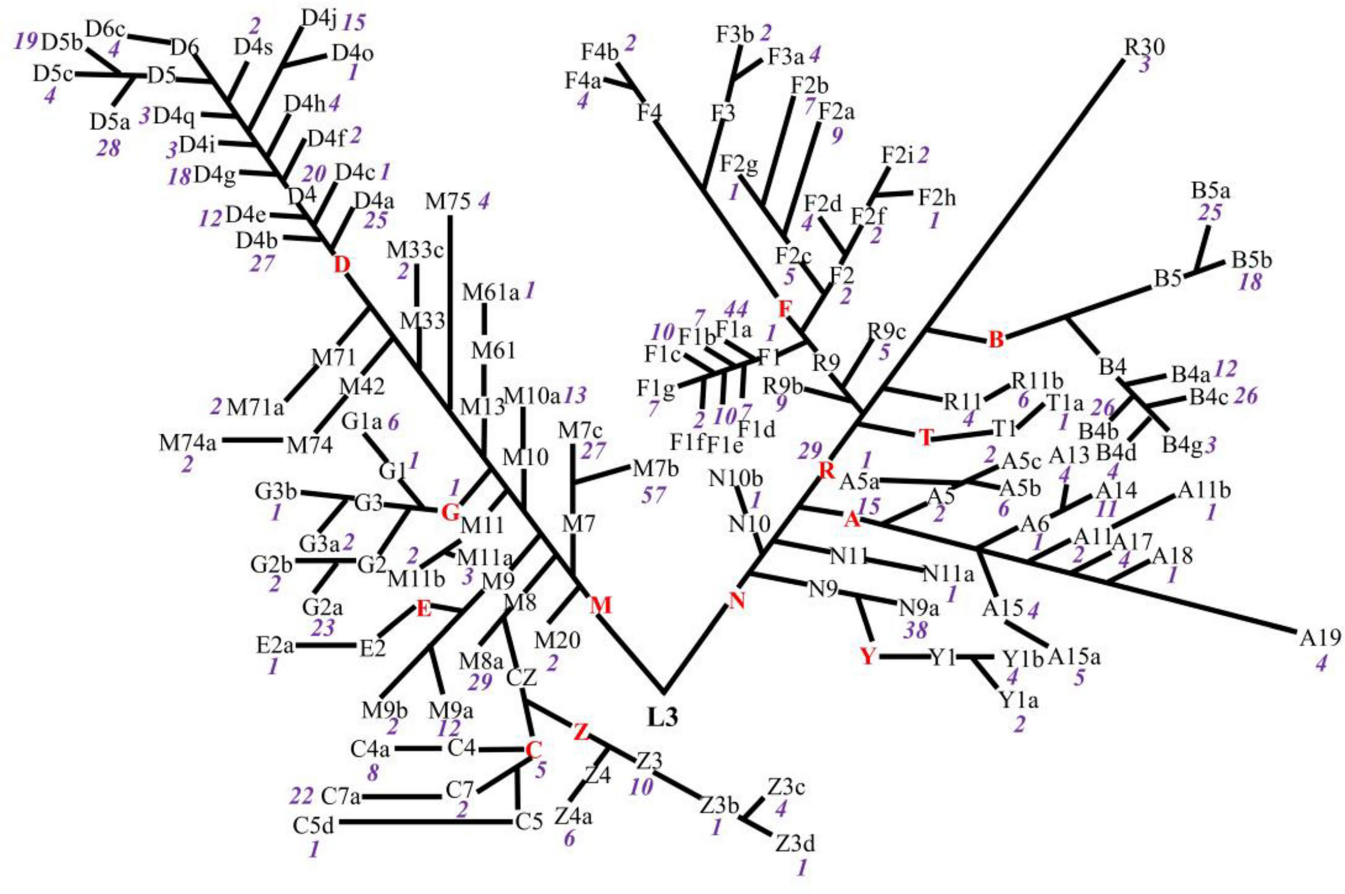
Classification tree of mtDNA haplotypes presenting the distributions of haplogroups in untreated PLWH. The red capital letters denote the major haplogroups, the black letters and/or numbers denote the subhaplogroups, and the purple numbers denote the frequency of each subhaplogroup.

Eight hundred fifty-six ART-naïve PLWH in Taizhou, a coastal prefecture of Zhejiang province in Eastern China, were enrolled; 74% were men. The median age was 37 years (interquartile range: 28–47 years); 95% were of the major Han ethnicity, and 69% and 29% were heterosexually and homosexually infected with HIV, respectively. The median pre-ART CD4+T-cell count at baseline enrollment was 219 cells/μl (interquartile range: 149–295 cells/μl), and 41% of participants had their CD4+T-cell counts lower than 200 cells/μl. Thirteen mtDNA major haplogroups (A, B, C, D, E, F, G, M, N, R, T, Y, and Z) were identified, of which D was the most prevalent, followed by M and F (**Table 1**). The distribution of subhaplogroups was shown in the classification tree (**Figure 1**). Seven hundred twenty-two (84.3%) participants had post-ART CD4+T-cell counts within the first 12 months after ART initiation, and two individuals had post-ART CD4+T-cell counts at the 14th- and 16th-month visits from the dates of their ART initiation, respectively. These 724 participants and overall 856 participants were similar in the distributions of gender, age, ethnicity, HIV transmission mode, CD4+T-cell count, and mtDNA haplogroup. The median change from pre- to post-ART in the CD4+T-cell count was 70 cells/μl (interquartile range: 12–142 cells/μl), and 77% of participants consistently took efavirenz/nevirapine + zidovudine + lamivudine (**Table 1**).

**Table 1:** Sociodemographic, clinical, and laboratory characteristics of participants.

Variable	Participants at the pre-ART visit (*N* = 856)	Participants at the post-ART visit (*N* = 724)
	Number (%)	Number (%)
Gender		
Male	635 (74.18)	540 (74.59)
Female	221 (25.82)	184 (25.41)
Age at the pre-ART visit [year, (median, (IQR)]	37 (28–47)	36 (28–46)
17–29	258 (30.14)	227 (31.35)
30–44	347 (40.54)	296 (40.88)
45–59	161 (18.81)	132 (18.23)
≥60	90 (10.51)	69 (9.53)
Ethnicity		
Han	809 (94.51)	685 (94.61)
Minorities	47 (5.49)	39 (5.39)
HIV transmission mode		
Heterosexual	591 (69.04)	506 (69.89)
Homosexual	245 (28.62)	208 (28.73)
Others	20 (2.34)	10 (1.38)
CD4+T cell count at the pre-ART visit [cells/μl, (median, (IQR)]	219 (149–295)	223 (155–292)
<200	353 (41.24)	292 (40.33)
≥200	500 (58.41)	430 (59.39)
Change from pre-ART to post-ART in the CD4+T cell count [cells/μl, (median, IQR)] ART regimen	–	70 (12–142)
EFV/NVP+3TC+AZT	–	558 (77.07)
EFV/NVP+ 3TC+d4T	–	118 (16.30)
Others^a^	–	48 (6.63)
ART treatment duration [months, (median, (IQR)]	–	3 (2–3)
<3	–	292 (40.33)
≥3	–	432 (59.67)
MtDNA haplogroup^b^		
A	63 (7.36)	55 (7.60)
B	114 (13.32)	97 (13.40)
C	38 (4.44)	30 (4.14)
D	188 (21.96)	158 (21.82)
E	1 (0.12)	–
F	133 (15.54)	110 (15.19)
G	36 (4.21)	31 (4.28)
M	158 (18.46)	136 (18.78)
N	40 (4.67)	35 (4.83)
R	56 (6.54)	48 (6.63)
T	1 (0.12)	1 (0.14)
Y	6 (0.70)	6 (0.83)
Z	22 (2.57)	17 (2.35)

IQR, interquartile range; EFV, efavirenz; 3TC, lamivudine; AZT, zidovudine; d4T, stavudine; TDF, tenofovir; NVP, nevirapine; LPV/RTV, lopinavir/ritonavir.

a Others included EFV+3TC+TDF, NVP+3TC+TDF, LPV/RTV+3TC+AZT, and LPV/RTV+3TC+d4T.

b Mitochondrial haplogroups were determined by mtDNA sequencing at the pre-ART visit.

### Profiles of Mitochondrial DNA in 856 ART-Naïve Chinese PLWH

**Figure 2:**
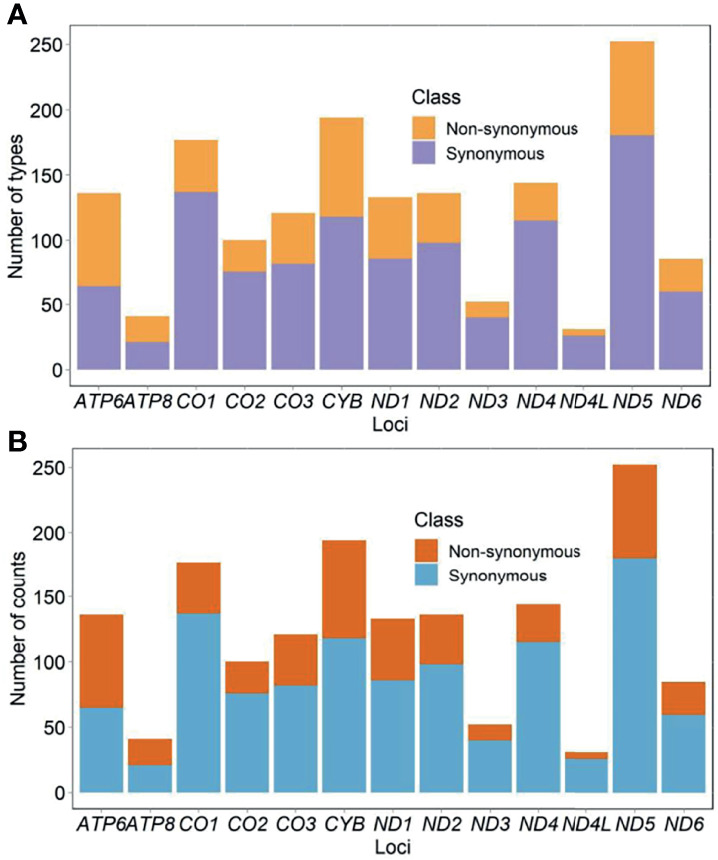
Diversity **(A)** and volume **(B)** of mtDNA substitutions in untreated PLWH.

**Figure 3:**
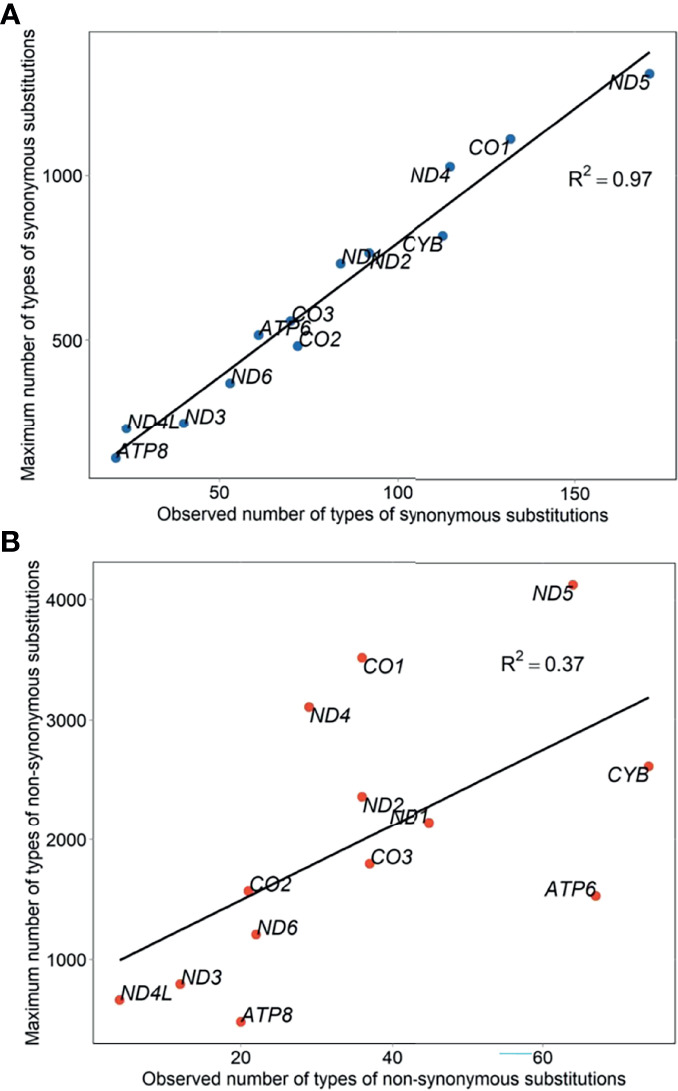
Relative diversity of mtDNA synonymous **(A)** and nonsynonymous **(B)** substitutions in untreated PLWH.

Eight hundred fifty-six participants showed 2,222 types of substitutions (30,578 counts) and 130 types of insertions and deletions (indels) (1,794 counts). Of those, 1,609 types (68% or 1,609/2,352) located in the protein-coding genes. The list of all identified mutations was included in **Supplementary Table S3**. Mutational diversity peaked in *D-loop* (18% or 430/2,352), *ND5* (11% or 250/2,352), and *CYB* (8% or 196/2,352), whereas mutational volume peaked in *D-loop* (30% or 9,785/32,372), *CYB* (12% or 3,808/32,372), and *RNR1* (7% or 2,330/32,372). Diversity of synonymous substitutions peaked in *ND5* (180), *CO1* (137), and *CYB* (118), whereas diversity of nonsynonymous substitutions peaked in *CYB* (76), *ND5* (72), and *ATP6* (71). Volume of synonymous substitutions peaked in *CYB* (1,807), *ND4* (1,697), and *CO1* (1,695), whereas volume of nonsynonymous substitutions peaked in *CYB* (1,999), *ATP6* (1,701), and *ND5* (656) (**Figures 2A, B** **)**. Relative diversity of synonymous and nonsynonymous substitutions was 13.0% (1,075 out of 8,291 possible) and 1.8% (469 out of 25,894 possible), respectively. We observed a strong consistency in relative diversity of synonymous substitutions across all protein-coding genes (*R* ^2^ = 0.97, **Figure 3A**). However, this consistency was less pronounced for nonsynonymous substitutions (*R* ^2^ = 0.37), where *ATP6* and *ATP8* had richer relative diversity but *CO1*, *ND4*, and *ND5* had poorer relative diversity (**Figure 3B**).

**Figure 4:**
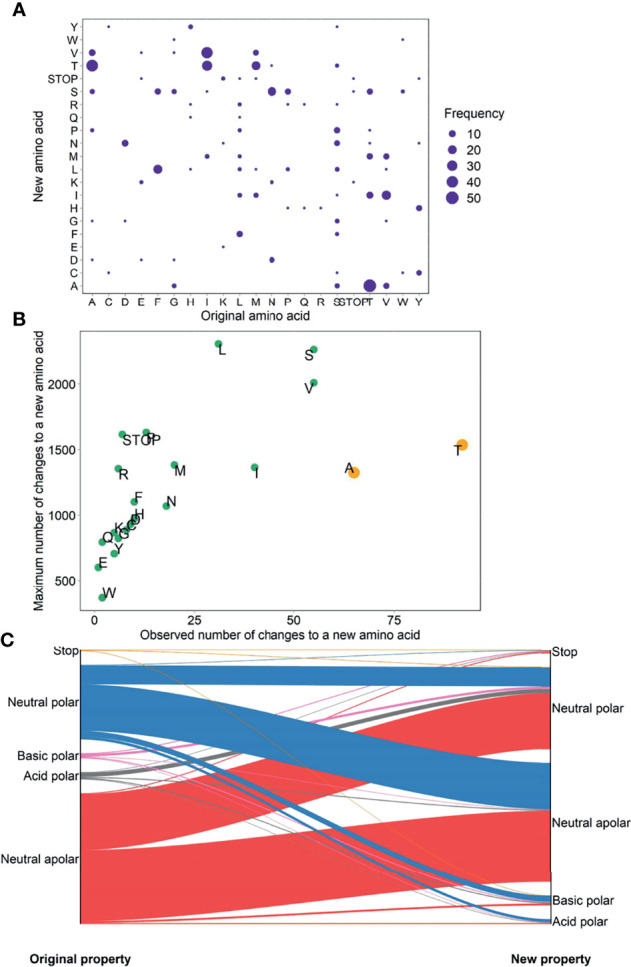
Prevalence **(A)**, bias **(B)**, and physicochemical property change **(C)** of amino acid changes in untreated PLWH.

The most prevalent amino acid change was from threonine (T) to alanine (A), followed by from A to T and from isoleucine (I) to valine (V) (**Figure 4A**). Marked bias towards amino acids changing to A and T were observed, suggesting a higher proportion of all possible changes to these two amino acids. By contrast, the other amino acids showed a relatively linear relationship between the observed number and total number of changes to a new amino acid, indicating that these variations were occurring at a roughly equal rate (**Figure 4B**). The most frequent alteration in the acidity and polarity properties of amino acids was from neutral apolar to neutral polar (25% or 117/460) (**Figure 4C**). Extensive profiles concerning the hydropathy, volume, chemical, charge, hydrogen donor or acceptor atoms, and polarity of amino acid replacements (61) are shown in **Supplementary Table S4**.

### Gender-, Age-, and Immune Level-Related Mutational Patterns of mtDNA in 806 ART-Naive Han Ethnic PLWH

**Figure 5:**
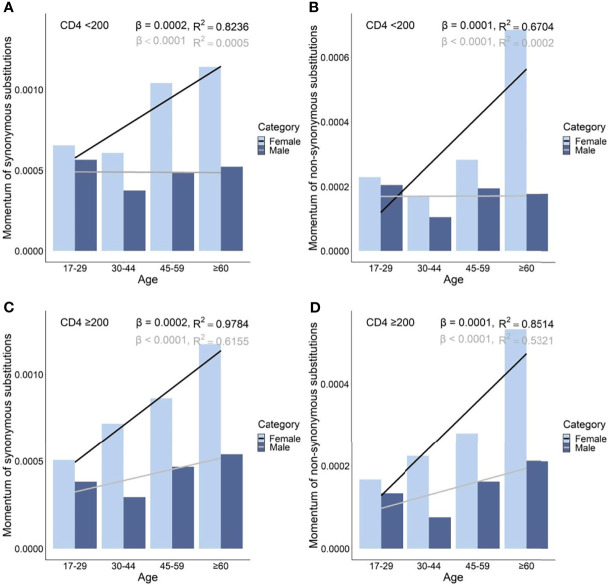
Momentum in diversity of synonymous **(A)**, and non-synonymous **(B)** substitutions in untreated PLWH with severe immunodeficiency and of synonymous **(C)** and non-synonymous **(D)** substitutions in untreated PLWH with mild immunodeficiency.

At each age and immune level, female PLWH had stronger momentum in both synonymous and nonsynonymous substitutions than male PLWH (**Figure 5**) . On average, female PLWH showed ~1.86-fold and ~2.06-fold stronger momentum of synonymous and nonsynonymous substitutions than age- and immune level-matched male PLWH. In PLWH with severe immunodeficiency, females showed more radical age-related uptrends of momentum of synonymous and nonsynonymous substitutions, compared with male counterparts (synonymous: β_female_ = 2 x 10^−4^ *vs.* β_male_ = −1 x 10^−6^; nonsynonymous: β_female_ = 1 x 10^−4^ *vs.* β_male_ = 5 x 10^−7^). By contrast, in PLWH with mild immunodeficiency, both females and males showed age-related uptrends of momentum of synonymous and nonsynonymous substitutions, though the extent of uptrends was more intense in females (synonymous: β_female_ = 2 x 10^−4^ *vs.* β_male_ = 6 x 10^−5^; nonsynonymous: β_female_ = 1 x 10^−4^ *vs.* β_male_ = 3 x 10^−5^; **Figure 5** and **Supplementary Table S5**).

**Figure 6:**
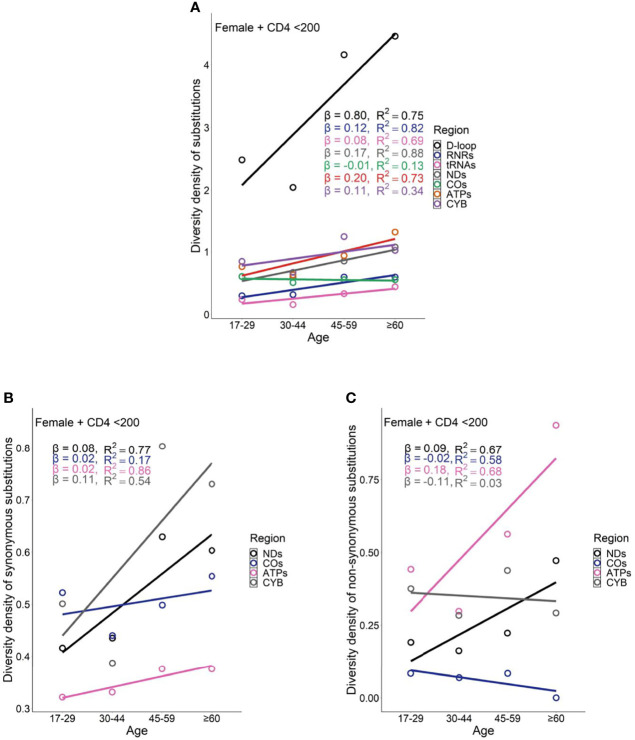
Age-related trend in diversity density of substitutions **(A)**, synonymous substitutions **(B)**, and non-synonymous substitutions **(C)** in untreated female PLWH with server immunodeficiency.

**Figure 7:**
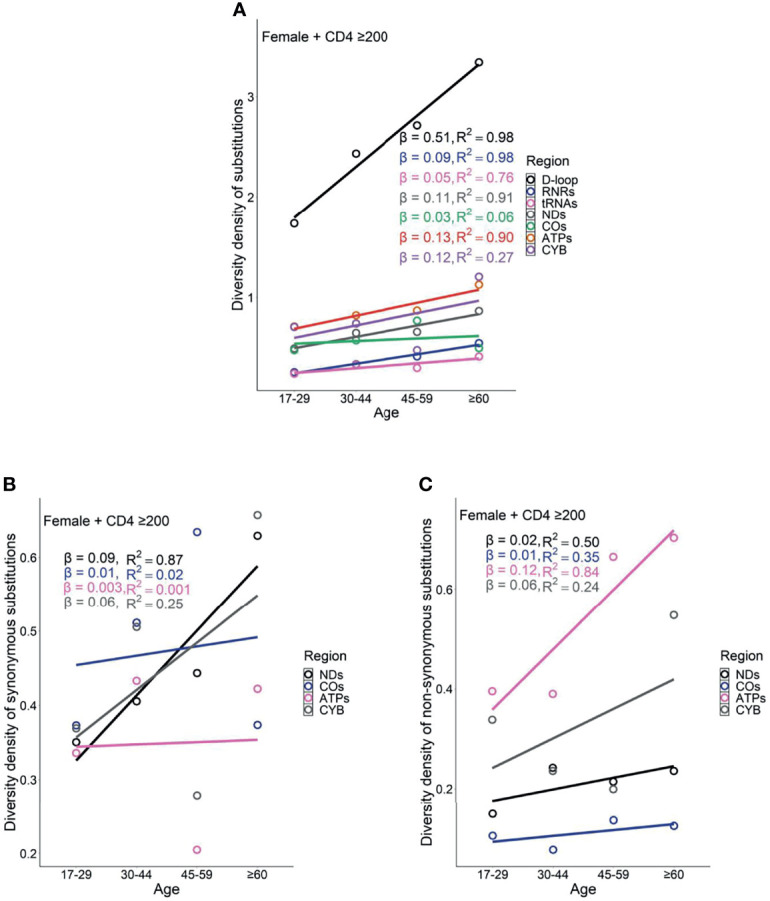
Age related trend in diversity density of substitutions **(A)**, synonymous substitutions **(B)**, and non-synonymous substitutions **(C)** in untreated female PLWH with mild immunodeficiency.

**Figure 8:**
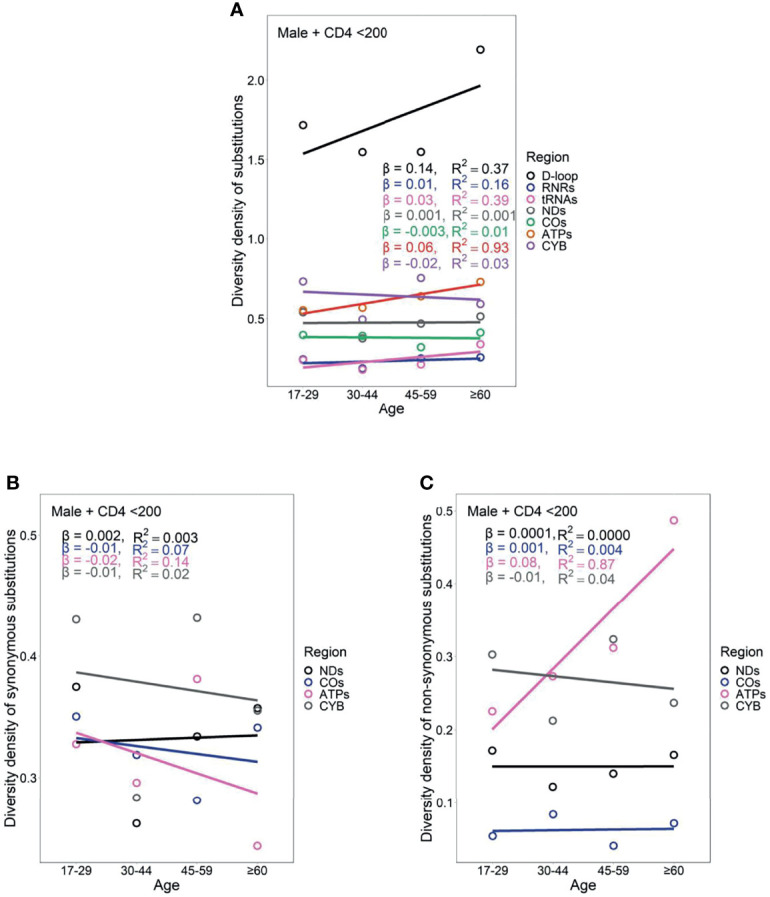
Age-related trend in diversity density of substitutions **(A)**, synonymous substitutions **(B)**, and non-synonymous substitutions **(C)** in untreated male PLWH with severe immunodeficiency.

**Figure 9:**
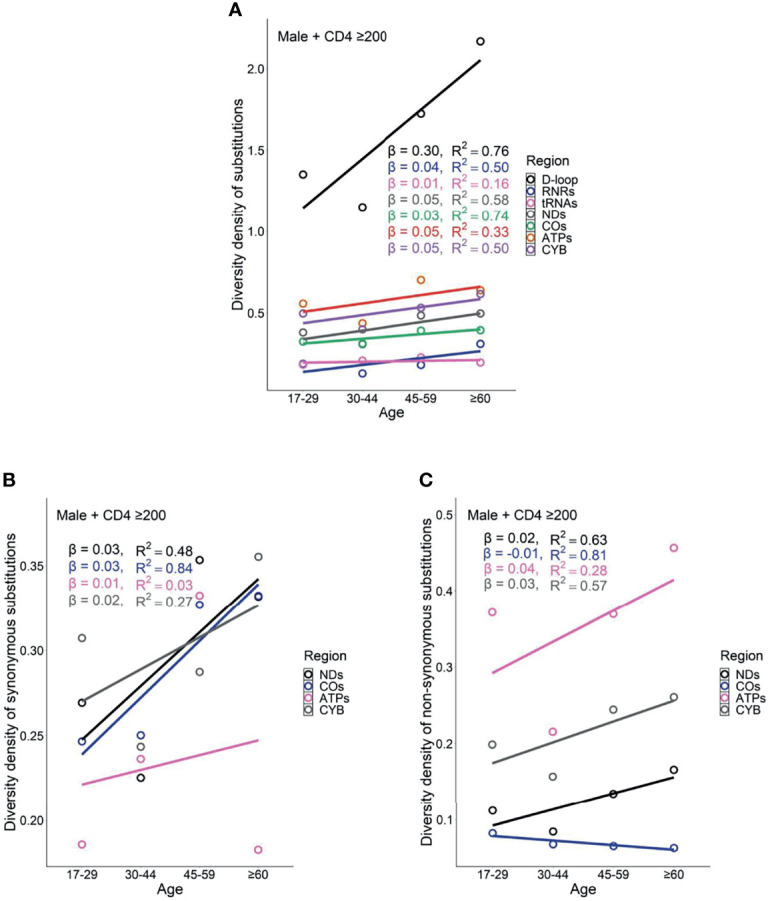
Age-related trend in diversity density of substitutions **(A)**, synonymous substitutions **(B)**, and non-synonymous substitutions **(C)** in untreated male PLWH with mild immunodeficiency.

Female PLWH with severe immunodeficiency, compared with female PLWH with mild immunodeficiency, experienced more radical age-related uptrends in diversity density of substitutions in D-loop (β_CD4 <200_ = 0.80 *vs.* β_CD4 ≥200_ = 0.51), RNRs (β_CD4 <200_ = 0.12 *vs.* β_CD4 ≥200_ = 0.09), tRNAs (β_CD4 <200_ = 0.08 *vs.* β_CD4 ≥200_ = 0.05), NDs (β_CD4 <200_ = 0.17 *vs.* β_CD4 ≥200_ = 0.11), and ATPs (β_CD4 <200_ = 0.20 *vs.* β_CD4 ≥200_ = 0.13; **Figures 6A** and **7A**). This contrast applied to the age-related uptrends in diversity density of synonymous substitutions for ATPs (β_CD4 <200_ = 0.020 *vs.* β_CD4 ≥200_ = 0.003), and CYB (β_CD4 <200_ = 0.11 *vs.* β_CD4 ≥200_ = 0.06; **Figures 6B** and **7B**), and nonsynonymous substitutions for NDs (β_CD4 <200_ = 0.09 *vs.* β_CD4 ≥200_ = 0.02) and ATPs (β_CD4 <200_ = 0.18 *vs.* β_CD4 ≥200_ = 0.12; **Figures 6C** and **7C**). By contrast, male PLWH with mild immunodeficiency, compared with male PLWH with severe immunodeficiency, showed more radical age-related uptrends in diversity density of substitutions in D-loop (β_CD4 ≥200_ = 0.30 *vs.* β_CD4 <200_ = 0.14), RNRs (β_CD4 ≥200_ = 0.04 *vs.* β_CD4 <200_ = 0.01), NDs (β_CD4 ≥200_ = 0.050 *vs.* β_CD4 <200_ = 0.001), COs (β_CD4 ≥200_ = 0.030 *vs.* β_CD4 <200_ = −0.003), and CYBs (β_CD4 ≥200_ = 0.05 *vs.* β_CD4 <200_ = −0.02) (**Figures 8A** and **9A**). The former population also showed more radical age-related uptrends in diversity density of synonymous substitutions for NDs (β_CD4 ≥200_ = 0.030 *vs.* β_CD4 <200_ = 0.002) and COs (β_CD4 ≥200_ = 0.03 *vs.* β_CD4 <200_ = −0.01; **Figures 8B** and **9B**), and nonsynonymous substitutions for NDs (β_CD4 ≥200_ = 0.02 *vs.* β_CD4 <200_ = 1 x 10^−4^) and CYBs (β_CD4 ≥200_ = 0.03 *vs.* β_CD4 <200_ = −0.01; **Figures 8C** and **9C**).

Female PLWH with severe immunodeficiency, compared with male PLWH with severe immunodeficiency, carried more radical age-related uptrends of diversity density of substitutions in more mtDNA divisions including D-loop (β_female_ = 0.80 *vs.* β_male_ = 0.14), RNRs (β_female_ = 0.12 *vs.* β_male_ = 0.01), tRNAs (β_female_ = 0.08 *vs.* β_male_ = 0.03), NDs (β_female_ = 0.170 *vs.* β_male_ = 0.001), ATPs (β_female_ = 0.20 *vs.* β_male_ = 0.06), and CYB (β_female_ = 0.11 *vs.* β_male_ = −0.02; **Figures 6A** and **8A**). This contrast applied to the age-related uptrends of diversity density of synonymous substitutions for NDs (β_female_ = 0.080 *vs.* β_male_ = 0.002), ATPs (β_female_ = 0.02 *vs.* β_male_ = −0.02), and CYB (β_female_ = 0.11 *vs.* β_male_ = −0.01; **Figures 6B** and **8B**), and nonsynonymous substitutions for NDs (β_female_ = 0.09 *vs.* β_male_ = 1 x 10^-4^) and ATPs (β_female_ = 0.18 *vs.* β_male_ = 0.08; **Figure 6C** and **8C**), respectively. These results were aligned with age-related uptrends of momentum of synonymous and nonsynonymous substitutions in females and males with severe immunodeficiency (**Figures 5A, B**).

However, the remarkable differences between females and males with severe immunodeficiency in age-related mutational uptrends were weakened between females and males with mild immunodeficiency. Smaller differences between females and males with mild immunodeficiency in age-related uptrends of substitutions were shown in D-loop (β_female_ = 0.51 *vs.* β_male_ = 0.30), RNRs (β_female_ = 0.09 *vs.* β_male_ = 0.04), NDs (β_female_ = 0.11 *vs.* β_male_ = 0.05), and ATPs (β_female_ = 0.13 *vs.* β_male_ = 0.05; **Figures 7A** and **9A**), synonymous substitutions for NDs (β_female_ = 0.09 *vs.* β_male_ = 0.03; **Figures 7B** and **9B**), and nonsynonymous substitutions for NDs (β_female_ = 0.02 *vs.* β_male_ = 0.02) and COs (β_female_ = 0.01 *vs.* β_male_ = -0.01; **Figures 7C** and **9C**), respectively. These results were aligned with age-related uptrends of momentum of synonymous and nonsynonymous substitutions in females and males with mild immunodeficiency (**Figures 5C, D**). Other interesting mtDNA features are shown in the **Supplementary Text 1**.

### Associations of mtDNA Substitutions With Pre-ART CD4+T-Cell Counts and Changes From Pre-ART to Post-ART in the CD4+T-Cell Count of PLWH

After adjustments of gender, age, ethnicity, and transmission mode, m.14470T>C (*p* = 0.019) and m.16362T>C (*p* = 0.016) were negatively associated with pre-ART CD4+T-cell count. After adjustments of gender, age, ethnicity, transmission mode, pre-ART CD4+T-cell count, ART regimens, and treatment duration, m.1005T>C (*p* = 0.005), m.1824T>C (*p* = 0.025), m.3394T>C (*p* = 0.006), m.4491G>A (*p* = 0.013), m.7828A>G (*p* = 0.025), m.9814T>C (*p* = 0.008), m.10586G>A (*p* = 0.025), m.12338T>C (*p* = 0.025), m.13708G>A (*p* = 0.035), and m.14308T>C (*p* = 8.22 × 10^−5^) were negatively associated with changes from pre-ART to post-ART in the CD4+T-cell count. Of them, m.14308T>C in ND6 conferred risks for CD4+T-cell recovery at the Bonferroni-corrected significance (nominal *p*-value <8.71 × 10^−5^ given 574 tests using the same dataset). By contrast, m.93A>G (*p* = 0.003), m.15218A>G (*p* = 0.030), and m.16399A>G (*p* = 0.010) were positively associated with changes from pre-ART to post-ART in the CD4+T-cell count (**Table 2** and **Supplementary Tables S6, S7**).

**Table 2:** Significant associations of mtDNA substitutions with CD4+T-cell counts in mitochondrial genome-wide association analyses.

Substitution	Region	*β*	*p*	95% CI	SE	*t*
Pre-ART CD4+T cell counts						
m.14470T>C	*ND6*	−73.67	0.019	(−135.40, −11.94)	−2.34	31.44
m.16362T>C	*D-loop*	−24.29	0.016	(−43.94, −4.63)	−2.43	10.01
Changes from pre-ART to post-ART in the CD4+T cell count						
m.93A>G	*D-loop*	49.56	0.003	(17.08, 82.05)	16.55	3.00
m.1005T>C	*RNR1*	−50.97	0.005	(−86.79, −15.14)	18.25	−2.79
m.1824T>C	*RNR2*	−44.14	0.025	(−82.59, −5.69)	19.59	−2.25
m.3394T>C	*ND1*	−41.05	0.006	(−70.32, −11.78)	14.91	−2.75
m.4491G>A	*ND2*	−47.81	0.013	(−85.56, −10.07)	19.22	−2.49
m.7828A>G	*CO2*	−44.14	0.025	(−82.59, −5.69)	19.59	−2.25
m.9814T>C	*CO3*	−43.49	0.008	(−75.43, −11.69)	16.27	−2.67
m.10586G>A	*ND4L*	−44.14	0.025	(−82.59, −5.69)	19.59	−2.25
m.12338T>C	*ND5*	−44.14	0.025	(−82.59, −5.69)	19.59	−2.25
m.13708G>A	*ND5*	−38.73	0.035	(−74.72, −2.69)	18.33	−2.11
m.14308T>C	*ND6*	−56.47	8.22 x 10−^5^	(−84.46, −28.48)	−3.96	14.26
m.15218A>G	*CYB*	70.49	0.030	(6.86, 134.11)	2.17	32.41
m.16399A>G	*D-loop*	145.12	0.010	(35.20, 255.05)	2.59	55.99

β, slope; 95% CI, 95% confidence interval; SE, standard error.

## Discussion

This work comprehensively characterizes the mutational features of mtDNA and identifies the contributions of mtDNA mutations to short-term CD4+T-cell recovery in Chinese PLWH. We elucidate the roles of single-nucleotide variants across mtDNA genomes in CD4+T-cell recovery within short-term antiretroviral medications on the basis of a prospective cohort of PLWH who started ART immediately after being newly diagnosed as HIV seropositivity. Eight hundred fifty-six full-length mtDNA sequences of East-Asian ancestry may add diversity to the global mtDNA genome datasets and benefit other associative mtDNA genetic analyses to evoke the medical relevance for East-Asian PLWH.

Because of diverse characterization metrics and a relatively large sample size, we first demonstrate that female PLWH rather than male PLWH carried stronger momentum in mutational diversity of nonsynonymous substitutions and female PLWH with severe immunodeficiency rather than male counterparts showed more radical age-related uptrends in nonsynonymous momentum and nonsynonymous diversity for mtDNA protein-coding genes such as NDs and ATPs. These results suggest mutational processes of mtDNA may be more exacerbated in female PLWH than male PLWH, which may predispose female PLWH to mtDNA aberrancy-related pathological conditions such as aging and age-related NCDs. Our identification of female PLWH as a risky population of aging and age-related NCDs inferred from its distinctive mtDNA features, coupled with premature aging in the female PLWH rather than male PLWH inferred from subjective complaints (62), may help the development of gender-based strategies for complicated HIV patient care due to CD4+T-cell loss. In general, females are prone to healthier aging and longer lifespans than males, because of efficient oxidative phosphorylation and low generation of reactive oxygen species (ROS) mediated by estrogen (63). However, compared with male PLWH, female PLWH was estimated to have lower life expectancy and momentum (64), which cannot be easily attributed to the previously defined role of estrogen in shaping the redox features. Given that mitochondrial oxidative stress amassed in the aging process interacts with mtDNA maintenance (65, 66), intensified mtDNA mutational diversity in female PLWH observed in our study, coupled with female-biased enrichment of mitochondrial oxidative stress (67–69) may provide an alternative explanation to the abnormal disparities of aging and life expectancy in the two sexes of PLWH.

Interestingly, we observe correlations between mild immunodeficiency and more radical age-related mutational uptrends in male PLWH, while this observation does not apply to female PLWH. In response to exacerbated mitochondrial dysfunction under severer immunodeficiency (70) and older age (71), nonselective proliferation of mtDNA occur to eliminate mutations in nondividing cells (72). Inferring from those findings, we speculate that male PLWH with severe immunodeficiency may enrich the stronger random genetic drift to purify mtDNA mutations whereas female counterparts may lack this purification mechanism. Studies on characteristics of mtDNA genetic drift between two sexes are needed to examine our speculation.

To pinpoint the contributions of mtDNA mutations to short-term CD4+T-cell recovery systematically and reliably, we probed single-nucleotide variants across the complete mtDNA sequences by excluding the potential bias from age, gender, ethnicity, transmission mode, pre-ART CD4+T-cell count, ART regimens, and ART treatment duration in the regression analyses, which have not been achieved in the previous Euromerican studies adequately (14, 33–35). A majority of single-nucleotide variants defining statistically significant haplogroups in Euromerican PLWH, for example, m.3010 G>A, m.14798T>C, and 15257G>A of haplogroup J (14, 35), m.14793A>G of haplogroup U5a, m.14798T>C of haplogroup Uk (14), polymorphisms at mtDNA nucleotide positions 2789, 7175, 7274, 7771, 9221, 10115, 11914, 13590, 13803, 14566, and 16390 of haplogroup L2 (33), polymorphisms at 4577 and 7025 of haplogroup HV (34, 35), and polymorphisms at 13366 and 13704 of haplogroup JT (35)), were not statistically significant in Chinese PLWH of this study. A variant m.15218A>G of haplogroup U5a1 (14) exerted an opposite protective effect on CD4+T-cell counts in Chinese PLWH. To our surprise, no matter in Euromerican PLWH (14) or our Chinese PLWH, 3394T>C and m.13708G>A of haplogroup J were negatively associated with CD4+T-cell counts. Many mtDNA haplogroups or single-nucleotide variants in Euromerican PLWH did not reach Bonferroni-corrected significance. Here, we show a variant m.14308T>C in *ND6* which conferred statistically significant risks for CD4+T-cell recovery after Bonferroni correction. m.14308T>C is a synonymous substitution which uses a tRNA anticodon CCU rather than the wild-type CCC. Differences in tRNA abundance may underlie the negative effect of this variant on CD4+T-cell recovery in response to ART (73).

We show that the ND region carried over half (six of 10) of statistically significant mutations associated with poor CD4 cell recovery. Mutations in the ND region may regulate CD4+T-cell counts through the disrupted pyroptosis and apoptosis processes in a HIV-related ROS-rich environment. Defects in *ND1* and *ND6* cause ROS overproduction (74, 75). ROS toxicity interacts with the mitochondrial-associated inflammasome, which promotes the binding between absent in melanoma 2 (AIM2) and mtDNA, where caspase 1, known to mediate pyroptosis that contributes to the death of over 95% of quiescent CD4+T cells by abortive viral infection, is further activated (76, 77). ROS-induced oxidative stress promotes the mitochondrial permeability transition pore, which triggers the release of cytochrome *c* and the conversion from procaspase-3 to caspase 3 (78). Caspase 3 facilitates the apoptosis of productively infected CD4+T cells (76). Moreover, HIV-1-encoded proteins can lead to the excessive amount of ROS (79) and trigger mitochondrial membrane permeabilization, an event of HIV-1-induced apoptosis (80). Interestingly, a variant m.3394T>C in *ND1* risky for short-term CD4+T-cell recovery which was found in our regression analyses contributes to the development of East-Asian metabolic syndrome and type 2 diabetes mellitus (81–84). Given that our study is a preliminary report of mtDNA mutation-CD4+T-cell recovery associations, more *in vitro* and *in vivo* studies are needed to examine the functional mechanisms behind those associations, which may relate to the cyto-destructive nature of mtDNA mutations in up-regulating ROS, and connections of those associations and mechanisms to the development of chronic diseases in the HIV context.

We show that ART-naive female PLWH had age-related uptrends in mtDNA mutational momentum and diversity, consistent with the hypothesis that mtDNA mutations may be a key molecular mechanism for aging (85–87), and the phenomenon that mtDNA mutational burdens increase with ages in female PLWH (88). Similar to general populations (89–91) and HIV-infected populations (25, 26, 92), we show that D-loop harbored the highest mutational diversities and volumes in 856 ART-naïve PLWH. Similar to mutational characteristics derived from 5,140 human mtDNA (93), we show that the observed number of types of synonymous substitutions rather than nonsynonymous substitutions linearly correlated with the maximum possible changes; a higher proportion of all possible changes occurred in A and T; and the most prevalent acidity and polarity change was from neutral apolar to neutral polar.

### Limitations

Small number of participants in some subpopulations needs additional replication of our findings in large-scale cohort studies that ensure the sufficient sample size after stratification by ethnicity, age, gender, and immune level. Furthermore, comparable gender-, age-, ethnicity-matched HIV-negative populations need to be included so that it is possible to distinguish mtDNA mutational patterns between general populations and PLWH precisely.

## Conclusion

We report baseline mtDNA characteristics in ART-naïve Chinese PLWH, showing different levels of gender, age, and immunity present diverse mtDNA mutational features. Specially, females rather than males bore more rise in mutational momentum and diversity with increasing age and experienced a remarkable correlation between severer immunodeficiency and age-related uptrends of mutational diversity in many mtDNA regions. Given that mtDNA plays an important role in aging and age-related NCDs, our results stress the attention to East-Asian female PLWH who may be at risk of CD4+T-cell loss-related aging and NCDs. This may further evoke the applications of mtDNA screening and intervention to personalize the complicated HIV patient management due to CD4+T-cell loss. Furthermore, mtDNA variants identified in regression analyses explain the variability of short-term CD4+T-cell recovery of East-Asian PLWH. Whether statistically significant relationships in our mtDNA genome-wide association study are causal remains to be examined by the functional mechanism experiments, but our finding clearly highlights the potential of mtDNA in informing immune recovery strategies in PLWH.

## Data Availability Statement

The original contributions presented in the study are publicly available. This data can be found here: GenBank, accession numbers OL697409 - OL697708.

## Ethics Statement

The studies involving human participants were reviewed and approved by the Institutional Review Board of Fudan University. Written informed consent to participate in this study was provided by the participants’ legal guardian/next of kin.

## Author Contributions

AL and NH proposed and developed the research question. AL designed and performed the bioinformatics and statistical analyses. AL and NH wrote, reviewed, and edited the manuscript. NH generally designed and supervised the study. QW and DZ performed the entire mtDNA sequencing experiments. HL and YD supervised field investigation, data management, and reviewed the manuscript. YS advised on bioinformatics analyses and reviewed and edited the manuscript. JH, ZM, SZ, XC, WS, and MG contributed to data collection. FL contributed to bioinformatics analyses. All authors contributed to the article and approved the submitted version.

## Funding

This work was supported by the China National Science and Technology Major Projects on Infectious Diseases (2018ZX10721102-004) and the National Natural Science Foundation of China (81773485, 81872671, 81803291) and partially supported by the Shanghai Municipal Health Commission (GWV-10.1-XK16; GWTD2015S05).

## Conflict of Interest

The authors declare that the research was conducted in the absence of any commercial or financial relationships that could be construed as a potential conflict of interest.

## Publisher’s Note

All claims expressed in this article are solely those of the authors and do not necessarily represent those of their affiliated organizations, or those of the publisher, the editors and the reviewers. Any product that may be evaluated in this article, or claim that may be made by its manufacturer, is not guaranteed or endorsed by the publisher.
