# Supplementary material for: Baseline Characteristics of Mitochondrial DNA and Mutations Associated With Short-Term Posttreatment CD4+T-Cell Recovery in Chinese People With HIV
Source: Front Immunol. 2021 Dec 14;12:793375. doi: 10.3389/fimmu.2021.793375 (PMC8712318; doi:10.3389/fimmu.2021.793375)
Supplement: Supplementary file 1 [file DataSheet_1.zip › SupplementaryMaterial/Supplementary Figure1.docx]

| Substitutions | | | |
| --- | --- | --- | --- |
| **A**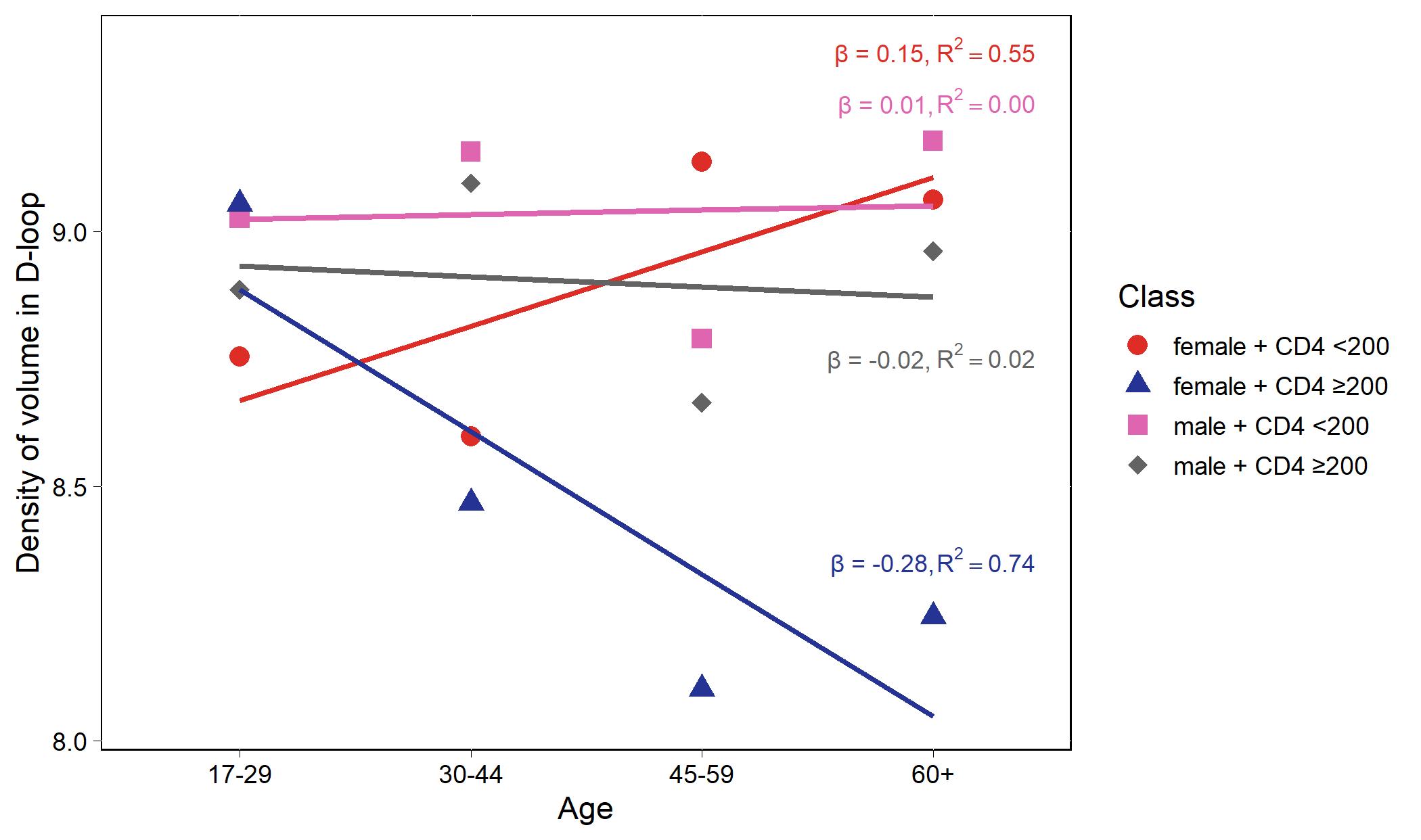 | **B** 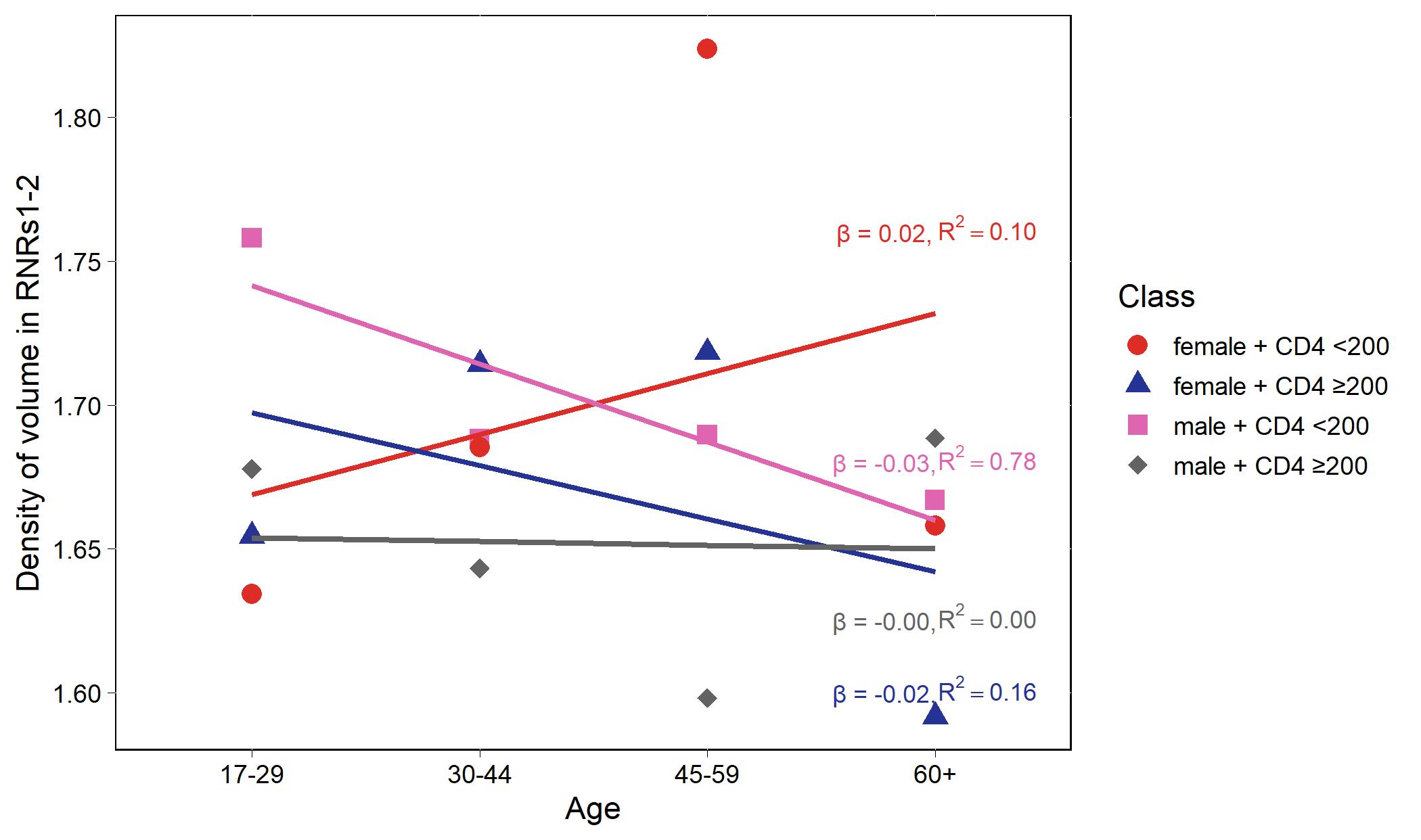 | **C** 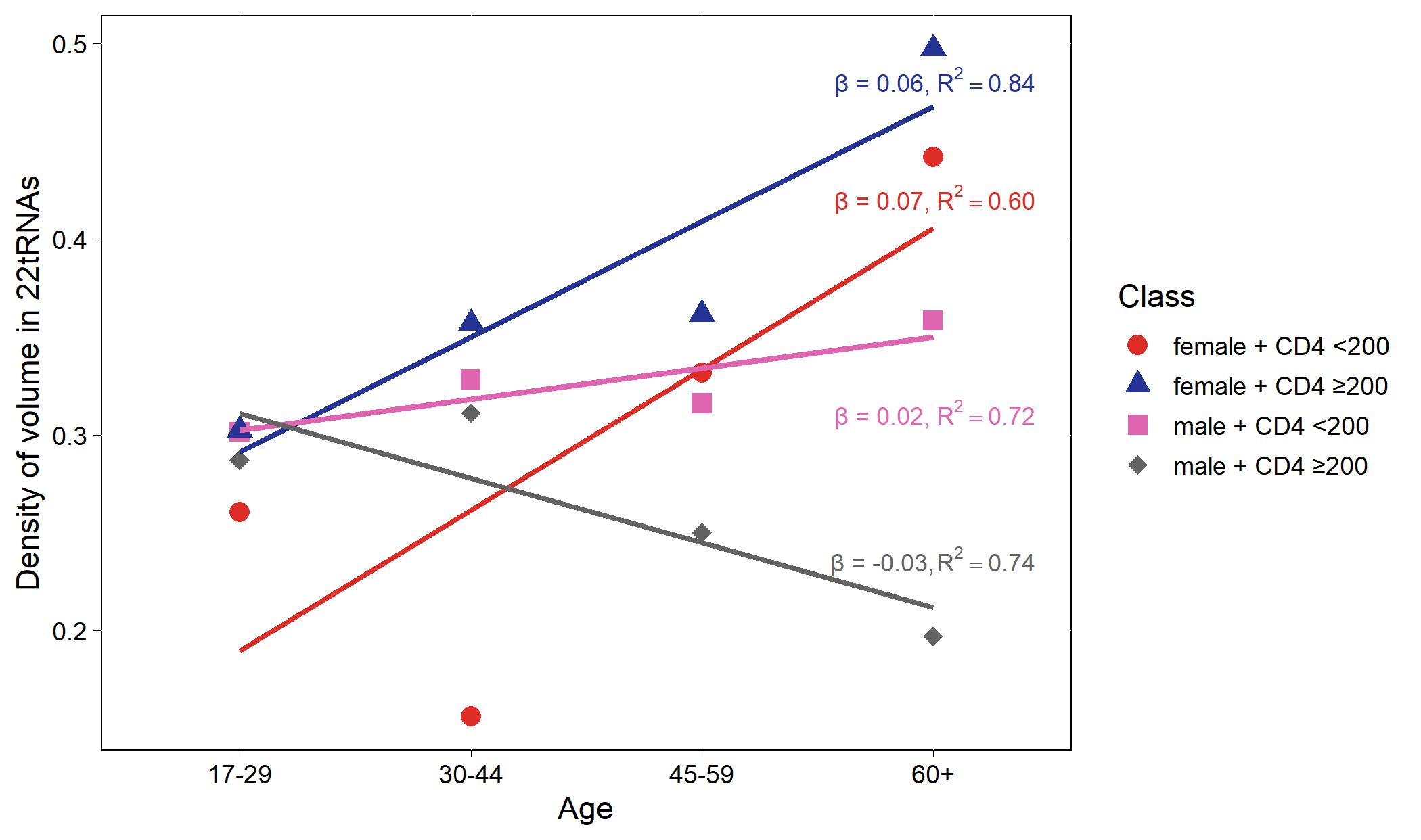 | **D**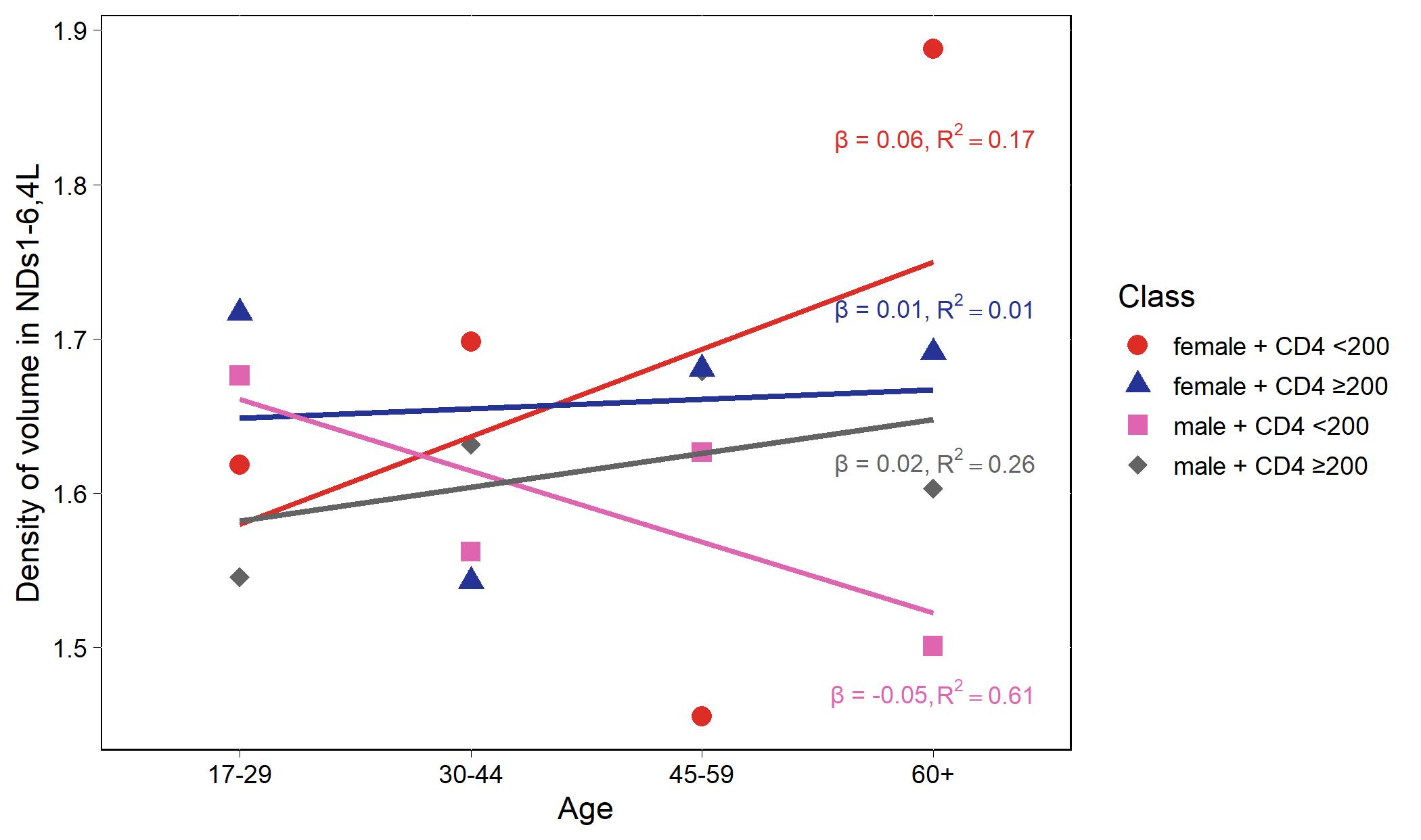 |
| **E** 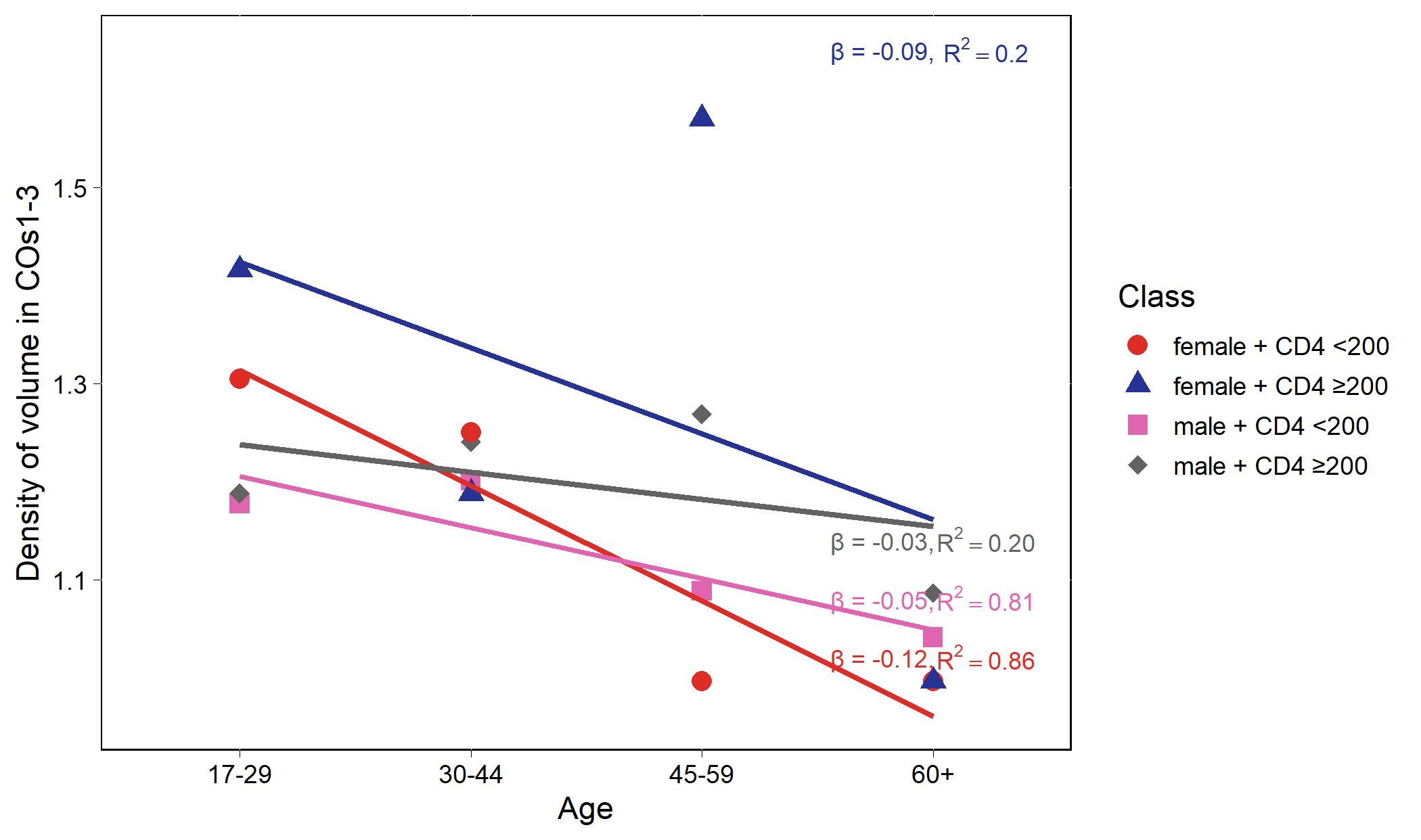 | **F** 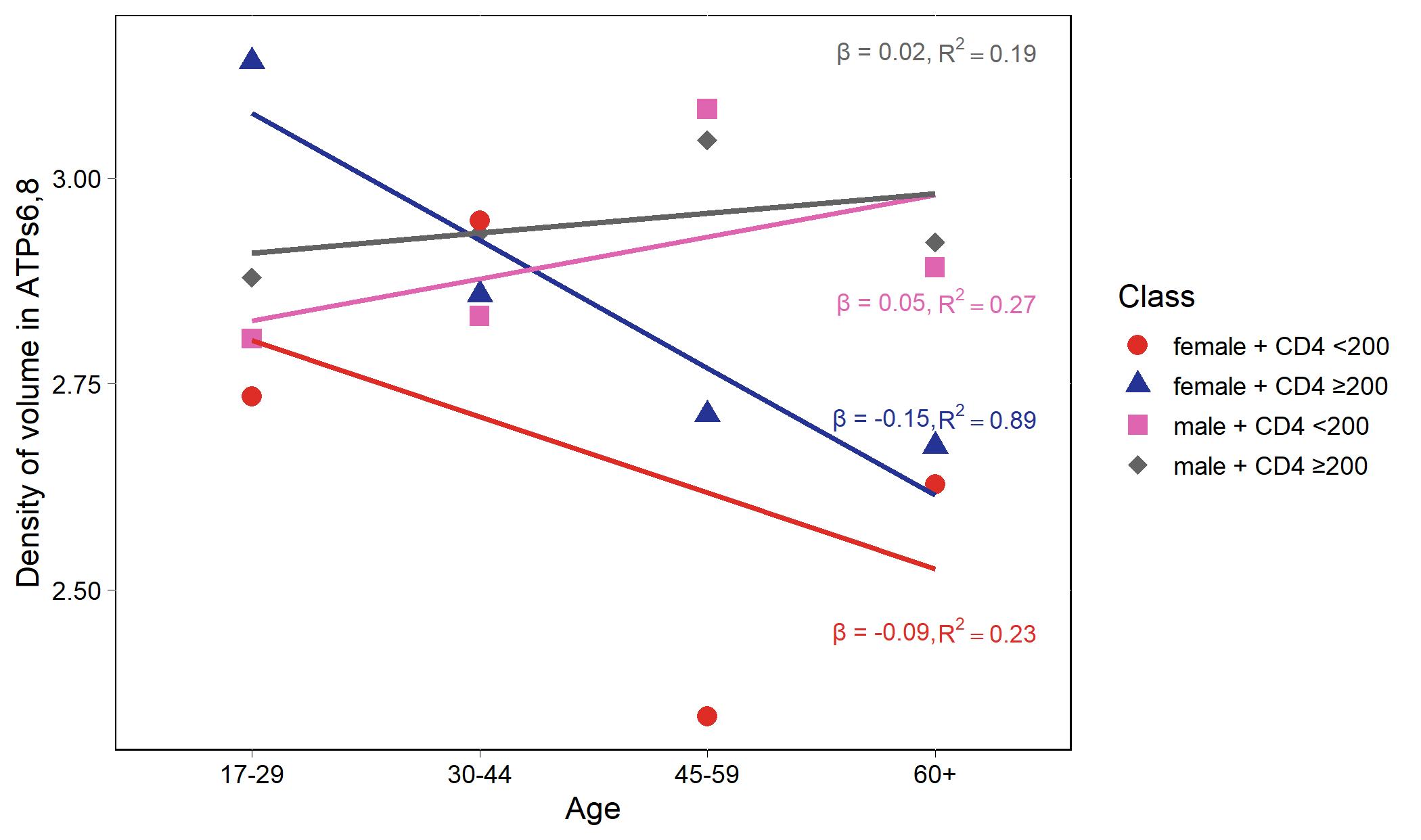 | **G** 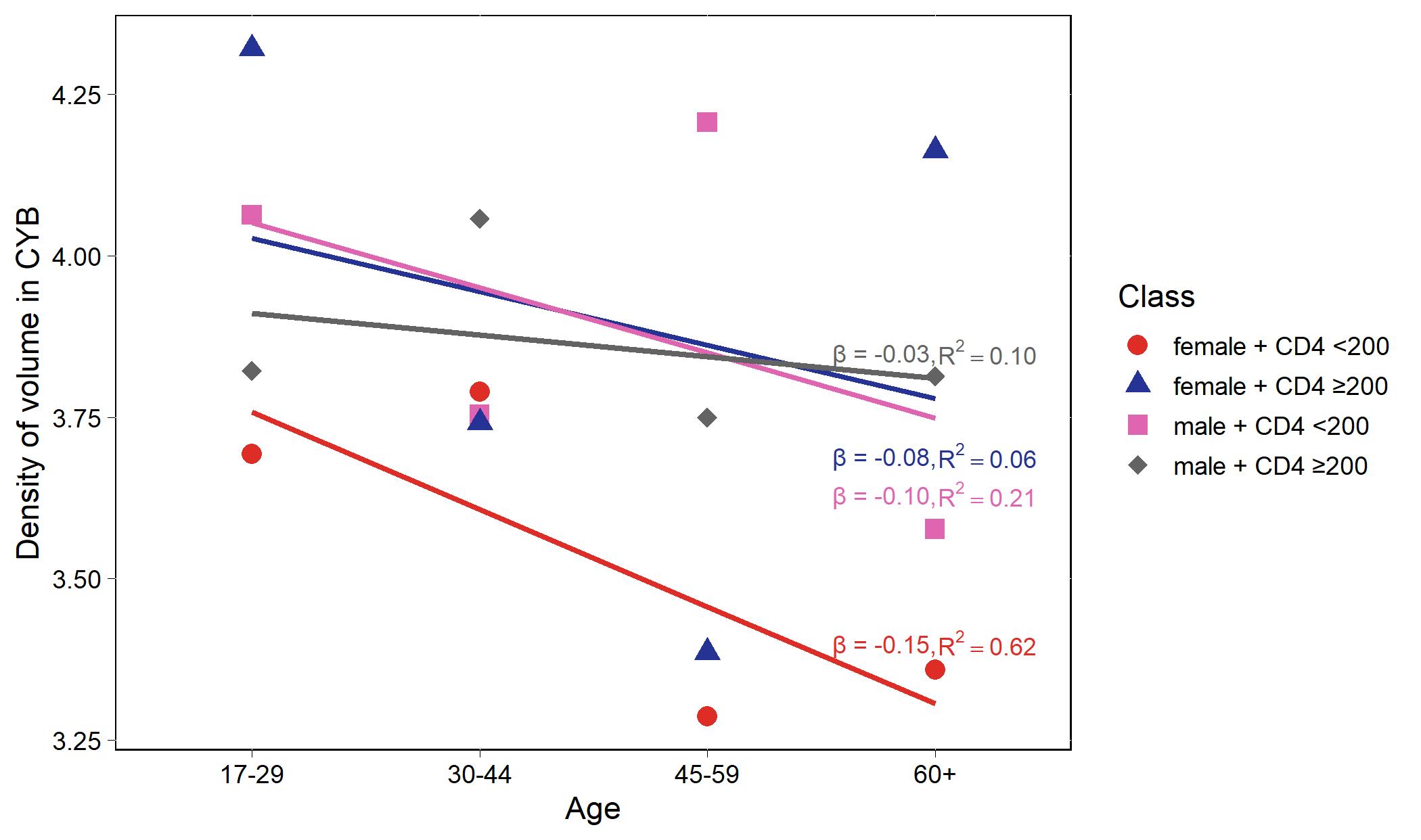 |  |
| Synonymous substitutions | | | |
| **H** 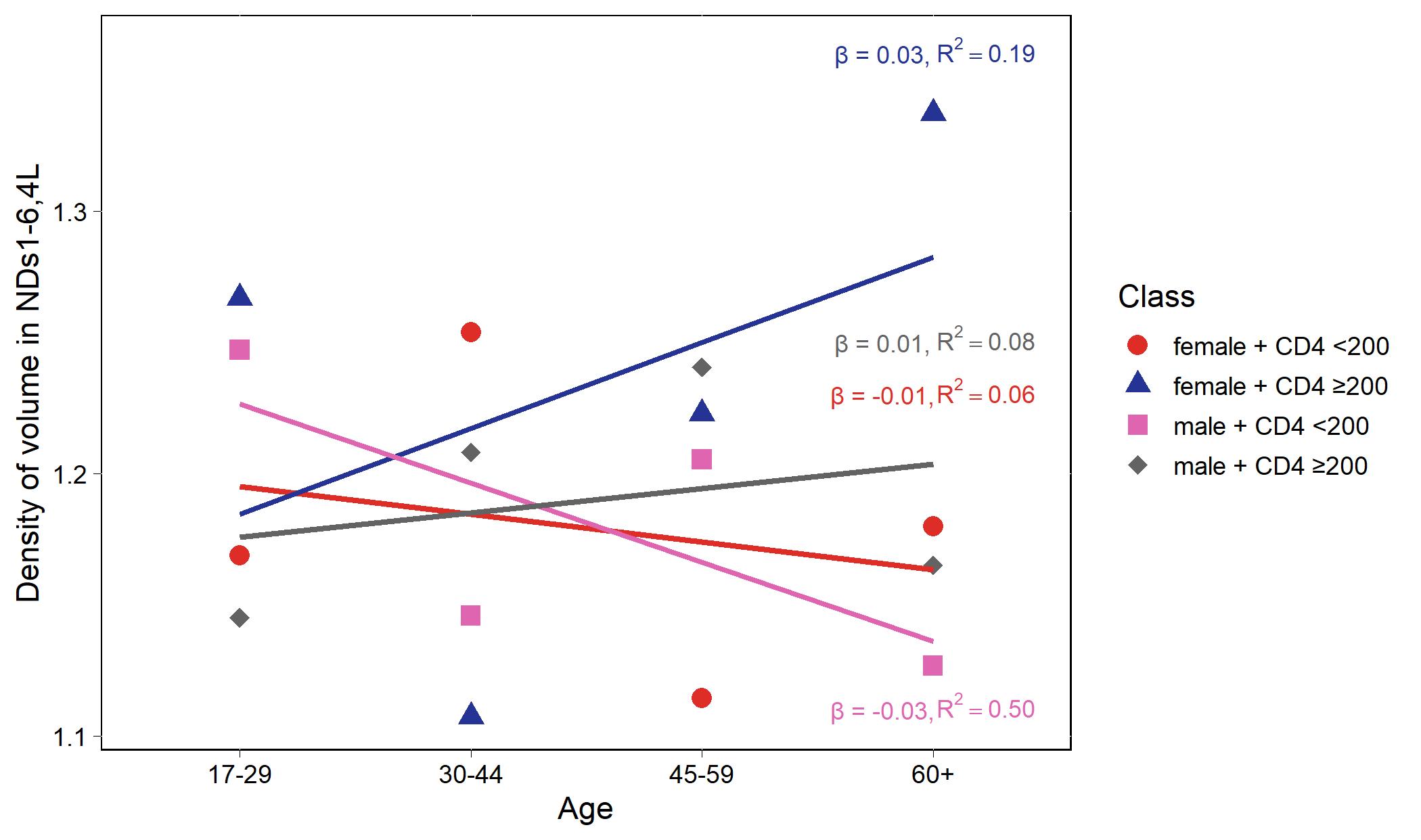 | **I** 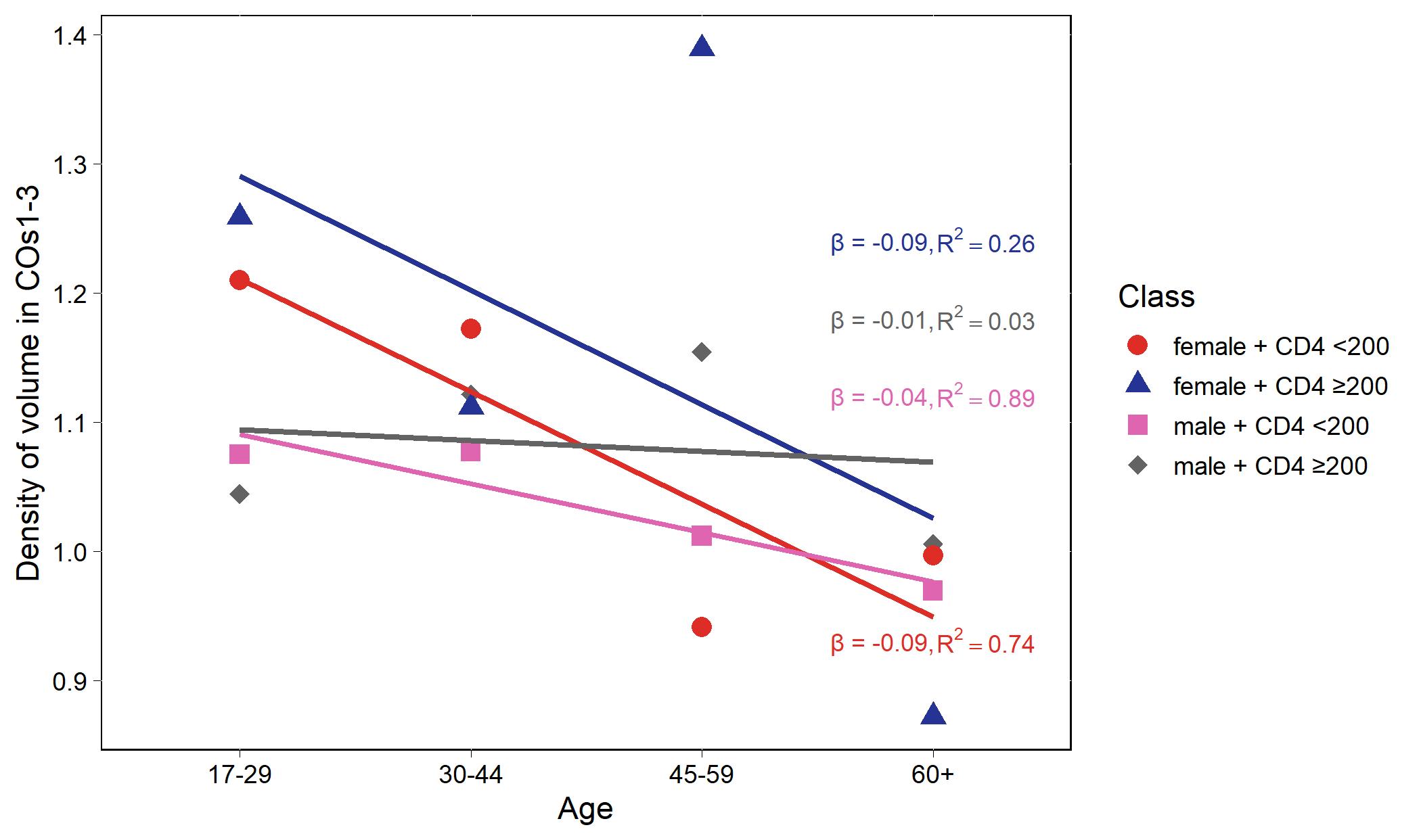 | **J** 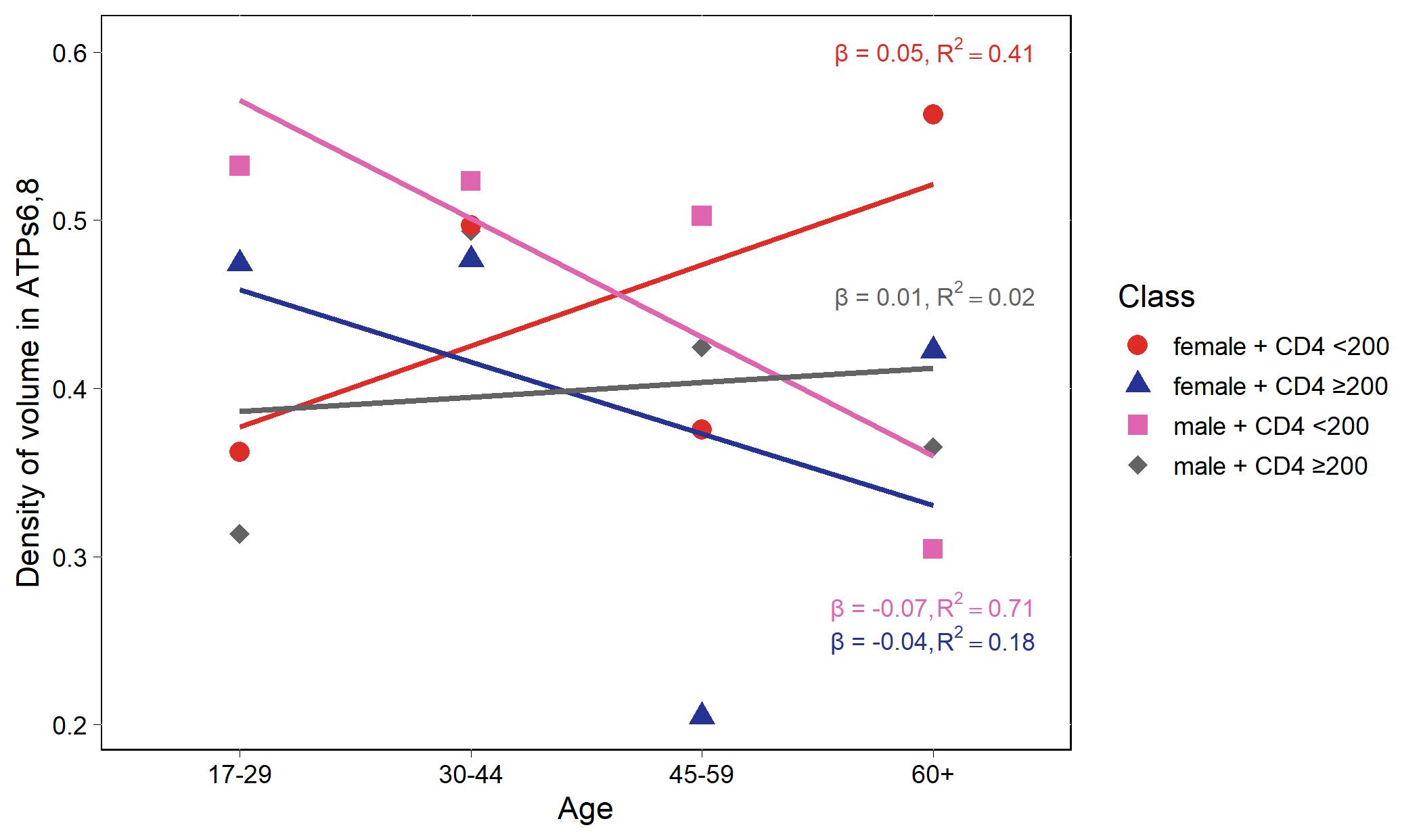 | **K**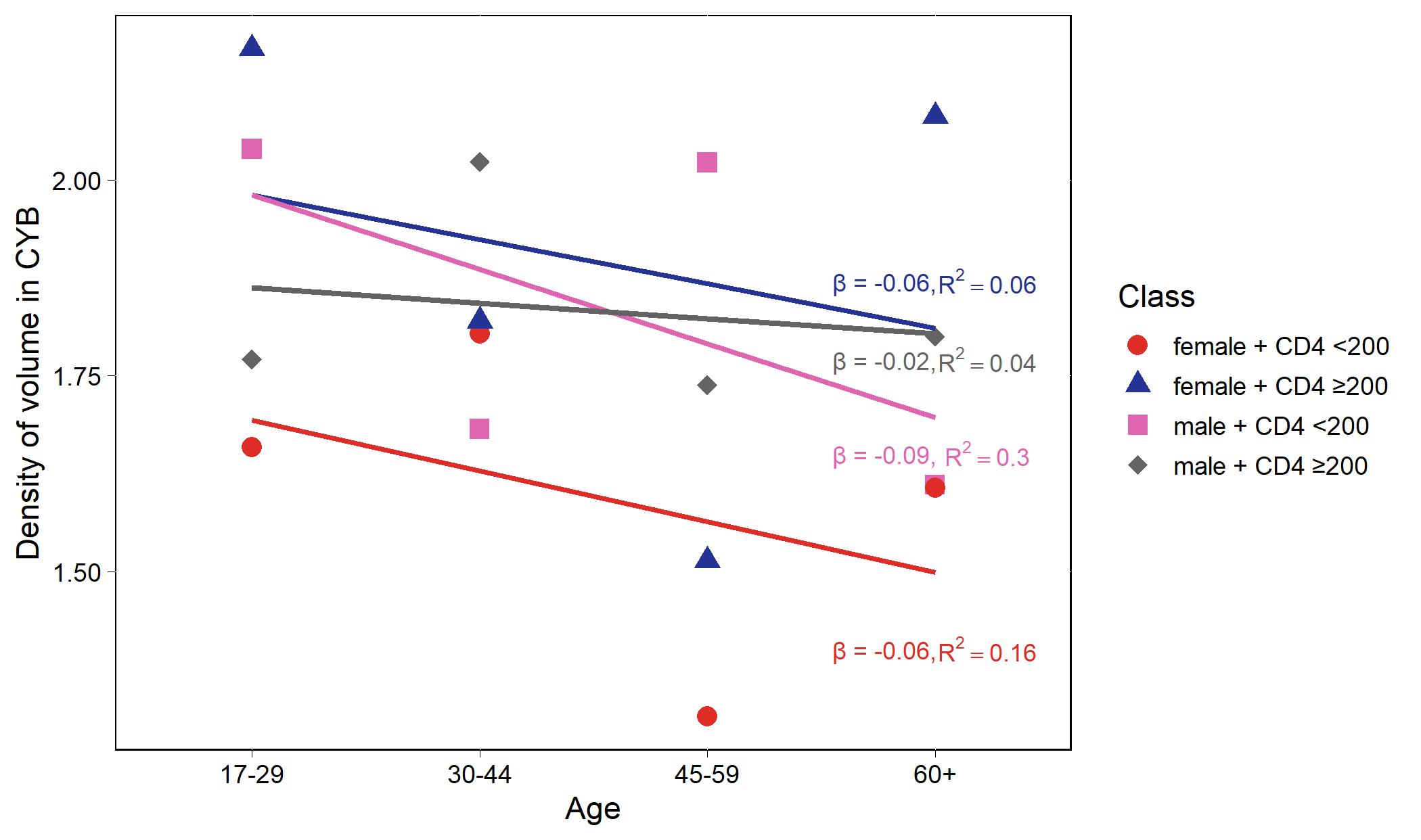 |
| Non-synonymous substitutions | | | |
| **L**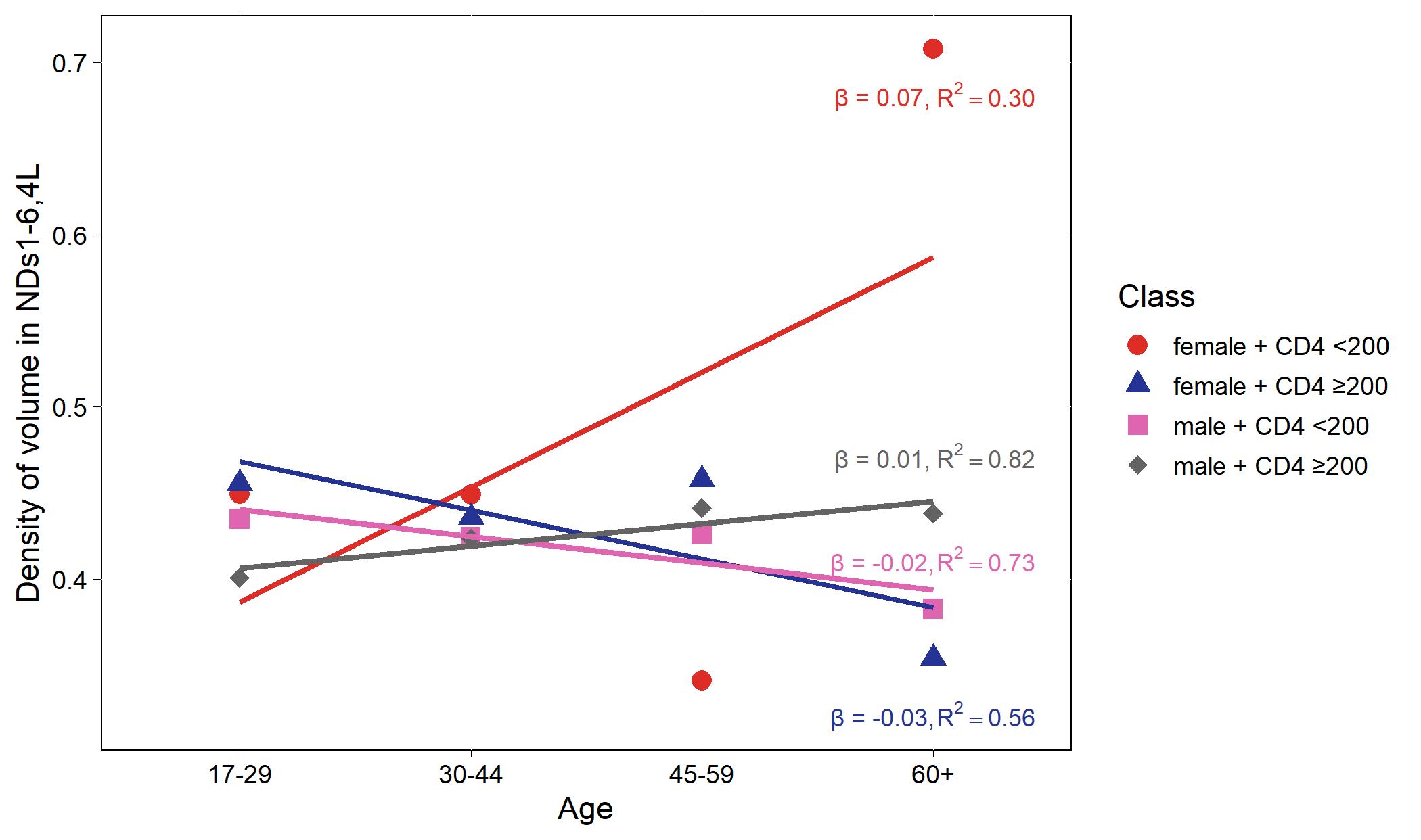 | **M**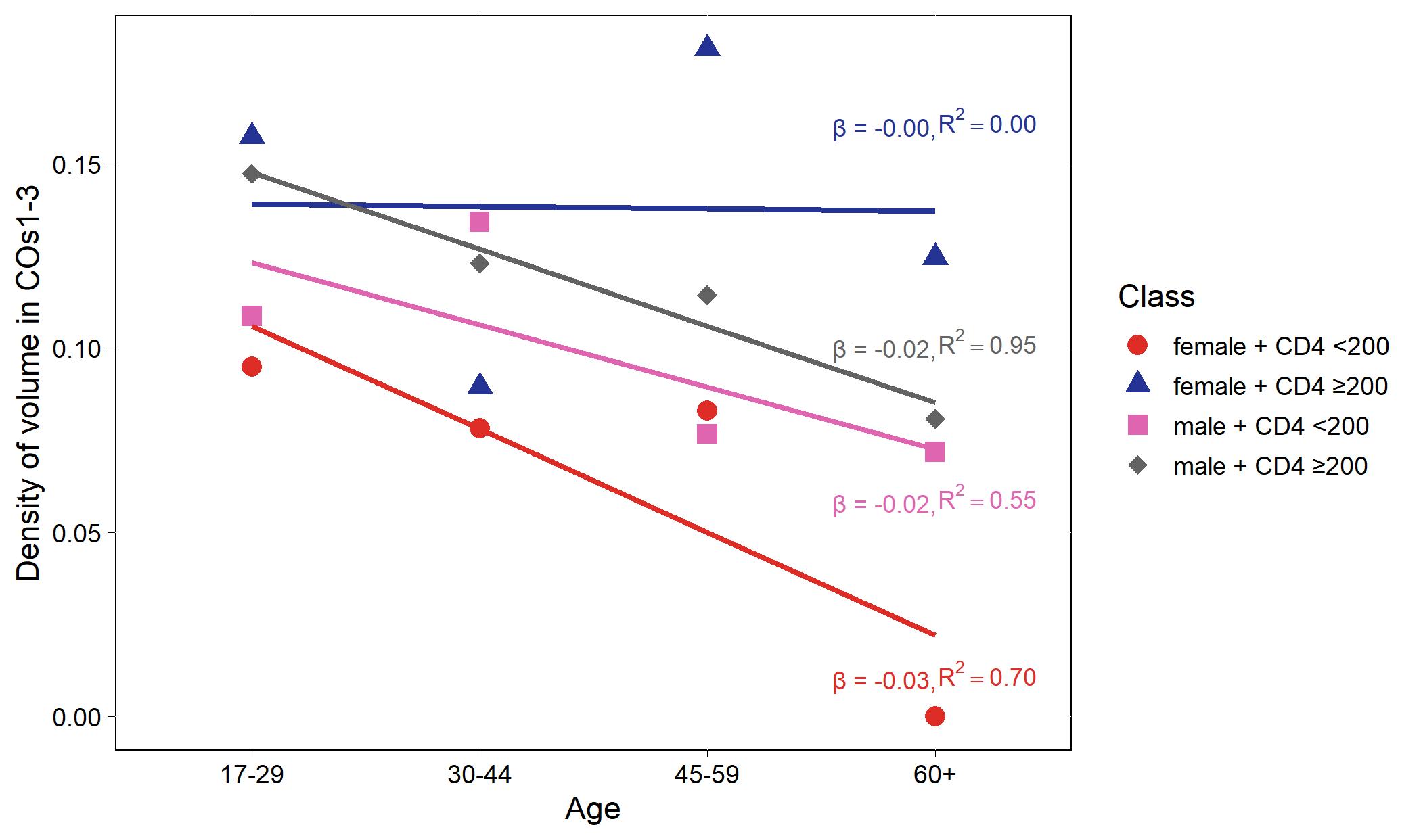 | **N** 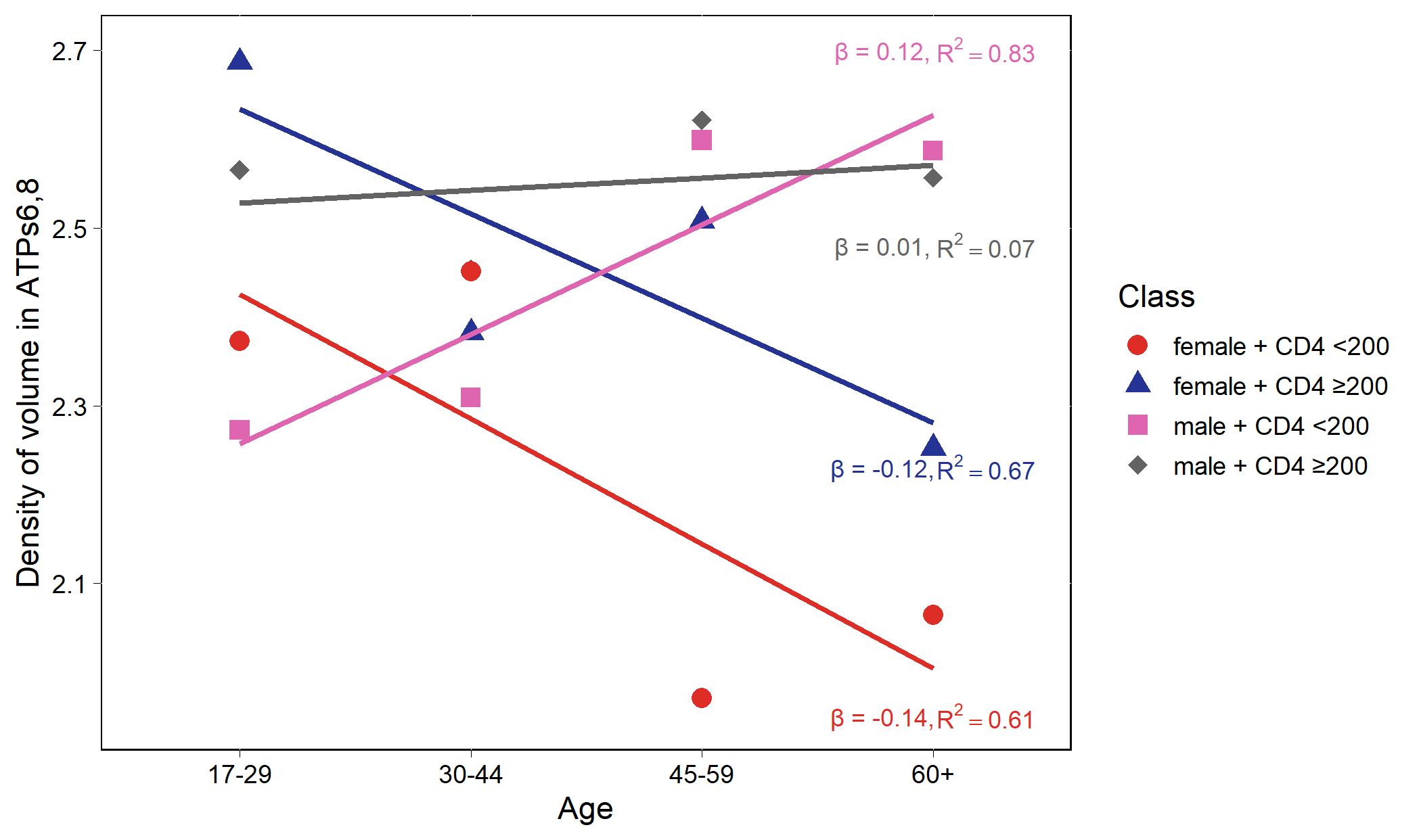 | **O**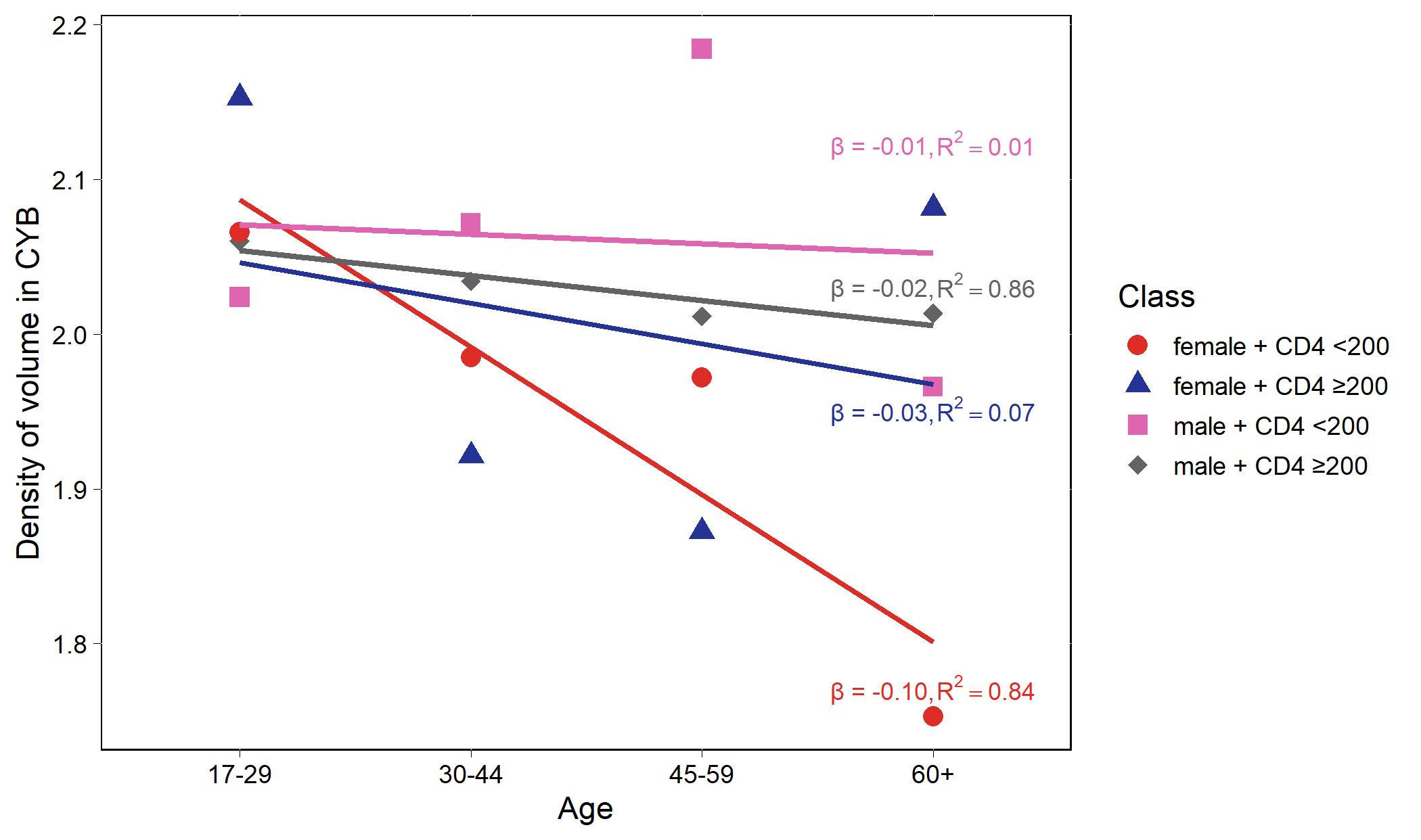 |

**Supplementary Figure 1.** Trends in mutational volume with ages in untreated PLWH.
