# Supplementary material for: Baseline Characteristics of Mitochondrial DNA and Mutations Associated With Short-Term Posttreatment CD4+T-Cell Recovery in Chinese People With HIV
Source: Front Immunol. 2021 Dec 14;12:793375. doi: 10.3389/fimmu.2021.793375 (PMC8712318; doi:10.3389/fimmu.2021.793375)
Supplement: Supplementary file 1 [file DataSheet_1.zip › SupplementaryMaterial/Supplementary Figure2.docx]

| **A**  Class 1: Male, Han ethnic, Age 17-29, CD4 <200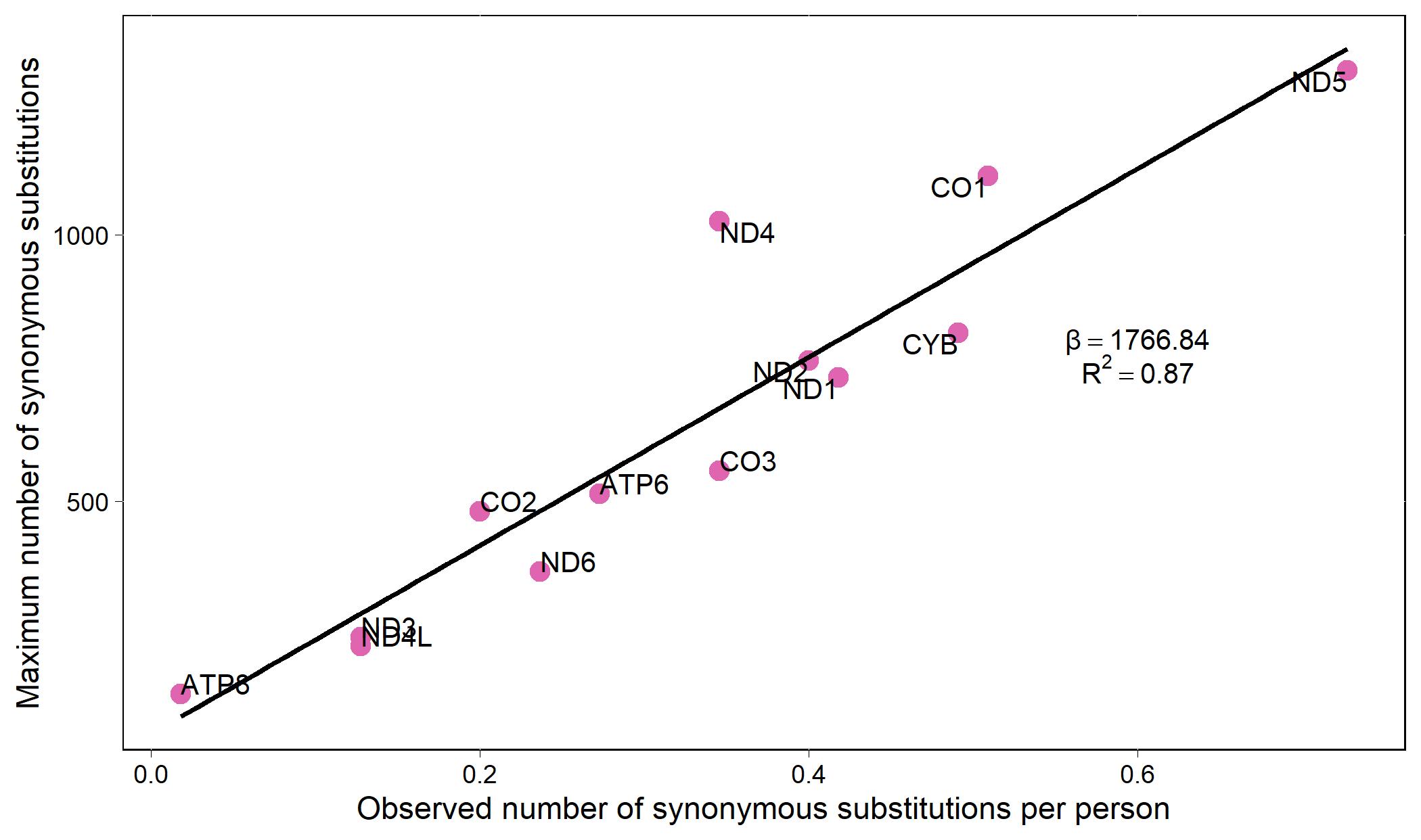 | **B**  Class 2: Male, Han ethnic, Age 30-44, CD4 <200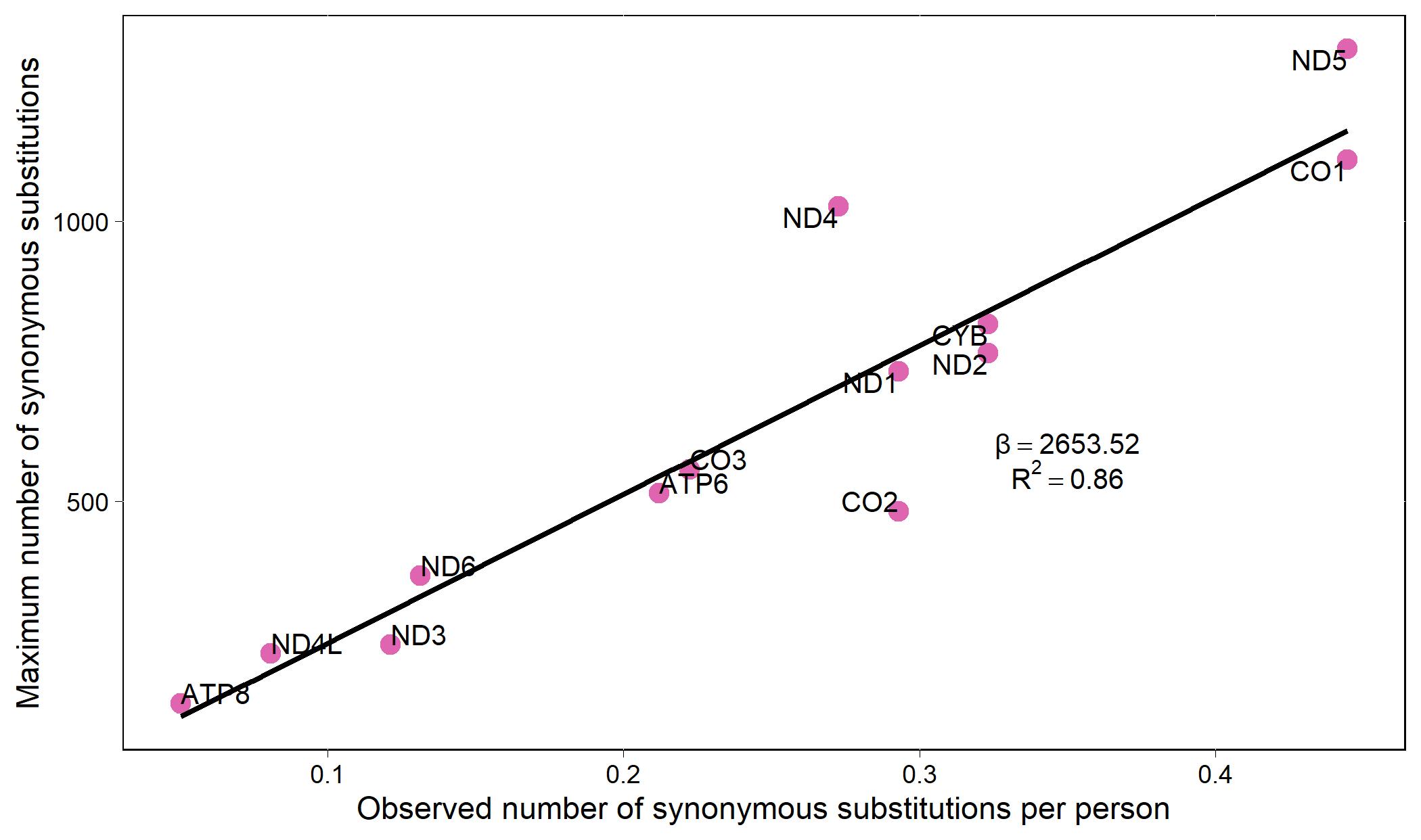 | **C**  Class 3: Male, Han ethnic, Age 45-59, CD4 <200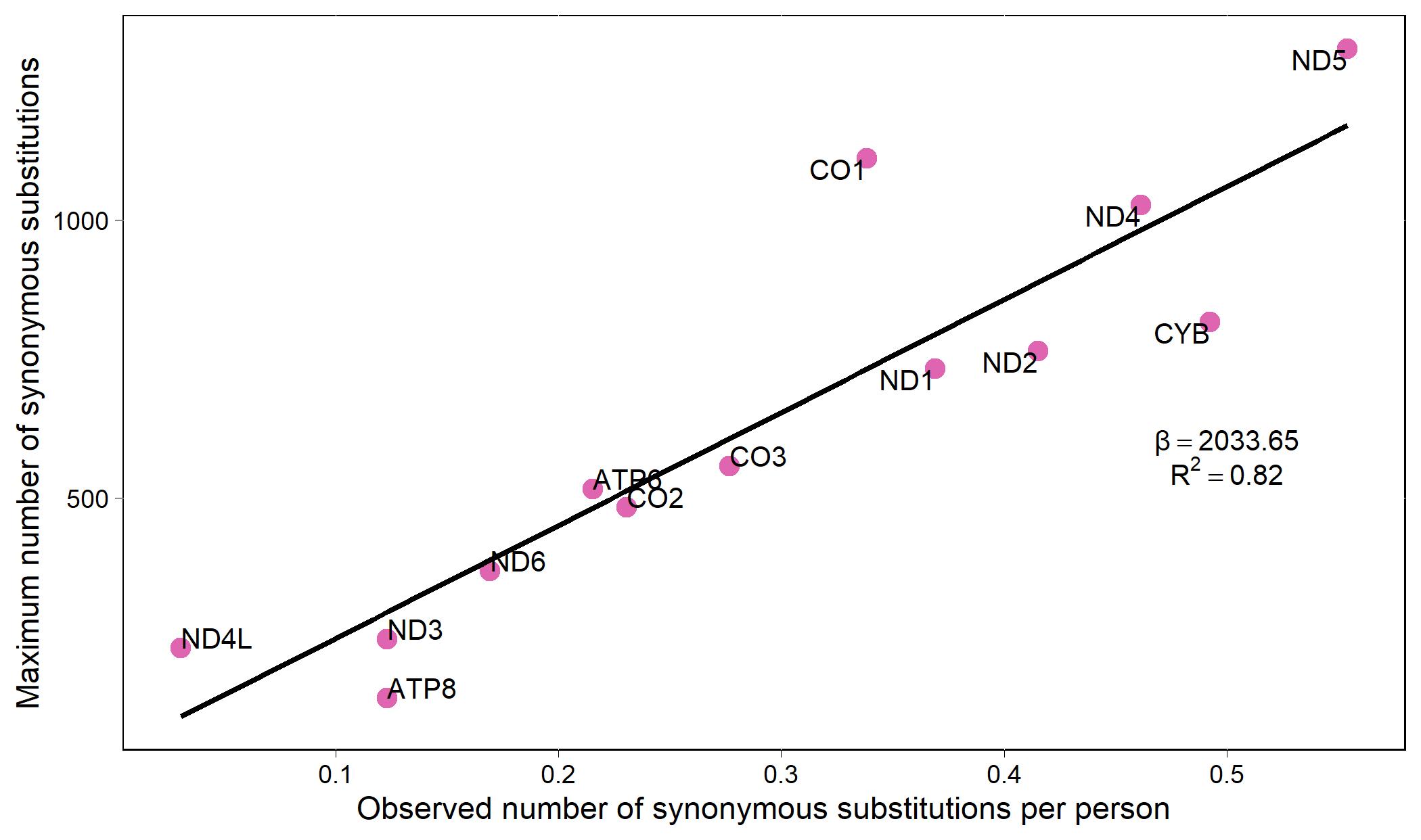 | **D**  Class 4: Male, Han ethnic, Age ≥60, CD4 <200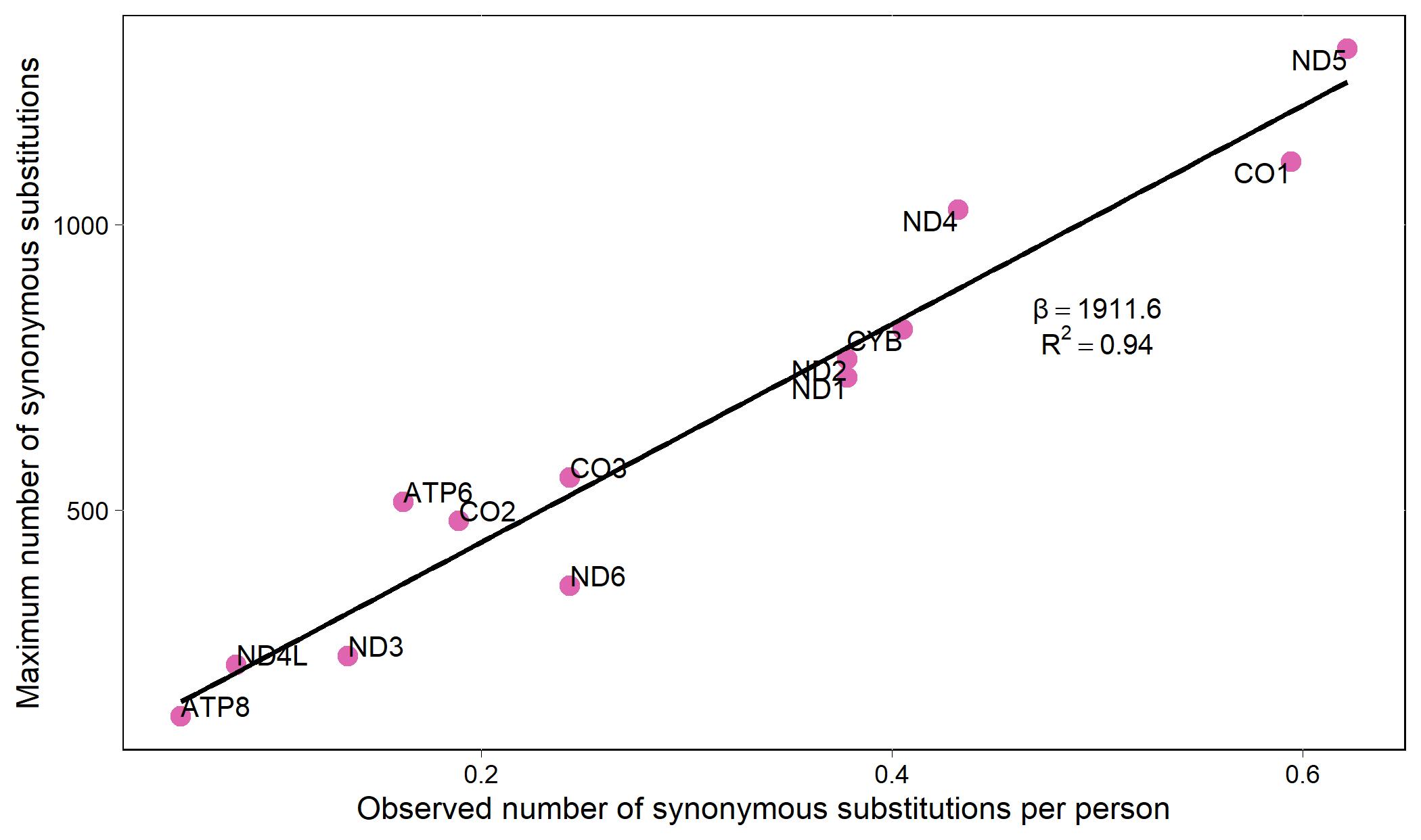 |
| --- | --- | --- | --- |
| **E**  Class 5: Male, Han ethnic, Age 17-29, CD4 ≥200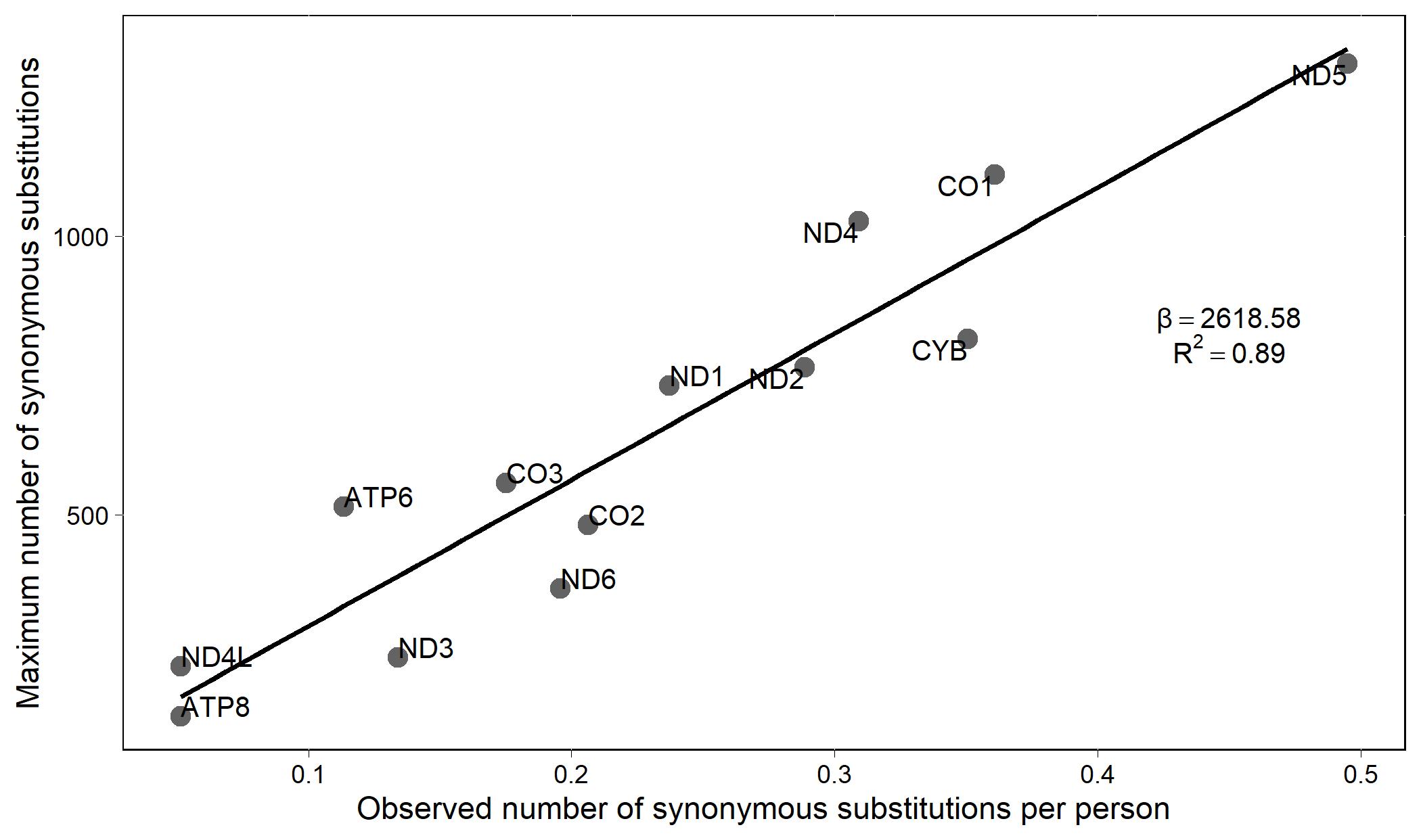 | F  Class 6: Male, Han ethnic, Age 30-44, CD4 ≥200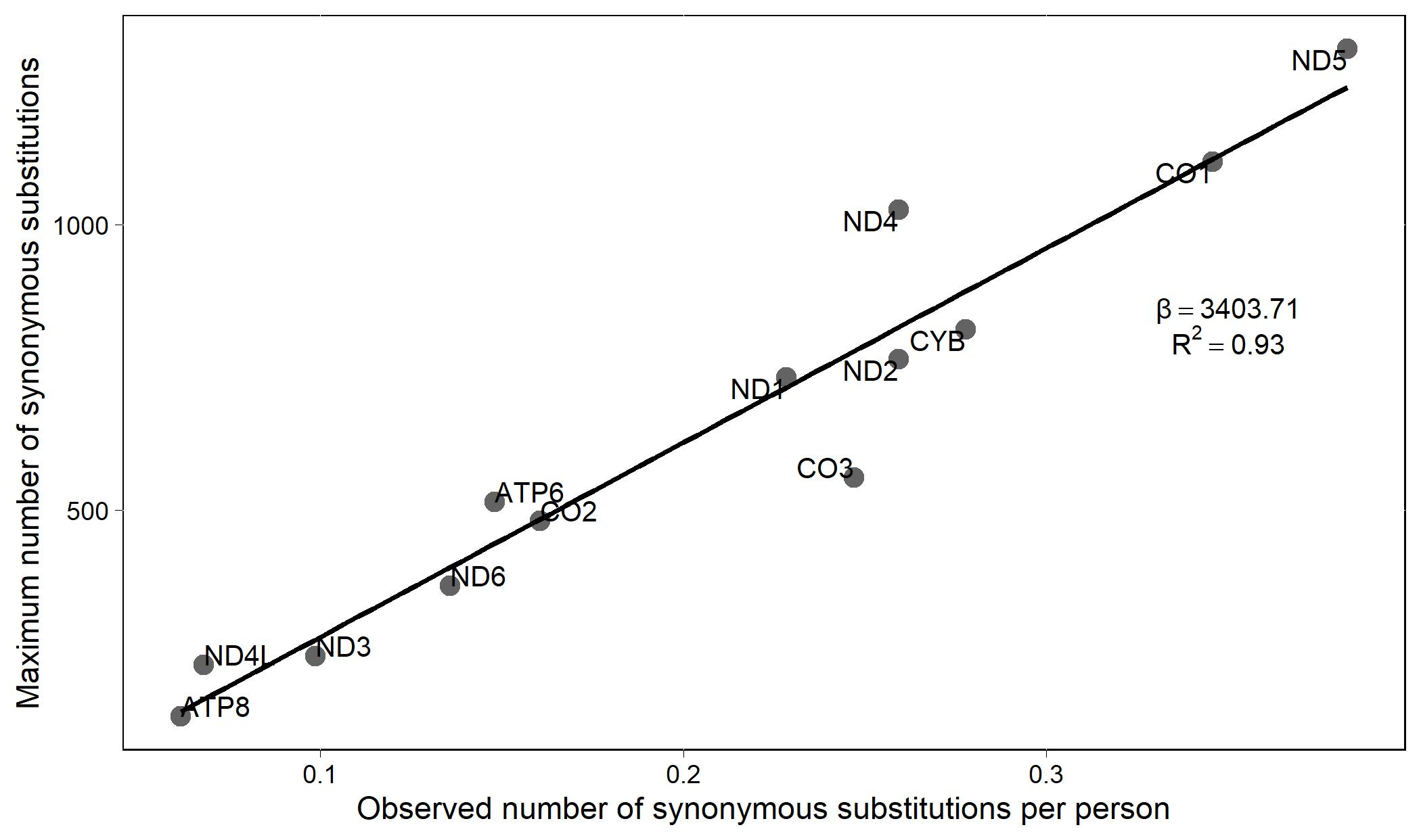 | G  Class 7: Male, Han ethnic, Age 45-59, CD4 ≥200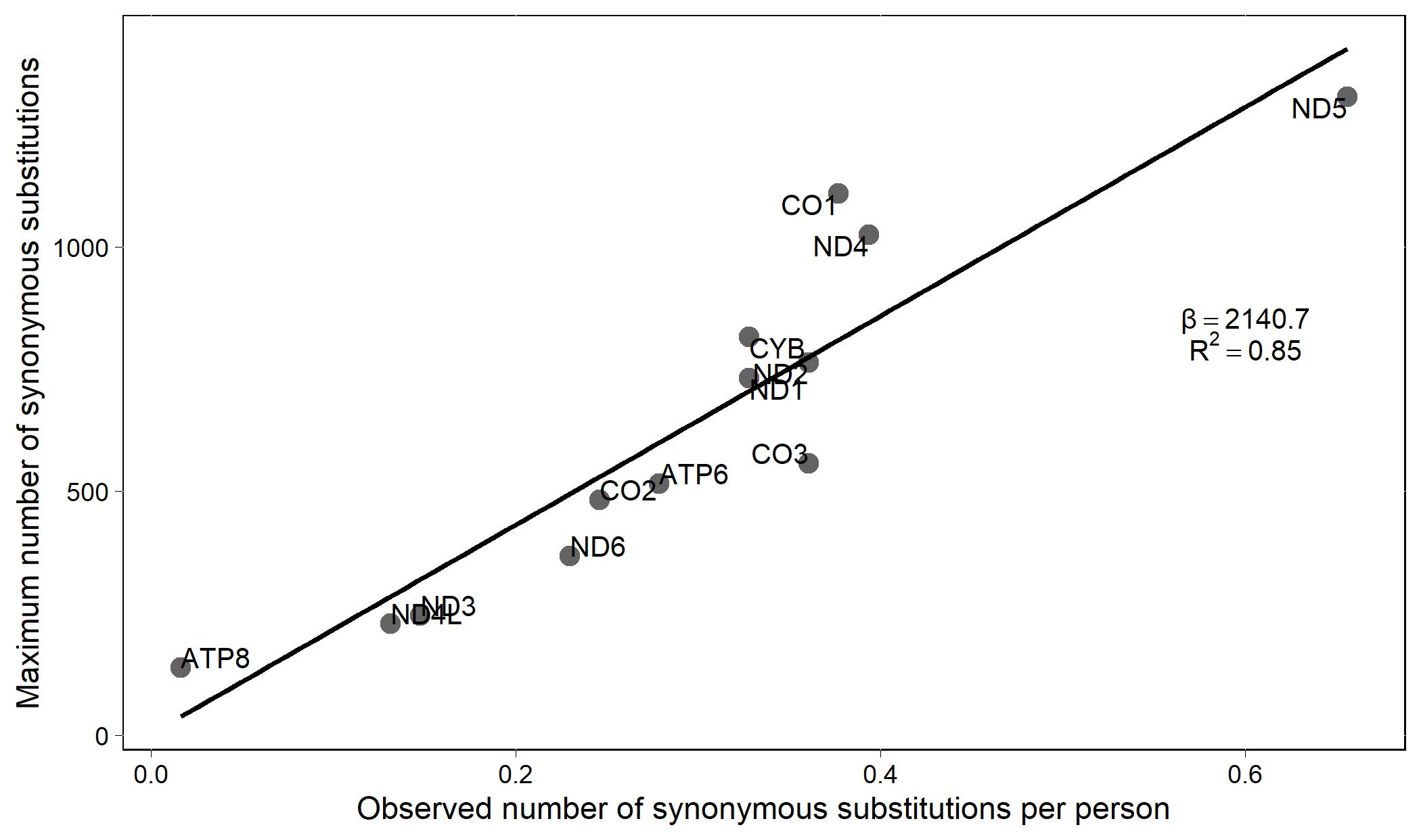 | H  Class 8: Male, Han ethnic, Age ≥60, CD4 ≥200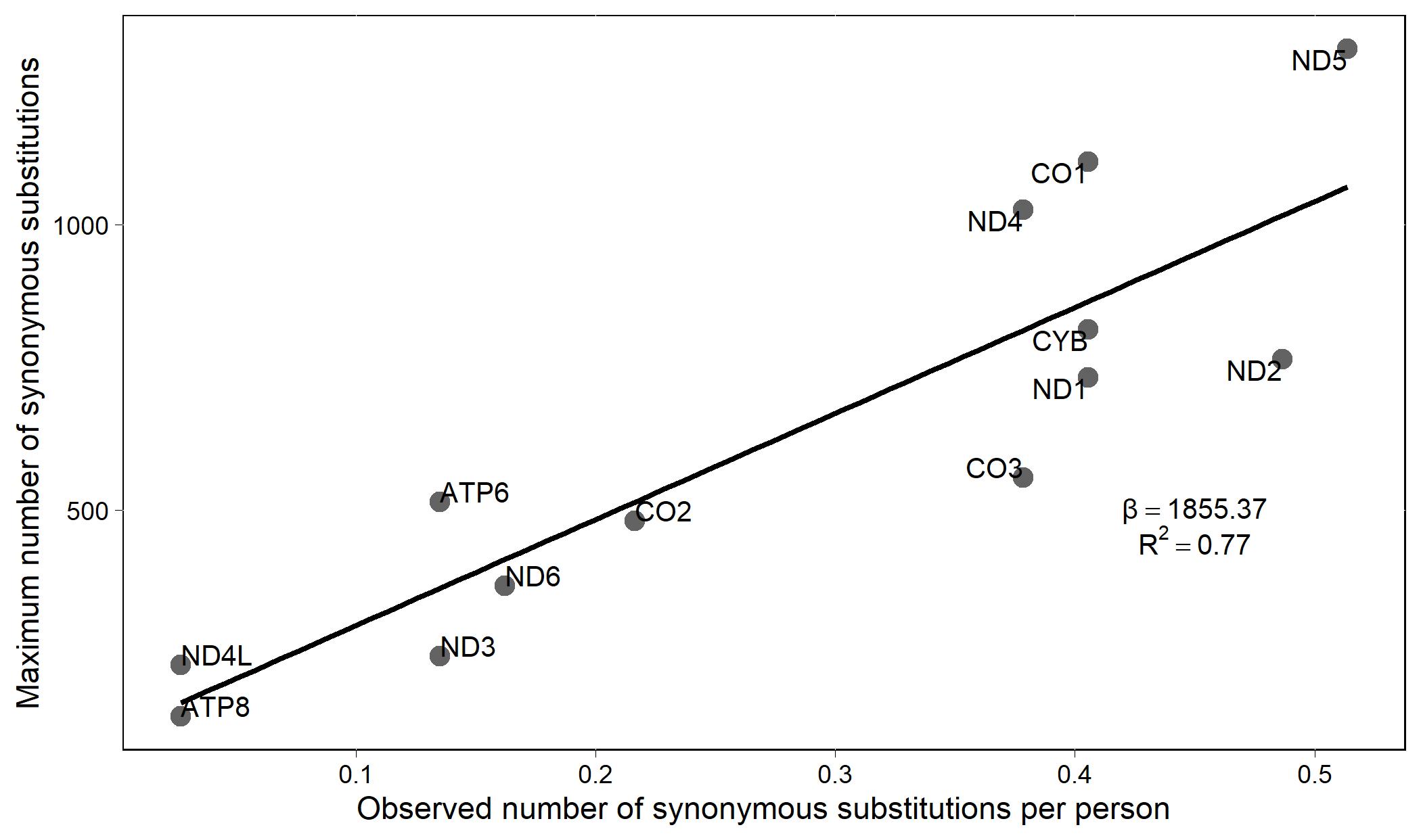 |
| I  Class 9: Female, Han ethnic, Age 17-29, CD4 <200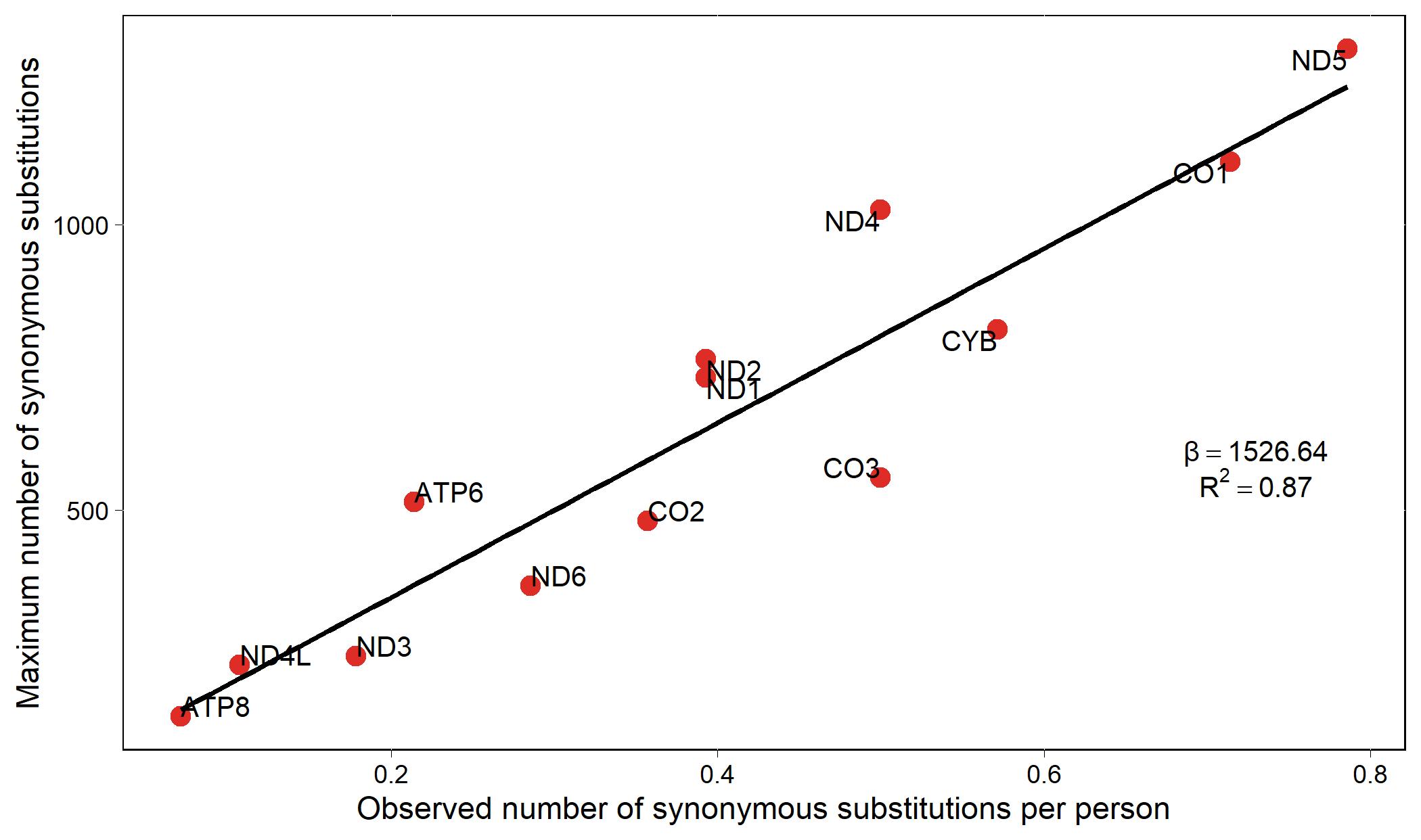 | J  Class 10: Female, Han ethnic, Age 30-44, CD4 <200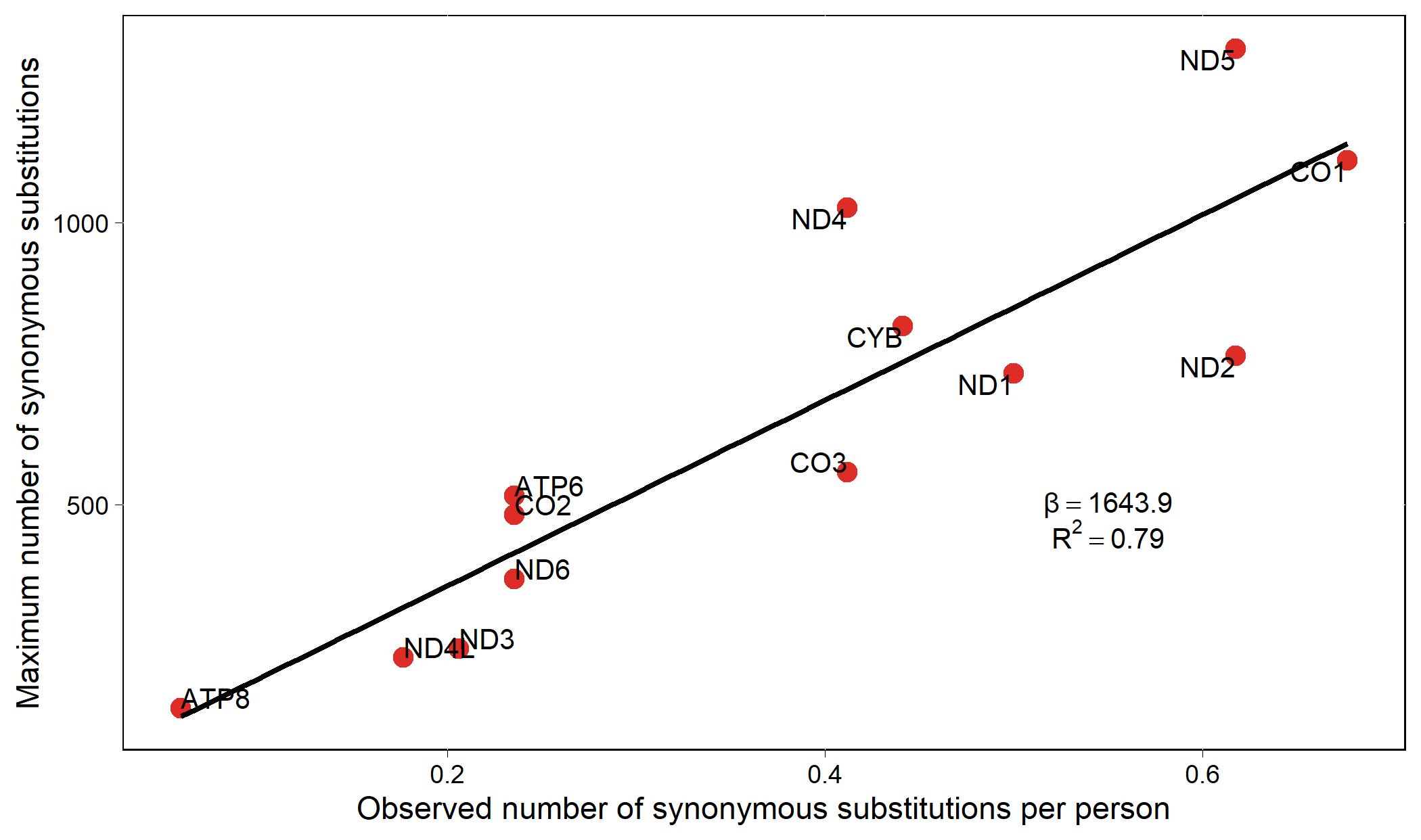 | K  Class 11: Female, Han ethnic, Age 45-59, CD4 <200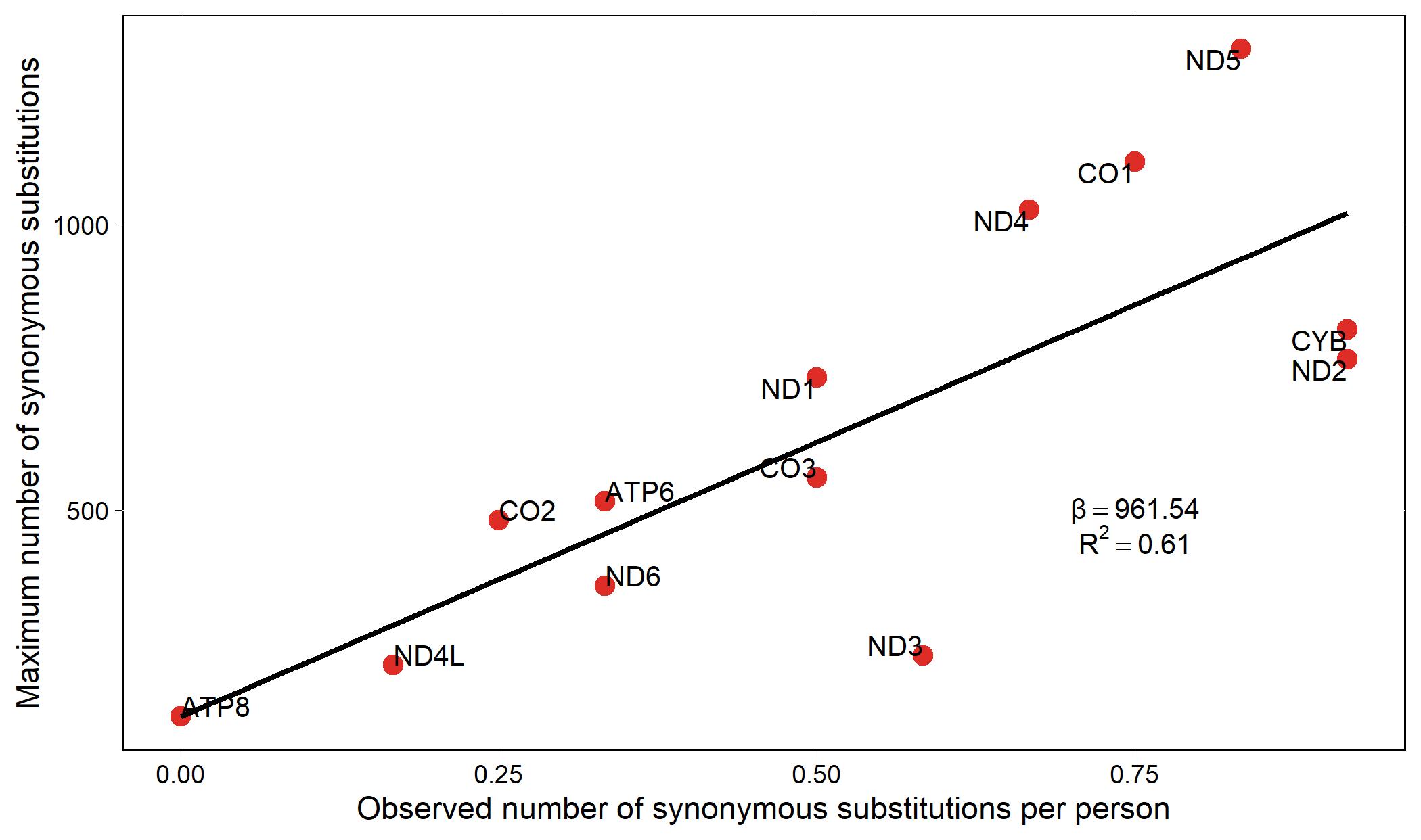 | L  Class 12: Female, Han ethnic, Age ≥60, CD4 <200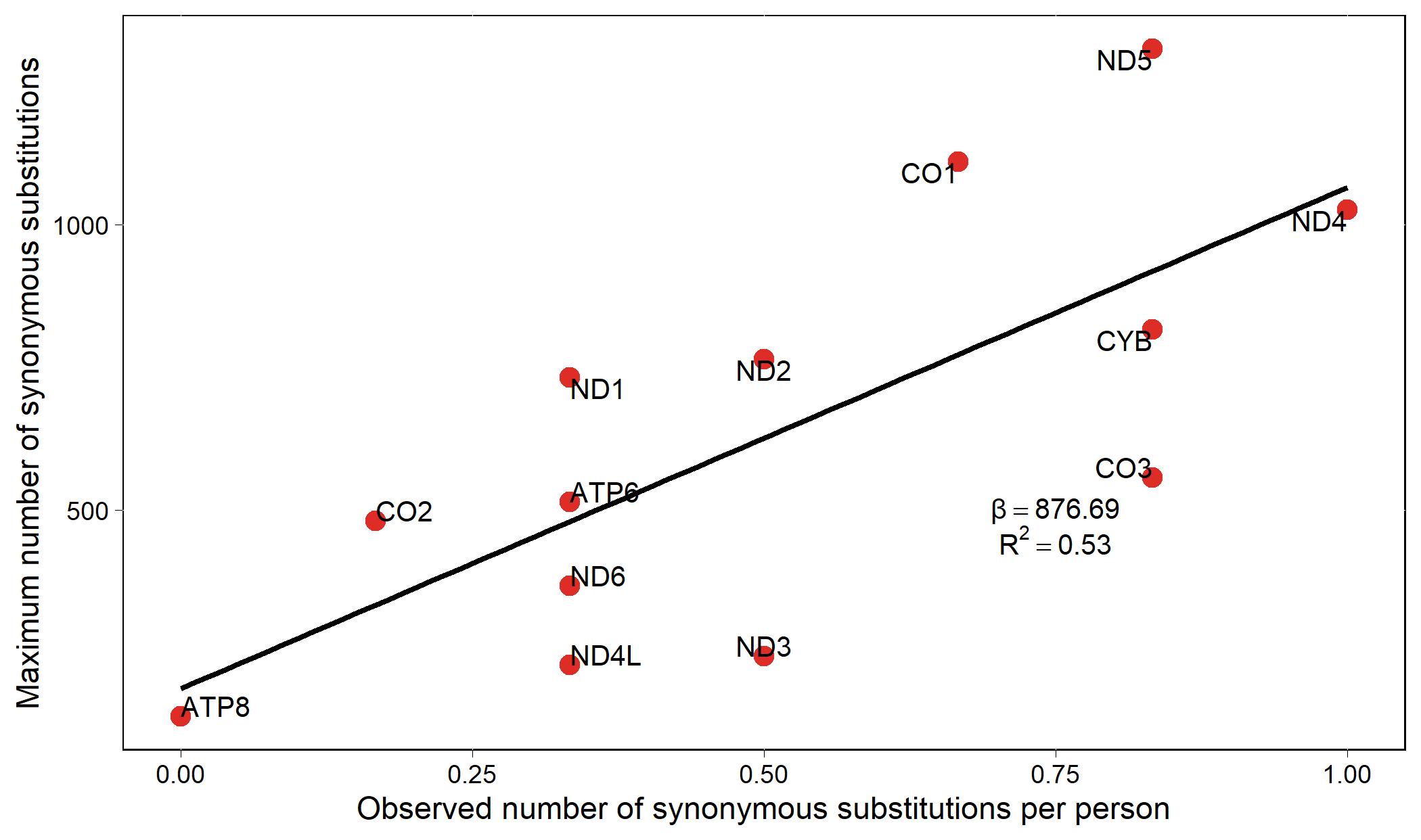 |
| M  Class 13: Female, Han ethnic, Age 17-29, CD4 ≥200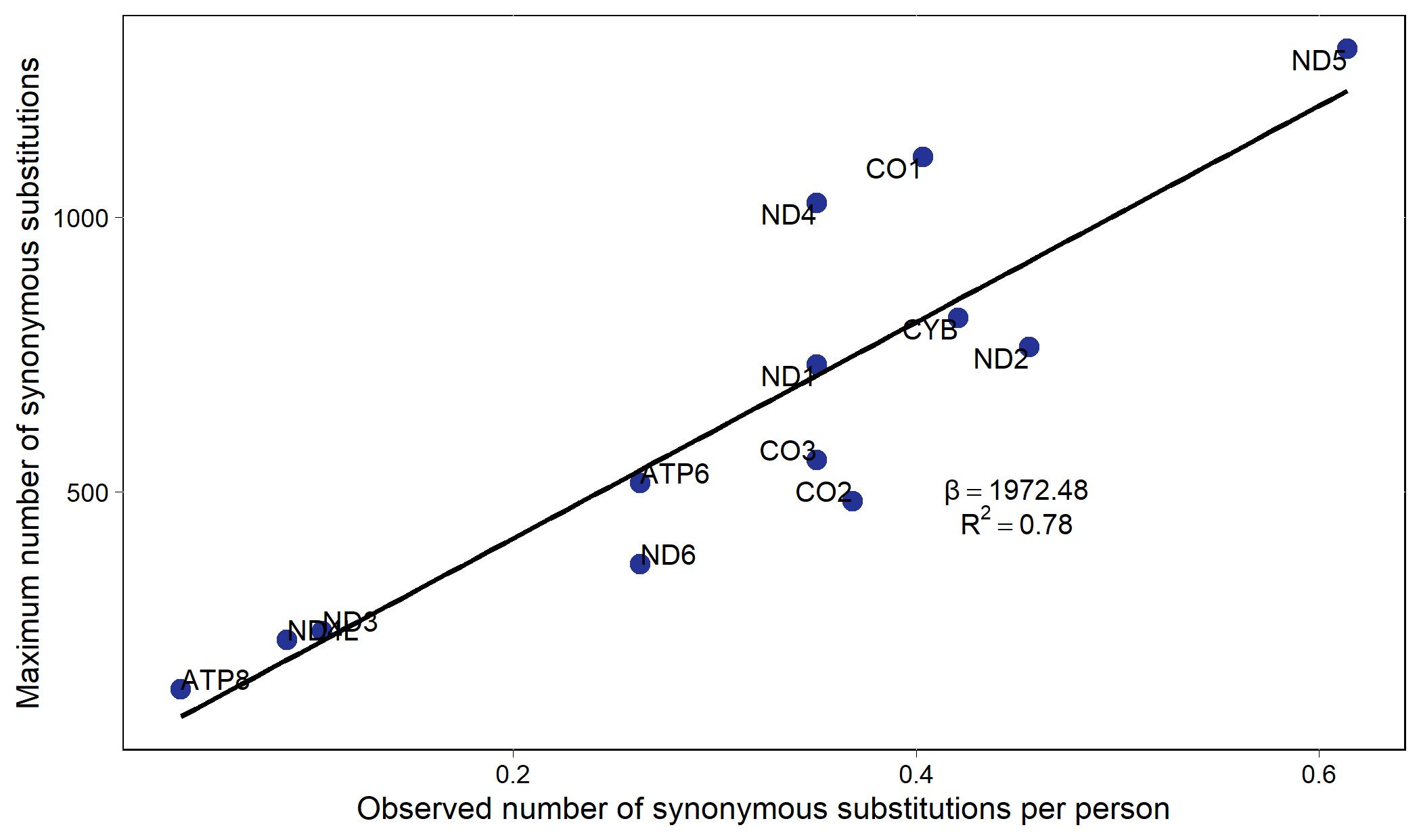 | N  Class 14: Female, Han ethnic, Age 30-44, CD4 ≥200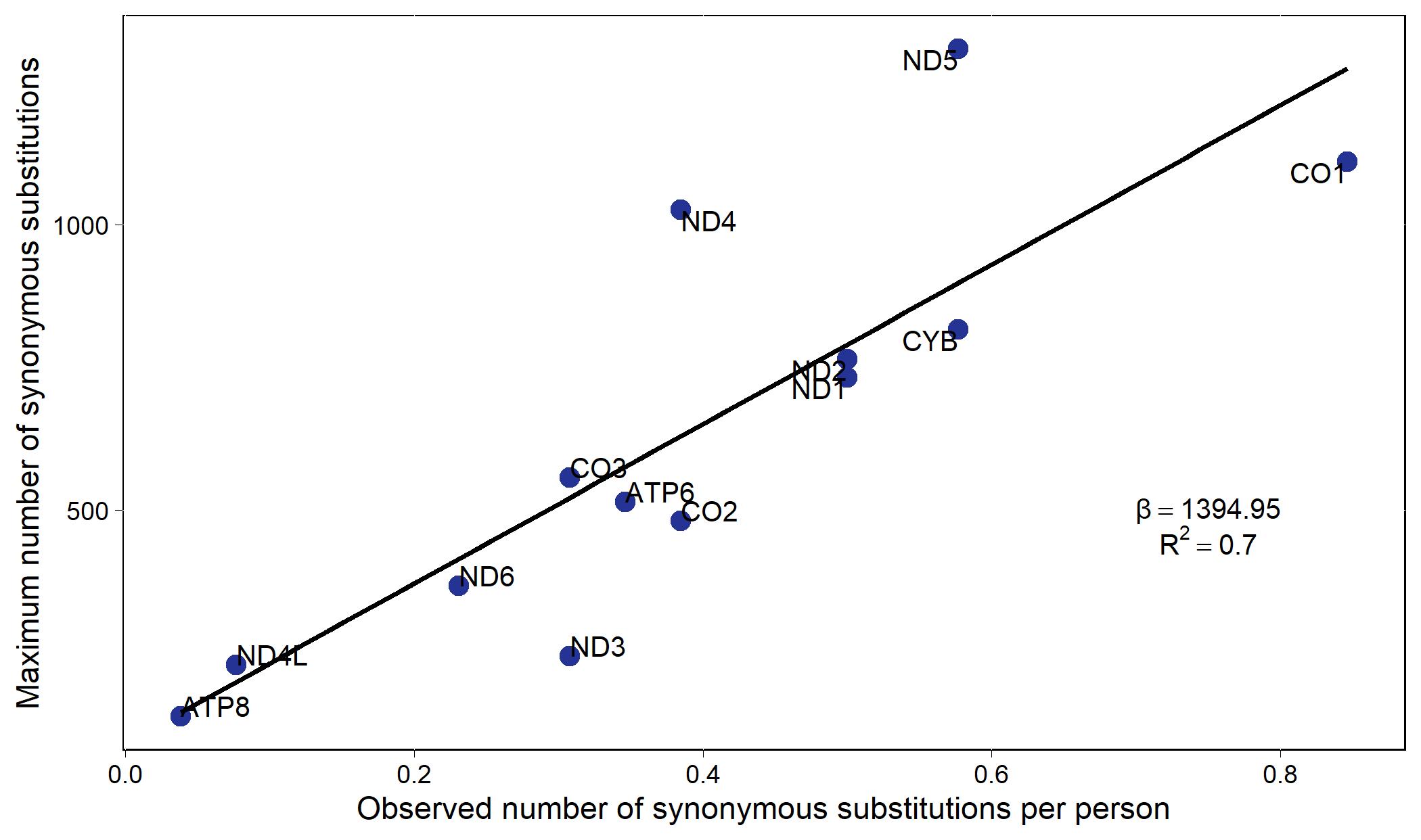 | O  Class 15: Female, Han ethnic, Age 45-59, CD4 ≥200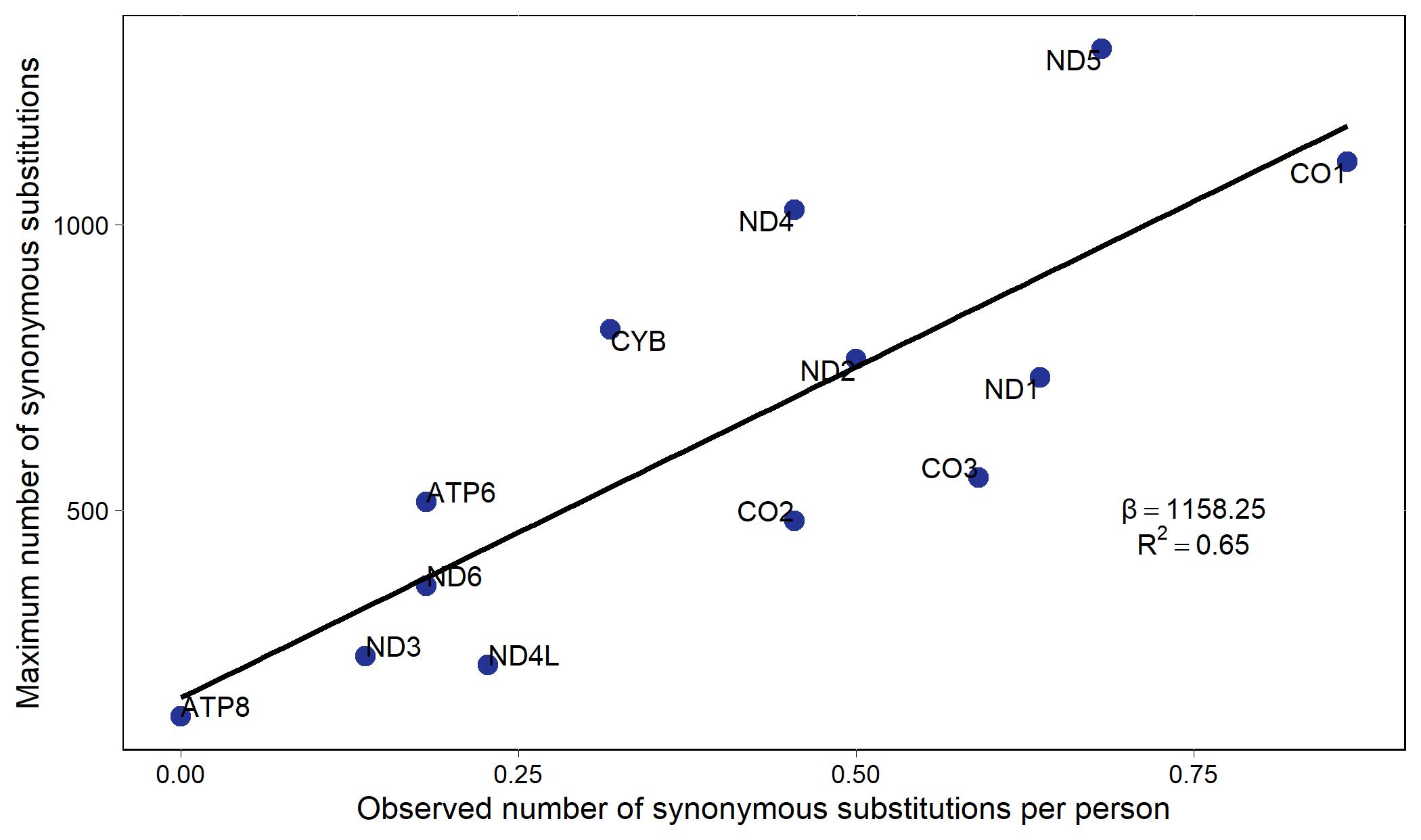 | P  Class 16: Female, Han ethnic, Age ≥60, CD4 ≥200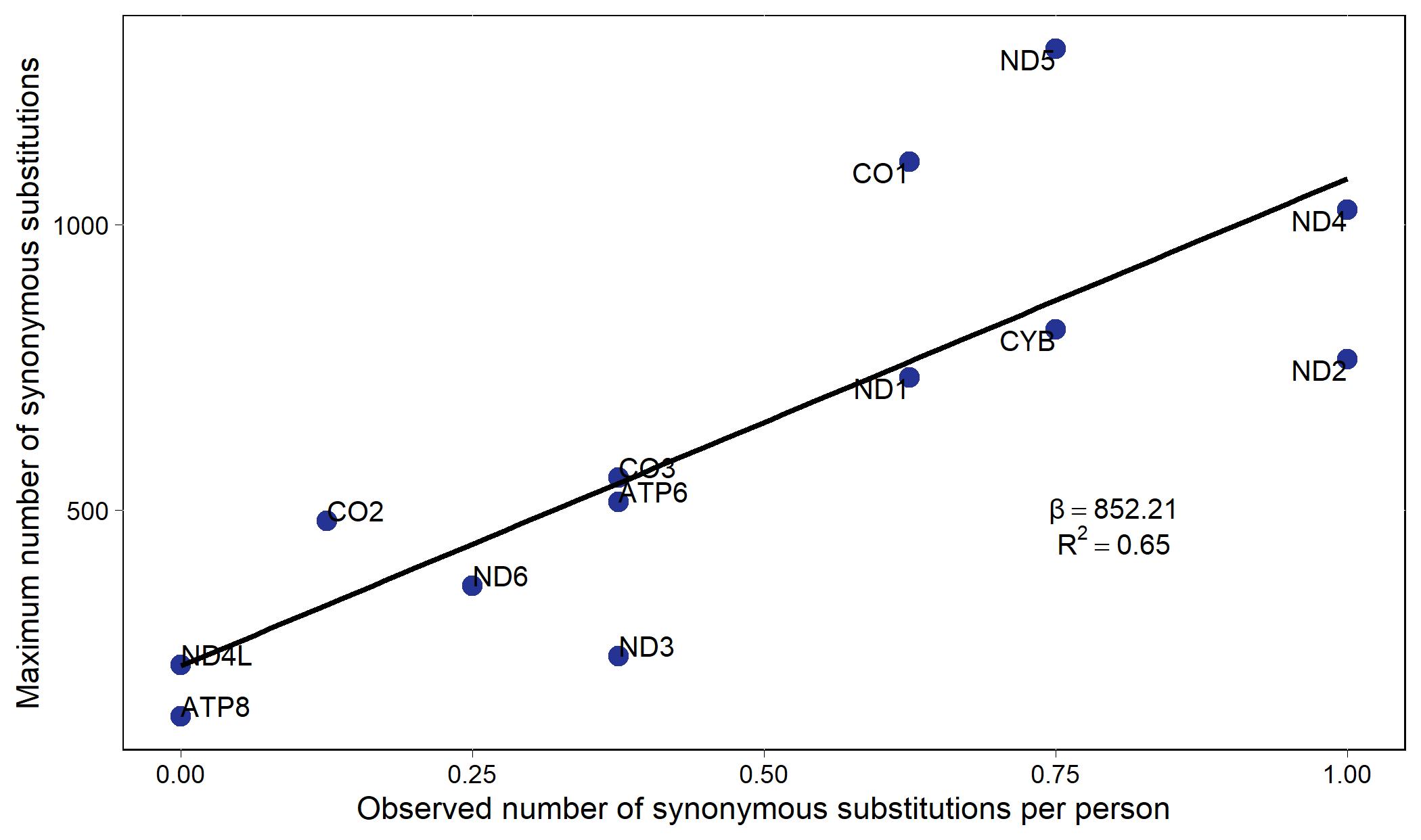 |

**Supplementary Figure 2.** Correlation between observed and maximum synonymous substitutions across 16 subpopulations of untreated PLWH.
