# Supplementary material for: Baseline Characteristics of Mitochondrial DNA and Mutations Associated With Short-Term Posttreatment CD4+T-Cell Recovery in Chinese People With HIV
Source: Front Immunol. 2021 Dec 14;12:793375. doi: 10.3389/fimmu.2021.793375 (PMC8712318; doi:10.3389/fimmu.2021.793375)
Supplement: Supplementary file 1 [file DataSheet_1.zip › SupplementaryMaterial/Supplementary Figure3.docx]

| A  Class 1: Male, Han ethnic, Age 17-29, CD4 <200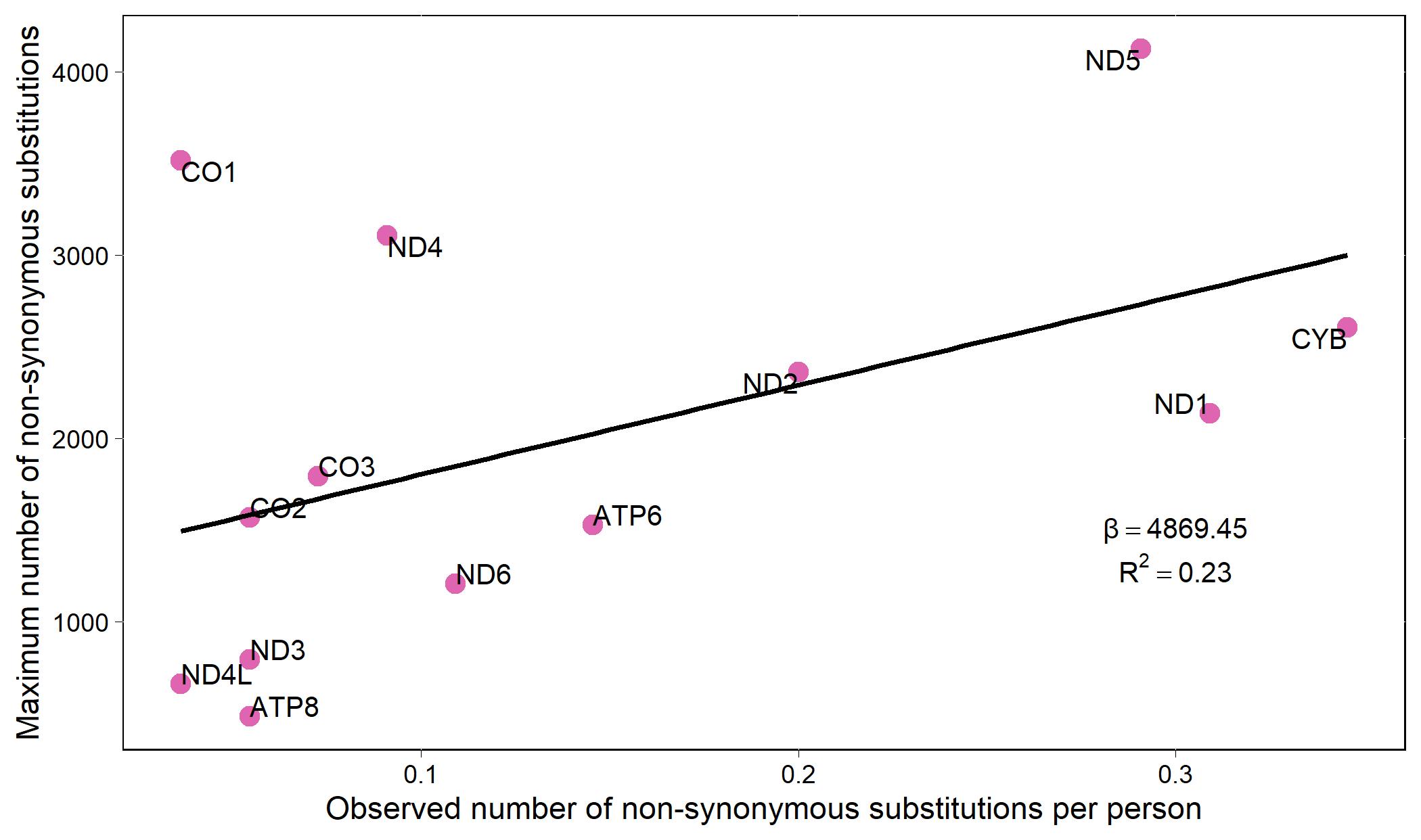 | b  Class 2: Male, Han ethnic, Age 30-44, CD4 <200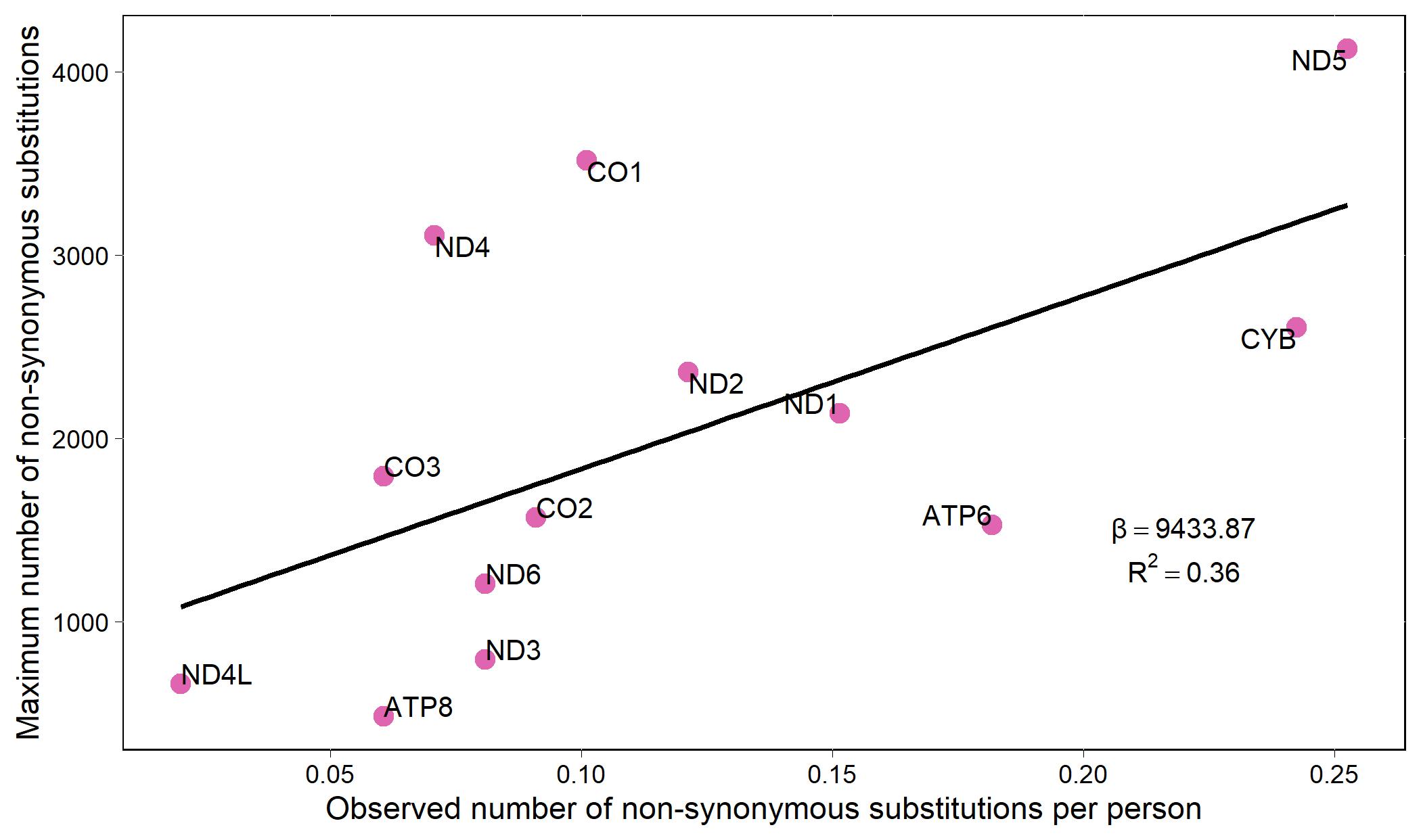 | c  Class 3: Male, Han ethnic, Age 45-59, CD4 <200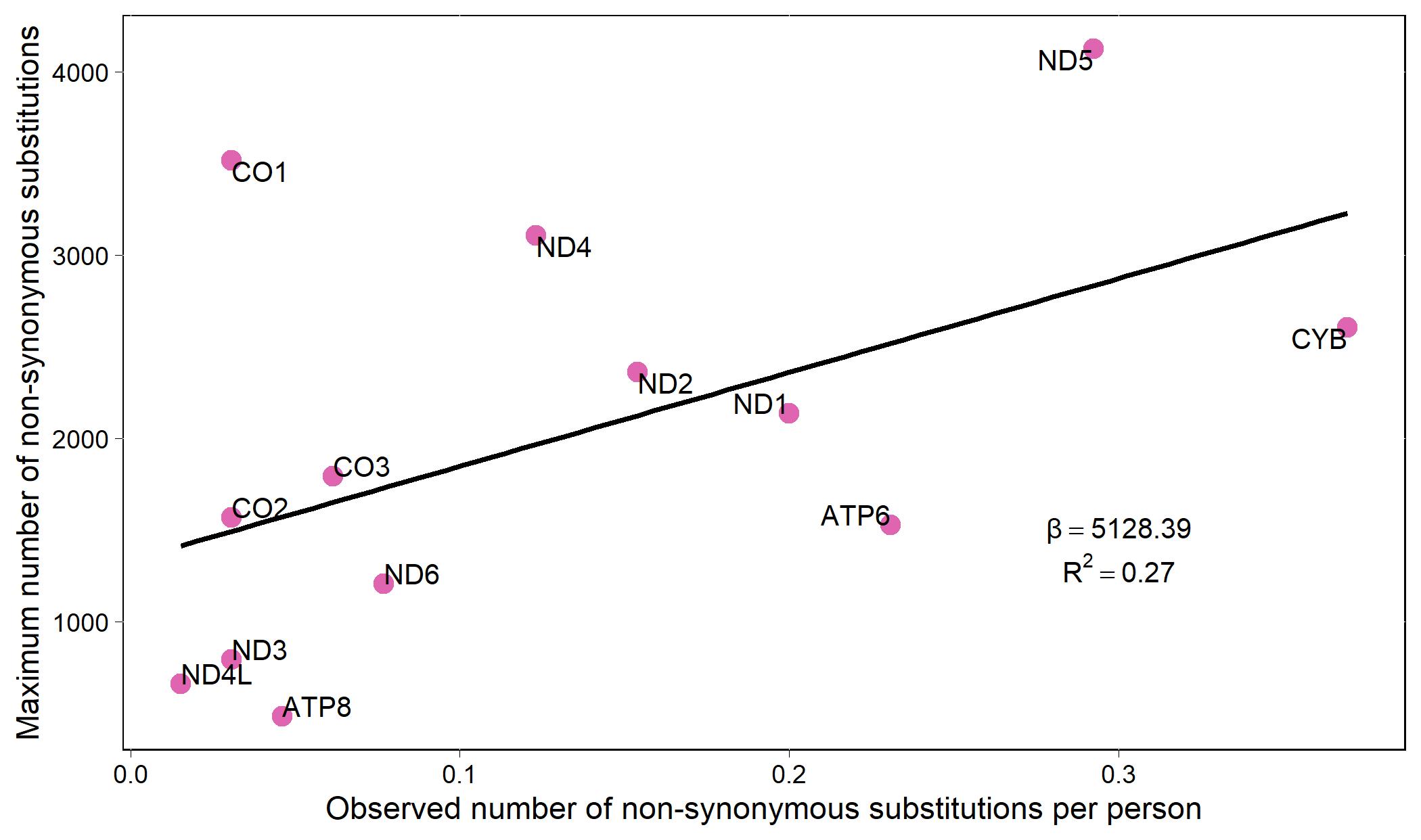 | d  Class 4: Male, Han ethnic, Age ≥60, CD4 <200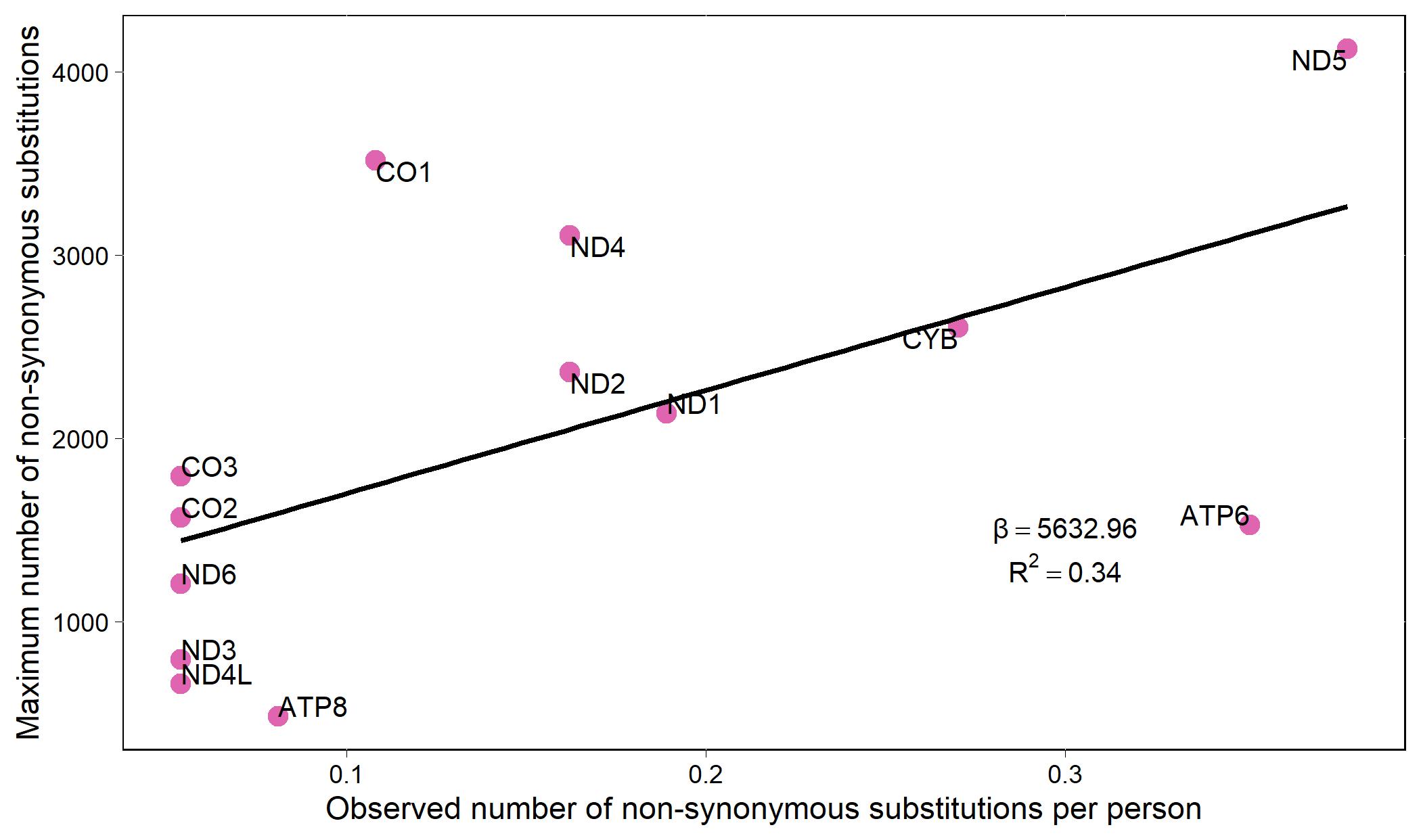 |
| --- | --- | --- | --- |
| e  Class 5: Male, Han ethnic, Age 17-29, CD4 ≥200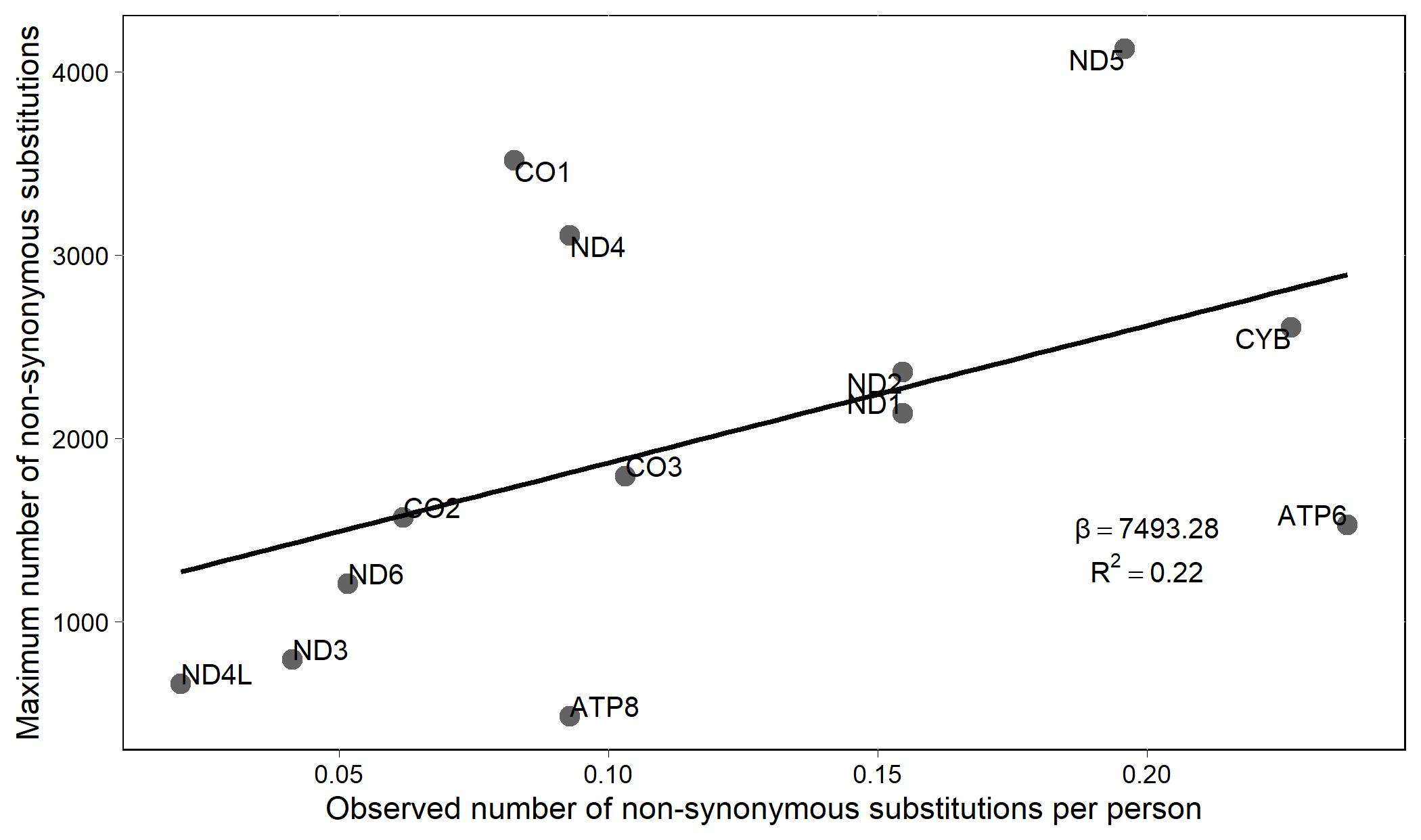 | f  Class 6: Male, Han ethnic, Age 30-44, CD4 ≥200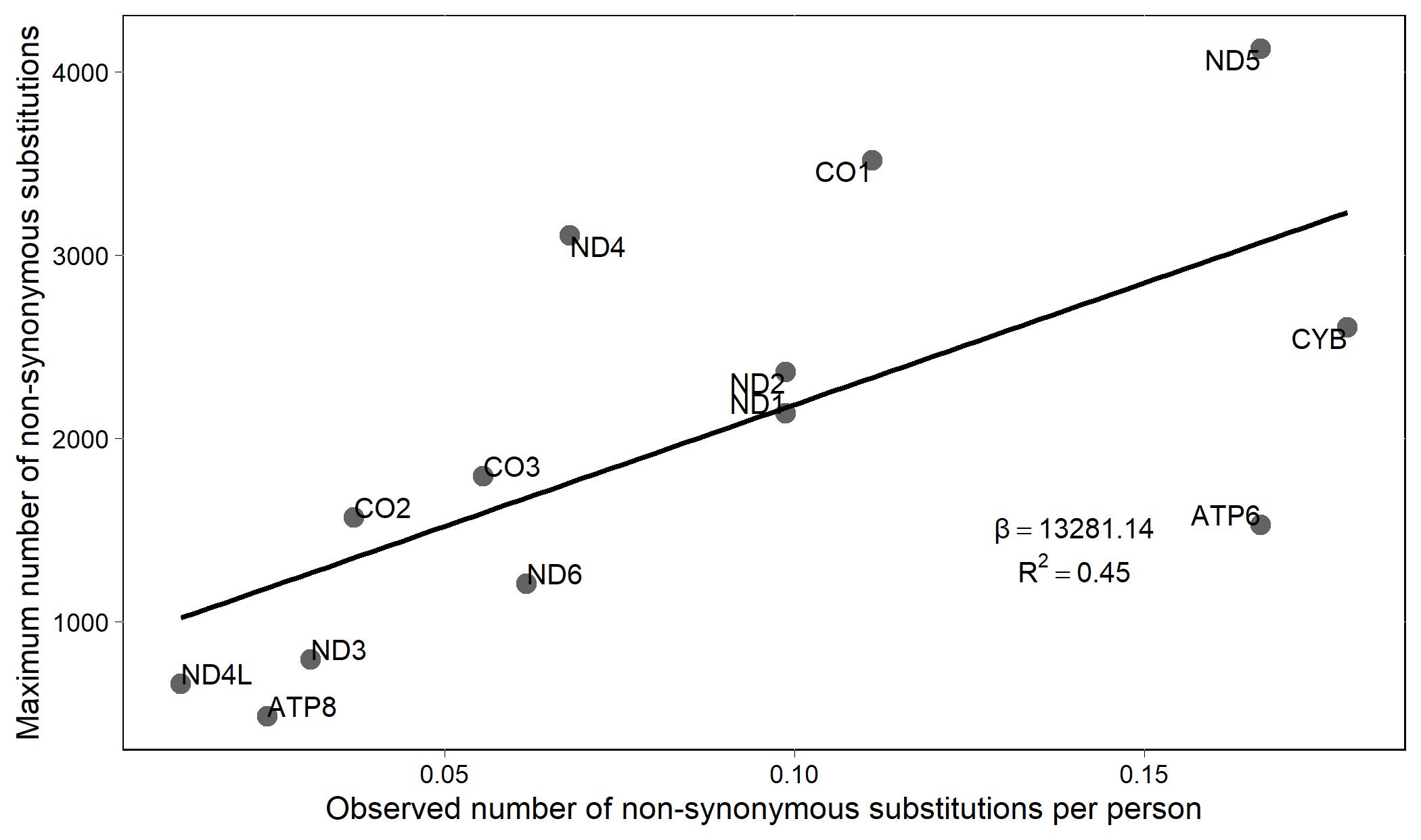 | g  Class 7: Male, Han ethnic, Age 45-59, CD4 ≥200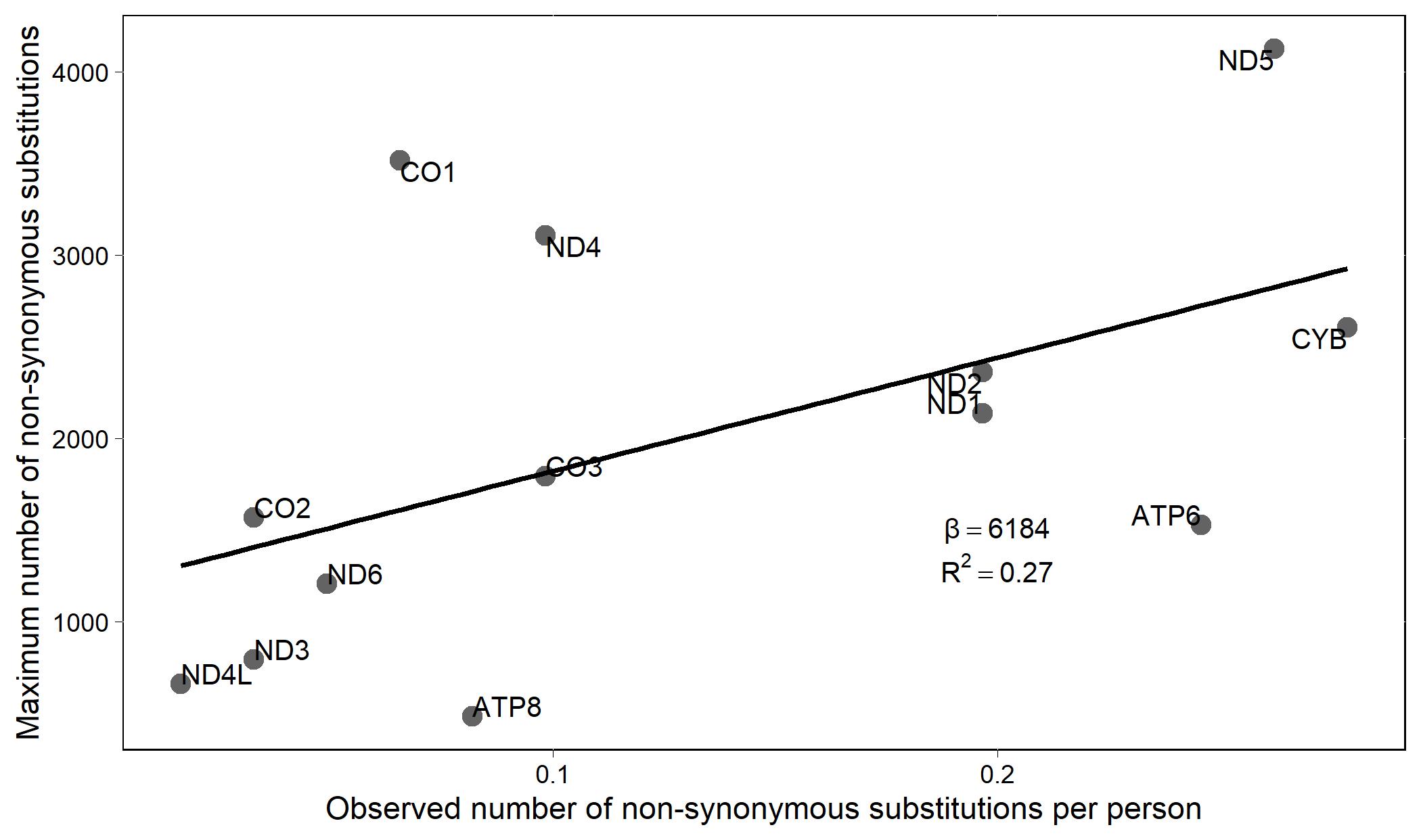 | h  Class 8: Male, Han ethnic, Age ≥60, CD4 ≥200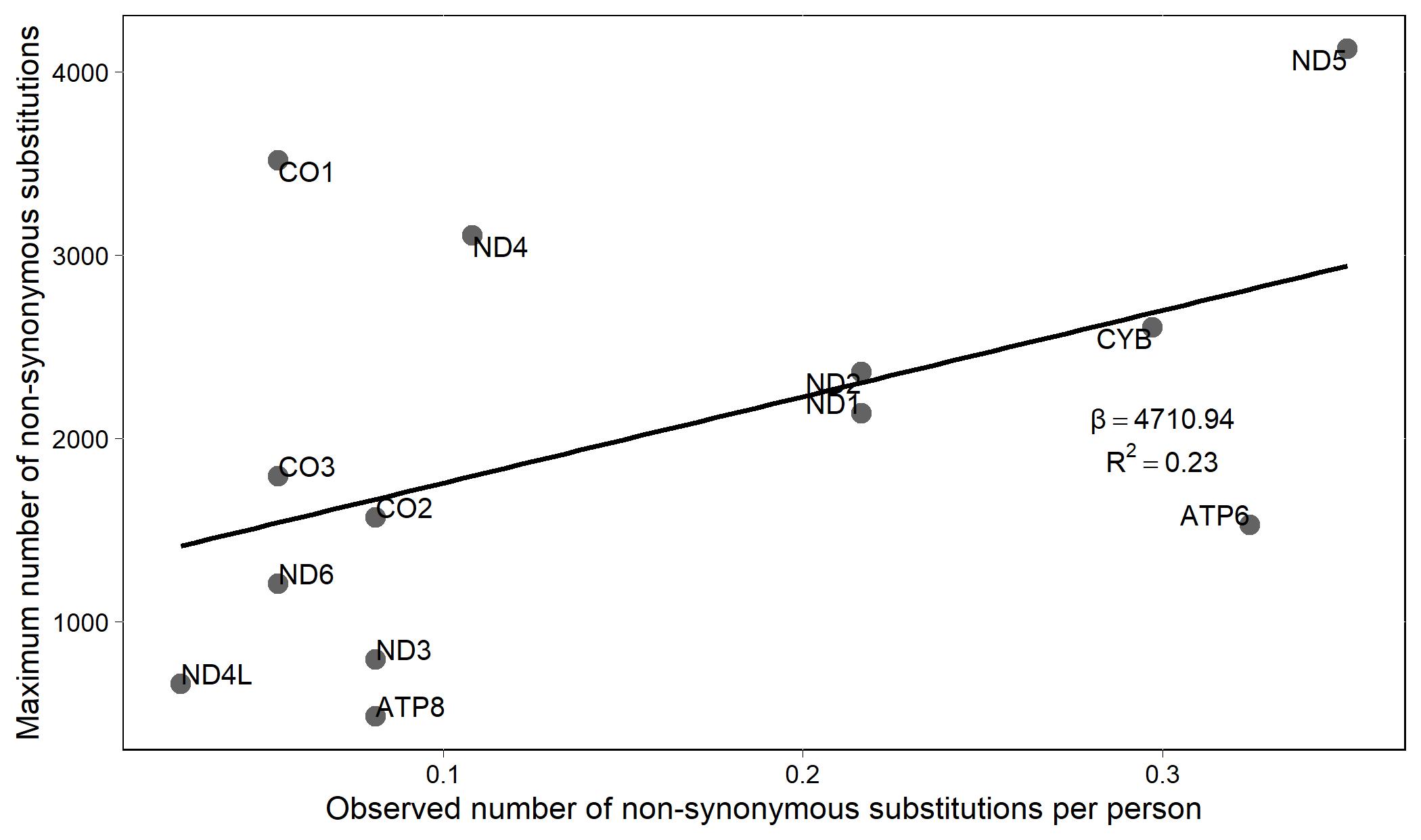 |
| i  Class 9: Female, Han ethnic, Age 17-29, CD4 <200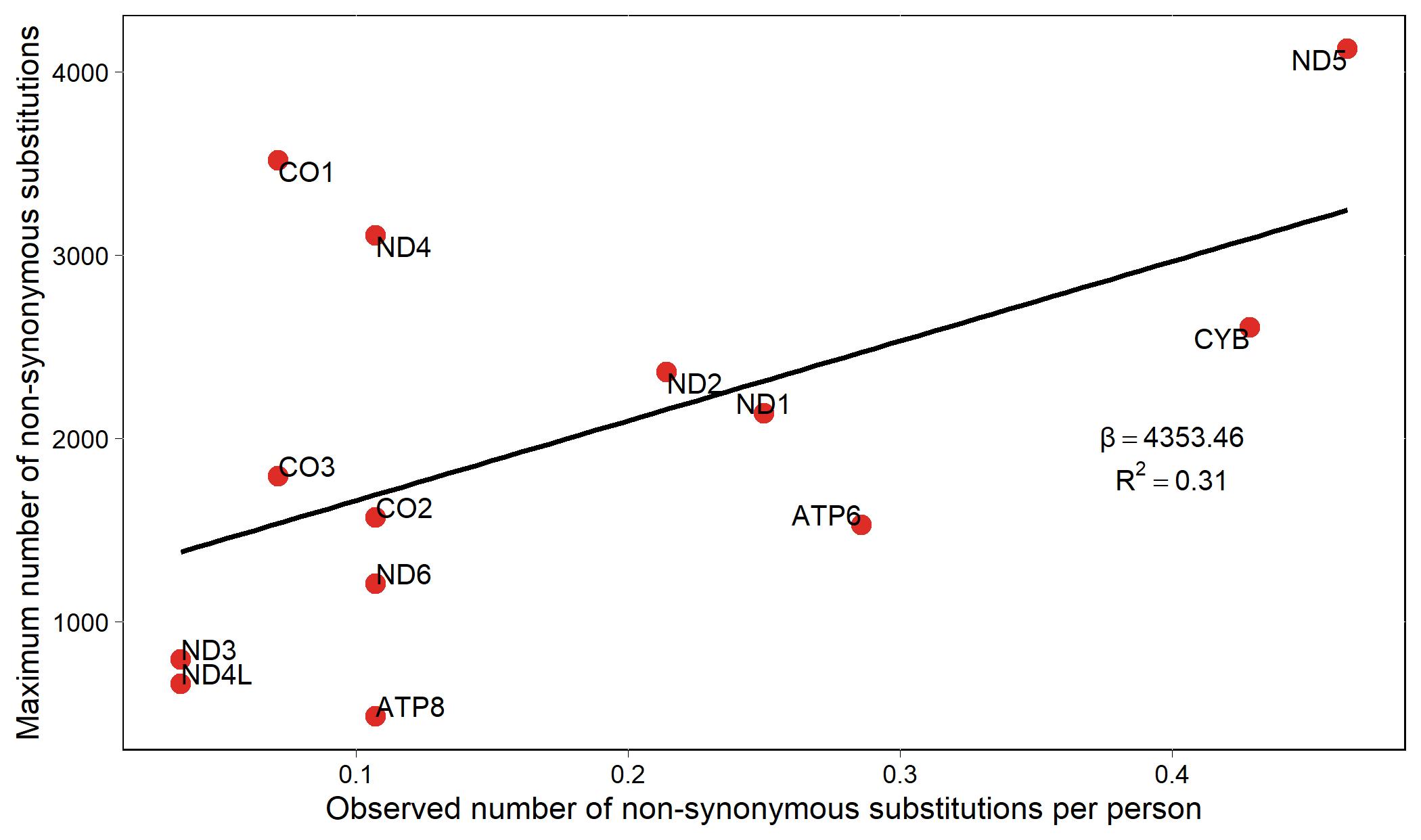 | j  Class 10: Female, Han ethnic, Age 30-44, CD4 <200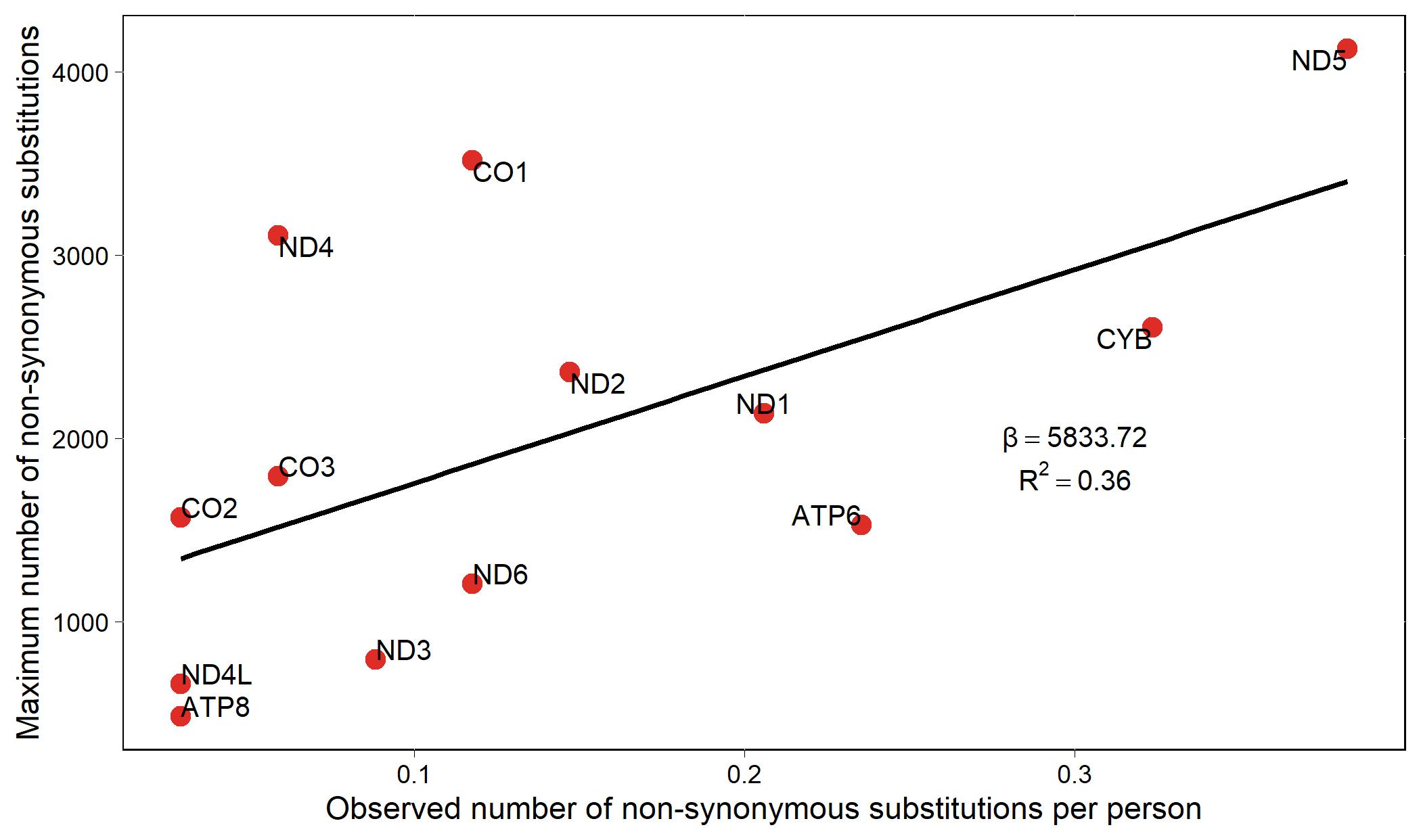 | k  Class 11: Female, Han ethnic, Age 45-59, CD4 <200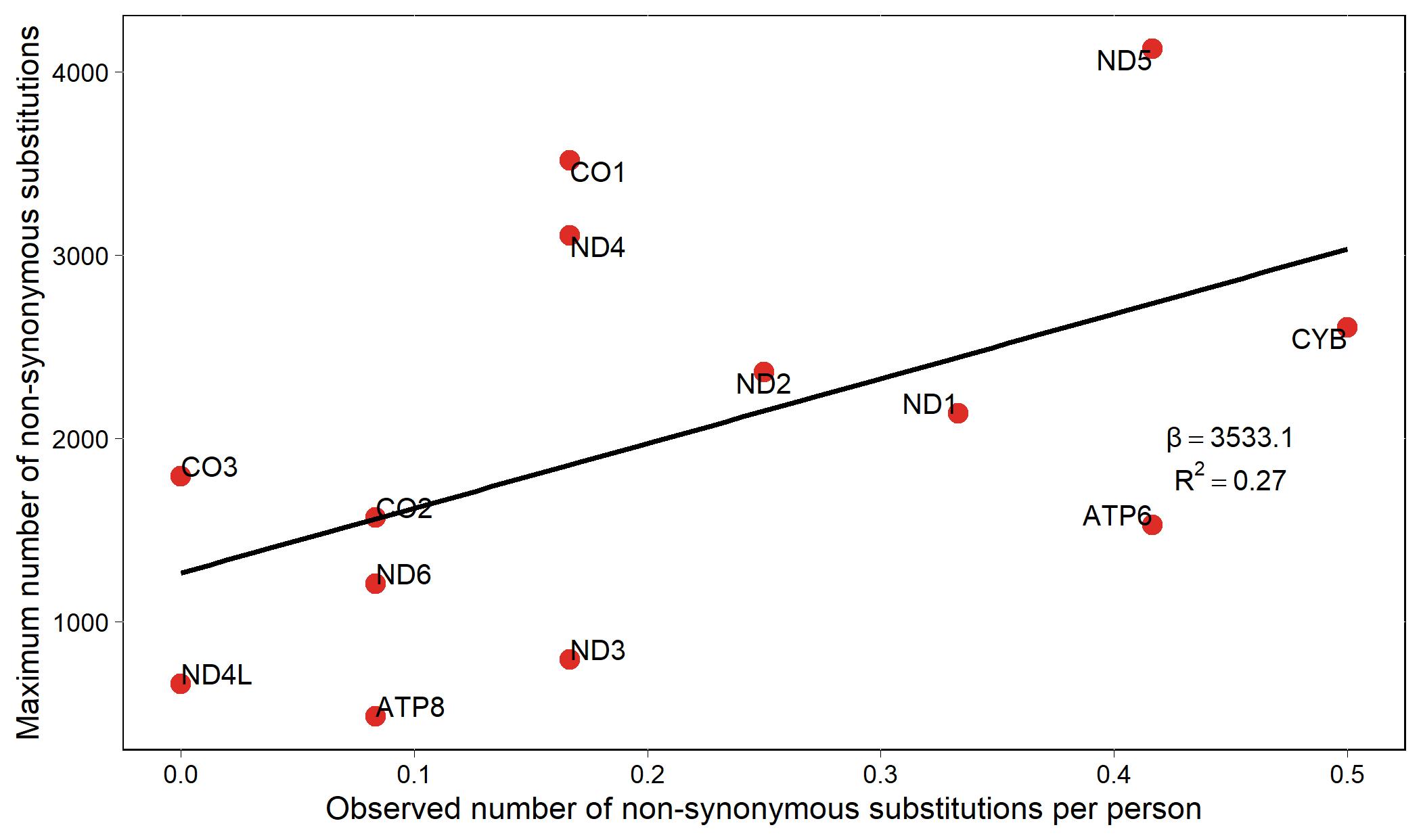 | l  Class 12: Female, Han ethnic, Age ≥60, CD4 <200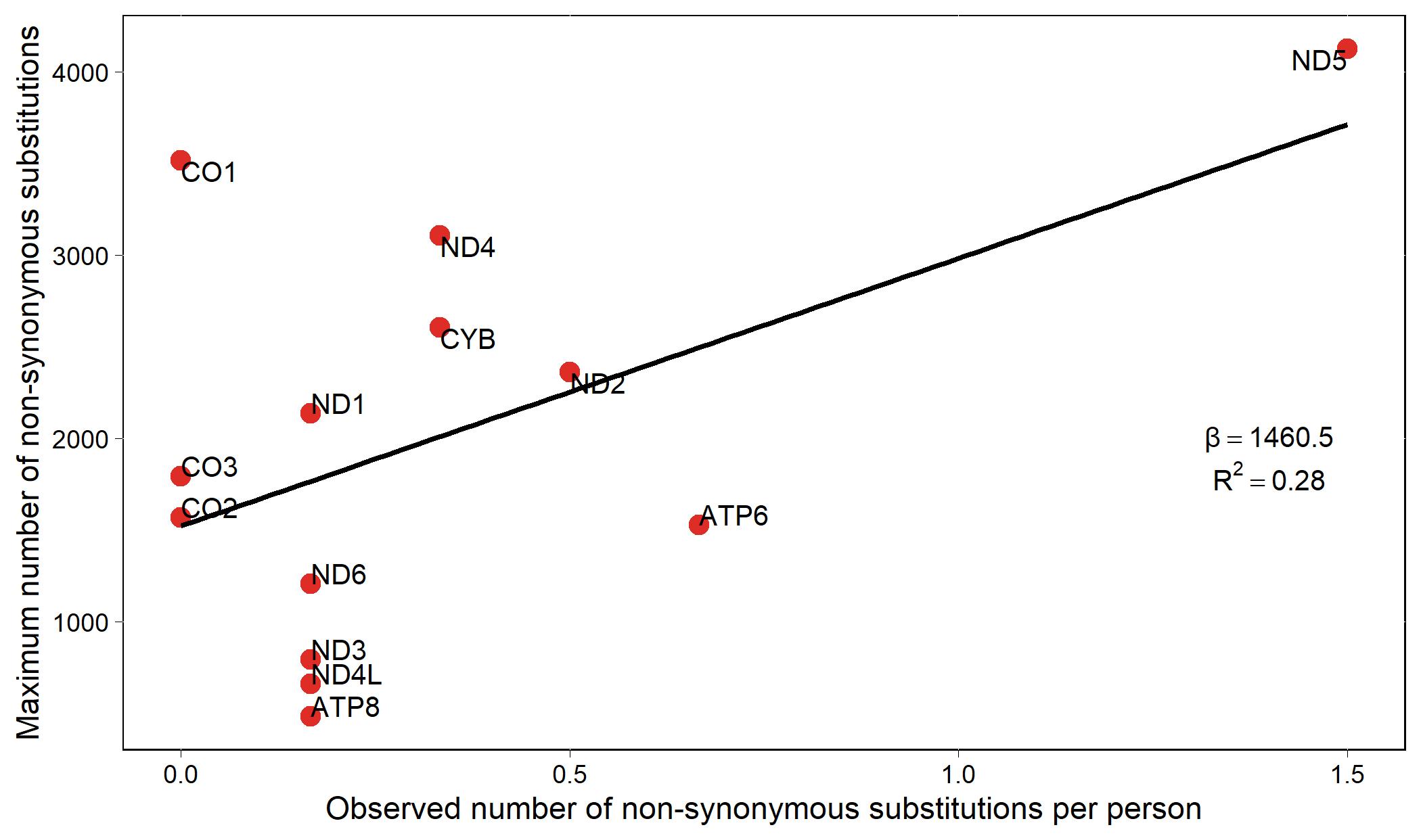 |
| m  Class 13: Female, Han ethnic, Age 17-29, CD4 ≥200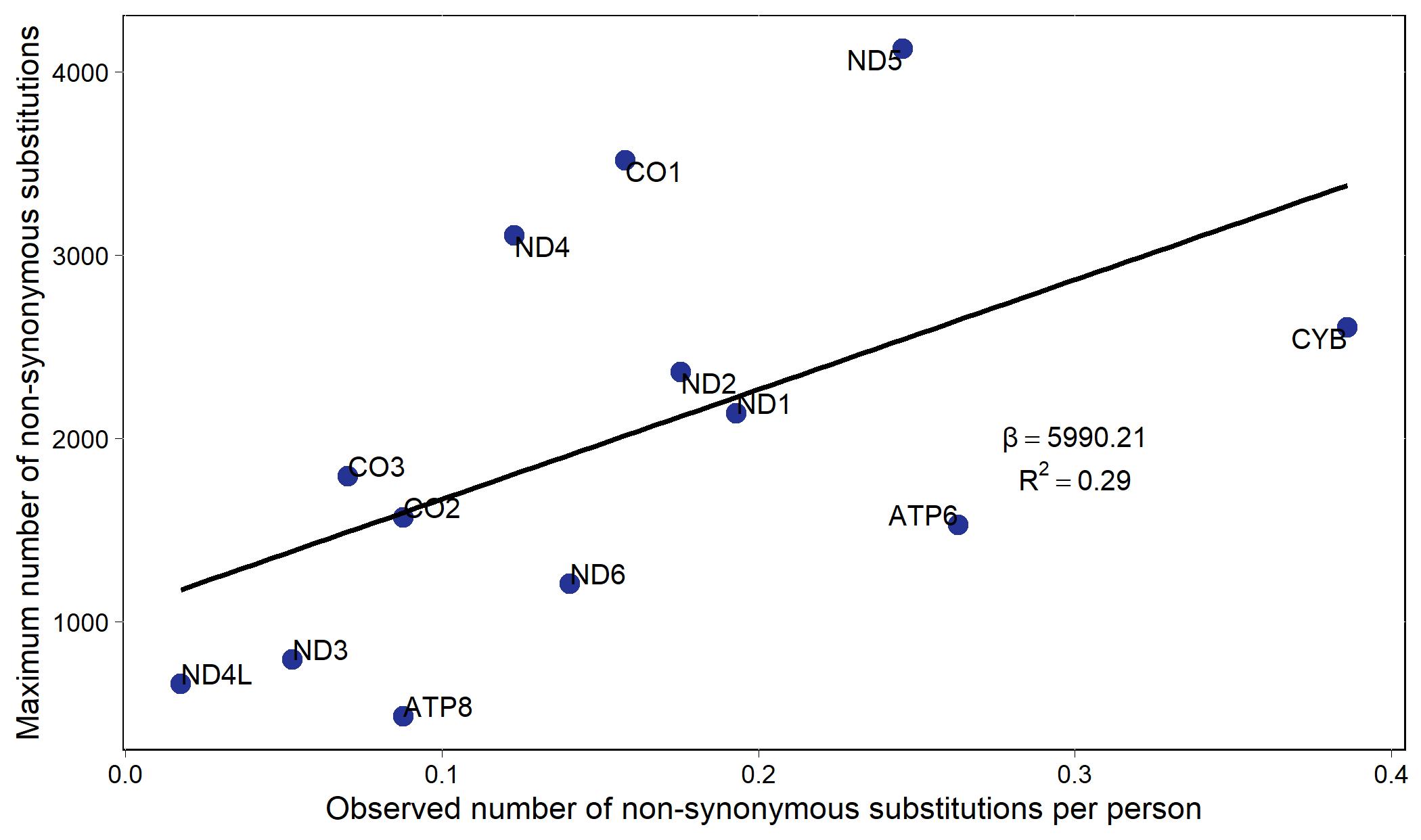 | n  Class 14: Female, Han ethnic, Age 30-44, CD4 ≥200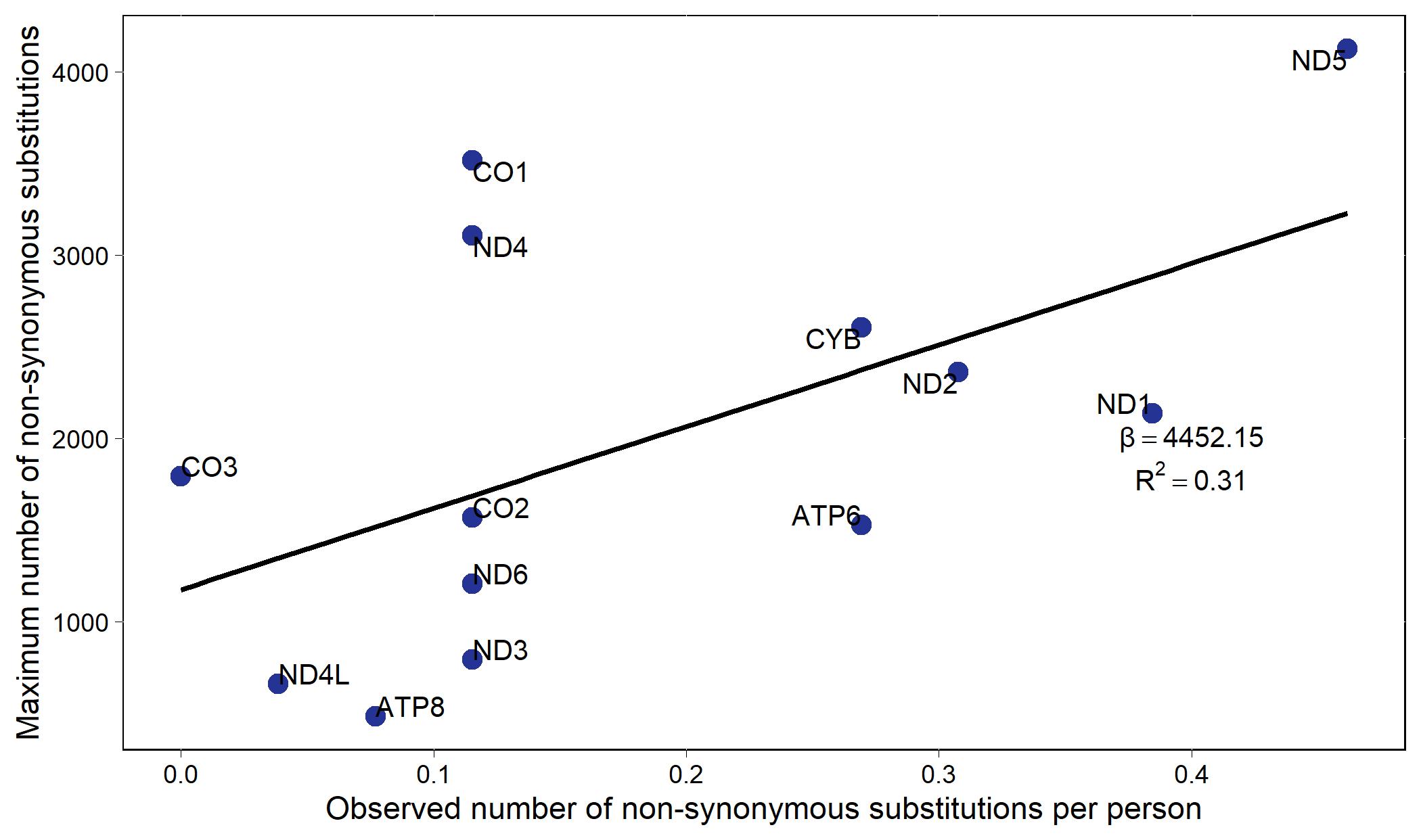 | o  Class 15: Female, Han ethnic, Age 45-59, CD4 ≥200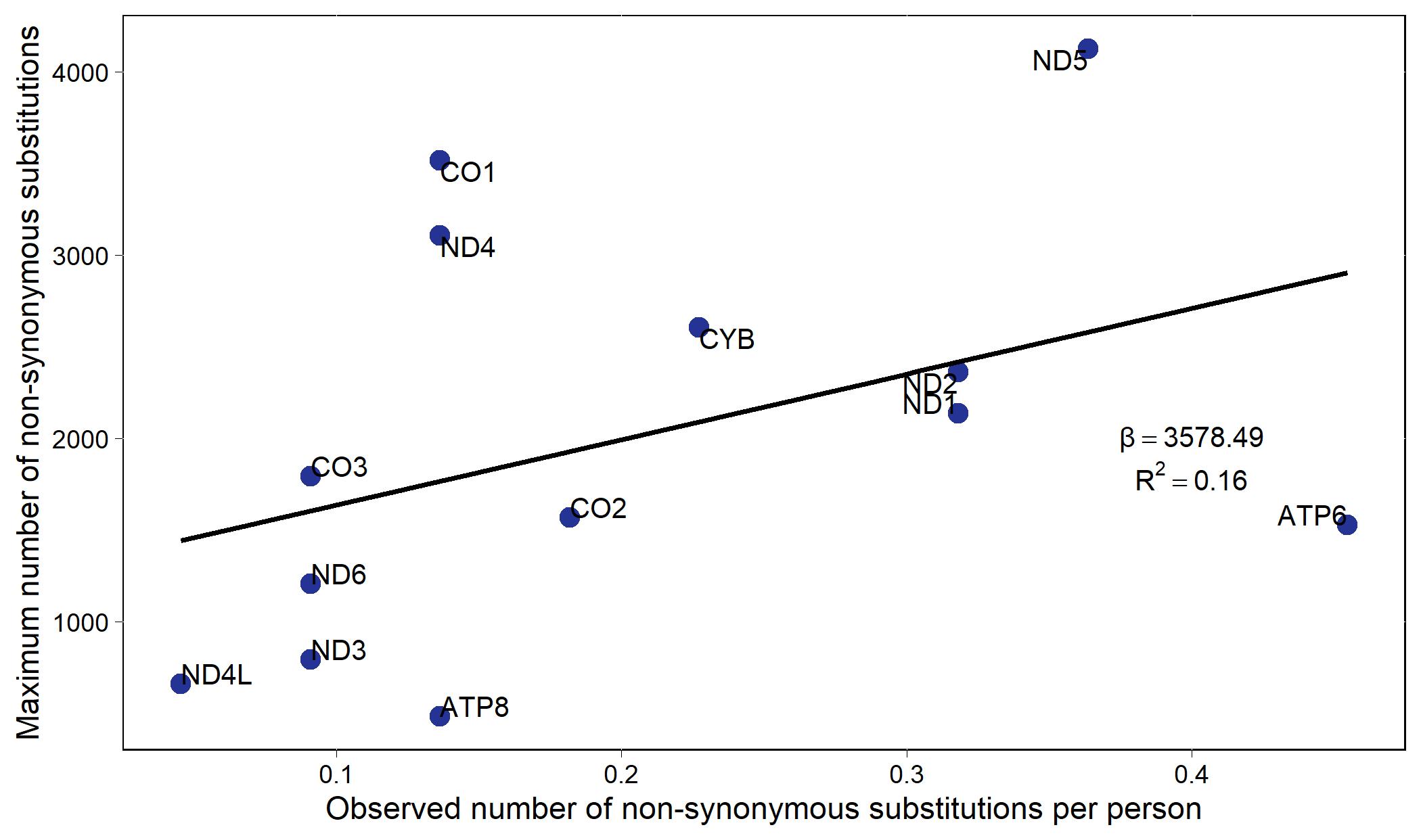 | p  Class 16: Female, Han ethnic, Age ≥60, CD4 ≥200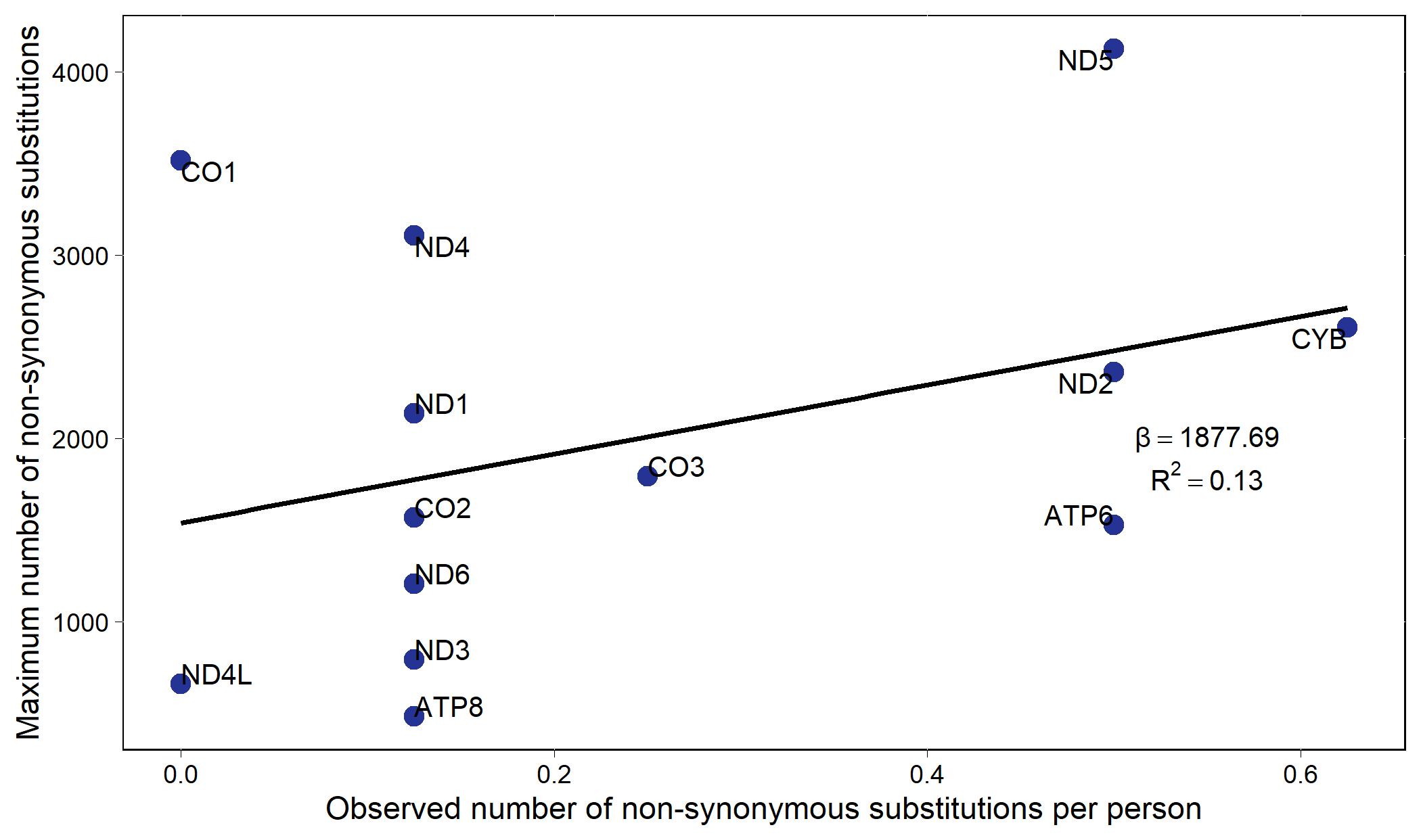 |

**Supplementary Figure 3.** Correlation between observed and maximum non-synonymous substitutions across 16 subpopulations of untreated PLWH.
