# Supplementary material for: Baseline Characteristics of Mitochondrial DNA and Mutations Associated With Short-Term Posttreatment CD4+T-Cell Recovery in Chinese People With HIV
Source: Front Immunol. 2021 Dec 14;12:793375. doi: 10.3389/fimmu.2021.793375 (PMC8712318; doi:10.3389/fimmu.2021.793375)
Supplement: Supplementary file 1 [file DataSheet_1.zip › SupplementaryMaterial/Supplementary Table1.docx]

| **Supplementary Table 1**. Distribution of mutations per division in untreated male and Han ethnic participants | | | | | |
| --- | --- | --- | --- | --- | --- |
| Division | Number of types (or counts) of base variants | Number of types (or counts) of substitutions | Number of types (or counts) of indels | Number of types (or counts) of synonymous substitutions^*^ | Number of types (or counts) of non-synonymous substitutions^*^ |
| Class1: Age 17-29; CD4 <200 (N=55) | | | | | |
| D-loop | 119 (636) | 106 (557) | 13 (79) | - | - |
| RNR1-2 | 38 (248) | 34 (243) | 4 (5) | - | - |
| 22tRNAs | 20 (25) | 20 (25) | 0 (0) | - | - |
| ND1-6, 4L | 191 (588) | 189 (586) | 2 (2) | 131 (436)^*^ | 60 (152)^*^ |
| CO1-3 | 67 (217) | 66 (195) | 1 (22) | 58 (178)^*^ | 9 (18)^*^ |
| ATP6,8 | 27 (137) | 27 (137) | 0 (0) | 16 (26) | 11 (111) |
| CYB | 46 (255) | 46 (255) | 0 (0) | 27 (128) | 19 (127) |
| Class2: Age 30-44; CD4 <200 (N=99) | | | | | |
| D-loop | 193 (1156) | 172 (1017) | 21 (139) | - | - |
| RNR1-2 | 51 (426) | 47 (420) | 4 (6) | - | - |
| 22tRNAs | 28 (50) | 27 (49) | 1 (1) | - | - |
| ND1-6, 4L | 237 (983) | 237 (983) | 0 (0) | 165 (721)^*^ | 77 (267)^*^ |
| CO1-3 | 119 (400) | 117 (358) | 2 (42) | 95 (321)^*^ | 25 (40)^*^ |
| ATP6,8 | 51 (250) | 50 (249) | 1 (1) | 26 (46) | 24 (203) |
| CYB | 56 (424) | 56 (424) | 0 (0) | 32 (190) | 24 (234) |

*Include ambiguous substitutions which are both synonymous and non-synonymous

| (Continue) **Supplementary Table 1**. Distribution of mutations per division in untreated male and Han ethnic participants | | | | | |
| --- | --- | --- | --- | --- | --- |
| Division | Number of types (or counts) of base variants | Number of types (or counts) of substitutions | Number of types (or counts) of indels | Number of types (or counts) of synonymous substitutions^*^ | Number of types (or counts) of non-synonymous substitutions^*^ |
| Class3: Age 45-59; CD4 <200 (N=65) | | | | | |
| D-loop | 130 (739) | 113 (641) | 17 (98) | - | - |
| RNR1-2 | 44 (281) | 41 (276) | 3 (5) | - | - |
| 22tRNAs | 24 (34) | 21 (31) | 3 (3) | - | - |
| ND1-6, 4L | 197 (675) | 194 (672) | 3 (3) | 138 (498)^*^ | 58 (176)^*^ |
| CO1-3 | 65 (242) | 63 (213) | 2 (29) | 55 (198) | 8 (15) |
| ATP6,8 | 37 (178) | 37 (178) | 0 (0) | 22 (29)^*^ | 18 (150)^*^ |
| CYB | 56 (312) | 56 (312) | 0 (0) | 32 (150) | 24 (162) |
| Class4: Age ≥60; CD4 <200 (N=37) | | | | | |
| D-loop | 106 (440) | 91 (381) | 15 (59) | - | - |
| RNR1-2 | 25 (156) | 24 (155) | 1 (1) | - | - |
| 22tRNAs | 20 (21) | 19 (20) | 1 (1) | - | - |
| ND1-6, 4L | 121 (353) | 121 (353) | 0 (0) | 84 (265)^*^ | 39 (90)^*^ |
| CO1-3 | 47 (132) | 46 (116) | 1 (16) | 38 (108) | 8 (8) |
| ATP6,8 | 24 (95) | 24 (95) | 0 (0) | 8 (10) | 16 (85) |
| CYB | 25 (151) | 25 (151) | 0 (0) | 15 (68) | 10 (83) |

*Include ambiguous substitutions which are both synonymous and non-synonymous

| (Continue)  **Supplementary Table 1**. Distribution of mutations per division in untreated male and Han ethnic participants | | | | | |
| --- | --- | --- | --- | --- | --- |
| Division | Number of types (or counts) of base variants | Number of types (or counts) of substitutions | Number of types (or counts) of indels | Number of types (or counts) of synonymous substitutions^*^ | Number of types (or counts) of non-synonymous substitutions^*^ |
| Class5: Age 17-29; CD4 ≥200 (N=97) | | | | | |
| D-loop | 159 (1077) | 147 (967) | 12 (110) | - | - |
| RNR1-2 | 49 (412) | 46 (409) | 3 (3) | - | - |
| 22tRNAs | 29 (44) | 27 (42) | 2 (2) | - | - |
| ND1-6, 4L | 237 (955) | 235 (953) | 2 (2) | 166 (706) | 69 (247) |
| CO1-3 | 98 (381) | 95 (347) | 3 (34) | 72 (305)^*^ | 24 (43)^*^ |
| ATP6,8 | 48 (248) | 48 (248) | 0 (0) | 16 (27) | 32 (221) |
| CYB | 56 (424) | 55 (423) | 1 (1) | 34 (196)^*^ | 22 (228)^*^ |
| Class6: Age 30-44; CD4 ≥200 (N=162) | | | | | |
| D-loop | 232 (1881) | 209 (1653) | 23 (228) | - | - |
| RNR1-2 | 59 (681) | 53 (669) | 6 (12) | - | - |
| 22tRNAs | 57 (83) | 51 (76) | 6 (7) | - | - |
| ND1-6, 4L | 324 (1686) | 319 (1680) | 5 (6) | 232 (1244) | 87 (436) |
| CO1-3 | 158 (675) | 153 (605) | 5 (70) | 122 (547)^*^ | 33 (60)^*^ |
| ATP6,8 | 63 (422) | 63 (422) | 0 (0) | 34 (71)^*^ | 31 (353)^*^ |
| CYB | 75 (751) | 74 (750) | 1 (1) | 45 (374) | 29 (376) |

*Include ambiguous substitutions which are both synonymous and non-synonymous

| (Continue)  **Supplementary Table 1**. Distribution of mutations per division in untreated male and Han ethnic participants | | | | | |
| --- | --- | --- | --- | --- | --- |
| Division | Number of types (or counts) of base variants | Number of types (or counts) of substitutions | Number of types (or counts) of indels | Number of types (or counts) of synonymous substitutions^*^ | Number of types (or counts) of non-synonymous substitutions^*^ |
| Class7: Age 45-59; CD4 ≥200 (N=61) | | | | | |
| D-loop | 132 (685) | 118 (593) | 14 (92) | - | - |
| RNR1-2 | 32 (252) | 28 (245) | 4 (7) | - | - |
| 22tRNAs | 22 (24) | 21 (23) | 1 (1) | - | - |
| ND1-6, 4L | 191 (654) | 188 (651) | 3 (3) | 137 (481)^*^ | 52 (171)^*^ |
| CO1-3 | 73 (251) | 72 (233) | 1 (18) | 60 (212) | 12 (21) |
| ATP6,8 | 38 (165) | 38 (165) | 0 (0) | 18 (23) | 20 (142) |
| CYB | 38 (262) | 37 (261) | 1 (1) | 20 (121) | 17 (140) |
| Class8: Age ≥60; CD4 ≥200 (N=37) | | | | | |
| D-loop | 101 (410) | 90 (372) | 11 (38) | - | - |
| RNR1-2 | 30 (159) | 29 (157) | 1 (2) | - | - |
| 22tRNAs | 11 (11) | 11 (11) | 0 (0) | - | - |
| ND1-6, 4L | 117 (377) | 117 (377) | 0 (0) | 78 (274) | 39 (103) |
| CO1-3 | 45 (133) | 44 (121) | 1 (12) | 37 (112) | 7 (9) |
| ATP6,8 | 21 (96) | 21 (96) | 0 (0) | 6 (12) | 15 (84) |
| CYB | 26 (161) | 26 (161) | 0 (0) | 15 (76) | 11 (85) |

*Include ambiguous substitutions which are both synonymous and non-synonymous

| (Continue) **Supplementary Table 1**. Distribution of mutations per division in untreated female and Han ethnic participants | | | | | |
| --- | --- | --- | --- | --- | --- |
| Division | Number of types (or counts) of base variants | Number of types (or counts) of substitutions | Number of types (or counts) of indels | Number of types (or counts) of synonymous substitutions^*^ | Number of types (or counts) of non-synonymous substitutions^*^ |
| Class9: Age 17-29; CD4 <200 (N=28) | | | | | |
| D-loop | 89 (325) | 78 (275) | 11 (50) | - | - |
| RNR1-2 | 21 (115) | 21 (115) | 0 (0) | - | - |
| 22tRNAs | 11 (12) | 10 (11) | 1 (1) | - | - |
| ND1-6, 4L | 108 (288) | 108 (288) | 0 (0) | 74 (208) | 34 (80) |
| CO1-3 | 52 (127) | 51 (110) | 1 (17) | 44 (102) | 7 (8) |
| ATP6,8 | 20 (69) | 19 (68) | 1 (1) | 8 (9) | 11 (59) |
| CYB | 27 (118) | 27 (118) | 0 (0) | 16 (53)^*^ | 12 (66)^*^ |
| Class10: Age 30-44; CD4 <200 (N=34) | | | | | |
| D-loop | 89 (380) | 78 (328) | 11 (52) | - | - |
| RNR1-2 | 31 (148) | 27 (144) | 4 (4) | - | - |
| 22tRNAs | 9 (9) | 8 (8) | 1 (1) | - | - |
| ND1-6, 4L | 130 (369) | 128 (367) | 2 (2) | 94 (271)^*^ | 35 (97)^*^ |
| CO1-3 | 54 (144) | 52 (128) | 2 (16) | 45 (120) | 7 (8) |
| ATP6,8 | 19 (89) | 19 (89) | 0 (0) | 10 (15) | 9 (74) |
| CYB | 26 (147) | 26 (147) | 0 (0) | 15 (70) | 11 (77) |

*Include ambiguous substitutions which are both synonymous and non-synonymous

| (Continue) **Supplementary Table 1**. Distribution of mutations per division in untreated female and Han ethnic participants | | | | | |
| --- | --- | --- | --- | --- | --- |
| Division | Number of types (or counts) of base variants | Number of types (or counts) of substitutions | Number of types (or counts) of indels | Number of types (or counts) of synonymous substitutions^*^ | Number of types (or counts) of non-synonymous substitutions^*^ |
| Class11: Age 45-59; CD4 <200 (N=12) | | | | | |
| D-loop | 61 (135) | 56 (123) | 5 (12) | - | - |
| RNR1-2 | 18 (55) | 18 (55) | 0 (0) | - | - |
| 22tRNAs | 6 (6) | 6 (6) | 0 (0) | - | - |
| ND1-6, 4L | 65 (111) | 65 (111) | 0 (0) | 48 (85) | 17 (26) |
| CO1-3 | 21 (38) | 20 (36) | 1 (2) | 18 (34)^*^ | 3 (3)^*^ |
| ATP6,8 | 10 (25) | 10 (25) | 0 (0) | 4 (4) | 6 (21) |
| CYB | 17 (45) | 17 (45) | 0 (0) | 11 (18) | 6 (27) |
| Class12: Age ≥60; CD4 <200 (N=6) | | | | | |
| D-loop | 37 (71) | 30 (61) | 7 (10) | - | - |
| RNR1-2 | 9 (25) | 9 (25) | 0 (0) | - | - |
| 22tRNAs | 5 (5) | 4 (4) | 1 (1) | - | - |
| ND1-6, 4L | 41 (72) | 41 (72) | 0 (0) | 23 (45) | 18 (27) |
| CO1-3 | 11 (20) | 10 (18) | 1 (2) | 10 (18) | 0 (0) |
| ATP6,8 | 7 (14) | 7 (14) | 0 (0) | 2 (3) | 5 (11) |
| CYB | 7 (23) | 7 (23) | 0 (0) | 5 (11) | 2 (12) |

*Include ambiguous substitutions which are both synonymous and non-synonymous

| (Continue) **Supplementary Table 1**. Distribution of mutations per division in untreated female and Han ethnic participants | | | | | |
| --- | --- | --- | --- | --- | --- |
| Division | Number of types (or counts) of base variants | Number of types (or counts) of substitutions | Number of types (or counts) of indels | Number of types (or counts) of synonymous substitutions^*^ | Number of types (or counts) of non-synonymous substitutions^*^ |
| Class13: Age 17-29;CD4 ≥200 (N=57) | | | | | |
| D-loop | 125 (663) | 111 (579) | 14 (84) | - | - |
| RNR1-2 | 41 (243) | 37 (237) | 4 (6) | - | - |
| 22tRNAs | 21 (26) | 21 (26) | 0 (0) | - | - |
| ND1-6, 4L | 181 (624) | 179 (622) | 2 (2) | 127 (459)^*^ | 54 (165)^*^ |
| CO1-3 | 83 (261) | 82 (243) | 1 (18) | 64 (216) | 18 (27) |
| ATP6,8 | 36 (159) | 36 (159) | 0 (0) | 17 (24)^*^ | 20 (136)^*^ |
| CYB | 46 (281) | 46 (281) | 0 (0) | 24 (141) | 22 (140) |
| Class14: Age 30-44; CD4 ≥200 (N=26) | | | | | |
| D-loop | 80 (277) | 71 (247) | 9 (30) | - | - |
| RNR1-2 | 23 (113) | 22 (112) | 1 (1) | - | - |
| 22tRNAs | 14 (15) | 13 (14) | 1 (1) | - | - |
| ND1-6, 4L | 107 (255) | 107 (255) | 0 (0) | 67 (183) | 40 (72) |
| CO1-3 | 46 (103) | 45 (93) | 1 (10) | 40 (87)^*^ | 6 (7)^*^ |
| ATP6,8 | 19 (66) | 19 (66) | 0 (0) | 10 (11) | 9 (55) |
| CYB | 22 (111) | 22 (111) | 0 (0) | 15 (54) | 7 (57) |

*Include ambiguous substitutions which are both synonymous and non-synonymous

| (Continue) **Supplementary Table 1**. Distribution of mutations per division in untreated female and Han ethnic participants | | | | | |
| --- | --- | --- | --- | --- | --- |
| Division | Number of types (or counts) of base variants | Number of types (or counts) of substitutions | Number of types (or counts) of indels | Number of types (or counts) of synonymous substitutions | Number of types (or counts) of non-synonymous substitutions |
| Class15: Age 45-59; CD4 ≥200 (N=22) | | | | | |
| D-loop | 75 (233) | 67 (200) | 8 (33) | - | - |
| RNR1-2 | 23 (95) | 23 (95) | 0 (0) | - | - |
| 22tRNAs | 10 (12) | 10 (12) | 0 (0) | - | - |
| ND1-6, 4L | 92 (235) | 92 (235) | 0 (0) | 62 (171) | 30 (64) |
| CO1-3 | 52 (116) | 51 (104) | 1 (12) | 42 (92) | 9 (12) |
| ATP6,8 | 17 (53) | 17 (53) | 0 (0) | 4 (4) | 13 (49) |
| CYB | 12 (85) | 12 (85) | 0 (0) | 7 (38) | 5 (47) |
| Class16: Age ≥60; CD4 ≥200 (N=8) | | | | | |
| D-loop | 36 (83) | 30 (74) | 6 (9) | - | - |
| RNR1-2 | 12 (33) | 11 (32) | 1 (1) | - | - |
| 22tRNAs | 6 (7) | 5 (6) | 1 (1) | - | - |
| ND1-6, 4L | 45 (87) | 44 (86) | 1 (1) | 32 (68) | 12 (18) |
| CO1-3 | 13 (26) | 12 (24) | 1 (2) | 9 (21) | 3 (3) |
| ATP6,8 | 8 (19) | 8 (19) | 0 (0) | 3 (3) | 5 (16) |
| CYB | 11 (38) | 11 (38) | 0 (0) | 6 (19) | 5 (19) |
