# Supplementary material for: Baseline Characteristics of Mitochondrial DNA and Mutations Associated With Short-Term Posttreatment CD4+T-Cell Recovery in Chinese People With HIV
Source: Front Immunol. 2021 Dec 14;12:793375. doi: 10.3389/fimmu.2021.793375 (PMC8712318; doi:10.3389/fimmu.2021.793375)
Supplement: Supplementary file 1 [file DataSheet_1.zip › SupplementaryMaterial/Supplementary Table10.docx]

| **Supplementary Table 10**. Distributions of transitions and transversions across the mtDNA genome and 13 protein-coding genes. | | |
| --- | --- | --- |
| Sub-population | Transition:transversion (global) | Transition:transversion (protein-coding) |
| Class 1: Male, Han ethnic, Age 17-29, CD4 <200 | 15.621:1 | 18.938:1 |
| Class 2: Male, Han ethnic, Age 30-44, CD4 <200 | 11.527:1 | 18.391:1 |
| Class 3: Male, Han ethnic, Age 45-59, CD4 <200 | 17.643:1 | 23.786:1 |
| Class 4: Male, Han ethnic, Age ≥60, CD4 <200 | 19.176:1 | 34.333:1 |
| Class 5: Male, Han ethnic, Age 17-29, CD4 ≥200 | 13.000:1 | 17.391:1 |
| Class 6: Male, Han ethnic, Age 30-44, CD4 ≥200 | 10.718:1 | 15.611:1 |
| Class 7: Male, Han ethnic, Age 45-59, CD4 ≥200 | 11.744:1 | 18.412:1 |
| Class 8: Male, Han ethnic, Age ≥60, CD4 ≥200 | 11.036:1 | 19.700:1 |
| Class 9: Female, Han ethnic, Age 17-29, CD4 <200 | 13.667:1 | 14.385:1 |
| Class 10: Female, Han ethnic, Age 30-44, CD4 <200 | 13.652:1 | 16.231:1 |
| Class 11: Female, Han ethnic, Age 45-59, CD4 <200 | 11.733:1 | 12.750:1 |
| Class 12: Female, Han ethnic, Age ≥60, CD4 <200 | 20.600:1 | 20.333:1 |
| Class 13: Female, Han ethnic, Age 17-29, CD4 ≥200 | 16.345:1 | 18.706:1 |
| Class 14: Female, Han ethnic, Age 30-44, CD4 ≥200 | 11.375:1 | 13.692:1 |
| Class 15: Female, Han ethnic, Age 45-59, CD4 ≥200 | 15.938:1 | 27.500:1 |
| Class 16: Female, Han ethnic, Age ≥60, CD4 ≥200 | 14.125:1 | 17.750:1 |
| Sub-population | A:C:T:G  (transition in protein-coding genes) | A:C:T:G  (transversion in protein-coding genes) |
| Class 1: Male, Han ethnic, Age 17-29, CD4 <200 | 1.131:1.475:1.000:1.361 | 3.500:1.500:1.000:2.000 |
| Class 2: Male, Han ethnic, Age 30-44, CD4 <200 | 1.184:1.345:1.000:1.333 | 3.000:1.000:2.333:1.333 |
| Class 3: Male, Han ethnic, Age 45-59, CD4 <200 | 1.041:1.162:1.000:1.297 | 6.000:3.000:4.000:1.000 |
| Class 4: Male, Han ethnic, Age ≥60, CD4 <200 | 1.386:1.114:1.182:1.000 | 2.000:1.000:2.000:1.000 |
| Class 5: Male, Han ethnic, Age 17-29, CD4 ≥200 | 1.184:1.618:1.000:1.461 | 2.250:1.500:1.000:1.000 |
| (Continue) **Supplementary Table 10**. Distributions of transitions and transversions across the mtDNA genome and 13 protein-coding genes. | | |
| Sub-population | Transition:transversion (global) | Transition:transversion (protein-coding) |
| Class 6: Male, Han ethnic, Age 30-44, CD4 ≥200 | 1.000:1.617:1.140:1.495 | 2.167:1.333:1.500:1.000 |
| Class 7: Male, Han ethnic, Age 45-59, CD4 ≥200 | 1.104:1.299:1.000:1.269 | 2.500:1.500:3.500:1.000 |
| Class 8: Male, Han ethnic, Age ≥60, CD4 ≥200 | 1.023:1.364:1.000:1.091 | 4.000:3.000:2.000:1.000 |
| Class 9: Female, Han ethnic, Age 17-29, CD4 <200 | 1.343:1.571:1.000:1.429 | - |
| Class 10: Female, Han ethnic, Age 30-44, CD4 <200 | 1.000:1.042:1.000:1.354 | 5.000:2.000:5.000:1.000 |
| Class 11: Female, Han ethnic, Age 45-59, CD4 <200 | 2.600:3.300:1.000:3.300 | 3.000:2.000:1.000:2.000 |
| Class 12: Female, Han ethnic, Age ≥60, CD4 <200 | 1.000:1.583:1.250:1.250 | - |
| Class 13: Female, Han ethnic, Age 17-29, CD4 ≥200 | 1.113:1.613:1.000:1.403 | 1.667:1.333:1.000:1.667 |
| Class 14: Female, Han ethnic, Age 30-44, CD4 ≥200 | 1.256:1.154:1.000:1.154 | 5.000:3.000:4.000:1.000 |
| Class 15: Female, Han ethnic, Age 45-59, CD4 ≥200 | 1.167:1.139:1.000:1.278 | - |
| Class 16: Female, Han ethnic, Age ≥60, CD4 ≥200 | 1.000:1.600:1.067:1.067 | 3.000:1.000:2.333:1.333 |
