# Supplementary material for: Baseline Characteristics of Mitochondrial DNA and Mutations Associated With Short-Term Posttreatment CD4+T-Cell Recovery in Chinese People With HIV
Source: Front Immunol. 2021 Dec 14;12:793375. doi: 10.3389/fimmu.2021.793375 (PMC8712318; doi:10.3389/fimmu.2021.793375)
Supplement: Supplementary file 1 [file DataSheet_1.zip › SupplementaryMaterial/Supplementary Table11.docx]

| **Supplementary Table 11a**. Diversity of transitions per person across 13 protein-coding genes in each sub-population. | | |  |
| --- | --- | --- | --- |
| Sub-population | | Plot |  |
| Class 1: Male, Han ethnic, Age 17-29, CD4 <200 | | 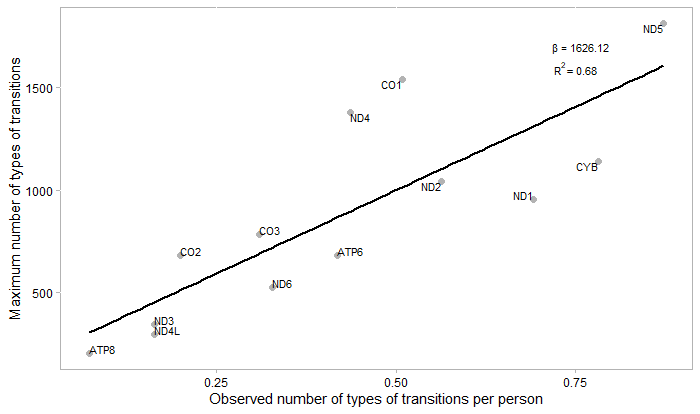 |  |
| Class 2: Male, Han ethnic, Age 30-44, CD4 <200 | | 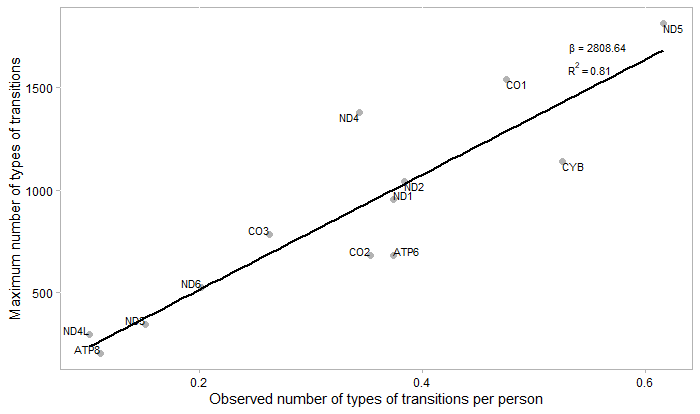 |  |
|  | | |  |
| (Continue) **Supplementary Table 11a**. Diversity of transitions per person across 13 protein-coding genes in each sub-population. | | |  |
| Sub-population | | Plot |  |
| Class 3: Male, Han ethnic, Age 45-59, CD4 <200 | | 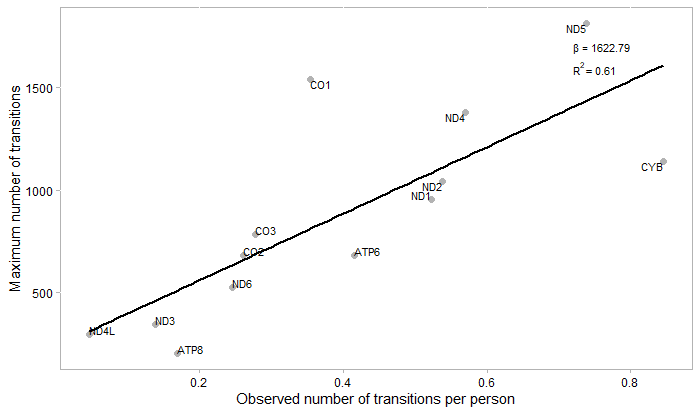 |  |
| Class 4: Male, Han ethnic, Age ≥60, CD4 <200 | | 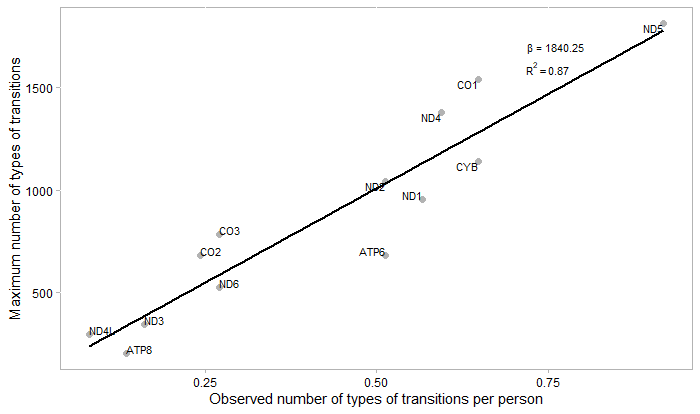 |  |
|  | |  |  |
| (Continue) **Supplementary Table 11a**. Diversity of transitions per person across 13 protein-coding genes in each sub-population. | | |  |
| Sub-population | | Plot |  |
| Class 5: Male, Han ethnic, Age 17-29, CD4 ≥200 | | 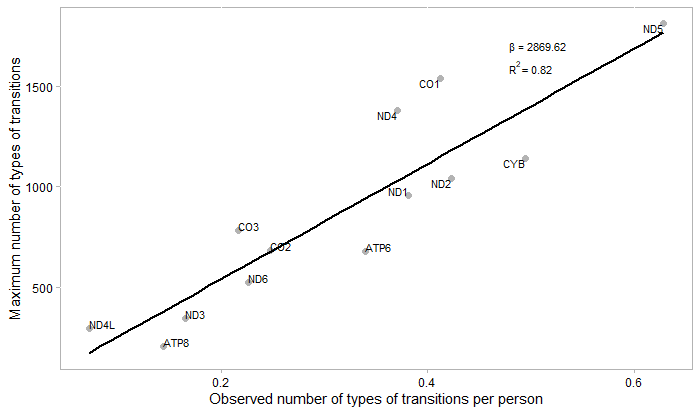 |  |
| Class 6: Male, Han ethnic, Age 30-44, CD4 ≥200 | | 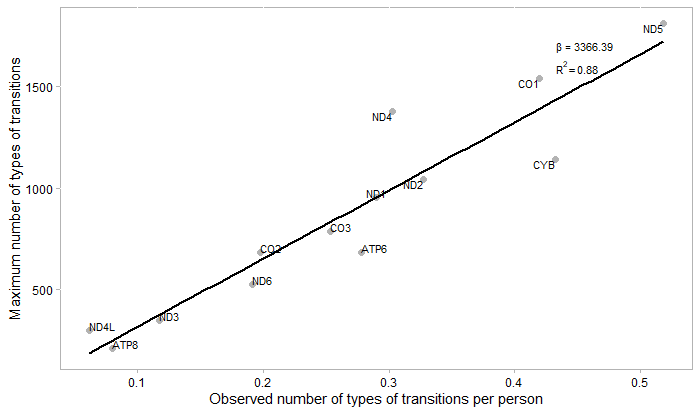 |  |
|  | |  |  |
|  | |  |  |
| (Continue) **Supplementary Table 11a**. Diversity of transitions per person across 13 protein-coding genes in each sub-population. | | |  |
| Sub-population | | Plot |  |
| Class 7: Male, Han ethnic, Age 45-59, CD4 ≥200 | | 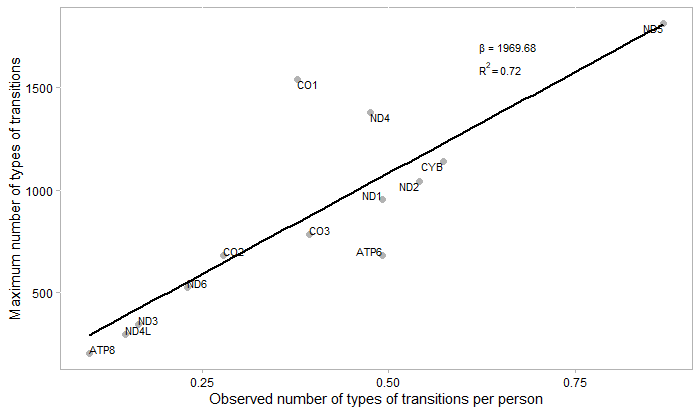 |  |
| Class 8: Male, Han ethnic, Age ≥60, CD4 ≥200 | | 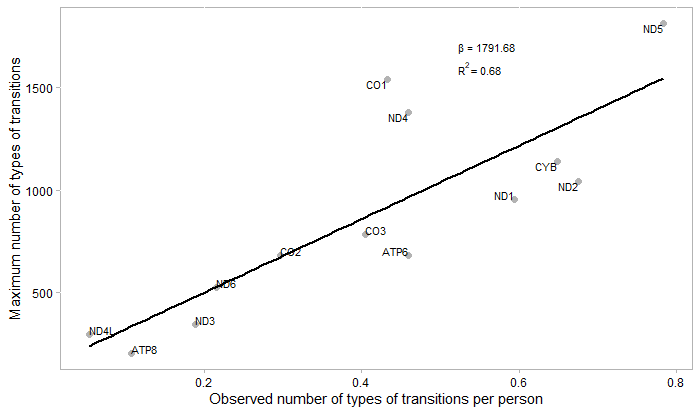 |  |
|  | |  |  |
|  | |  |  |
| (Continue) **Supplementary Table 11a**. Diversity of transitions per person across 13 protein-coding genes in each sub-population. | | |  |
| Sub-population | | Plot |  |
| Class 9: Female, Han ethnic, Age 17-29, CD4 <200 | | 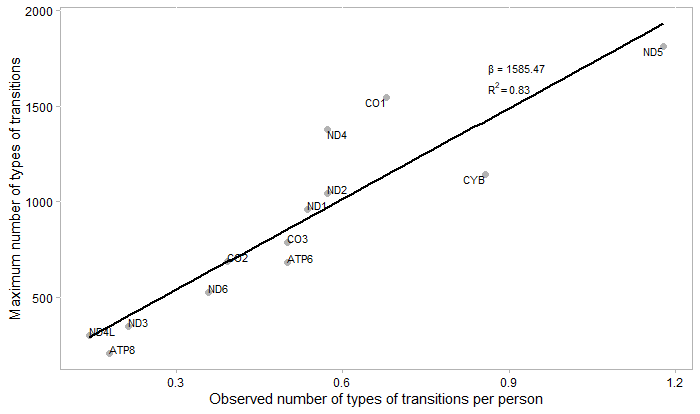 |  |
| Class 10: Female, Han ethnic, Age 30-44, CD4 <200 | | 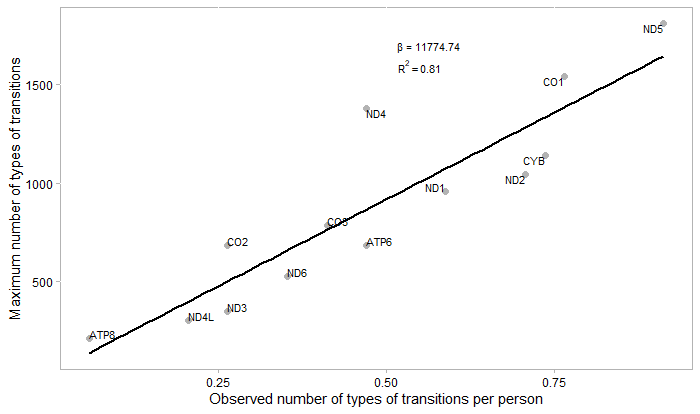 |  |
|  | |  |  |
|  | |  |  |
| (Continue) **Supplementary Table 11a**. Diversity of transitions per person across 13 protein-coding genes in each sub-population. | | |  |
| Sub-population | | Plot |  |
| Class 11: Female, Han ethnic, Age 45-59, CD4 <200 | | 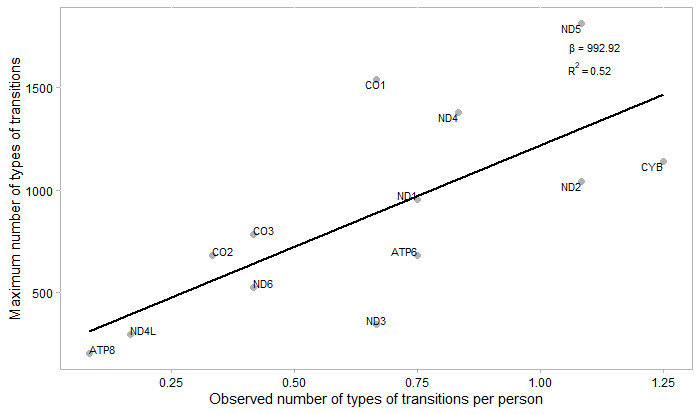 |  |
| Class 12: Female, Han ethnic, Age ≥60, CD4 <200 | | 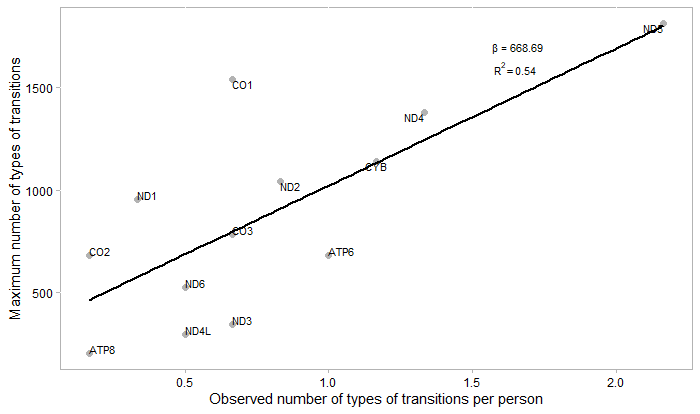 |  |
|  | |  |  |
|  | |  |  |
| (Continue) **Supplementary Table 11a**. Diversity of transitions per person across 13 protein-coding genes in each sub-population. | | |  |
| Sub-population | | Plot |  |
| Class 13: Female, Han ethnic, Age 17-29, CD4 ≥200 | | 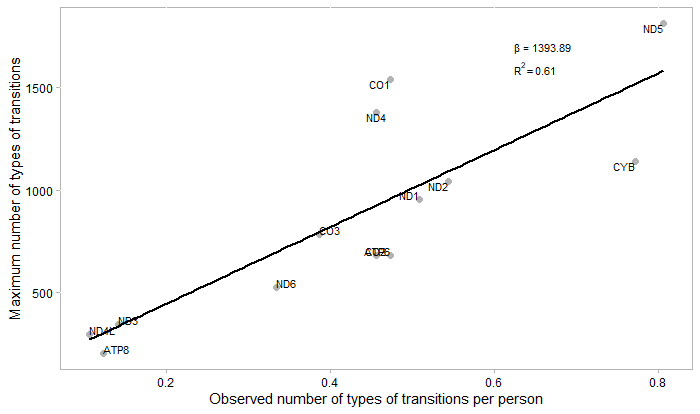 |  |
| Class 14: Female, Han ethnic, Age 30-44, CD4 ≥200 | | 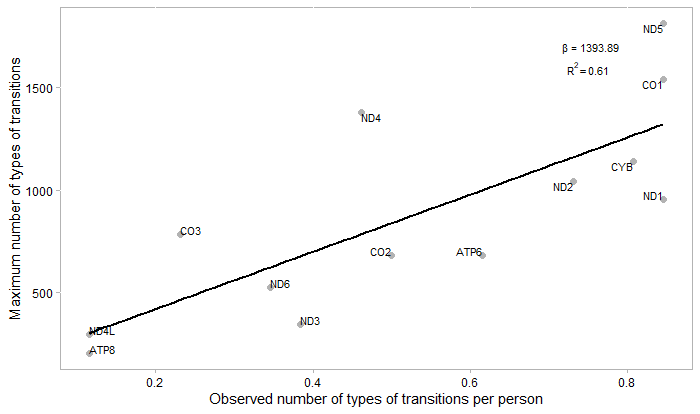 |  |
|  | |  |  |
|  | |  |  |
| (Continue) **Supplementary Table 11a**. Diversity of transitions per person across 13 protein-coding genes in each sub-population. | | |  |
| Sub-population | | Plot |  |
| Class 15: Female, Han ethnic, Age 45-59, CD4 ≥200 | | 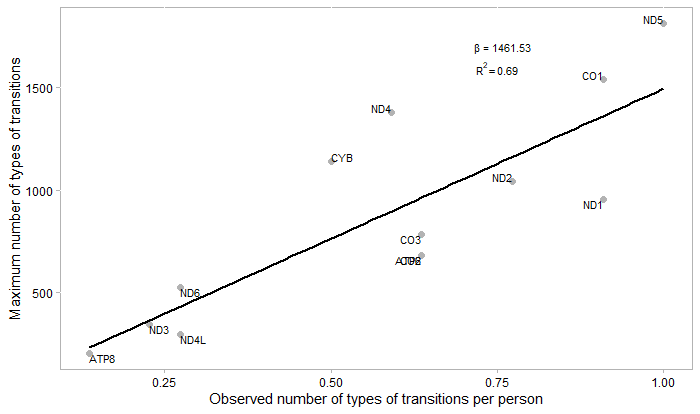 |  |
| Class 16: Female, Han ethnic, Age ≥60, CD4 ≥200 | | 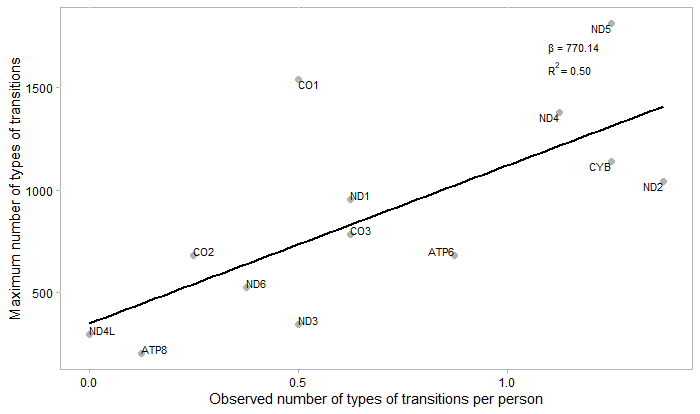 |  |
| Average of linear slopes for classes 1-8 | | 2236.90 |  |
| Average of linear slopes for classes 9-16 | | 1314.85 |  |
| **Supplementary Table 11b**. Diversity of transversions per person across 13 protein-coding genes in each sub-population. | | | |
| Sub-population | Plot | | |
| Class 1: Male, Han ethnic, Age 17-29, CD4 <200 | 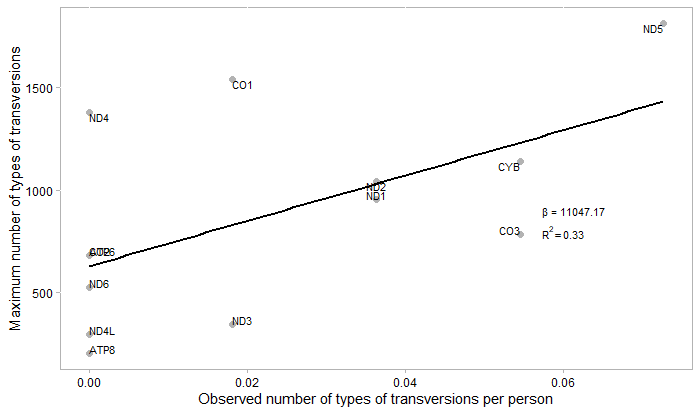 | | |
| Class 2: Male, Han ethnic, Age 30-44, CD4 <200 | 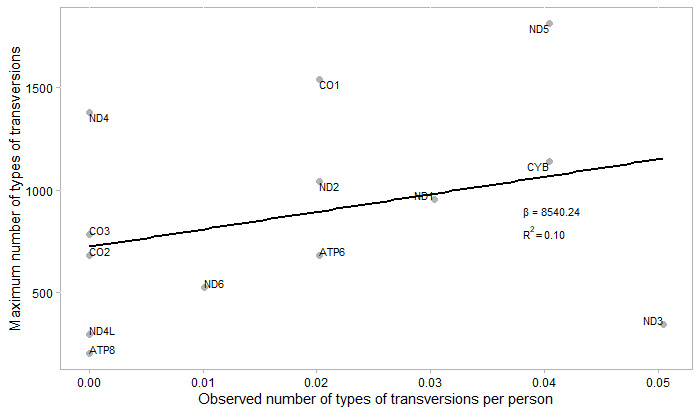 | | |
|  |  | | |
|  |  | | |
| (Continue) **Supplementary Table 11b**. Diversity of transversions per person across 13 protein-coding genes in each sub-population. | | | |
| Sub-population | Plot | | |
| Class 3: Male, Han ethnic, Age 45-59, CD4 <200 | 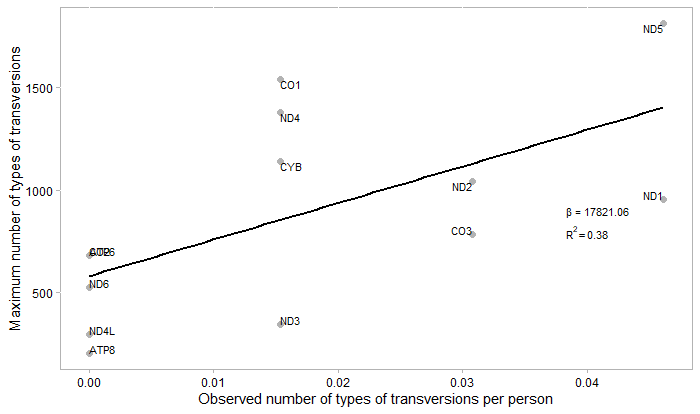 | | |
| Class 4: Male, Han ethnic, Age ≥60, CD4 <200 | 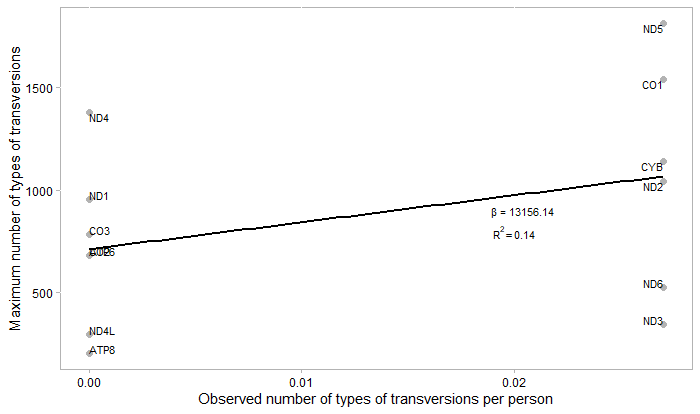 | | |
|  |  | | |
|  |  | | |
| (Continue) **Supplementary Table 11b**. Diversity of transversions per person across 13 protein-coding genes in each sub-population. | | | |
| Sub-population | Plot | | |
| Class 5: Male, Han ethnic, Age 17-29, CD4 ≥200 | 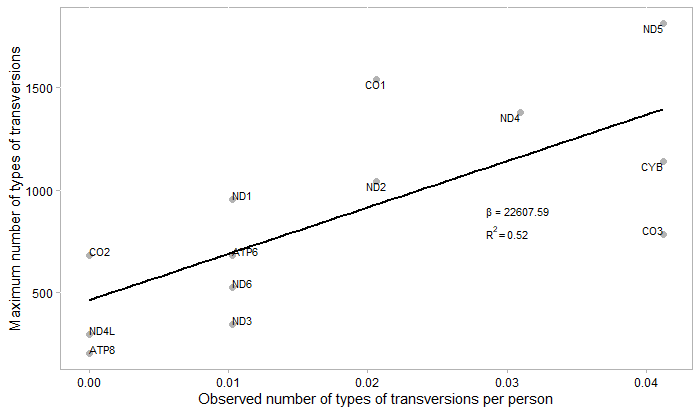 | | |
| Class 6: Male, Han ethnic, Age 30-44, CD4 ≥200 | 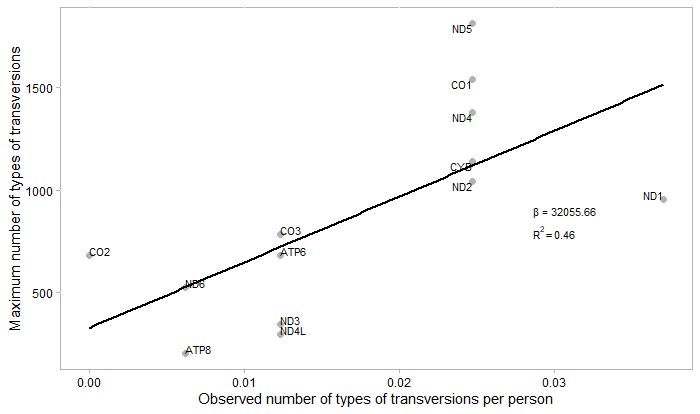 | | |
|  |  | | |
|  |  | | |
| (Continue) **Supplementary Table 11b**. Diversity of transversions per person across 13 protein-coding genes in each sub-population. | | | |
| Sub-population | Plot | | |
| Class 7: Male, Han ethnic, Age 45-59, CD4 ≥200 | 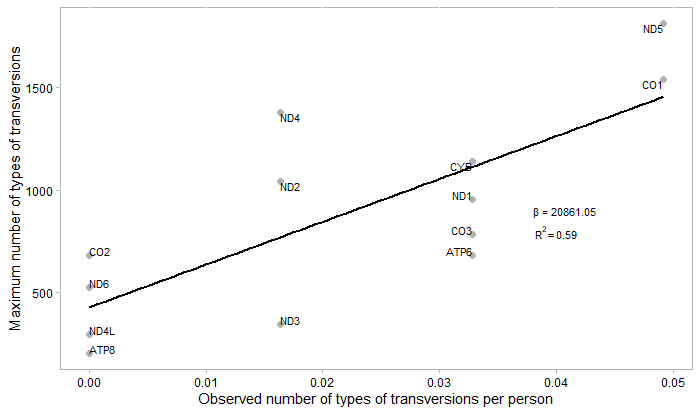 | | |
| Class 8: Male, Han ethnic, Age ≥60, CD4 ≥200 | 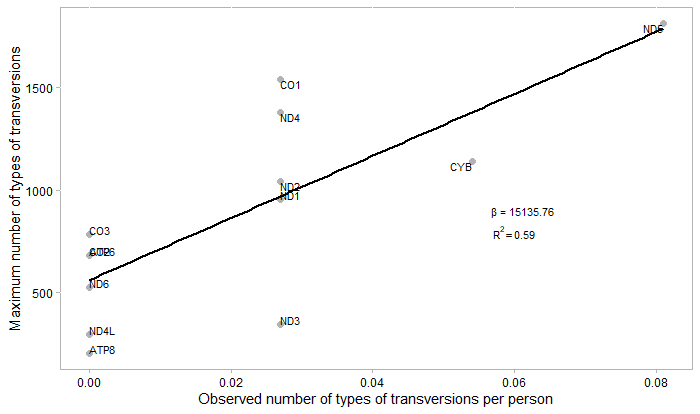 | | |
|  |  | | |
|  |  | | |
| (Continue) **Supplementary Table 11b**. Diversity of transversions per person across 13 protein-coding genes in each sub-population. | | | |
| Sub-population | Plot | | |
| Class 9: Female, Han ethnic, Age 17-29, CD4 <200 | 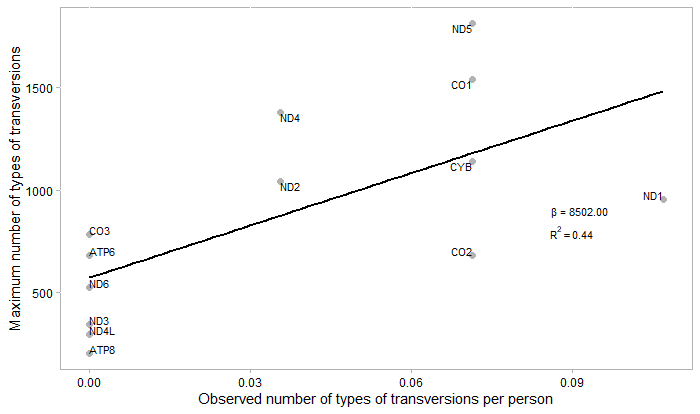 | | |
| Class 10: Female, Han ethnic, Age 30-44, CD4 <200 | 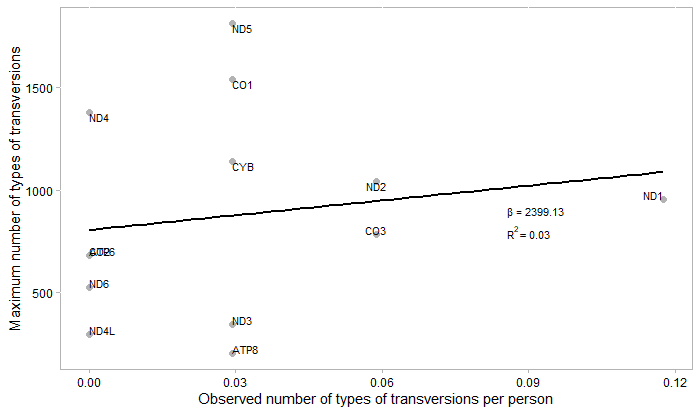 | | |
|  |  | | |
|  |  | | |
| (Continue) **Supplementary Table 11b**. Diversity of transversions per person across 13 protein-coding genes in each sub-population. | | | |
| Sub-population | Plot | | |
| Class 11: Female, Han ethnic, Age 45-59, CD4 <200 | 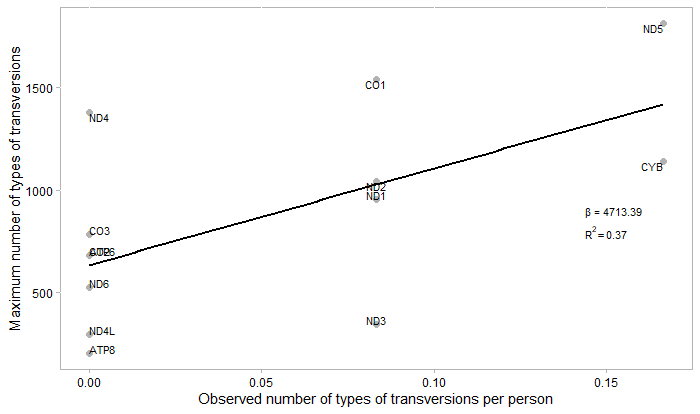 | | |
| Class 12: Female, Han ethnic, Age ≥60, CD4 <200 | 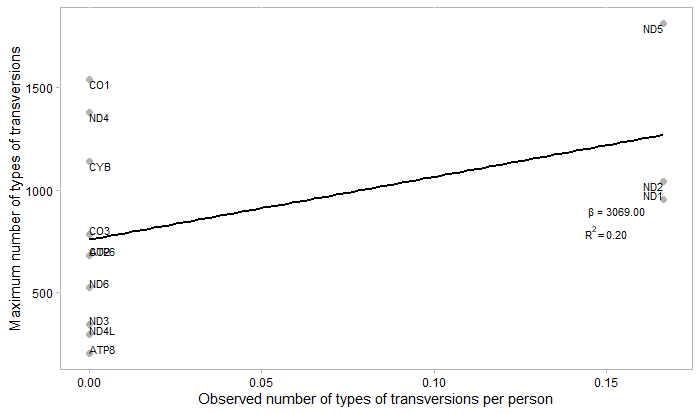 | | |
|  |  | | |
|  |  | | |
| (Continue) **Supplementary Table 11b**. Diversity of transversions per person across 13 protein-coding genes in each sub-population. | | | |
| Sub-population | Plot | | |
| Class 13: Female, Han ethnic, Age 17-29, CD4 ≥200 | 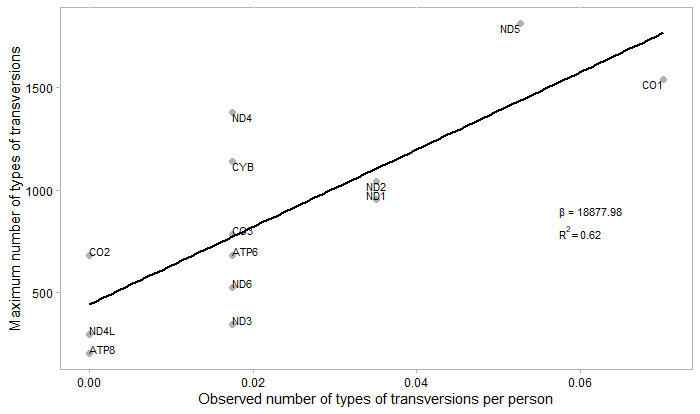 | | |
| Class 14: Female, Han ethnic, Age 30-44, CD4 ≥200 | 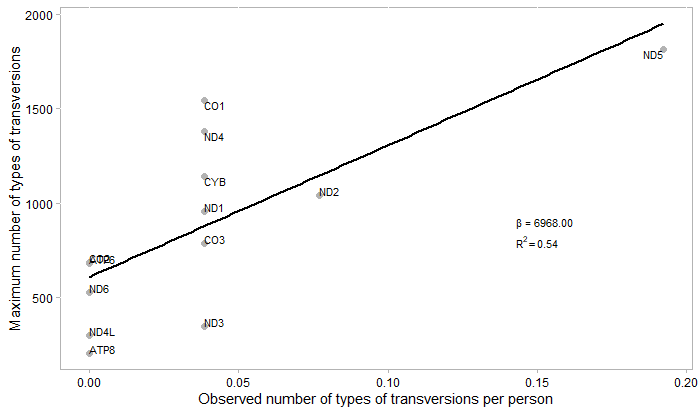 | | |
|  |  | | |
|  |  | | |
| (Continue) **Supplementary Table 11b**. Diversity of transversions per person across 13 protein-coding genes in each sub-population. | | | |
| Sub-population | Plot | | |
| Class 15: Female, Han ethnic, Age 45-59, CD4 ≥200 | 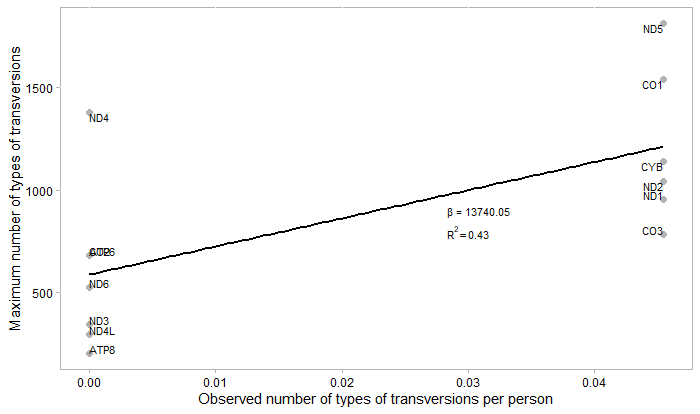 | | |
| Class 16: Female, Han ethnic, Age ≥60, CD4 ≥200 | 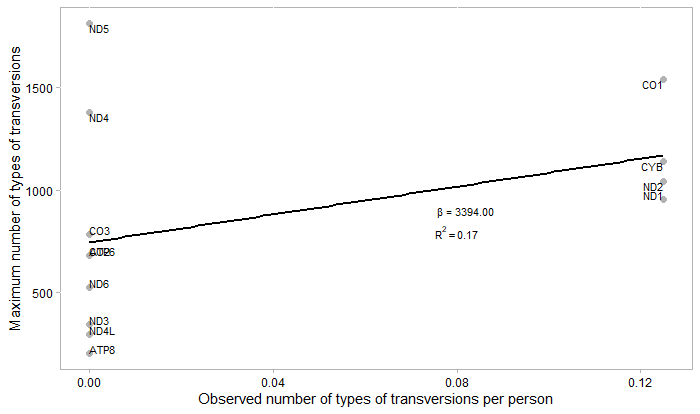 | | |
|  |  | | |
| (Continue) **Supplementary Table 11b**. Diversity of transversions per person across 13 protein-coding genes in each sub-population. | | | |
| Average of linear slopes for classes 1-8 | 17653.08 | | |
| Average of linear slopes for classes 9-16 | 7707.94 | | |
