# Supplementary material for: Baseline Characteristics of Mitochondrial DNA and Mutations Associated With Short-Term Posttreatment CD4+T-Cell Recovery in Chinese People With HIV
Source: Front Immunol. 2021 Dec 14;12:793375. doi: 10.3389/fimmu.2021.793375 (PMC8712318; doi:10.3389/fimmu.2021.793375)
Supplement: Supplementary file 1 [file DataSheet_1.zip › SupplementaryMaterial/Supplementary Table12.docx]

| **Supplementary Table 12**. Bias densities of amino acid changes across sub-populations. | | | | |
| --- | --- | --- | --- | --- |
| Changes to an amino acid | Class1: Male; Age 17-29; CD4 <200 (N=55) | Class2: Male; Age 30-44; CD4 <200 (N=99) | Class3: Male; Age 45-59; CD4 <200 (N=65) | Class4: Male; Age ≥60; CD4 <200 (N=37) |
| A | 0.000233452 | 0.000152583 | 0.000197537 | 0.000204132 |
| C | 0.000040996 | 0.000022776 | 0.000034689 | 0.000030470 |
| D | 0.000039101 | 0.000021723 | 0.000016543 | 0.000000000 |
| E | 0.000000000 | 0.000000000 | 0.000000000 | 0.000044970 |
| F | 0.000033028 | 0.000045872 | 0.000041920 | 0.000073643 |
| G | 0.000044238 | 0.000012288 | 0.000000000 | 0.000000000 |
| H | 0.000093624 | 0.000031208 | 0.000047532 | 0.000083503 |
| I | 0.000146520 | 0.000118400 | 0.000146520 | 0.000158400 |
| K | 0.000020995 | 0.000000000 | 0.000000000 | 0.000000000 |
| L | 0.000015769 | 0.000052564 | 0.000033358 | 0.000046881 |
| M | 0.000039468 | 0.000029236 | 0.000055661 | 0.000058669 |
| N | 0.000051025 | 0.000085041 | 0.000043175 | 0.000101130 |
| P | 0.000011154 | 0.000006197 | 0.000009438 | 0.000016581 |
| Q | 0.000000000 | 0.000000000 | 0.000000000 | 0.000000000 |
| R | 0.000000000 | 0.000007460 | 0.000000000 | 0.000000000 |
| S | 0.000064304 | 0.000049121 | 0.000054411 | 0.000035845 |
| T | 0.000224905 | 0.000190709 | 0.000270433 | 0.000299127 |
| STOP | 0.000022516 | 0.000006254 | 0.000000000 | 0.000000000 |
| V | 0.000126703 | 0.000090502 | 0.000099552 | 0.000134530 |
| W | 0.000000000 | 0.000027750 | 0.000000000 | 0.000000000 |
| Y | 0.000051507 | 0.000014307 | 0.000065374 | 0.000076564 |

| (Continue) **Supplementary Table 12**. Bias densities of amino acid changes across sub-populations. | | | | |
| --- | --- | --- | --- | --- |
| Changes to an amino acid | Class5: Male; Age 17-29; CD4 ≥200 (N=97) | Class6: Male; Age 30-44; CD4 ≥200 (N=162) | Class7: Male; Age 45-59; CD4 ≥200 (N=61) | Class8: Male; Age ≥60; CD4 ≥200 (N=37) |
| A | 0.000155729 | 0.000149192 | 0.000247635 | 0.000326611 |
| C | 0.000011623 | 0.000020878 | 0.000018482 | 0.000030470 |
| D | 0.000022170 | 0.000013275 | 0.000035255 | 0.000000000 |
| E | 0.000000000 | 0.000010271 | 0.000000000 | 0.000000000 |
| F | 0.000037454 | 0.000022426 | 0.000044669 | 0.000098191 |
| G | 0.000025083 | 0.000007510 | 0.000000000 | 0.000000000 |
| H | 0.000053086 | 0.000038143 | 0.000067532 | 0.000083503 |
| I | 0.000083078 | 0.000063311 | 0.000108089 | 0.000178200 |
| K | 0.000035713 | 0.000014256 | 0.000018930 | 0.000000000 |
| L | 0.000040236 | 0.000026769 | 0.000028436 | 0.000046881 |
| M | 0.000052218 | 0.000031266 | 0.000047448 | 0.000039113 |
| N | 0.000038575 | 0.000046195 | 0.000076677 | 0.000050565 |
| P | 0.000037948 | 0.000007574 | 0.000010057 | 0.000016581 |
| Q | 0.000000000 | 0.000007774 | 0.000000000 | 0.000000000 |
| R | 0.000007614 | 0.000004559 | 0.000000000 | 0.000000000 |
| S | 0.000086594 | 0.000049121 | 0.000072473 | 0.000059741 |
| T | 0.000214777 | 0.000124582 | 0.000202783 | 0.000263936 |
| STOP | 0.000000000 | 0.000003822 | 0.000000000 | 0.000000000 |
| V | 0.000071842 | 0.000086033 | 0.000106080 | 0.000080718 |
| W | 0.000000000 | 0.000016958 | 0.000000000 | 0.000000000 |
| Y | 0.000014602 | 0.000017487 | 0.000046440 | 0.000076564 |

| (Continue) **Supplementary Table 12**. Bias densities of amino acid changes across sub-populations. | | | | |
| --- | --- | --- | --- | --- |
| Changes to an amino acid | Class9: Female; Age 17-29; CD4 <200 (N=28) | Class10: Female; Age 30-44; CD4 <200 (N=34) | Class11: Female; Age 45-59; CD4 <200 (N=12) | Class12: Female; Age ≥60; CD4 <200 (N=6) |
| A | 0.000323694 | 0.000222143 | 0.000440584 | 0.000755287 |
| C | 0.000000000 | 0.000000000 | 0.000093950 | 0.000000000 |
| D | 0.000076805 | 0.000000000 | 0.000000000 | 0.000000000 |
| E | 0.000000000 | 0.000000000 | 0.000000000 | 0.000000000 |
| F | 0.000097314 | 0.000026714 | 0.000000000 | 0.000302755 |
| G | 0.000000000 | 0.000000000 | 0.000000000 | 0.000000000 |
| H | 0.000073562 | 0.000060580 | 0.000085822 | 0.000000000 |
| I | 0.000235479 | 0.000172377 | 0.000183150 | 0.000244200 |
| K | 0.000000000 | 0.000067926 | 0.000000000 | 0.000000000 |
| L | 0.000030975 | 0.000025509 | 0.000036138 | 0.000072275 |
| M | 0.000025842 | 0.000021282 | 0.000060299 | 0.000120598 |
| N | 0.000100227 | 0.000055027 | 0.000000000 | 0.000155909 |
| P | 0.000021911 | 0.000018044 | 0.000000000 | 0.000102249 |
| Q | 0.000000000 | 0.000000000 | 0.000000000 | 0.000000000 |
| R | 0.000000000 | 0.000000000 | 0.000000000 | 0.000000000 |
| S | 0.000094733 | 0.000052010 | 0.000110522 | 0.000221043 |
| T | 0.000302269 | 0.000363817 | 0.000488281 | 0.000542535 |
| STOP | 0.000022114 | 0.000000000 | 0.000000000 | 0.000000000 |
| V | 0.000106663 | 0.000117120 | 0.000165920 | 0.000248880 |
| W | 0.000000000 | 0.000000000 | 0.000000000 | 0.000000000 |
| Y | 0.000101174 | 0.000041660 | 0.000118036 | 0.000000000 |

| (Continue) **Supplementary Table 12**. Bias densities of amino acid changes across sub-populations. | | | | |
| --- | --- | --- | --- | --- |
| Changes to an amino acid | Class13: Female; Age 17-29; CD4 ≥200 (N=57) | Class14: Female; Age 30-44; CD4 ≥200 (N=26) | Class15: Female; Age 45-59; CD4 ≥200 (N=22) | Class16: Female; Age ≥60; CD4 ≥200 (N=8) |
| A | 0.000251762 | 0.000232396 | 0.000308981 | 0.000660876 |
| C | 0.000059337 | 0.000043361 | 0.000051245 | 0.000000000 |
| D | 0.000056593 | 0.000000000 | 0.000048876 | 0.000000000 |
| E | 0.000000000 | 0.000000000 | 0.000000000 | 0.000000000 |
| F | 0.000031869 | 0.000069867 | 0.000082570 | 0.000113533 |
| G | 0.000000000 | 0.000046790 | 0.000000000 | 0.000000000 |
| H | 0.000072271 | 0.000118831 | 0.000140436 | 0.000000000 |
| I | 0.000077116 | 0.000253593 | 0.000166500 | 0.000183150 |
| K | 0.000020258 | 0.000044413 | 0.000000000 | 0.000000000 |
| L | 0.000022824 | 0.000050037 | 0.000039423 | 0.000054206 |
| M | 0.000038084 | 0.000055661 | 0.000065781 | 0.000090449 |
| N | 0.000082057 | 0.000071958 | 0.000127562 | 0.000116932 |
| P | 0.000000000 | 0.000000000 | 0.000000000 | 0.000076687 |
| Q | 0.000022096 | 0.000000000 | 0.000000000 | 0.000000000 |
| R | 0.000012957 | 0.000000000 | 0.000000000 | 0.000000000 |
| S | 0.000069803 | 0.000051010 | 0.000080379 | 0.000165782 |
| T | 0.000331232 | 0.000300481 | 0.000473485 | 0.000325521 |
| STOP | 0.000000000 | 0.000000000 | 0.000000000 | 0.000077399 |
| V | 0.000157187 | 0.000229735 | 0.000158378 | 0.000124440 |
| W | 0.000000000 | 0.000000000 | 0.000000000 | 0.000000000 |
| Y | 0.000049699 | 0.000054478 | 0.000064383 | 0.000177054 |
