# Supplementary material for: Baseline Characteristics of Mitochondrial DNA and Mutations Associated With Short-Term Posttreatment CD4+T-Cell Recovery in Chinese People With HIV
Source: Front Immunol. 2021 Dec 14;12:793375. doi: 10.3389/fimmu.2021.793375 (PMC8712318; doi:10.3389/fimmu.2021.793375)
Supplement: Supplementary file 1 [file DataSheet_1.zip › SupplementaryMaterial/Supplementary Table13.docx]

| **Supplementary Table 13**. Comparison of overall distributions of bias densities of amino acid changes between each sub-population of pre-ART CD4 <200 and its counterpart of pre-ART CD4 ≥200 with identical levels of age and gender using the Wilcoxon rank sum test. | | |
| --- | --- | --- |
|  | W | P |
| Class 1 VS Class 5 | 241 | 0.6048 |
| Class 2 VS Class 6 | 228.5 | 0.8405 |
| Class 3 VS Class 7 | 208 | 0.7496 |
| Class 4 VS Class 8 | 229.5 | 0.8167 |
| Class 9 VS Class 13 | 231.5 | 0.7795 |
| Class 10 VS Class 14 | 187 | 0.3884 |
| Class 11 VS Class 15 | 201 | 0.6066 |
| Class 12 VS Class 16 | 220.5 | 1 |

**Class1**: Male; Age 17-29; CD4 <200; **Class2**: Male; Age 30-44; CD4 <200; **Class3**: Male; Age 45-59; CD4 <200; **Class4**: Male; Age ≥60; CD4 <200; **Class5:** Male; Age 17-29; CD4 ≥200; **Class6**: Male; Age 30-44; CD4 ≥200; **Class7**: Male; Age 45-59; CD4 ≥200; **Class8**: Male; Age ≥60; CD4 ≥200; **Class9**: Female; Age 17-29; CD4 <200; **Class10**: Female; Age 30-44; CD4 <200; **Class11**: Female; Age 45-59; CD4 <200; **Class12**: Female; Age ≥60; CD4 <200; **Class13**: Female; Age 17-29; CD4 ≥200; **Class14**: Female; Age 30-44; CD4 ≥200; **Class15**: Female; Age 45-59; CD4 ≥200; **Class16**: Female; Age ≥60; CD4 ≥200
