# Supplementary material for: Baseline Characteristics of Mitochondrial DNA and Mutations Associated With Short-Term Posttreatment CD4+T-Cell Recovery in Chinese People With HIV
Source: Front Immunol. 2021 Dec 14;12:793375. doi: 10.3389/fimmu.2021.793375 (PMC8712318; doi:10.3389/fimmu.2021.793375)
Supplement: Supplementary file 1 [file DataSheet_1.zip › SupplementaryMaterial/Supplementary Table2.docx]

| **Supplementary Table 2a**. Primer pairs for entire mtDNA amplification | | | | |
| --- | --- | --- | --- | --- |
| Primer name | Nucleotide position | Sequence (5’–3’) | Primer length (bp) | Segment length (bp) |
| L13894 | 13866-13894 | ACTTAAAATAAAATCCCCACTATGCACAT | 29 | 4863 |
| H2187 | 2216-2187 | TGTTGAGCTTGAACGCTTTCTTAATTGGTG | 30 |  |
| L1677 | 1653-1677 | TTAACTTGACCGCTCTGAGCTAAAC | 25 | 4829 |
| H6505 | 6532-6505 | AGTAGTATAGTGATGCCAGCAGCTAGGA | 28 |  |
| L5868 | 5840-5868 | CTAACCCCTGTCTTTAGATTTACAGTCCA | 29 | 4851 |
| H10718 | 10746-10718 | GGTTATGTACGTAGTCTAGGCCATATGTG | 29 |  |
| L9877 | 9853-9877 | CTATCTGCTTCATCCGCCAACTAAT | 25 | 4800 |
| H14676 | 14704-14676 | ATTGGTCGTGGTTGTAGTCCGTGCGAGAA | 29 |  |

| **Supplementary Table 2b**. Primer pairs for entire mtDNA sequencing | |  |
| --- | --- | --- |
| Primer name | Nucleotide position | Sequence (5’–3’) |
| L713 | 696-713 | TGCAAGCATCCCCGTTCC |
| H902 | 922-902 | GACTTGGGTTAATCGTGTGAC |
| L1156 | 1138-1156 | GAACACTACGAGCCACAGC |
| H1172 | 1190-1172 | GATATGAAGCACCGCCAGG |
| L2025 | 2004-2025 | GCCTGGTGATAGCTGGTTGTCC |
| H2053 | 2073-2053 | TTAGAGGGTTCTGTGGGCAAA |
| L2415 | 2395-2415 | ACCAACAAGTCATTATTACCC |
| H2426 | 2444-2426 | TGAGCATGCCTGTGTTGGG |
| L3179 | 3160-3179 | AGCGCCTTCCCCCGTAAATG |
| H3274 | 3293-3274 | GGAATTGAACCTCTGACTGT |
| L4210 | 4189-4210 | CCACTCACCCTAGCATTACTTA |
| H4227 | 4247-4227 | ATGCTGGAGATTGTAATGGGT |
| L4499 | 4480-4499 | TGGCCCAACCCGTCATCTAC |
| H4792 | 4813-4792 | ACTCAGAAGTGAAAGGGGGCTA |
| L5278 | 5259-5278 | TGGGCCATTATCGAAGAATT |
| H5442 | 5461-5442 | GCGATGAGTGTGGGGAGGAA |
| L6337 | 6318-6337 | CCTGGAGCCTCCGTAGACCT |
| H6367 | 6387-6367 | TGGCCCCTAAGATAGAGGAGA |
| L7356 | 7337-7356 | GAAGCGAAAAGTCCTAATAG |
| H7406 | 7427-7406 | GGGTTCTTCGAATGTGTGGTAG |
| L8215 | 8196-8215 | ACAGTTTCATGCCCATCGTC |
| H8345 | 11963-11944 | TTTCACTGTAAAGAGGTGTTGG |
| L8581 | 12008-12028 | ACAATCCTAGGCCTACCCG |
| H8861 | 12361-12341 | GAGCGAAAGCCTATAATCACTG |

| (Continue) **Supplementary Table 2b**. Primer pairs for entire mtDNA sequencing | |  |
| --- | --- | --- |
| Primer name | Nucleotide position | Sequence (5’–3’) |
| L9794 | 12553-12572 | GACGGCATCTACGGCTCAACA |
| H9848 | 12897-12878 | GGATGAAGCAGATAGTGAGG |
| L10170 | 13031-13049 | ACATAGAAAAATCCACCCCTTACG |
| H10356 | 13143-13124 | TCACTCATAGGCCAGACTTAG |
| L11004 | 14035-14054 | ACAATCATGGCAAGCCAACG |
| H11081 | 14206-14186 | TCTGTGGCTGTGAATGTTAT |
| L11718 | 14970-14989 | CGCAGTCATTCTCATAATCGCCCACGG |
| H11944 | 15105-15086 | CTAGTATGTTGAGTCCTGTA |
| L12028 | 15372-15391 | GGCTCACTCACCCACCACATT |
| H12341 | 15419-15400 | TGGTTATAGTAGTGTGCATGG |
| L12572 | 16190-16209 | ACAACCCAGCTCTCCCTAAG |
| H12878 | 16517-16498 | TGCTAAGGCGAGGATGAAAC |
| L13049 | 8-29 | GACTCCCCTCAGCCATAGA |
| H13124 | 11963-11944 | ATTTTCTGCTAGGGGGTGGA |
| L14054 | 12008-12028 | TCACAGCACCAAATCTCCAC |
| H14186 | 12361-12341 | TGGTTGAACATTGTTTGTTGG |
| L14989 | 12553-12572 | ATGGCTGAATCATCCGCTAC |
| H15086 | 12897-12878 | AGGAGGATAATGCCGATGTT |
| L15391 | 13031-13049 | TAGGAATCACCTCCCATTCC |
| H15400 | 13143-13124 | TGTAGTAAGGGTGGAAGGTG |
| L16209 | 14035-14054 | CCCCATGCTTACAAGCAAGT |
| H16498 | 14206-14186 | CCTGAAGTAGGAACCAGATG |
| L29 | 14970-14989 | GGTCTATCACCCTATTAACCAC |
