# Supplementary material for: Baseline Characteristics of Mitochondrial DNA and Mutations Associated With Short-Term Posttreatment CD4+T-Cell Recovery in Chinese People With HIV
Source: Front Immunol. 2021 Dec 14;12:793375. doi: 10.3389/fimmu.2021.793375 (PMC8712318; doi:10.3389/fimmu.2021.793375)
Supplement: Supplementary file 1 [file DataSheet_1.zip › SupplementaryMaterial/Supplementary Table3.docx]

| **Supplementary Table 3**. The distribution of mitochondrial DNA mutations per 856 untreated PLWH. | |
| --- | --- |
| Distinctive types of nucleotide variants | Count |
| m.3T>C | 2 |
| m.16A>T | 3 |
| m.36G>A | 1 |
| m.37A>G | 1 |
| m.52T>C | 2 |
| m.53G>A | 2 |
| m.54G>C | 2 |
| m.61C>A | 2 |
| m.61C>T | 1 |
| m.62G>A | 2 |
| m.63T>C | 2 |
| m.64C>T | 3 |
| m.65T>A | 1 |
| m.65T>G | 1 |
| m.66G>A | 2 |
| m.68G>A | 2 |
| m.72T>C | 1 |
| m.73A>G | 856 |
| m.89T>C | 1 |
| m.93A>G | 9 |
| m.94G>A | 7 |
| m.103G>A | 13 |
| m.125T>G | 1 |
| m.131T>C | 4 |
| m.143G>A | 10 |
| m.146T>C | 119 |
| m.146T>A | 7 |
| m.149T>C | 2 |
| m.150C>T | 216 |
| m.150C>Y | 1 |
| m.150C>A | 2 |
| m.151C>T | 26 |
| m.151C>Y | 1 |
| m.152T>C | 214 |
| m.152T>Y | 3 |
| m.153A>G | 16 |
| m.156A>G | 2 |
| m.159T>C | 3 |
| m.165A>G | 1 |
| m.182C>T | 4 |
| m.183A>G | 4 |
| (Continue) **Supplementary Table 3**. The distribution of mitochondrial DNA mutations per 856 untreated PLWH. | |
| Distinctive types of nucleotide variants | Count |
| m.184G>A | 3 |
| m.185G>A | 18 |
| m.188A>G | 2 |
| m.189A>G | 17 |
| m.193A>G | 7 |
| m.194C>T | 16 |
| m.195T>C | 73 |
| m.195T>Y | 2 |
| m.198C>T | 6 |
| m.199T>C | 92 |
| m.199T>Y | 1 |
| m.200A>G | 32 |
| m.202A>G | 4 |
| m.203G>A | 2 |
| m.204T>C | 59 |
| m.204T>Y | 1 |
| m.205G>A | 2 |
| m.207G>A | 46 |
| m.208T>C | 1 |
| m.209T>G | 3 |
| m.210A>G | 27 |
| m.211A>G | 1 |
| m.214A>G | 7 |
| m.215A>G | 9 |
| m.217T>C | 11 |
| m.222C>T | 1 |
| m.225G>A | 2 |
| m.226T>C | 1 |
| m.228G>A | 4 |
| m.228G>T | 2 |
| m.234A>G | 8 |
| m.235A>G | 68 |
| m.237A>G | 2 |
| m.239T>C | 2 |
| m.242C>T | 1 |
| m.245A>G | 1 |
| m.248A>G | 30 |
| m.251G>A | 2 |
| m.252T>C | 1 |
| m.260G>A | 5 |
| (Continue) **Supplementary Table 3**. The distribution of mitochondrial DNA mutations per 856 untreated PLWH. | |
| Distinctive types of nucleotide variants | Count |
| m.263A>G | 843 |
| m.266T>C | 1 |
| m.271C>T | 1 |
| m.275G>A | 2 |
| m.279T>C | 1 |
| m.281A>G | 2 |
| m.292T>A | 1 |
| m.293T>C | 1 |
| m.294T>C | 5 |
| m.298C>T | 16 |
| m.303C>A | 1 |
| m.309C>T | 3 |
| m.310T>C | 481 |
| m.310T>Y | 1 |
| m.316G>A | 5 |
| m.316G>T | 1 |
| m.316G>C | 1 |
| m.317C>A | 1 |
| m.317C>Y | 1 |
| m.318T>C | 8 |
| m.318T>G | 1 |
| m.319T>C | 1 |
| m.324C>G | 5 |
| m.324C>S | 1 |
| m.326A>G | 4 |
| m.328A>G | 1 |
| m.332C>T | 2 |
| m.334T>C | 1 |
| m.340C>T | 1 |
| m.341A>G | 1 |
| m.357A>G | 2 |
| m.362C>A | 1 |
| m.373A>G | 1 |
| m.374A>G | 2 |
| m.385A>G | 3 |
| m.390A>G | 1 |
| m.390A>T | 1 |
| m.392T>C | 1 |
| m.393T>C | 1 |
| m.399T>C | 1 |
| m.400T>A | 1 |
| (Continue) **Supplementary Table 3**. The distribution of mitochondrial DNA mutations per 856 untreated PLWH. | |
| Distinctive types of nucleotide variants | Count |
| m.408T>A | 1 |
| m.418C>G | 1 |
| m.431C>T | 1 |
| m.450T>C | 2 |
| m.451A>T | 1 |
| m.452T>Y | 1 |
| m.453T>A | 2 |
| m.454T>C | 1 |
| m.456C>T | 21 |
| m.466T>C | 1 |
| m.470A>G | 1 |
| m.474T>G | 1 |
| m.477T>C | 1 |
| m.479A>G | 3 |
| m.480T>C | 2 |
| m.481C>T | 1 |
| m.482T>G | 1 |
| m.489T>C | 442 |
| m.496C>T | 1 |
| m.498C>T | 1 |
| m.499G>A | 27 |
| m.501C>A | 1 |
| m.501C>T | 2 |
| m.513G>A | 3 |
| m.518C>A | 1 |
| m.521A>G | 1 |
| m.538A>G | 2 |
| m.548C>T | 4 |
| m.552C>T | 1 |
| m.556A>T | 2 |
| m.567A>C | 6 |
| m.593T>C | 4 |
| m.593T>Y | 1 |
| m.596T>C | 1 |
| m.654T>C | 1 |
| m.663A>G | 63 |
| m.681T>C | 19 |
| m.709G>A | 173 |
| m.710T>C | 2 |
| m.723A>G | 2 |
| m.735A>G | 11 |
| (Continue) **Supplementary Table 3**. The distribution of mitochondrial DNA mutations per 856 untreated PLWH. | |
| Distinctive types of nucleotide variants | Count |
| m.739C>T | 2 |
| m.745A>G | 2 |
| m.747A>G | 3 |
| m.750A>G | 852 |
| m.752C>T | 28 |
| m.769G>A | 3 |
| m.783A>G | 1 |
| m.789T>C | 1 |
| m.792C>T | 1 |
| m.813A>G | 4 |
| m.824T>C | 1 |
| m.827A>G | 36 |
| m.827A>R | 1 |
| m.865A>G | 1 |
| m.911T>C | 1 |
| m.921T>C | 1 |
| m.929A>G | 1 |
| m.930G>A | 7 |
| m.951G>A | 1 |
| m.961T>C | 13 |
| m.980T>C | 4 |
| m.990T>C | 1 |
| m.1005T>C | 34 |
| m.1009C>T | 4 |
| m.1027A>G | 2 |
| m.1041A>G | 8 |
| m.1047A>G | 1 |
| m.1048C>T | 20 |
| m.1095T>C | 4 |
| m.1107T>C | 51 |
| m.1119T>C | 28 |
| m.1187T>C | 1 |
| m.1192C>A | 2 |
| m.1193T>C | 1 |
| m.1236C>T | 1 |
| m.1240A>G | 1 |
| m.1243T>C | 1 |
| m.1275A>G | 1 |
| m.1310C>T | 1 |
| m.1342C>T | 1 |
| m.1377C>A | 1 |
| (Continue) **Supplementary Table 3**. The distribution of mitochondrial DNA mutations per 856 untreated PLWH. | |
| Distinctive types of nucleotide variants | Count |
| m.1381A>C | 2 |
| m.1382A>C | 23 |
| m.1383A>C | 1 |
| m.1393G>R | 1 |
| m.1393G>A | 1 |
| m.1420T>C | 1 |
| m.1438A>G | 837 |
| m.1452T>C | 1 |
| m.1462G>A | 2 |
| m.1462G>T | 1 |
| m.1503G>A | 4 |
| m.1520T>C | 1 |
| m.1535T>C | 1 |
| m.1536A>G | 1 |
| m.1541T>C | 9 |
| m.1555A>G | 2 |
| m.1555A>R | 1 |
| m.1598G>A | 18 |
| m.1607T>C | 2 |
| m.1654T>C | 1 |
| m.1656A>C | 1 |
| m.1664G>A | 3 |
| m.1692A>G | 2 |
| m.1709G>A | 9 |
| m.1709G>T | 2 |
| m.1711C>T | 2 |
| m.1715C>T | 8 |
| m.1716T>C | 1 |
| m.1719G>A | 14 |
| m.1734C>T | 7 |
| m.1735A>G | 1 |
| m.1736A>G | 60 |
| m.1806T>C | 1 |
| m.1809T>C | 2 |
| m.1811A>G | 6 |
| m.1819T>C | 4 |
| m.1824T>C | 33 |
| m.1833C>T | 1 |
| m.1881A>G | 1 |
| m.1888G>A | 1 |
| m.1892A>G | 2 |
| (Continue) **Supplementary Table 3**. The distribution of mitochondrial DNA mutations per 856 untreated PLWH. | |
| Distinctive types of nucleotide variants | Count |
| m.1927G>A | 2 |
| m.1978A>G | 3 |
| m.2000C>T | 1 |
| m.2007T>A | 1 |
| m.2010T>C | 1 |
| m.2056G>A | 3 |
| m.2220A>G | 5 |
| m.2242T>C | 1 |
| m.2246A>G | 1 |
| m.2305T>G | 2 |
| m.2308A>G | 1 |
| m.2322C>A | 3 |
| m.2352T>C | 1 |
| m.2356A>G | 1 |
| m.2361G>A | 3 |
| m.2363A>G | 1 |
| m.2378C>T | 1 |
| m.2379C>T | 1 |
| m.2389C>T | 7 |
| m.2392T>C | 2 |
| m.2404T>C | 3 |
| m.2432A>C | 1 |
| m.2442T>C | 4 |
| m.2581A>G | 1 |
| m.2589A>G | 2 |
| m.2626T>C | 2 |
| m.2706A>G | 852 |
| m.2707A>C | 1 |
| m.2735G>A | 1 |
| m.2755A>G | 2 |
| m.2772C>T | 3 |
| m.2831G>A | 4 |
| m.2833A>G | 1 |
| m.2835C>T | 20 |
| m.2857T>C | 1 |
| m.2885T>C | 1 |
| m.2887T>C | 3 |
| m.2904A>G | 1 |
| m.3010G>A | 136 |
| m.3027T>C | 2 |
| m.3083T>C | 1 |
| (Continue) **Supplementary Table 3**. The distribution of mitochondrial DNA mutations per 856 untreated PLWH. | |
| Distinctive types of nucleotide variants | Count |
| m.3111A>T | 1 |
| m.3116C>T | 2 |
| m.3129A>T | 1 |
| m.3144A>G | 1 |
| m.3150T>C | 2 |
| m.3200T>C | 3 |
| m.3203A>G | 2 |
| m.3204C>T | 3 |
| m.3206C>T | 25 |
| m.3209A>T | 1 |
| m.3221A>G | 4 |
| m.3278T>C | 1 |
| m.3290T>C | 5 |
| m.3316G>A | 28 |
| m.3335T>C | 2 |
| m.3336T>C | 4 |
| m.3338T>C | 4 |
| m.3342C>T | 1 |
| m.3351C>T | 1 |
| m.3364C>T | 1 |
| m.3391G>A | 1 |
| m.3394T>C | 21 |
| m.3396T>C | 2 |
| m.3397A>G | 6 |
| m.3398T>C | 11 |
| m.3421G>A | 3 |
| m.3423T>C | 1 |
| m.3426A>G | 1 |
| m.3434A>G | 6 |
| m.3435C>T | 7 |
| m.3438G>A | 3 |
| m.3447A>G | 2 |
| m.3450C>T | 1 |
| m.3453C>T | 4 |
| m.3456T>C | 2 |
| m.3465A>T | 3 |
| m.3477C>T | 1 |
| m.3483G>A | 11 |
| m.3496G>T | 2 |
| m.3497C>T | 26 |
| m.3504T>C | 7 |
| (Continue) **Supplementary Table 3**. The distribution of mitochondrial DNA mutations per 856 untreated PLWH. | |
| Distinctive types of nucleotide variants | Count |
| m.3505A>R | 1 |
| m.3510C>T | 1 |
| m.3511A>G | 2 |
| m.3523A>G | 2 |
| m.3528C>T | 4 |
| m.3531G>A | 3 |
| m.3535T>C | 1 |
| m.3537A>G | 28 |
| m.3546C>T | 3 |
| m.3548T>C | 1 |
| m.3552T>A | 38 |
| m.3571C>T | 20 |
| m.3573C>T | 3 |
| m.3588C>T | 1 |
| m.3591G>A | 5 |
| m.3593T>C | 1 |
| m.3594C>T | 1 |
| m.3600C>T | 1 |
| m.3603C>T | 3 |
| m.3606A>G | 11 |
| m.3618T>C | 2 |
| m.3621T>C | 2 |
| m.3644T>C | 5 |
| m.3645T>C | 2 |
| m.3648C>T | 1 |
| m.3654C>T | 1 |
| m.3661T>G | 1 |
| m.3666G>A | 3 |
| m.3669A>G | 1 |
| m.3687C>T | 1 |
| m.3705G>A | 3 |
| m.3714A>G | 8 |
| m.3721A>G | 1 |
| m.3735A>G | 1 |
| m.3736G>A | 7 |
| m.3738C>T | 2 |
| m.3744A>G | 2 |
| m.3746C>T | 1 |
| m.3753T>C | 3 |
| m.3759A>G | 8 |
| m.3763A>G | 1 |
| (Continue) **Supplementary Table 3**. The distribution of mitochondrial DNA mutations per 856 untreated PLWH. | |
| Distinctive types of nucleotide variants | Count |
| m.3764C>T | 2 |
| m.3765A>C | 1 |
| m.3768A>G | 1 |
| m.3789C>T | 1 |
| m.3795C>T | 1 |
| m.3828A>G | 2 |
| m.3834G>A | 10 |
| m.3849G>A | 1 |
| m.3865A>G | 1 |
| m.3866T>C | 5 |
| m.3882G>A | 12 |
| m.3892A>G | 1 |
| m.3897C>T | 1 |
| m.3912A>G | 1 |
| m.3918G>A | 4 |
| m.3952G>T | 1 |
| m.3970C>T | 147 |
| m.3988T>A | 1 |
| m.4002T>C | 1 |
| m.4025C>T | 3 |
| m.4038A>G | 2 |
| m.4041A>T | 1 |
| m.4047T>C | 6 |
| m.4048G>A | 58 |
| m.4062T>C | 1 |
| m.4071C>T | 84 |
| m.4079A>G | 2 |
| m.4080T>C | 1 |
| m.4084G>A | 1 |
| m.4086C>T | 44 |
| m.4092G>A | 3 |
| m.4093A>G | 2 |
| m.4102C>T | 1 |
| m.4104A>G | 1 |
| m.4113G>A | 8 |
| m.4117T>C | 2 |
| m.4129A>G | 6 |
| m.4131A>G | 3 |
| m.4135T>C | 3 |
| m.4136A>G | 5 |
| m.4140C>T | 13 |
| (Continue) **Supplementary Table 3**. The distribution of mitochondrial DNA mutations per 856 untreated PLWH. | |
| Distinctive types of nucleotide variants | Count |
| m.4164A>G | 57 |
| m.4167C>T | 4 |
| m.4191A>T | 4 |
| m.4195A>T | 1 |
| m.4200A>T | 4 |
| m.4200A>G | 2 |
| m.4203A>G | 2 |
| m.4205T>A | 1 |
| m.4205T>Y | 1 |
| m.4214G>C | 1 |
| m.4215A>G | 1 |
| m.4216T>C | 14 |
| m.4218T>C | 2 |
| m.4219G>A | 1 |
| m.4231A>G | 1 |
| m.4232T>C | 3 |
| m.4233T>C | 1 |
| m.4243A>G | 2 |
| m.4245C>T | 1 |
| m.4248T>C | 63 |
| m.4254T>C | 1 |
| m.4277T>C | 1 |
| m.4310A>G | 1 |
| m.4313T>C | 2 |
| m.4314T>C | 1 |
| m.4317A>G | 1 |
| m.4343A>G | 2 |
| m.4353T>C | 2 |
| m.4356C>T | 1 |
| m.4385A>T | 2 |
| m.4386T>C | 17 |
| m.4394C>T | 7 |
| m.4418T>C | 2 |
| m.4435A>R | 1 |
| m.4452T>C | 1 |
| m.4454T>C | 3 |
| m.4491G>A | 16 |
| m.4500T>C | 2 |
| m.4502T>C | 1 |
| m.4512G>A | 1 |
| m.4526C>T | 2 |
| (Continue) **Supplementary Table 3**. The distribution of mitochondrial DNA mutations per 856 untreated PLWH. | |
| Distinctive types of nucleotide variants | Count |
| m.4538C>T | 2 |
| m.4553T>C | 1 |
| m.4592T>C | 1 |
| m.4622T>C | 1 |
| m.4643G>A | 2 |
| m.4652C>T | 1 |
| m.4655G>A | 2 |
| m.4670C>T | 2 |
| m.4670C>G | 1 |
| m.4679T>C | 7 |
| m.4688T>C | 3 |
| m.4695T>C | 2 |
| m.4703T>C | 3 |
| m.4706A>G | 2 |
| m.4715A>G | 91 |
| m.4721A>G | 6 |
| m.4722A>G | 1 |
| m.4728A>G | 1 |
| m.4732A>G | 8 |
| m.4740A>G | 1 |
| m.4748C>T | 1 |
| m.4754A>G | 1 |
| m.4760A>G | 1 |
| m.4768T>C | 1 |
| m.4769A>G | 855 |
| m.4772T>C | 4 |
| m.4778A>G | 1 |
| m.4793A>G | 2 |
| m.4799C>T | 1 |
| m.4802T>C | 1 |
| m.4811A>G | 7 |
| m.4814C>A | 1 |
| m.4820G>A | 27 |
| m.4823T>C | 6 |
| m.4824A>G | 69 |
| m.4826C>T | 2 |
| m.4833A>G | 36 |
| m.4841G>A | 1 |
| m.4850C>T | 27 |
| m.4853G>A | 5 |
| m.4865A>M | 1 |
| (Continue) **Supplementary Table 3**. The distribution of mitochondrial DNA mutations per 856 untreated PLWH. | |
| Distinctive types of nucleotide variants | Count |
| m.4883C>T | 187 |
| m.4890A>G | 1 |
| m.4895A>G | 4 |
| m.4905T>G | 1 |
| m.4907T>C | 3 |
| m.4917A>G | 1 |
| m.4923A>G | 1 |
| m.4924G>A | 2 |
| m.4931C>T | 1 |
| m.4959G>A | 2 |
| m.4961A>G | 1 |
| m.4977T>C | 1 |
| m.4991G>A | 5 |
| m.4994A>G | 1 |
| m.5021T>C | 1 |
| m.5033A>G | 1 |
| m.5036A>G | 1 |
| m.5046G>A | 1 |
| m.5048T>C | 5 |
| m.5054G>A | 7 |
| m.5054G>C | 2 |
| m.5057C>T | 1 |
| m.5060C>T | 1 |
| m.5063T>C | 3 |
| m.5074T>C | 1 |
| m.5076C>T | 1 |
| m.5081T>C | 1 |
| m.5090T>C | 3 |
| m.5093T>C | 4 |
| m.5094A>R | 1 |
| m.5100C>T | 7 |
| m.5108T>C | 40 |
| m.5121C>T | 1 |
| m.5126C>S | 1 |
| m.5129C>T | 1 |
| m.5130T>C | 3 |
| m.5141C>T | 1 |
| m.5147G>A | 18 |
| m.5153A>G | 20 |
| m.5177G>A | 2 |
| m.5178C>A | 188 |
| (Continue) **Supplementary Table 3**. The distribution of mitochondrial DNA mutations per 856 untreated PLWH. | |
| Distinctive types of nucleotide variants | Count |
| m.5181A>G | 1 |
| m.5186A>C | 1 |
| m.5189A>G | 2 |
| m.5191C>T | 1 |
| m.5196T>C | 1 |
| m.5196T>Y | 1 |
| m.5201T>C | 3 |
| m.5205T>C | 1 |
| m.5222A>G | 1 |
| m.5225A>G | 1 |
| m.5231G>A | 55 |
| m.5231G>R | 1 |
| m.5237G>A | 3 |
| m.5252G>A | 4 |
| m.5258A>G | 2 |
| m.5261G>A | 6 |
| m.5262G>A | 1 |
| m.5263C>T | 8 |
| m.5285A>G | 1 |
| m.5291T>C | 2 |
| m.5301A>G | 52 |
| m.5302T>C | 2 |
| m.5303C>A | 1 |
| m.5312C>T | 1 |
| m.5315A>G | 1 |
| m.5319A>G | 7 |
| m.5321C>T | 1 |
| m.5351A>G | 57 |
| m.5372A>G | 1 |
| m.5399C>A | 1 |
| m.5417G>A | 44 |
| m.5420T>C | 1 |
| m.5424C>T | 1 |
| m.5426T>C | 1 |
| m.5438C>G | 1 |
| m.5441A>G | 4 |
| m.5441A>T | 4 |
| m.5442T>C | 26 |
| m.5459C>T | 1 |
| m.5460G>A | 69 |
| m.5460G>R | 1 |
| (Continue) **Supplementary Table 3**. The distribution of mitochondrial DNA mutations per 856 untreated PLWH. | |
| Distinctive types of nucleotide variants | Count |
| m.5465T>C | 22 |
| m.5466A>G | 2 |
| m.5480A>G | 1 |
| m.5483T>G | 1 |
| m.5492T>C | 5 |
| m.5508T>C | 2 |
| m.5514A>G | 4 |
| m.5528T>C | 1 |
| m.5553T>C | 1 |
| m.5558A>G | 1 |
| m.5563G>A | 2 |
| m.5566A>G | 1 |
| m.5580T>C | 1 |
| m.5581A>G | 2 |
| m.5582A>C | 1 |
| m.5582A>G | 1 |
| m.5585G>A | 9 |
| m.5585G>R | 1 |
| m.5587T>C | 10 |
| m.5601C>T | 25 |
| m.5604C>T | 1 |
| m.5604C>Y | 1 |
| m.5605A>R | 1 |
| m.5628T>C | 9 |
| m.5711A>G | 1 |
| m.5744G>A | 1 |
| m.5747A>G | 2 |
| m.5773G>A | 3 |
| m.5774T>C | 1 |
| m.5783G>A | 1 |
| m.5786T>C | 1 |
| m.5802T>C | 5 |
| m.5813A>C | 1 |
| m.5814T>C | 4 |
| m.5821G>A | 30 |
| m.5893C>T | 3 |
| m.5894A>T | 1 |
| m.5894A>G | 7 |
| m.5894A>C | 1 |
| m.5895C>G | 1 |
| m.5910G>A | 1 |
| (Continue) **Supplementary Table 3**. The distribution of mitochondrial DNA mutations per 856 untreated PLWH. | |
| Distinctive types of nucleotide variants | Count |
| m.5911C>T | 3 |
| m.5913G>A | 6 |
| m.5951A>G | 3 |
| m.5964T>C | 6 |
| m.5978A>G | 8 |
| m.5979G>A | 1 |
| m.5979G>R | 1 |
| m.5985G>A | 1 |
| m.5987C>T | 5 |
| m.6023G>A | 14 |
| m.6026G>A | 8 |
| m.6032G>A | 2 |
| m.6040A>G | 1 |
| m.6071T>C | 1 |
| m.6092T>C | 2 |
| m.6098A>G | 1 |
| m.6101C>T | 1 |
| m.6113A>G | 1 |
| m.6125A>G | 6 |
| m.6164C>T | 1 |
| m.6179G>A | 29 |
| m.6182G>A | 2 |
| m.6185T>C | 5 |
| m.6194A>G | 1 |
| m.6216T>C | 10 |
| m.6228C>T | 3 |
| m.6239G>A | 2 |
| m.6242C>T | 1 |
| m.6245A>G | 1 |
| m.6253T>C | 18 |
| m.6254A>G | 1 |
| m.6260G>A | 5 |
| m.6261G>A | 1 |
| m.6266A>G | 1 |
| m.6267G>A | 1 |
| m.6281A>G | 1 |
| m.6284A>G | 1 |
| m.6293T>C | 3 |
| m.6302A>G | 2 |
| m.6320T>A | 2 |
| m.6320T>K | 1 |
| (Continue) **Supplementary Table 3**. The distribution of mitochondrial DNA mutations per 856 untreated PLWH. | |
| Distinctive types of nucleotide variants | Count |
| m.6324G>C | 1 |
| m.6326C>T | 1 |
| m.6338A>G | 7 |
| m.6340C>T | 1 |
| m.6351T>C | 1 |
| m.6365T>C | 1 |
| m.6378T>C | 1 |
| m.6392T>C | 129 |
| m.6395C>T | 1 |
| m.6413T>C | 13 |
| m.6427C>T | 1 |
| m.6443A>G | 2 |
| m.6446G>A | 8 |
| m.6455C>T | 84 |
| m.6480G>A | 1 |
| m.6482C>T | 1 |
| m.6485A>G | 1 |
| m.6488T>C | 3 |
| m.6497T>C | 1 |
| m.6515T>C | 3 |
| m.6524T>C | 4 |
| m.6531C>T | 6 |
| m.6557C>M | 1 |
| m.6575A>G | 2 |
| m.6578A>G | 1 |
| m.6591C>T | 1 |
| m.6599A>G | 9 |
| m.6605A>G | 1 |
| m.6620T>C | 4 |
| m.6626T>C | 2 |
| m.6641T>C | 2 |
| m.6647A>G | 1 |
| m.6650A>G | 1 |
| m.6653C>T | 2 |
| m.6663A>G | 1 |
| m.6671T>C | 3 |
| m.6674T>C | 1 |
| m.6680T>C | 58 |
| m.6683C>T | 1 |
| m.6689C>T | 1 |
| m.6707T>C | 1 |
| (Continue) **Supplementary Table 3**. The distribution of mitochondrial DNA mutations per 856 untreated PLWH. | |
| Distinctive types of nucleotide variants | Count |
| m.6710A>G | 1 |
| m.6716A>G | 2 |
| m.6719T>C | 1 |
| m.6722G>A | 2 |
| m.6728A>G | 1 |
| m.6734G>A | 2 |
| m.6737A>G | 4 |
| m.6752A>G | 23 |
| m.6755G>A | 1 |
| m.6761C>T | 1 |
| m.6779A>G | 1 |
| m.6782T>C | 2 |
| m.6791A>G | 2 |
| m.6815T>C | 2 |
| m.6842T>C | 2 |
| m.6857C>T | 1 |
| m.6863A>G | 6 |
| m.6881A>G | 1 |
| m.6887C>T | 1 |
| m.6895A>R | 2 |
| m.6896T>C | 1 |
| m.6899G>A | 4 |
| m.6902A>G | 1 |
| m.6908T>C | 2 |
| m.6917G>A | 4 |
| m.6932A>G | 1 |
| m.6956T>C | 2 |
| m.6960C>T | 19 |
| m.6962G>A | 89 |
| m.6965T>C | 1 |
| m.7001A>G | 3 |
| m.7004A>G | 1 |
| m.7028C>T | 856 |
| m.7051T>C | 1 |
| m.7051T>G | 1 |
| m.7052A>G | 1 |
| m.7055A>G | 2 |
| m.7076A>G | 3 |
| m.7080T>C | 3 |
| m.7082C>T | 1 |
| m.7091A>G | 1 |
| (Continue) **Supplementary Table 3**. The distribution of mitochondrial DNA mutations per 856 untreated PLWH. | |
| Distinctive types of nucleotide variants | Count |
| m.7103C>T | 3 |
| m.7104T>A | 1 |
| m.7119G>A | 1 |
| m.7130C>T | 1 |
| m.7142T>C | 4 |
| m.7158A>G | 3 |
| m.7170C>T | 3 |
| m.7175T>C | 1 |
| m.7181C>T | 2 |
| m.7196C>A | 88 |
| m.7202A>G | 2 |
| m.7217A>G | 1 |
| m.7220T>C | 2 |
| m.7223C>T | 2 |
| m.7250A>C | 1 |
| m.7250A>G | 13 |
| m.7256C>T | 1 |
| m.7257A>G | 2 |
| m.7258T>C | 2 |
| m.7269G>A | 2 |
| m.7270T>C | 3 |
| m.7271A>G | 1 |
| m.7278T>C | 1 |
| m.7295A>G | 1 |
| m.7298A>G | 1 |
| m.7299A>G | 1 |
| m.7313C>T | 4 |
| m.7316G>C | 1 |
| m.7319T>C | 1 |
| m.7324A>T | 2 |
| m.7325A>T | 2 |
| m.7325A>G | 3 |
| m.7327C>G | 1 |
| m.7336C>Y | 22 |
| m.7336C>T | 1 |
| m.7337G>A | 3 |
| m.7340G>A | 2 |
| m.7346A>G | 1 |
| m.7364A>G | 3 |
| m.7372T>C | 1 |
| m.7376C>T | 2 |
| (Continue) **Supplementary Table 3**. The distribution of mitochondrial DNA mutations per 856 untreated PLWH. | |
| Distinctive types of nucleotide variants | Count |
| m.7378T>A | 1 |
| m.7402C>G | 1 |
| m.7403A>G | 3 |
| m.7419G>A | 2 |
| m.7424A>G | 4 |
| m.7428G>R | 1 |
| m.7433C>T | 2 |
| m.7439A>G | 1 |
| m.7444G>A | 10 |
| m.7492C>T | 1 |
| m.7493C>T | 1 |
| m.7501T>C | 1 |
| m.7521G>A | 5 |
| m.7533C>T | 1 |
| m.7551A>G | 1 |
| m.7561T>C | 1 |
| m.7572T>C | 1 |
| m.7581T>C | 2 |
| m.7598G>A | 9 |
| m.7600G>A | 23 |
| m.7604G>A | 1 |
| m.7609T>C | 2 |
| m.7621T>C | 2 |
| m.7630T>C | 1 |
| m.7642G>A | 5 |
| m.7667C>T | 1 |
| m.7673A>G | 3 |
| m.7675C>T | 1 |
| m.7678T>C | 2 |
| m.7679T>C | 1 |
| m.7684T>C | 59 |
| m.7691T>C | 1 |
| m.7692T>C | 2 |
| m.7696A>G | 1 |
| m.7701T>K | 1 |
| m.7705T>C | 1 |
| m.7706G>A | 1 |
| m.7711T>C | 1 |
| m.7738T>C | 7 |
| m.7741T>C | 2 |
| m.7754G>A | 3 |
| (Continue) **Supplementary Table 3**. The distribution of mitochondrial DNA mutations per 856 untreated PLWH. | |
| Distinctive types of nucleotide variants | Count |
| m.7762G>A | 2 |
| m.7765A>G | 6 |
| m.7774C>T | 2 |
| m.7783T>C | 2 |
| m.7789G>A | 4 |
| m.7804A>G | 1 |
| m.7807C>T | 1 |
| m.7822A>G | 4 |
| m.7825C>T | 1 |
| m.7828A>G | 33 |
| m.7830G>A | 1 |
| m.7837T>C | 1 |
| m.7849C>T | 1 |
| m.7852G>A | 2 |
| m.7853G>A | 81 |
| m.7859G>A | 1 |
| m.7861T>C | 10 |
| m.7864C>T | 1 |
| m.7867C>T | 6 |
| m.7888C>T | 1 |
| m.7897G>A | 1 |
| m.7912G>A | 1 |
| m.7924C>T | 1 |
| m.7933A>G | 2 |
| m.7934A>G | 2 |
| m.7941A>R | 1 |
| m.7954T>C | 1 |
| m.7957C>T | 1 |
| m.7958C>T | 1 |
| m.7960A>G | 1 |
| m.7961T>C | 2 |
| m.7964T>C | 1 |
| m.7975A>G | 1 |
| m.7978C>T | 1 |
| m.7979G>A | 1 |
| m.7987A>G | 1 |
| m.7993T>C | 1 |
| m.7999T>C | 8 |
| m.8020G>A | 29 |
| m.8027G>A | 2 |
| m.8038T>C | 1 |
| (Continue) **Supplementary Table 3**. The distribution of mitochondrial DNA mutations per 856 untreated PLWH. | |
| Distinctive types of nucleotide variants | Count |
| m.8041A>G | 1 |
| m.8047T>C | 1 |
| m.8084A>G | 1 |
| m.8087T>C | 2 |
| m.8104T>C | 3 |
| m.8107A>G | 2 |
| m.8108A>G | 5 |
| m.8110T>C | 8 |
| m.8119T>C | 2 |
| m.8130C>T | 1 |
| m.8137C>T | 1 |
| m.8149A>G | 11 |
| m.8155G>A | 2 |
| m.8158A>G | 2 |
| m.8167T>C | 1 |
| m.8179A>G | 2 |
| m.8188A>G | 2 |
| m.8195C>Y | 2 |
| m.8200T>C | 10 |
| m.8206G>A | 2 |
| m.8209C>T | 1 |
| m.8215C>T | 1 |
| m.8227T>C | 8 |
| m.8230C>T | 1 |
| m.8245A>G | 2 |
| m.8251G>A | 6 |
| m.8251G>R | 1 |
| m.8254C>T | 1 |
| m.8257A>G | 2 |
| m.8264C>T | 1 |
| m.8268A>C | 1 |
| m.8269G>C | 1 |
| m.8269G>A | 5 |
| m.8271A>C | 2 |
| m.8273C>A | 1 |
| m.8274C>T | 1 |
| m.8277T>C | 7 |
| m.8279T>C | 5 |
| m.8280A>C | 1 |
| m.8281C>G | 9 |
| m.8282C>A | 1 |
| (Continue) **Supplementary Table 3**. The distribution of mitochondrial DNA mutations per 856 untreated PLWH. | |
| Distinctive types of nucleotide variants | Count |
| m.8284C>A | 2 |
| m.8292G>A | 2 |
| m.8293C>T | 1 |
| m.8296A>G | 1 |
| m.8315A>G | 1 |
| m.8334G>A | 1 |
| m.8343A>G | 7 |
| m.8345C>T | 1 |
| m.8348A>G | 1 |
| m.8361G>A | 1 |
| m.8371C>T | 1 |
| m.8375C>T | 1 |
| m.8383T>C | 1 |
| m.8389A>G | 3 |
| m.8392G>A | 6 |
| m.8393C>T | 1 |
| m.8400T>C | 1 |
| m.8404T>C | 1 |
| m.8405A>G | 1 |
| m.8406C>T | 2 |
| m.8407C>A | 1 |
| m.8410C>T | 2 |
| m.8412T>C | 1 |
| m.8414C>T | 132 |
| m.8433T>C | 2 |
| m.8437C>A | 1 |
| m.8440A>G | 6 |
| m.8441C>T | 1 |
| m.8442T>C | 1 |
| m.8448T>C | 1 |
| m.8453A>G | 1 |
| m.8459A>G | 9 |
| m.8461C>T | 1 |
| m.8473T>C | 19 |
| m.8479A>G | 1 |
| m.8485G>A | 2 |
| m.8490T>C | 2 |
| m.8506T>C | 2 |
| m.8508A>G | 1 |
| m.8512A>G | 2 |
| m.8515C>T | 1 |
| (Continue) **Supplementary Table 3**. The distribution of mitochondrial DNA mutations per 856 untreated PLWH. | |
| Distinctive types of nucleotide variants | Count |
| m.8521A>G | 5 |
| m.8530A>G | 1 |
| m.8531A>G | 1 |
| m.8542T>C | 2 |
| m.8553C>T | 1 |
| m.8557G>A | 1 |
| m.8563A>G | 12 |
| m.8567T>C | 3 |
| m.8568C>T | 1 |
| m.8572G>A | 2 |
| m.8573G>A | 1 |
| m.8575C>T | 2 |
| m.8577A>G | 1 |
| m.8584G>A | 136 |
| m.8586A>G | 1 |
| m.8595C>T | 1 |
| m.8597T>C | 1 |
| m.8599C>T | 1 |
| m.8602T>C | 2 |
| m.8603T>C | 3 |
| m.8605C>T | 1 |
| m.8609C>T | 3 |
| m.8613A>G | 1 |
| m.8614T>C | 3 |
| m.8618T>C | 2 |
| m.8623A>G | 1 |
| m.8639T>C | 1 |
| m.8654T>C | 1 |
| m.8659A>G | 1 |
| m.8675T>C | 1 |
| m.8684C>T | 29 |
| m.8688A>T | 1 |
| m.8696T>C | 1 |
| m.8697G>A | 7 |
| m.8701A>G | 442 |
| m.8705T>C | 1 |
| m.8711A>G | 2 |
| m.8718A>G | 3 |
| m.8721A>G | 1 |
| m.8724A>G | 1 |
| m.8727C>T | 1 |
| (Continue) **Supplementary Table 3**. The distribution of mitochondrial DNA mutations per 856 untreated PLWH. | |
| Distinctive types of nucleotide variants | Count |
| m.8730A>G | 2 |
| m.8743G>R | 1 |
| m.8745A>G | 1 |
| m.8748C>T | 1 |
| m.8764G>A | 6 |
| m.8765C>T | 1 |
| m.8767A>T | 1 |
| m.8772T>C | 8 |
| m.8784A>G | 12 |
| m.8790G>A | 1 |
| m.8793T>C | 16 |
| m.8794C>T | 63 |
| m.8802T>C | 3 |
| m.8812A>T | 2 |
| m.8821T>G | 1 |
| m.8823T>C | 3 |
| m.8829C>T | 19 |
| m.8838G>A | 1 |
| m.8839G>A | 2 |
| m.8843T>C | 1 |
| m.8848T>C | 2 |
| m.8853A>G | 2 |
| m.8854G>A | 1 |
| m.8856G>A | 13 |
| m.8860A>G | 856 |
| m.8865G>A | 1 |
| m.8876T>C | 1 |
| m.8877T>C | 3 |
| m.8884A>G | 1 |
| m.8887A>G | 1 |
| m.8895T>C | 2 |
| m.8901A>G | 1 |
| m.8906A>G | 1 |
| m.8913A>G | 1 |
| m.8931A>G | 1 |
| m.8945T>C | 4 |
| m.8947C>T | 1 |
| m.8952T>C | 1 |
| m.8961A>G | 1 |
| m.8964C>T | 22 |
| m.8965A>G | 1 |
| (Continue) **Supplementary Table 3**. The distribution of mitochondrial DNA mutations per 856 untreated PLWH. | |
| Distinctive types of nucleotide variants | Count |
| m.8979T>C | 1 |
| m.8981A>G | 1 |
| m.8998G>A | 5 |
| m.9004C>T | 2 |
| m.9006A>G | 1 |
| m.9010G>A | 1 |
| m.9016A>G | 1 |
| m.9021T>C | 2 |
| m.9033A>G | 1 |
| m.9038T>C | 2 |
| m.9046A>G | 1 |
| m.9047T>W | 1 |
| m.9053G>A | 53 |
| m.9055G>A | 5 |
| m.9064G>R | 1 |
| m.9071C>T | 2 |
| m.9078T>C | 3 |
| m.9080A>G | 1 |
| m.9081C>T | 1 |
| m.9084T>C | 2 |
| m.9088T>Y | 1 |
| m.9088T>C | 2 |
| m.9090T>C | 22 |
| m.9098T>C | 1 |
| m.9099C>T | 2 |
| m.9100A>G | 1 |
| m.9101T>G | 3 |
| m.9104T>C | 2 |
| m.9106A>G | 1 |
| m.9112C>T | 1 |
| m.9115A>G | 1 |
| m.9117T>C | 1 |
| m.9119T>G | 1 |
| m.9123G>A | 22 |
| m.9126T>C | 5 |
| m.9127A>G | 2 |
| m.9128T>C | 9 |
| m.9135A>G | 1 |
| m.9139G>A | 2 |
| m.9141T>C | 1 |
| m.9144C>T | 1 |
| (Continue) **Supplementary Table 3**. The distribution of mitochondrial DNA mutations per 856 untreated PLWH. | |
| Distinctive types of nucleotide variants | Count |
| m.9152T>C | 1 |
| m.9162C>T | 1 |
| m.9163G>A | 1 |
| m.9174T>C | 1 |
| m.9179T>C | 1 |
| m.9180A>G | 49 |
| m.9181A>G | 2 |
| m.9186C>T | 1 |
| m.9189C>T | 4 |
| m.9191T>C | 1 |
| m.9236A>G | 2 |
| m.9242A>G | 5 |
| m.9254A>G | 2 |
| m.9266G>A | 2 |
| m.9266G>R | 1 |
| m.9296C>T | 19 |
| m.9300G>A | 1 |
| m.9305G>R | 1 |
| m.9315T>C | 1 |
| m.9316T>C | 1 |
| m.9317C>T | 4 |
| m.9329G>A | 1 |
| m.9332C>T | 1 |
| m.9335C>T | 2 |
| m.9336A>G | 1 |
| m.9337T>C | 1 |
| m.9337T>Y | 1 |
| m.9338A>T | 1 |
| m.9344C>T | 1 |
| m.9351A>R | 1 |
| m.9355A>G | 1 |
| m.9357A>G | 1 |
| m.9368A>G | 1 |
| m.9371C>T | 1 |
| m.9377A>G | 25 |
| m.9389A>G | 5 |
| m.9419C>T | 1 |
| m.9423C>T | 1 |
| m.9425A>G | 6 |
| m.9428T>C | 1 |
| m.9431C>T | 1 |
| (Continue) **Supplementary Table 3**. The distribution of mitochondrial DNA mutations per 856 untreated PLWH. | |
| Distinctive types of nucleotide variants | Count |
| m.9438G>A | 1 |
| m.9446A>G | 2 |
| m.9448A>G | 1 |
| m.9449C>T | 3 |
| m.9452G>A | 2 |
| m.9455A>G | 1 |
| m.9459C>T | 1 |
| m.9461A>G | 1 |
| m.9464T>Y | 1 |
| m.9468A>G | 1 |
| m.9477G>A | 1 |
| m.9494A>G | 2 |
| m.9495T>C | 2 |
| m.9500C>Y | 1 |
| m.9512C>T | 2 |
| m.9530T>C | 2 |
| m.9531A>G | 5 |
| m.9536C>T | 11 |
| m.9536C>A | 1 |
| m.9539A>C | 1 |
| m.9540T>C | 442 |
| m.9545A>G | 37 |
| m.9547G>C | 1 |
| m.9548G>A | 33 |
| m.9554G>A | 6 |
| m.9557C>A | 1 |
| m.9575G>A | 24 |
| m.9591G>A | 1 |
| m.9604A>G | 1 |
| m.9612G>C | 1 |
| m.9614A>G | 1 |
| m.9615T>C | 3 |
| m.9617A>G | 1 |
| m.9638C>A | 1 |
| m.9641C>T | 1 |
| m.9647T>C | 3 |
| m.9650C>T | 3 |
| m.9655G>A | 1 |
| m.9667A>G | 1 |
| m.9670A>G | 1 |
| m.9695G>K | 1 |
| (Continue) **Supplementary Table 3**. The distribution of mitochondrial DNA mutations per 856 untreated PLWH. | |
| Distinctive types of nucleotide variants | Count |
| m.9713G>A | 1 |
| m.9716T>C | 1 |
| m.9719C>Y | 1 |
| m.9722T>C | 1 |
| m.9725T>C | 1 |
| m.9732C>T | 2 |
| m.9738G>A | 1 |
| m.9740C>T | 1 |
| m.9758T>C | 4 |
| m.9764C>T | 1 |
| m.9767C>T | 1 |
| m.9773C>T | 3 |
| m.9773C>Y | 35 |
| m.9773C>S | 1 |
| m.9794A>G | 1 |
| m.9797T>C | 2 |
| m.9800T>C | 1 |
| m.9801G>A | 1 |
| m.9802T>G | 1 |
| m.9804G>A | 2 |
| m.9814T>C | 16 |
| m.9824T>C | 22 |
| m.9824T>A | 7 |
| m.9833T>C | 1 |
| m.9845T>C | 3 |
| m.9852A>T | 2 |
| m.9856T>C | 5 |
| m.9860C>T | 1 |
| m.9861T>C | 1 |
| m.9872A>G | 1 |
| m.9875A>G | 1 |
| m.9899T>C | 2 |
| m.9911C>A | 1 |
| m.9921G>A | 1 |
| m.9932G>A | 7 |
| m.9944T>C | 2 |
| m.9947G>A | 5 |
| m.9948G>A | 4 |
| m.9950T>C | 53 |
| m.9957T>C | 1 |
| m.9962G>A | 5 |
| (Continue) **Supplementary Table 3**. The distribution of mitochondrial DNA mutations per 856 untreated PLWH. | |
| Distinctive types of nucleotide variants | Count |
| m.9966G>A | 4 |
| m.9966G>R | 1 |
| m.9968C>T | 4 |
| m.9977T>C | 1 |
| m.9980A>G | 1 |
| m.9986G>R | 1 |
| m.9992C>T | 1 |
| m.9998T>A | 1 |
| m.10005A>G | 1 |
| m.10006A>G | 1 |
| m.10007T>C | 1 |
| m.10018A>G | 1 |
| m.10031T>C | 8 |
| m.10034T>C | 3 |
| m.10042A>G | 3 |
| m.10055A>G | 2 |
| m.10084T>C | 3 |
| m.10095C>T | 1 |
| m.10097A>G | 6 |
| m.10101T>C | 3 |
| m.10112A>G | 1 |
| m.10112A>T | 1 |
| m.10115T>C | 1 |
| m.10118T>C | 2 |
| m.10136A>G | 1 |
| m.10142C>T | 1 |
| m.10172G>A | 3 |
| m.10181C>T | 4 |
| m.10187T>C | 1 |
| m.10188A>G | 1 |
| m.10192C>T | 2 |
| m.10205C>A | 1 |
| m.10208T>C | 16 |
| m.10211C>T | 4 |
| m.10223C>T | 1 |
| m.10227T>C | 1 |
| m.10232A>G | 1 |
| m.10235T>C | 2 |
| m.10237T>C | 1 |
| m.10238T>C | 10 |
| m.10245T>C | 3 |
| (Continue) **Supplementary Table 3**. The distribution of mitochondrial DNA mutations per 856 untreated PLWH. | |
| Distinctive types of nucleotide variants | Count |
| m.10250A>G | 1 |
| m.10256T>C | 1 |
| m.10262A>G | 1 |
| m.10268C>T | 2 |
| m.10274T>C | 2 |
| m.10283A>G | 1 |
| m.10286A>G | 1 |
| m.10289A>G | 2 |
| m.10304T>C | 2 |
| m.10310G>A | 139 |
| m.10319A>G | 1 |
| m.10320G>A | 6 |
| m.10325G>A | 11 |
| m.10343C>T | 1 |
| m.10345T>C | 2 |
| m.10355C>T | 1 |
| m.10364G>A | 1 |
| m.10373G>A | 1 |
| m.10383A>T | 1 |
| m.10389T>C | 2 |
| m.10397A>G | 57 |
| m.10398A>G | 505 |
| m.10399C>A | 1 |
| m.10399C>G | 54 |
| m.10400C>T | 434 |
| m.10401G>T | 1 |
| m.10403A>G | 1 |
| m.10410T>C | 6 |
| m.10427G>A | 1 |
| m.10446A>G | 1 |
| m.10454T>C | 10 |
| m.10463T>C | 5 |
| m.10490T>C | 2 |
| m.10493T>C | 1 |
| m.10497C>T | 1 |
| m.10523A>G | 6 |
| m.10527C>T | 1 |
| m.10529A>G | 7 |
| m.10535T>C | 26 |
| m.10586G>A | 34 |
| m.10587C>T | 1 |
| (Continue) **Supplementary Table 3**. The distribution of mitochondrial DNA mutations per 856 untreated PLWH. | |
| Distinctive types of nucleotide variants | Count |
| m.10589G>A | 3 |
| m.10595T>C | 1 |
| m.10596A>G | 1 |
| m.10601T>G | 1 |
| m.10604T>C | 5 |
| m.10609T>C | 88 |
| m.10610A>G | 1 |
| m.10634A>G | 1 |
| m.10640T>C | 2 |
| m.10646G>A | 15 |
| m.10646G>R | 1 |
| m.10679A>G | 2 |
| m.10682A>G | 1 |
| m.10685G>A | 6 |
| m.10724T>C | 3 |
| m.10732A>R | 1 |
| m.10736C>T | 2 |
| m.10742T>C | 1 |
| m.10742T>G | 1 |
| m.10750A>G | 3 |
| m.10754A>G | 1 |
| m.10790T>C | 2 |
| m.10801G>A | 2 |
| m.10804A>C | 1 |
| m.10807A>G | 1 |
| m.10810T>C | 9 |
| m.10828T>C | 1 |
| m.10843C>T | 1 |
| m.10845C>T | 1 |
| m.10861T>C | 1 |
| m.10873T>C | 443 |
| m.10876A>G | 1 |
| m.10891A>G | 5 |
| m.10907T>C | 1 |
| m.10908T>C | 1 |
| m.10915T>C | 5 |
| m.10922A>G | 1 |
| m.10927T>C | 1 |
| m.10956T>G | 1 |
| m.10972A>G | 3 |
| m.10975C>T | 1 |
| (Continue) **Supplementary Table 3**. The distribution of mitochondrial DNA mutations per 856 untreated PLWH. | |
| Distinctive types of nucleotide variants | Count |
| m.10976C>T | 7 |
| m.10978A>G | 5 |
| m.10990C>T | 2 |
| m.11002A>G | 5 |
| m.11015A>C | 1 |
| m.11016G>A | 2 |
| m.11020A>G | 2 |
| m.11023A>C | 2 |
| m.11023A>G | 1 |
| m.11059C>T | 7 |
| m.11061C>T | 8 |
| m.11065A>G | 5 |
| m.11084A>G | 5 |
| m.11087T>C | 3 |
| m.11092A>G | 2 |
| m.11101A>G | 1 |
| m.11116T>C | 1 |
| m.11137T>C | 1 |
| m.11147T>C | 1 |
| m.11149G>A | 5 |
| m.11150G>A | 5 |
| m.11151C>T | 8 |
| m.11167A>G | 2 |
| m.11172A>G | 1 |
| m.11176G>A | 7 |
| m.11194A>G | 2 |
| m.11203C>T | 1 |
| m.11204T>C | 2 |
| m.11207C>T | 1 |
| m.11215C>T | 12 |
| m.11239A>G | 4 |
| m.11251A>G | 1 |
| m.11253T>C | 1 |
| m.11254T>C | 3 |
| m.11255T>C | 2 |
| m.11257C>T | 3 |
| m.11260T>C | 1 |
| m.11287T>C | 2 |
| m.11296C>T | 1 |
| m.11299T>C | 2 |
| m.11308C>T | 1 |
| (Continue) **Supplementary Table 3**. The distribution of mitochondrial DNA mutations per 856 untreated PLWH. | |
| Distinctive types of nucleotide variants | Count |
| m.11323C>T | 2 |
| m.11335C>T | 1 |
| m.11339T>C | 2 |
| m.11347A>G | 1 |
| m.11353T>C | 3 |
| m.11365T>C | 3 |
| m.11371A>G | 1 |
| m.11377G>A | 2 |
| m.11380A>G | 7 |
| m.11383T>C | 2 |
| m.11386T>C | 1 |
| m.11389C>T | 1 |
| m.11392A>C | 1 |
| m.11407C>T | 2 |
| m.11410T>C | 4 |
| m.11422C>T | 1 |
| m.11431C>T | 1 |
| m.11432A>G | 1 |
| m.11437T>C | 1 |
| m.11440G>A | 9 |
| m.11453G>A | 1 |
| m.11465T>C | 2 |
| m.11476C>T | 1 |
| m.11491A>G | 1 |
| m.11503C>T | 1 |
| m.11506T>C | 1 |
| m.11536C>T | 11 |
| m.11557A>G | 1 |
| m.11560A>G | 1 |
| m.11566A>G | 1 |
| m.11569T>C | 1 |
| m.11575A>G | 1 |
| m.11581C>A | 1 |
| m.11590A>G | 1 |
| m.11609T>G | 1 |
| m.11617T>C | 1 |
| m.11623C>T | 2 |
| m.11626T>C | 1 |
| m.11635C>T | 1 |
| m.11642G>T | 1 |
| m.11647C>T | 2 |
| (Continue) **Supplementary Table 3**. The distribution of mitochondrial DNA mutations per 856 untreated PLWH. | |
| Distinctive types of nucleotide variants | Count |
| m.11654A>G | 1 |
| m.11665C>T | 27 |
| m.11674C>A | 1 |
| m.11696G>A | 22 |
| m.11713C>T | 1 |
| m.11716C>T | 2 |
| m.11719G>A | 856 |
| m.11722T>C | 1 |
| m.11732T>C | 3 |
| m.11776T>C | 2 |
| m.11782C>T | 1 |
| m.11800A>G | 2 |
| m.11809T>C | 2 |
| m.11810C>T | 1 |
| m.11815C>A | 2 |
| m.11824A>G | 2 |
| m.11854T>C | 1 |
| m.11864T>C | 1 |
| m.11878T>C | 1 |
| m.11881C>T | 1 |
| m.11887G>A | 1 |
| m.11893A>G | 2 |
| m.11902G>A | 1 |
| m.11914G>A | 61 |
| m.11923A>G | 1 |
| m.11928A>G | 3 |
| m.11929T>C | 2 |
| m.11944T>C | 28 |
| m.11950A>G | 1 |
| m.11969G>A | 14 |
| m.11974A>G | 2 |
| m.11992T>C | 2 |
| m.11998A>C | 1 |
| m.12007G>A | 14 |
| m.12026A>G | 28 |
| m.12033A>G | 1 |
| m.12082A>G | 1 |
| m.12091T>C | 27 |
| m.12092C>A | 1 |
| m.12121T>C | 1 |
| m.12130T>C | 3 |
| (Continue) **Supplementary Table 3**. The distribution of mitochondrial DNA mutations per 856 untreated PLWH. | |
| Distinctive types of nucleotide variants | Count |
| m.12134T>C | 2 |
| m.12141A>G | 2 |
| m.12153C>T | 2 |
| m.12172A>G | 2 |
| m.12173T>G | 1 |
| m.12173T>C | 1 |
| m.12179A>G | 1 |
| m.12189T>C | 2 |
| m.12192G>A | 3 |
| m.12196C>T | 1 |
| m.12216C>T | 1 |
| m.12223A>G | 1 |
| m.12224C>T | 1 |
| m.12234A>G | 4 |
| m.12235T>C | 1 |
| m.12236G>A | 2 |
| m.12237C>T | 1 |
| m.12250C>T | 2 |
| m.12279A>G | 4 |
| m.12280A>G | 5 |
| m.12285T>A | 1 |
| m.12311T>C | 4 |
| m.12338T>C | 33 |
| m.12346C>T | 1 |
| m.12351T>C | 2 |
| m.12354T>C | 8 |
| m.12358A>G | 45 |
| m.12360C>T | 1 |
| m.12361A>G | 20 |
| m.12363C>T | 2 |
| m.12366A>G | 2 |
| m.12372G>A | 43 |
| m.12373A>G | 1 |
| m.12384T>C | 2 |
| m.12396T>C | 1 |
| m.12397A>G | 1 |
| m.12402C>T | 1 |
| m.12405C>T | 57 |
| m.12406G>A | 91 |
| m.12411C>T | 2 |
| m.12414T>C | 2 |
| (Continue) **Supplementary Table 3**. The distribution of mitochondrial DNA mutations per 856 untreated PLWH. | |
| Distinctive types of nucleotide variants | Count |
| m.12425A>G | 3 |
| m.12429A>G | 1 |
| m.12432C>T | 1 |
| m.12438T>C | 1 |
| m.12468T>C | 1 |
| m.12477T>C | 1 |
| m.12495A>G | 1 |
| m.12501G>A | 6 |
| m.12507A>C | 1 |
| m.12528G>A | 1 |
| m.12534A>G | 5 |
| m.12545C>T | 1 |
| m.12549C>T | 13 |
| m.12561G>A | 7 |
| m.12609T>C | 1 |
| m.12612A>G | 6 |
| m.12618G>A | 4 |
| m.12621C>T | 4 |
| m.12622G>A | 1 |
| m.12630G>A | 10 |
| m.12633C>T | 5 |
| m.12633C>A | 1 |
| m.12634A>G | 3 |
| m.12642A>G | 1 |
| m.12654A>G | 5 |
| m.12666A>C | 5 |
| m.12672A>G | 8 |
| m.12684G>A | 1 |
| m.12696T>G | 1 |
| m.12699A>G | 1 |
| m.12705C>T | 552 |
| m.12709C>T | 3 |
| m.12714T>C | 8 |
| m.12715A>G | 1 |
| m.12717C>T | 2 |
| m.12720A>G | 1 |
| m.12727T>C | 1 |
| m.12732T>C | 4 |
| m.12747A>G | 3 |
| m.12771G>A | 11 |
| m.12772G>R | 1 |
| (Continue) **Supplementary Table 3**. The distribution of mitochondrial DNA mutations per 856 untreated PLWH. | |
| Distinctive types of nucleotide variants | Count |
| m.12774C>T | 1 |
| m.12793T>C | 1 |
| m.12801C>T | 2 |
| m.12809G>C | 1 |
| m.12810A>G | 4 |
| m.12811T>C | 55 |
| m.12819A>G | 1 |
| m.12825T>C | 1 |
| m.12843T>C | 1 |
| m.12850A>G | 2 |
| m.12864T>C | 2 |
| m.12879T>C | 3 |
| m.12882C>T | 89 |
| m.12892T>C | 2 |
| m.12903T>C | 1 |
| m.12904A>G | 2 |
| m.12906C>A | 4 |
| m.12909A>G | 2 |
| m.12924A>G | 1 |
| m.12939A>G | 1 |
| m.12940G>A | 1 |
| m.12945T>C | 1 |
| m.12950A>G | 10 |
| m.12952G>A | 1 |
| m.12954T>C | 1 |
| m.12957T>C | 13 |
| m.12975A>G | 1 |
| m.12988C>T | 2 |
| m.13001G>C | 1 |
| m.13020T>C | 1 |
| m.13036C>T | 1 |
| m.13044C>T | 2 |
| m.13050A>G | 2 |
| m.13074A>G | 5 |
| m.13090A>C | 2 |
| m.13098A>T | 1 |
| m.13098A>G | 1 |
| m.13104A>G | 18 |
| m.13105A>G | 19 |
| m.13111T>C | 1 |
| m.13122C>T | 1 |
| (Continue) **Supplementary Table 3**. The distribution of mitochondrial DNA mutations per 856 untreated PLWH. | |
| Distinctive types of nucleotide variants | Count |
| m.13131C>T | 2 |
| m.13135G>A | 19 |
| m.13145G>A | 4 |
| m.13146C>T | 1 |
| m.13149A>G | 2 |
| m.13152A>G | 13 |
| m.13153A>G | 1 |
| m.13158A>G | 1 |
| m.13167A>G | 1 |
| m.13182T>C | 1 |
| m.13186A>T | 1 |
| m.13191T>C | 1 |
| m.13192C>T | 2 |
| m.13194G>A | 3 |
| m.13203A>G | 1 |
| m.13215T>C | 3 |
| m.13260T>C | 4 |
| m.13263A>G | 37 |
| m.13269A>G | 11 |
| m.13275A>G | 1 |
| m.13281T>C | 2 |
| m.13287C>T | 3 |
| m.13290C>T | 1 |
| m.13299A>G | 1 |
| m.13329C>T | 1 |
| m.13368G>A | 1 |
| m.13374C>T | 1 |
| m.13383C>T | 2 |
| m.13386T>C | 1 |
| m.13388A>G | 1 |
| m.13395A>G | 3 |
| m.13401T>C | 1 |
| m.13422A>G | 4 |
| m.13434A>G | 8 |
| m.13477G>A | 3 |
| m.13488T>C | 3 |
| m.13500T>C | 1 |
| m.13512A>G | 2 |
| m.13528A>G | 1 |
| m.13547C>T | 1 |
| m.13563A>G | 26 |
| (Continue) **Supplementary Table 3**. The distribution of mitochondrial DNA mutations per 856 untreated PLWH. | |
| Distinctive types of nucleotide variants | Count |
| m.13563A>R | 1 |
| m.13590G>A | 30 |
| m.13593A>G | 1 |
| m.13602T>C | 7 |
| m.13617T>C | 1 |
| m.13618C>A | 1 |
| m.13620T>C | 1 |
| m.13626C>T | 1 |
| m.13629A>G | 5 |
| m.13635T>C | 1 |
| m.13649C>A | 1 |
| m.13650C>T | 2 |
| m.13651A>G | 2 |
| m.13656T>C | 1 |
| m.13659T>Y | 1 |
| m.13662C>T | 2 |
| m.13681A>G | 12 |
| m.13707G>A | 4 |
| m.13708G>A | 42 |
| m.13711G>R | 1 |
| m.13712C>A | 1 |
| m.13713C>T | 1 |
| m.13720C>T | 1 |
| m.13722A>G | 6 |
| m.13726G>S | 1 |
| m.13731A>G | 1 |
| m.13740T>C | 1 |
| m.13743T>C | 2 |
| m.13748A>G | 2 |
| m.13749C>T | 1 |
| m.13754C>A | 1 |
| m.13754C>Y | 1 |
| m.13759G>A | 61 |
| m.13767C>T | 1 |
| m.13768T>C | 2 |
| m.13780A>G | 2 |
| m.13782C>T | 2 |
| m.13788C>T | 2 |
| m.13812T>C | 1 |
| m.13819T>C | 1 |
| m.13821C>T | 1 |
| (Continue) **Supplementary Table 3**. The distribution of mitochondrial DNA mutations per 856 untreated PLWH. | |
| Distinctive types of nucleotide variants | Count |
| m.13824A>G | 1 |
| m.13827A>G | 3 |
| m.13830T>C | 3 |
| m.13834A>G | 1 |
| m.13842A>G | 1 |
| m.13857A>G | 1 |
| m.13858A>G | 2 |
| m.13866A>G | 1 |
| m.13879T>C | 3 |
| m.13890C>T | 3 |
| m.13896T>C | 2 |
| m.13907A>G | 2 |
| m.13914C>A | 2 |
| m.13926T>C | 1 |
| m.13927A>T | 1 |
| m.13928G>C | 142 |
| m.13928G>R | 1 |
| m.13928G>T | 1 |
| m.13933A>G | 1 |
| m.13934C>T | 3 |
| m.13937A>T | 1 |
| m.13942A>G | 7 |
| m.13950C>T | 1 |
| m.13953T>C | 1 |
| m.13954C>T | 1 |
| m.13966A>M | 1 |
| m.13968G>A | 3 |
| m.13974A>G | 1 |
| m.13980G>A | 3 |
| m.13983C>T | 1 |
| m.13986A>T | 1 |
| m.13989C>T | 2 |
| m.13995A>G | 1 |
| m.13999C>T | 1 |
| m.14002A>G | 1 |
| m.14005T>Y | 2 |
| m.14007A>G | 2 |
| m.14016G>A | 3 |
| m.14020T>C | 2 |
| m.14028A>G | 1 |
| m.14034T>C | 1 |
| (Continue) **Supplementary Table 3**. The distribution of mitochondrial DNA mutations per 856 untreated PLWH. | |
| Distinctive types of nucleotide variants | Count |
| m.14040G>A | 3 |
| m.14047A>G | 1 |
| m.14049C>T | 2 |
| m.14053A>G | 8 |
| m.14063T>C | 2 |
| m.14064C>T | 2 |
| m.14067C>T | 9 |
| m.14071A>G | 1 |
| m.14073C>T | 1 |
| m.14088T>C | 2 |
| m.14097C>T | 1 |
| m.14100C>T | 1 |
| m.14110T>C | 7 |
| m.14112C>A | 1 |
| m.14115C>T | 1 |
| m.14124C>T | 1 |
| m.14129C>T | 2 |
| m.14131C>T | 1 |
| m.14133A>G | 4 |
| m.14146T>K | 1 |
| m.14148A>G | 5 |
| m.14149C>T | 4 |
| m.14155C>T | 2 |
| m.14167C>T | 1 |
| m.14175C>S | 1 |
| m.14178T>C | 6 |
| m.14180T>C | 4 |
| m.14182T>C | 1 |
| m.14198G>A | 3 |
| m.14199T>C | 1 |
| m.14200T>C | 16 |
| m.14203A>G | 1 |
| m.14209A>G | 2 |
| m.14212T>C | 1 |
| m.14218T>C | 3 |
| m.14221T>C | 2 |
| m.14224A>T | 1 |
| m.14227C>T | 1 |
| m.14239C>T | 1 |
| m.14257A>G | 1 |
| m.14258G>A | 1 |
| (Continue) **Supplementary Table 3**. The distribution of mitochondrial DNA mutations per 856 untreated PLWH. | |
| Distinctive types of nucleotide variants | Count |
| m.14259G>A | 2 |
| m.14263C>T | 1 |
| m.14278T>C | 1 |
| m.14280A>G | 3 |
| m.14302T>C | 3 |
| m.14305G>A | 3 |
| m.14308T>C | 15 |
| m.14311T>C | 4 |
| m.14314A>G | 2 |
| m.14317A>C | 1 |
| m.14318T>C | 37 |
| m.14319T>C | 4 |
| m.14323G>A | 1 |
| m.14325T>C | 3 |
| m.14326T>C | 1 |
| m.14328C>T | 1 |
| m.14329C>T | 1 |
| m.14334C>T | 3 |
| m.14335C>T | 1 |
| m.14340C>T | 3 |
| m.14348T>K | 1 |
| m.14356C>T | 1 |
| m.14364G>A | 4 |
| m.14370A>G | 1 |
| m.14371T>C | 1 |
| m.14384G>A | 4 |
| m.14392C>T | 1 |
| m.14410G>A | 5 |
| m.14410G>R | 1 |
| m.14417A>G | 5 |
| m.14418C>T | 1 |
| m.14419C>T | 1 |
| m.14420C>A | 1 |
| m.14425C>T | 1 |
| m.14431T>C | 1 |
| m.14436C>S | 1 |
| m.14470T>C | 34 |
| m.14476G>A | 7 |
| m.14485C>T | 3 |
| m.14494T>C | 1 |
| m.14502T>C | 14 |
| (Continue) **Supplementary Table 3**. The distribution of mitochondrial DNA mutations per 856 untreated PLWH. | |
| Distinctive types of nucleotide variants | Count |
| m.14518A>G | 3 |
| m.14553C>T | 1 |
| m.14554A>G | 1 |
| m.14560G>A | 13 |
| m.14560G>C | 1 |
| m.14569G>A | 39 |
| m.14572A>R | 3 |
| m.14572A>M | 1 |
| m.14581T>C | 3 |
| m.14587A>G | 11 |
| m.14588C>G | 1 |
| m.14590C>T | 1 |
| m.14605A>R | 1 |
| m.14605A>G | 3 |
| m.14617A>G | 1 |
| m.14620C>T | 1 |
| m.14632C>T | 3 |
| m.14650C>T | 1 |
| m.14659C>T | 1 |
| m.14668C>T | 134 |
| m.14669A>G | 2 |
| m.14687A>G | 3 |
| m.14692A>G | 1 |
| m.14693A>G | 6 |
| m.14696A>G | 3 |
| m.14751C>T | 7 |
| m.14752C>T | 1 |
| m.14755A>C | 1 |
| m.14755A>G | 3 |
| m.14757T>C | 1 |
| m.14763A>G | 2 |
| m.14766C>T | 853 |
| m.14782A>C | 1 |
| m.14783T>C | 441 |
| m.14790A>G | 1 |
| m.14815C>T | 1 |
| m.14815C>M | 1 |
| m.14831G>A | 1 |
| m.14839A>G | 1 |
| m.14854C>A | 1 |
| m.14857T>C | 6 |
| (Continue) **Supplementary Table 3**. The distribution of mitochondrial DNA mutations per 856 untreated PLWH. | |
| Distinctive types of nucleotide variants | Count |
| m.14861G>A | 8 |
| m.14869G>A | 1 |
| m.14871T>C | 1 |
| m.14875C>T | 1 |
| m.14880T>C | 1 |
| m.14883C>T | 1 |
| m.14890A>G | 1 |
| m.14896C>T | 1 |
| m.14903A>G | 1 |
| m.14905G>A | 4 |
| m.14927A>G | 6 |
| m.14968T>C | 2 |
| m.14971T>C | 6 |
| m.14974C>T | 2 |
| m.14978A>G | 20 |
| m.14979T>C | 25 |
| m.14989C>T | 1 |
| m.15001T>C | 1 |
| m.15022C>T | 1 |
| m.15024G>A | 10 |
| m.15034A>G | 1 |
| m.15038A>G | 3 |
| m.15040C>T | 13 |
| m.15041G>T | 3 |
| m.15043G>A | 443 |
| m.15047G>A | 2 |
| m.15071T>C | 13 |
| m.15077G>A | 2 |
| m.15077G>R | 1 |
| m.15079A>G | 1 |
| m.15080A>G | 1 |
| m.15094C>T | 1 |
| m.15095A>G | 2 |
| m.15104C>T | 1 |
| m.15106G>A | 3 |
| m.15109T>C | 5 |
| m.15110G>A | 3 |
| m.15113A>G | 1 |
| m.15114C>G | 1 |
| m.15115T>C | 1 |
| m.15119G>A | 4 |
| (Continue) **Supplementary Table 3**. The distribution of mitochondrial DNA mutations per 856 untreated PLWH. | |
| Distinctive types of nucleotide variants | Count |
| m.15139T>C | 2 |
| m.15142C>T | 2 |
| m.15143C>T | 1 |
| m.15148G>A | 1 |
| m.15151A>G | 1 |
| m.15172G>A | 3 |
| m.15175C>T | 1 |
| m.15178A>G | 1 |
| m.15184T>C | 1 |
| m.15203A>G | 1 |
| m.15204T>C | 9 |
| m.15217G>A | 5 |
| m.15218A>G | 15 |
| m.15221G>A | 3 |
| m.15223C>T | 18 |
| m.15226A>G | 2 |
| m.15229T>C | 3 |
| m.15232A>G | 1 |
| m.15235A>G | 28 |
| m.15236A>G | 17 |
| m.15244A>G | 7 |
| m.15257G>A | 3 |
| m.15258A>G | 1 |
| m.15263C>T | 1 |
| m.15268C>T | 1 |
| m.15289T>C | 4 |
| m.15295C>T | 2 |
| m.15299T>C | 2 |
| m.15301G>A | 445 |
| m.15313T>C | 3 |
| m.15314G>A | 3 |
| m.15317G>A | 2 |
| m.15322A>G | 2 |
| m.15323G>T | 1 |
| m.15323G>A | 10 |
| m.15325A>G | 1 |
| m.15326A>G | 846 |
| m.15343C>T | 1 |
| m.15346G>A | 28 |
| m.15355G>A | 2 |
| m.15361A>R | 1 |
| (Continue) **Supplementary Table 3**. The distribution of mitochondrial DNA mutations per 856 untreated PLWH. | |
| Distinctive types of nucleotide variants | Count |
| m.15363A>G | 1 |
| m.15370C>T | 1 |
| m.15388T>C | 1 |
| m.15395A>R | 1 |
| m.15402C>T | 6 |
| m.15403C>T | 1 |
| m.15412T>G | 2 |
| m.15412T>C | 1 |
| m.15430C>T | 1 |
| m.15434C>T | 1 |
| m.15436C>T | 4 |
| m.15440T>C | 5 |
| m.15445T>C | 1 |
| m.15450T>C | 1 |
| m.15452C>A | 1 |
| m.15458T>C | 2 |
| m.15460C>T | 4 |
| m.15466G>A | 2 |
| m.15470T>C | 4 |
| m.15475A>G | 6 |
| m.15479T>C | 6 |
| m.15481C>T | 1 |
| m.15487A>T | 88 |
| m.15496A>G | 1 |
| m.15497G>A | 8 |
| m.15505A>G | 1 |
| m.15508C>T | 18 |
| m.15510A>C | 1 |
| m.15511T>C | 3 |
| m.15514T>C | 2 |
| m.15518C>T | 2 |
| m.15529C>A | 4 |
| m.15529C>T | 1 |
| m.15530T>C | 1 |
| m.15535C>T | 34 |
| m.15535C>Y | 1 |
| m.15538C>T | 1 |
| m.15547C>T | 1 |
| m.15550C>T | 1 |
| m.15559A>G | 1 |
| m.15562A>G | 2 |
| (Continue) **Supplementary Table 3**. The distribution of mitochondrial DNA mutations per 856 untreated PLWH. | |
| Distinctive types of nucleotide variants | Count |
| m.15565T>C | 6 |
| m.15601T>C | 1 |
| m.15607A>G | 1 |
| m.15613A>G | 1 |
| m.15616C>T | 1 |
| m.15617G>A | 1 |
| m.15622T>C | 4 |
| m.15629T>C | 2 |
| m.15632C>A | 1 |
| m.15637C>T | 1 |
| m.15655A>G | 1 |
| m.15661C>T | 1 |
| m.15662A>G | 18 |
| m.15670T>C | 7 |
| m.15672T>C | 3 |
| m.15679A>G | 1 |
| m.15685A>G | 2 |
| m.15688C>T | 3 |
| m.15691A>G | 3 |
| m.15697T>C | 1 |
| m.15703A>G | 2 |
| m.15712A>G | 2 |
| m.15714C>T | 1 |
| m.15721T>C | 1 |
| m.15724A>G | 16 |
| m.15725C>T | 1 |
| m.15731G>A | 1 |
| m.15734G>A | 5 |
| m.15737G>A | 1 |
| m.15746A>G | 3 |
| m.15747T>C | 1 |
| m.15754C>T | 1 |
| m.15758A>G | 8 |
| m.15773G>A | 1 |
| m.15774T>C | 2 |
| m.15777G>A | 1 |
| m.15779T>C | 1 |
| m.15784T>C | 25 |
| m.15787T>C | 1 |
| m.15814A>G | 1 |
| m.15828C>T | 1 |
| (Continue) **Supplementary Table 3**. The distribution of mitochondrial DNA mutations per 856 untreated PLWH. | |
| Distinctive types of nucleotide variants | Count |
| m.15832C>T | 1 |
| m.15848A>C | 1 |
| m.15850T>C | 4 |
| m.15851A>G | 19 |
| m.15852T>C | 1 |
| m.15860A>G | 4 |
| m.15862T>C | 2 |
| m.15877C>T | 1 |
| m.15883G>A | 2 |
| m.15884G>A | 5 |
| m.15884G>C | 1 |
| m.15885C>T | 2 |
| m.15889T>C | 3 |
| m.15900T>C | 2 |
| m.15903A>G | 1 |
| m.15907A>G | 1 |
| m.15908T>C | 2 |
| m.15910C>T | 2 |
| m.15924A>G | 13 |
| m.15927G>A | 20 |
| m.15928G>A | 1 |
| m.15930G>A | 10 |
| m.15940T>C | 4 |
| m.15941T>C | 4 |
| m.15951A>G | 5 |
| m.15954A>G | 2 |
| m.15961G>A | 1 |
| m.15968T>C | 8 |
| m.15970T>C | 2 |
| m.15983T>C | 1 |
| m.15993A>R | 2 |
| m.16000G>T | 2 |
| m.16017T>C | 1 |
| m.16047G>A | 2 |
| m.16048G>A | 2 |
| m.16051A>G | 16 |
| m.16066A>G | 2 |
| m.16068T>C | 4 |
| m.16069C>T | 1 |
| m.16071C>T | 1 |
| m.16075T>C | 2 |
| (Continue) **Supplementary Table 3**. The distribution of mitochondrial DNA mutations per 856 untreated PLWH. | |
| Distinctive types of nucleotide variants | Count |
| m.16079C>T | 1 |
| m.16086T>C | 20 |
| m.16092T>C | 32 |
| m.16092T>A | 7 |
| m.16093T>C | 57 |
| m.16093T>Y | 2 |
| m.16095C>T | 3 |
| m.16108C>T | 8 |
| m.16111C>T | 32 |
| m.16111C>G | 1 |
| m.16111C>Y | 1 |
| m.16114C>A | 1 |
| m.16124T>C | 9 |
| m.16126T>C | 24 |
| m.16127A>G | 1 |
| m.16129G>A | 192 |
| m.16131T>C | 2 |
| m.16131T>G | 2 |
| m.16132A>G | 1 |
| m.16134C>T | 4 |
| m.16136T>C | 31 |
| m.16138A>T | 1 |
| m.16140T>C | 65 |
| m.16145G>A | 8 |
| m.16147C>T | 2 |
| m.16147C>G | 1 |
| m.16148C>T | 9 |
| m.16150C>T | 7 |
| m.16153G>A | 3 |
| m.16157T>C | 4 |
| m.16158A>G | 1 |
| m.16161T>A | 1 |
| m.16162A>G | 32 |
| m.16163A>G | 1 |
| m.16164A>G | 12 |
| m.16166A>C | 1 |
| m.16166A>G | 5 |
| m.16167C>T | 3 |
| m.16168C>T | 4 |
| m.16169C>T | 2 |
| m.16170A>T | 1 |
| (Continue) **Supplementary Table 3**. The distribution of mitochondrial DNA mutations per 856 untreated PLWH. | |
| Distinctive types of nucleotide variants | Count |
| m.16171A>G | 5 |
| m.16172T>C | 80 |
| m.16173C>T | 6 |
| m.16174C>T | 6 |
| m.16176C>T | 4 |
| m.16178T>C | 1 |
| m.16179C>T | 6 |
| m.16179C>A | 1 |
| m.16180A>C | 1 |
| m.16181A>C | 5 |
| m.16182A>C | 126 |
| m.16183A>C | 215 |
| m.16184C>T | 28 |
| m.16185C>T | 25 |
| m.16186C>Y | 1 |
| m.16186C>T | 2 |
| m.16187C>A | 1 |
| m.16187C>T | 6 |
| m.16188C>T | 4 |
| m.16189T>C | 376 |
| m.16189T>Y | 1 |
| m.16192C>T | 33 |
| m.16193C>T | 3 |
| m.16194A>C | 3 |
| m.16195T>C | 3 |
| m.16196G>A | 1 |
| m.16201C>T | 1 |
| m.16203A>G | 9 |
| m.16207A>G | 6 |
| m.16209T>C | 9 |
| m.16211C>T | 1 |
| m.16213G>A | 9 |
| m.16214C>T | 6 |
| m.16215A>G | 1 |
| m.16216A>C | 1 |
| m.16216A>G | 1 |
| m.16217T>C | 95 |
| m.16218C>T | 13 |
| m.16220A>C | 4 |
| m.16220A>G | 1 |
| m.16221C>T | 2 |
| (Continue) **Supplementary Table 3**. The distribution of mitochondrial DNA mutations per 856 untreated PLWH. | |
| Distinctive types of nucleotide variants | Count |
| m.16222C>T | 3 |
| m.16223C>T | 517 |
| m.16224T>C | 5 |
| m.16226A>C | 1 |
| m.16227A>G | 13 |
| m.16229T>C | 1 |
| m.16230A>C | 1 |
| m.16230A>G | 1 |
| m.16231T>C | 9 |
| m.16232C>A | 7 |
| m.16234C>T | 37 |
| m.16235A>G | 5 |
| m.16235A>R | 1 |
| m.16237A>C | 1 |
| m.16238T>C | 1 |
| m.16239C>T | 2 |
| m.16240A>G | 6 |
| m.16240A>C | 1 |
| m.16242C>T | 2 |
| m.16242C>A | 2 |
| m.16243T>C | 20 |
| m.16245C>T | 3 |
| m.16248C>T | 8 |
| m.16249T>A | 2 |
| m.16249T>C | 18 |
| m.16255G>A | 3 |
| m.16256C>T | 16 |
| m.16257C>A | 38 |
| m.16257C>T | 1 |
| m.16258A>C | 1 |
| m.16259C>A | 1 |
| m.16259C>T | 2 |
| m.16260C>T | 32 |
| m.16261C>T | 70 |
| m.16261C>A | 4 |
| m.16262C>T | 3 |
| m.16263T>C | 2 |
| m.16263T>A | 3 |
| m.16264C>T | 2 |
| m.16265A>C | 1 |
| m.16265A>T | 1 |
| (Continue) **Supplementary Table 3**. The distribution of mitochondrial DNA mutations per 856 untreated PLWH. | |
| Distinctive types of nucleotide variants | Count |
| m.16265A>G | 1 |
| m.16266C>A | 22 |
| m.16266C>T | 33 |
| m.16266C>G | 7 |
| m.16267C>T | 2 |
| m.16269A>G | 3 |
| m.16270C>T | 6 |
| m.16271T>C | 9 |
| m.16272A>G | 6 |
| m.16274G>A | 55 |
| m.16278C>T | 36 |
| m.16280A>G | 1 |
| m.16284A>G | 9 |
| m.16286C>T | 2 |
| m.16287C>T | 3 |
| m.16288T>C | 3 |
| m.16289A>G | 1 |
| m.16290C>T | 66 |
| m.16291C>T | 17 |
| m.16291C>A | 1 |
| m.16292C>T | 8 |
| m.16293A>C | 8 |
| m.16293A>G | 5 |
| m.16293A>T | 3 |
| m.16294C>A | 1 |
| m.16294C>T | 7 |
| m.16295C>A | 1 |
| m.16295C>T | 15 |
| m.16297T>C | 59 |
| m.16298T>C | 107 |
| m.16299A>G | 12 |
| m.16299A>C | 1 |
| m.16300A>G | 9 |
| m.16301C>T | 2 |
| m.16302A>G | 5 |
| m.16302A>C | 40 |
| m.16303G>A | 2 |
| m.16304T>C | 141 |
| m.16305A>T | 2 |
| m.16309A>G | 9 |
| m.16311T>C | 152 |
| (Continue) **Supplementary Table 3**. The distribution of mitochondrial DNA mutations per 856 untreated PLWH. | |
| Distinctive types of nucleotide variants | Count |
| m.16311T>Y | 1 |
| m.16316A>G | 14 |
| m.16318A>G | 1 |
| m.16319G>A | 111 |
| m.16320C>T | 6 |
| m.16324T>C | 2 |
| m.16325T>C | 11 |
| m.16327C>T | 39 |
| m.16335A>G | 22 |
| m.16342T>C | 1 |
| m.16343A>C | 2 |
| m.16344C>T | 2 |
| m.16352T>C | 1 |
| m.16354C>T | 1 |
| m.16355C>T | 10 |
| m.16356T>C | 3 |
| m.16357T>C | 9 |
| m.16360C>T | 9 |
| m.16361G>A | 1 |
| m.16362T>C | 247 |
| m.16365C>T | 1 |
| m.16368T>C | 2 |
| m.16374A>C | 1 |
| m.16380C>T | 4 |
| m.16381T>C | 3 |
| m.16390G>A | 24 |
| m.16391G>A | 2 |
| m.16398G>A | 2 |
| m.16399A>G | 16 |
| m.16400C>T | 2 |
| m.16443T>C | 1 |
| m.16445T>C | 1 |
| m.16463A>G | 7 |
| m.16463A>T | 1 |
| m.16465C>T | 1 |
| m.16468T>C | 3 |
| m.16470G>A | 5 |
| m.16471G>A | 6 |
| m.16473G>A | 5 |
| m.16474G>A | 1 |
| m.16497A>G | 4 |
| (Continue) **Supplementary Table 3**. The distribution of mitochondrial DNA mutations per 856 untreated PLWH. | |
| Distinctive types of nucleotide variants | Count |
| m.16519T>C | 473 |
| m.16519T>Y | 1 |
| m.16523A>G | 1 |
| m.16526G>A | 5 |
| m.16526G>T | 1 |
| m.16527C>T | 4 |
| m.16540C>T | 1 |
| m.16542C>T | 1 |
| m.42_43insC | 1 |
| m.42_43insG | 3 |
| m.66del | 1 |
| m.193_194insC | 1 |
| m.204del | 1 |
| m.248del | 164 |
| m.285_286insA | 1 |
| m.286del | 1 |
| m.302_303insC | 4 |
| m.302_303insCC | 6 |
| m.302_303insCCC | 6 |
| m.309_310insCT | 279 |
| m.309_310insCCT | 121 |
| m.309_310insCCCT | 24 |
| m.310_311insC | 212 |
| m.310_311insCC | 3 |
| m.310_311insTC | 1 |
| m.316del | 1 |
| m.352_353insC | 2 |
| m.404_405insT | 1 |
| m.451_452insWT | 1 |
| m.456del | 3 |
| m.513_514insCA | 10 |
| m.513_514insCACA | 1 |
| m.514_515del | 268 |
| m.514_517del | 1 |
| m.565_566insCCCCC | 1 |
| m.565_566insCCC | 3 |
| m.567_568insC | 3 |
| m.567_568insCC | 1 |
| m.567_568insCCC | 5 |
| m.567_568insCCCC | 5 |
| m.567_568insCCCCC | 5 |
| (Continue) **Supplementary Table 3**. The distribution of mitochondrial DNA mutations per 856 untreated PLWH. | |
| Distinctive types of nucleotide variants | Count |
| m.568del | 1 |
| m.593_594insC | 1 |
| m.956del | 1 |
| m.955_956insC | 18 |
| m.955_956insCC | 4 |
| m.955_956insCCCC | 1 |
| m.2150_2151insA | 1 |
| m.2226_2227insA | 10 |
| m.2226_2227insAA | 1 |
| m.2368_2369insA | 2 |
| m.2400_2401insA | 1 |
| m.2449_2450insA | 1 |
| m.3107del | 2 |
| m.3167_3168insC | 11 |
| m.3209_3210insC | 1 |
| m.3571_3572insY | 1 |
| m.3572_3573insC | 1 |
| m.4121_4122insA | 1 |
| m.4136_4137insC | 1 |
| m.4183del | 3 |
| m.4188_4189insC | 1 |
| m.4312_4313insT | 1 |
| m.4878_4879insC | 1 |
| m.5243_5244insG | 1 |
| m.5480_5481insC | 1 |
| m.5533_5534insC | 2 |
| m.5542_5543insT | 1 |
| m.5676_5677insC | 3 |
| m.5747del | 1 |
| m.5891_5892insTT | 1 |
| m.5892_5893insTTCC | 1 |
| m.5894_5895insC | 16 |
| m.5895del | 1 |
| m.6583_6584insC | 1 |
| m.6691_6692insA | 1 |
| m.7174_7175insT | 1 |
| m.7358_7359insG | 1 |
| m.7465_7466insC | 5 |
| m.7466del | 1 |
| m.7490_7491insC | 1 |
| m.7514_7515insA | 1 |
| (Continue) **Supplementary Table 3**. The distribution of mitochondrial DNA mutations per 856 untreated PLWH. | |
| Distinctive types of nucleotide variants | Count |
| m.7515del | 1 |
| m.7699_7700insG | 1 |
| m.8245_8246insATAGGGCCCGAT | 1 |
| m.8269_8270insCCC | 1 |
| ｍ.8271_8279del | 118 |
| m.8271_8272insCCCCCC | 1 |
| m.8271_8272insCC | 2 |
| m.8271_8272insCCC | 1 |
| m.8280_8281insGCAG | 1 |
| m.8280_8281insGAG | 1 |
| m.9044_9045insA | 1 |
| m.9202_9205del | 1 |
| m.9378_9379insG | 3 |
| m.9795del | 1 |
| m.9813del | 331 |
| m.9823del | 1 |
| m.10569_10570insA | 2 |
| m.10635_10636insC | 1 |
| m.11139_11140insC | 1 |
| m.11233_11234insC | 1 |
| m.11749del | 1 |
| m.12380_12381insA | 1 |
| m.12397_12398insC | 1 |
| m.13753_13754insC | 2 |
| m.13762_13763insC | 1 |
| m.13781_13782insC | 2 |
| m.14153del | 1 |
| m.14572del | 1 |
| m.14769_14770insC | 1 |
| m.14808_14809insC | 1 |
| m.14813_14814insC | 1 |
| m.15674_15675insC | 1 |
| m.15940del | 5 |
| m.15942_15943insA | 1 |
| m.16032_16033insG | 1 |
| m.16057_16058insA | 1 |
| m.16091_16092insC | 1 |
| m.16161_16162insA | 1 |
| m.16180del | 3 |
| m.16180_16181del | 2 |
| m.16180_16181insC | 1 |
| (Continue) **Supplementary Table 3**. The distribution of mitochondrial DNA mutations per 856 untreated PLWH. | |
| Distinctive types of nucleotide variants | Count |
| m.16180_16181insT | 1 |
| m.16181_16182insC | 8 |
| m.16181_16182insCC | 5 |
| m.16182_16183insC | 11 |
| m.16182_16183insCC | 2 |
| m.16183_16184insT | 1 |
| m.16183_16184insC | 6 |
| m.16183_16184insCC | 1 |
| m.16186_16187insT | 2 |
| m.16189_16190insCC | 2 |
| m.16189del | 3 |
| m.16192_16193insT | 2 |
| m.16192_16193insC | 1 |
| m.16569insGATCACAGGTCTATCACCCTAT | 1 |
| m.16569del | 1 |
