# Supplementary material for: Baseline Characteristics of Mitochondrial DNA and Mutations Associated With Short-Term Posttreatment CD4+T-Cell Recovery in Chinese People With HIV
Source: Front Immunol. 2021 Dec 14;12:793375. doi: 10.3389/fimmu.2021.793375 (PMC8712318; doi:10.3389/fimmu.2021.793375)
Supplement: Supplementary file 1 [file DataSheet_1.zip › SupplementaryMaterial/Supplementary Table4.docx]

| **Supplementary Table 4a**. Physicochemical characteristics of the amino acid changes arose from 460 types of definite non-synonymous substitutions in 856 untreated PLWH. | |
| --- | --- |
| Category of change | N (%) |
| **Hydropathy** |  |
| Hydrophilic 🡪 hydrophilic | 23 (5.00) |
| Hydrophilic 🡪 hydrophobic | 1 (0.22) |
| Hydrophilic 🡪 neutral | 25 (5.43) |
| Hydrophilic 🡪 none (arose from the amino acid change to STOP) | 3 (0.65) |
| Hydrophobic 🡪 hydrophilic | 4 (0.87) |
| Hydrophobic 🡪 hydrophobic | 137 (29.78) |
| Hydrophobic 🡪 neutral | 111 (24.13) |
| Hydrophobic 🡪 none (arose from the amino acid change to STOP) | 2 (0.43) |
| Neutral 🡪 hydrophilic | 13 (2.83) |
| Neutral 🡪 hydrophobic | 93 (20.22) |
| Neutral 🡪 neutral | 44 (9.57) |
| Neutral 🡪 none (arose from the amino acid change to STOP) | 1 (0.22) |
| None (arose from the original amino acid of STOP) 🡪 hydrophilic | 1 (0.22) |
| None (arose from the original amino acid of STOP) 🡪 neutral | 1 (0.22) |
| None (arose from the original amino acid of STOP) 🡪 none (arose from the amino acid change to STOP) | 1 (0.22) |
| **Volume** |  |
| Large 🡪 large | 17 (3.70) |
| Large 🡪 medium | 47 (10.22) |
| Large 🡪 small | 51 (11.09) |
| Large 🡪 very large | 8 (1.74) |
| Large 🡪 very small | 1 (0.22) |
| Large 🡪 none (arose from the amino acid change to STOP) | 4 (0.87) |
| Medium 🡪 large | 35 (7.61) |
| Medium 🡪 medium | 3 (0.65) |
| Medium 🡪 small | 1 (0.22) |
| Medium 🡪 very large | 3 (0.65) |
| Medium 🡪 very small | 8 (1.74) |
| Medium 🡪 none (arose from the amino acid change to STOP) | 1 (0.22) |
| Small 🡪 large | 23 (5.00) |
| Small 🡪 medium | 1 (0.22) |
| Small 🡪 small | 19 (4.13) |
| Small 🡪 very large | 1 (0.22) |
| Small 🡪 very small | 87 (18.91) |
| Very large 🡪 large | 21 (4.57) |
| Very large 🡪 medium | 7 (1.52) |
| Very large 🡪 small | 7 (1.52) |
| Very large 🡪 very large | 1 (0.22) |
| Very large 🡪 very small | 10 (2.17) |
| (Continue) **Supplementary Table 4a**. Physicochemical characteristics of the amino acid changes arose from 460 types of definite non-synonymous substitutions in 856 untreated PLWH. | |
| Category of change | N (%) |
| Very large 🡪 none (arose from the amino acid change to STOP) | 1 (0.22) |
| Very small 🡪 large | 5 (1.09) |
| Very small 🡪 medium | 10 (2.17) |
| Very small 🡪 small | 62 (13.48) |
| Very small 🡪 very large | 4 (0.87) |
| Very small 🡪 very small | 19 (4.13) |
| None (arose from the original amino acid of STOP) 🡪 large | 1 (0.22) |
| None (arose from the original amino acid of STOP) 🡪 very small | 1 (0.22) |
| None (arose from the original amino acid of STOP) 🡪 none (arose from the amino acid change to STOP) | 1 (0.22) |
| **Chemical** |  |
| Acidic 🡪 acidic | 1 (0.22) |
| Acidic 🡪 aliphatic | 2 (0.43) |
| Acidic 🡪 amide | 9 (1.96) |
| Acidic 🡪 basic | 2 (0.43) |
| Acidic 🡪 none (arose from the amino acid change to STOP) | 1 (0.22) |
| Aliphatic 🡪 acidic | 2 (0.43) |
| Aliphatic 🡪 aliphatic | 94 (20.43) |
| Aliphatic 🡪 amide | 1 (0.22) |
| Aliphatic 🡪 aromatic | 9 (1.96) |
| Aliphatic 🡪 basic | 4 (0.87) |
| Aliphatic 🡪 hydroxyl | 86 (18.70) |
| Aliphatic 🡪 sulfur | 12 (2.61) |
| Aliphatic 🡪 none (arose from the amino acid change to STOP) | 1 (0.22) |
| Amide 🡪 acidic | 6 (1.30) |
| Amide 🡪 basic | 4 (0.87) |
| Amide 🡪 hydroxyl | 22 (4.78) |
| Aromatic 🡪 aliphatic | 21 (4.57) |
| Aromatic 🡪 amide | 1 (0.22) |
| Aromatic 🡪 aromatic | 1 (0.22) |
| Aromatic 🡪 basic | 7 (1.52) |
| Aromatic 🡪 hydroxyl | 10 (2.17) |
| Aromatic 🡪 sulfur | 6 (1.30) |
| Aromatic 🡪 none (arose from the amino acid change to STOP) | 1 (0.22) |
| Basic 🡪 acidic | 1 (0.22) |
| Basic 🡪 aliphatic | 1 (0.22) |
| Basic 🡪 amide | 2 (0.43) |
| Basic 🡪 aromatic | 3 (0.65) |
| Basic 🡪 basic | 2 (0.43) |
| Basic 🡪 none (arose from the amino acid change to STOP) | 2 (0.43) |
| (Continue) **Supplementary Table 4a**. Physicochemical characteristics of the amino acid changes arose from 460 types of definite non-synonymous substitutions in 856 untreated PLWH. | |
| Category of change | N (%) |
| Hydroxyl 🡪 aliphatic | 81 (17.61) |
| Hydroxyl 🡪 amide | 7 (1.52) |
| Hydroxyl 🡪 aromatic | 3 (0.65) |
| Hydroxyl 🡪 basic | 1 (0.22) |
| Hydroxyl 🡪 hydroxyl | 9 (1.96) |
| Hydroxyl 🡪 sulfur | 9 (1.96) |
| Sulfur 🡪 aliphatic | 11 (2.39) |
| Sulfur 🡪 aromatic | 1 (0.22) |
| Sulfur 🡪 hydroxyl | 19 (4.13) |
| Sulfur 🡪 sulfur | 1 (0.22) |
| Sulfur 🡪 none (arose from the amino acid change to STOP) | 1 (0.22) |
| None (arose from the original amino acid of STOP) 🡪 basic | 1 (0.22) |
| None (arose from the original amino acid of STOP) 🡪 hydroxyl | 1 (0.22) |
| None (arose from the original amino acid of STOP) 🡪 None (arose from the amino acid change to STOP) | 1 (0.22) |
| **Charge** |  |
| Negative 🡪 negative | 1 (0.22) |
| Negative 🡪 positive | 2 (0.43) |
| Negative 🡪 uncharged | 11 (2.39) |
| Negative 🡪 none (arose from the amino acid change to STOP) | 1 (0.22) |
| Positive 🡪 negative | 1 (0.22) |
| Positive 🡪 positive | 2 (0.43) |
| Positive 🡪 uncharged | 6 (1.30) |
| Positive 🡪 none (arose from the amino acid change to STOP) | 2 (0.43) |
| Uncharged 🡪 negative | 8 (1.74) |
| Uncharged 🡪 positive | 16 (3.48) |
| Uncharged 🡪 uncharged | 404 (87.83) |
| Uncharged 🡪 none (arose from the amino acid change to STOP) | 3 (0.65) |
| None (arose from the original amino acid of STOP) 🡪 positive | 1 (0.22) |
| None (arose from the original amino acid of STOP) 🡪 uncharged | 1 (0.22) |
| None (arose from the original amino acid of STOP) 🡪 none (arose from the amino acid change to STOP) | 1 (0.22) |
| **Hydrogen donor or acceptor atoms** |  |
| Acceptor 🡪 acceptor | 1 (0.22) |
| Acceptor 🡪 donor | 2 (0.43) |
| Acceptor 🡪 donor and acceptor | 9 (1.96) |
| Acceptor 🡪 neither donor nor acceptor | 2 (0.43) |
| Acceptor 🡪 none (arose from the amino acid change to STOP) | 1 (0.22) |
| Donor 🡪 acceptor | 1 (0.22) |
| Donor 🡪 donor | 1 (0.22) |
| (Continue) **Supplementary Table 4a**. Physicochemical characteristics of the amino acid changes arose from 460 types of definite non-synonymous substitutions in 856 untreated PLWH. | |
| Category of change | N (%) |
| Donor 🡪 donor and acceptor | 4 (0.87) |
| Donor 🡪 neither donor nor acceptor | 1 (0.22) |
| Donor 🡪 none (arose from the amino acid change to STOP) | 2 (0.43) |
| Donor and acceptor 🡪 acceptor | 6 (1.30) |
| Donor and acceptor 🡪 donor | 5 (1.09) |
| Donor and acceptor 🡪 donor and acceptor | 52 (11.30) |
| Donor and acceptor 🡪 neither donor nor acceptor | 98 (21.30) |
| Donor and acceptor 🡪 none (arose from the amino acid change to STOP) | 1 (0.22) |
| Neither donor nor acceptor 🡪 acceptor | 2 (0.43) |
| Neither donor nor acceptor 🡪 donor | 4 (0.87) |
| Neither donor nor acceptor 🡪 donor and acceptor | 116 (25.22) |
| Neither donor nor acceptor 🡪 neither donor nor acceptor | 147 (31.96) |
| Neither donor nor acceptor 🡪 none (arose from the amino acid change to STOP) | 2 (0.43) |
| None (arose from the original amino acid of STOP) 🡪 donor | 1 (0.22) |
| None (arose from the original amino acid of STOP) 🡪 donor and acceptor | 1 (0.22) |
| None (arose from the original amino acid of STOP) 🡪 none (arose from the amino acid change to STOP) | 1 (0.22) |
| **Polarity** |  |
| Nonpolar 🡪 nonpolar | 150 (32.61) |
| Nonpolar 🡪 polar | 123 (26.74) |
| Nonpolar 🡪 none (arose from the amino acid change to STOP) | 2 (0.43) |
| Polar 🡪 nonpolar | 100 (21.74) |
| Polar 🡪 polar | 78 (16.96) |
| Polar 🡪 none (arose from the amino acid change to STOP) | 4 (0.87) |
| None (arose from the original amino acid of STOP) 🡪 polar | 2 (0.43) |
| None (arose from the original amino acid of STOP) 🡪 none (arose from the amino acid change to STOP) | 1 (0.22) |

| **Supplementary Table 4b**. Physicochemical characteristics of the amino acid changes arose from 460 types of definite non-synonymous substitutions per sub-population. | |
| --- | --- |
| Category of change | N (%) |
| *Class1: Male, Han ethnic, Age 17-29, CD4 <200 (N=55)* | |
| **Hydropathy** |  |
| Hydrophilic 🡪 hydrophilic | 5 (5.68) |
| Hydrophilic 🡪 neutral | 5 (5.68) |
| Hydrophilic 🡪 none (arose from the amino acid change to STOP) | 1 (1.14) |
| Hydrophobic 🡪 hydrophobic | 30 (34.09) |
| Hydrophobic 🡪 neutral | 16 (18.18) |
| Neutral 🡪 hydrophilic | 1 (1.14) |
| Neutral 🡪 hydrophobic | 18 (20.45) |
| Neutral 🡪 neutral | 11 (12.50) |
| Neutral 🡪 none (arose from the amino acid change to STOP) | 1 (1.14) |
| **Volume** |  |
| Large 🡪 large | 1 (1.14) |
| Large 🡪 medium | 10 (11.36) |
| Large 🡪 small | 6 (6.82) |
| Large 🡪 very large | 2 (2.27) |
| Large 🡪 very small | 1 (1.14) |
| Large 🡪 none (arose from the amino acid change to STOP) | 12 (13.64) |
| Medium 🡪 large | 2 (2.27) |
| Medium 🡪 very large | 3 (3.41) |
| Medium 🡪 very small | 4 (4.55) |
| Medium 🡪 none (arose from the amino acid change to STOP) | 4 (4.55) |
| Small 🡪 large | 17 (19.32) |
| Small 🡪 small | 1 (1.14) |
| Small 🡪 very small | 4 (4.55) |
| Very large 🡪 large | 2 (2.27) |
| Very large 🡪 medium | 1 (1.14) |
| Very large 🡪 small | 1 (1.14) |
| Very large 🡪 very small | 3 (3.41) |
| Very large 🡪 none (arose from the amino acid change to STOP) | 12 (13.64) |
| Very small 🡪 medium | 2 (2.27) |
| Very small 🡪 small | 1 (1.14) |
| Very small 🡪 very small | 10 (11.36) |
| **Chemical** |  |
| Acidic 🡪 amide | 2 (2.27) |
| Acidic 🡪 basic | 1 (1.14) |
| Aliphatic 🡪 aliphatic | 25 (28.41) |
| Aliphatic 🡪 aromatic | 2 (2.27) |
| Aliphatic 🡪 hydroxyl | 11 (12.50) |
| Aliphatic 🡪 sulfur | 2 (2.27) |
| (Continue) **Supplementary Table 4b**. Physicochemical characteristics of the amino acid changes arose from 460 types of definite non-synonymous substitutions per sub-population. | |
| Category of change | N (%) |
| Amide 🡪 acidic | 2 (2.27) |
| Amide 🡪 hydroxyl | 5 (5.68) |
| Aromatic 🡪 aliphatic | 1 (1.14) |
| Aromatic 🡪 basic | 4 (4.55) |
| Aromatic 🡪 hydroxyl | 1 (1.14) |
| Aromatic 🡪 sulfur | 2 (2.27) |
| Aromatic 🡪 none (arose from the amino acid change to STOP) | 1 (1.14) |
| Basic 🡪 aliphatic | 1 (1.14) |
| Basic 🡪 aromatic | 2 (2.27) |
| Basic 🡪 none (arose from the amino acid change to STOP) | 1 (1.14) |
| Hydroxyl 🡪 aliphatic | 17 (19.32) |
| Hydroxyl 🡪 amide | 1 (1.14) |
| Hydroxyl 🡪 hydroxyl | 2 (2.27) |
| Hydroxyl 🡪 sulfur | 1 (1.14) |
| Sulfur 🡪 hydroxyl | 4 (4.55) |
| None (arose from the original amino acid of STOP) 🡪 None (arose from the amino acid change to STOP) | 2 (2.27) |
| **Charge** |  |
| Negative 🡪 positive | 1 (1.14) |
| Negative 🡪 uncharged | 2 (2.27) |
| Positive 🡪 uncharged | 3 (3.41) |
| Positive 🡪 none (arose from the amino acid change to STOP) | 1 (1.14) |
| Uncharged 🡪 negative | 2 (2.27) |
| Uncharged 🡪 positive | 4 (4.55) |
| Uncharged 🡪 uncharged | 74 (84.09) |
| Uncharged 🡪 none (arose from the amino acid change to STOP) | 1 (1.14) |
| None (arose from the original amino acid of STOP) 🡪 none (arose from the amino acid change to STOP) | 1 (1.14) |
| **Hydrogen donor or acceptor atoms** |  |
| Acceptor 🡪 donor | 1 (1.14) |
| Acceptor 🡪 donor and acceptor | 2 (2.27) |
| Donor 🡪 none (arose from the amino acid change to STOP) | 1 (1.14) |
| Donor and acceptor 🡪 acceptor | 2 (2.27) |
| Donor and acceptor 🡪 donor and acceptor | 14 (15.91) |
| Donor and acceptor 🡪 neither donor nor acceptor | 21 (23.86) |
| Donor and acceptor 🡪 none (arose from the amino acid change to STOP) | 1 (1.14) |
| Neither donor nor acceptor 🡪 donor and acceptor | 16 (18.18) |
| Neither donor nor acceptor 🡪 none (arose from the amino acid change to STOP) | 30 (34.09) |
| (Continue) **Supplementary Table 4b**. Physicochemical characteristics of the amino acid changes arose from 460 types of definite non-synonymous substitutions per sub-population. | |
| Category of change | N (%) |
| None (arose from the original amino acid of STOP) 🡪 none (arose from the amino acid change to STOP) | 1 (1.14) |
| **Polarity** |  |
| Nonpolar 🡪 nonpolar | 30 (34.09) |
| Nonpolar 🡪 polar | 16 (18.18) |
| Polar 🡪 nonpolar | 21 (23.86) |
| Polar 🡪 polar | 19 (21.59) |
| Polar 🡪 none (arose from the amino acid change to STOP) | 2 (2.27) |
| None (arose from the original amino acid of STOP) 🡪 none (arose from the amino acid change to STOP) | 30 (34.09) |
| *Class2: Male, Han ethnic, Age 30-44, CD4 <200 (N=99)* | |
| **Hydropathy** |  |
| Hydrophilic 🡪 hydrophilic | 6 (4.65) |
| Hydrophilic 🡪 neutral | 5 (3.88) |
| Hydrophobic 🡪 hydrophilic | 1 (0.78) |
| Hydrophobic 🡪 hydrophobic | 45 (34.88) |
| Hydrophobic 🡪 neutral | 28 (21.71) |
| Neutral 🡪 hydrophilic | 5 (3.88) |
| Neutral 🡪 hydrophobic | 28 (21.71) |
| Neutral 🡪 neutral | 10 (7.75) |
| None (arose from the original amino acid of STOP) 🡪 none (arose from the amino acid change to STOP) | 1 (0.78) |
| **Volume** |  |
| Large 🡪 large | 4 (3.10) |
| Large 🡪 medium | 13 (10.08) |
| Large 🡪 small | 11 (8.53) |
| Large 🡪 very large | 3 (2.33) |
| Medium 🡪 large | 11 (8.53) |
| Medium 🡪 very large | 1 (0.78) |
| Medium 🡪 very small | 4 (3.10) |
| Small 🡪 large | 7 (5.43) |
| Small 🡪 small | 7 (5.43) |
| Small 🡪 very small | 24 (18.60) |
| Very large 🡪 large | 7 (5.43) |
| Very large 🡪 medium | 3 (2.33) |
| Very large 🡪 small | 3 (2.33) |
| Very large 🡪 very small | 2 (1.55) |
| Very small 🡪 large | 1 (0.78) |
| Very small 🡪 medium | 3 (2.33) |
| Very small 🡪 small | 20 (15.50) |
| (Continue) **Supplementary Table 4b**. Physicochemical characteristics of the amino acid changes arose from 460 types of definite non-synonymous substitutions per sub-population. | |
| Category of change | N (%) |
| Very small 🡪 very large | 3 (2.33) |
| Very small 🡪 very small | 1 (0.78) |
| None (arose from the original amino acid of STOP) 🡪 none (arose from the amino acid change to STOP) | 1 (0.78) |
| **Chemical** |  |
| Acidic 🡪 aliphatic | 1 (0.78) |
| Acidic 🡪 amide | 5 (3.88) |
| Aliphatic 🡪 acidic | 1 (0.78) |
| Aliphatic 🡪 aliphatic | 32 (24.81) |
| Aliphatic 🡪 aromatic | 4 (3.10) |
| Aliphatic 🡪 basic | 1 (0.78) |
| Aliphatic 🡪 hydroxyl | 21 (16.28) |
| Aliphatic 🡪 sulfur | 2 (1.55) |
| Amide 🡪 acidic | 1 (0.78) |
| Amide 🡪 hydroxyl | 4 (3.10) |
| Aromatic 🡪 aliphatic | 7 (5.43) |
| Aromatic 🡪 amide | 1 (0.78) |
| Aromatic 🡪 basic | 3 (2.33) |
| Aromatic 🡪 hydroxyl | 2 (1.55) |
| Aromatic 🡪 sulfur | 2 (1.55) |
| Basic 🡪 aromatic | 1 (0.78) |
| Hydroxyl 🡪 aliphatic | 22 (17.05) |
| Hydroxyl 🡪 amide | 3 (2.33) |
| Hydroxyl 🡪 aromatic | 2 (1.55) |
| Hydroxyl 🡪 hydroxyl | 3 (2.33) |
| Hydroxyl 🡪 sulfur | 1 (0.78) |
| Sulfur 🡪 aliphatic | 2 (1.55) |
| Sulfur 🡪 hydroxyl | 7 (5.43) |
| None (arose from the original amino acid of STOP) 🡪 None (arose from the amino acid change to STOP) | 1 (0.78) |
| **Charge** |  |
| Negative 🡪 uncharged | 6 (4.65) |
| Positive 🡪 uncharged | 1 (0.78) |
| Uncharged 🡪 negative | 1 (1.55) |
| Uncharged 🡪 positive | 4 (3.10) |
| Uncharged 🡪 uncharged | 115 (89.15) |
| None (arose from the original amino acid of STOP) 🡪 none (arose from the amino acid change to STOP) | 1 (0.78) |
| **Hydrogen donor or acceptor atoms** |  |
| Acceptor 🡪 donor and acceptor | 5 (3.88) |
| (Continue) **Supplementary Table 4b**. Physicochemical characteristics of the amino acid changes arose from 460 types of definite non-synonymous substitutions per sub-population. | |
| Category of change | N (%) |
| Acceptor 🡪 neither donor nor acceptor | 1 (0.78) |
| Donor and acceptor 🡪 acceptor | 1 (0.78) |
| Donor and acceptor 🡪 donor and acceptor | 15 (11.63) |
| Donor and acceptor 🡪 neither donor nor acceptor | 27 (20.93) |
| Neither donor nor acceptor 🡪 acceptor | 1 (0.78) |
| Neither donor nor acceptor 🡪 donor | 2 (1.55) |
| Neither donor nor acceptor 🡪 donor and acceptor | 30 (23.26) |
| Neither donor nor acceptor 🡪 neither donor nor acceptor | 46 (35.66) |
| None (arose from the original amino acid of STOP) 🡪 none (arose from the amino acid change to STOP) | 1 (0.78) |
| **Polarity** |  |
| Nonpolar 🡪 nonpolar | 47 (36.43) |
| Nonpolar 🡪 polar | 32 (24.81) |
| Polar 🡪 nonpolar | 28 (21.71) |
| Polar 🡪 polar | 21 (16.28) |
| None (arose from the original amino acid of STOP) 🡪 none (arose from the amino acid change to STOP) | 1 (0.78) |
| *Class3: Male, Han ethnic, Age 45-59, CD4 <200 (N=65)* | |
| **Hydropathy** |  |
| Hydrophilic 🡪 hydrophilic | 3 (3.03) |
| Hydrophilic 🡪 neutral | 4 (4.04) |
| Hydrophobic 🡪 hydrophobic | 31 (31.31) |
| Hydrophobic 🡪 neutral | 28 (28.28) |
| Neutral 🡪 hydrophilic | 1 (1.01) |
| Neutral 🡪 hydrophobic | 23 (23.23) |
| Neutral 🡪 neutral | 9 (9.09) |
| **Volume** |  |
| Large 🡪 large | 2 (2.02) |
| Large 🡪 medium | 11 (11.11) |
| Large 🡪 small | 10 (10.10) |
| Large 🡪 very large | 2 (2.02) |
| Medium 🡪 large | 9 (9.09) |
| Medium 🡪 very large | 2 (2.02) |
| Medium 🡪 very small | 1 (1.01) |
| Small 🡪 large | 5 (5.05) |
| Small 🡪 small | 3 (3.03) |
| Small 🡪 very large | 1 (1.01) |
| Small 🡪 very small | 19 (19.19) |
| Very large 🡪 large | 4 (4.04) |
| Very large 🡪 medium | 3 (3.03) |
| (Continue) **Supplementary Table 4b**. Physicochemical characteristics of the amino acid changes arose from 460 types of definite non-synonymous substitutions per sub-population. | |
| Category of change | N (%) |
| Very large 🡪 small | 2 (2.02) |
| Very large 🡪 very small | 1 (1.01) |
| Very small 🡪 medium | 2 (2.02) |
| Very small 🡪 small | 19 (19.19) |
| Very small 🡪 very large | 1 (1.01) |
| Very small 🡪 very small | 2 (2.02) |
| **Chemical** |  |
| Acidic 🡪 amide | 2 (2.02) |
| Aliphatic 🡪 aliphatic | 24 (24.24) |
| Aliphatic 🡪 aromatic | 2 (2.02) |
| Aliphatic 🡪 hydroxyl | 24 (24.24) |
| Aliphatic 🡪 sulfur | 3 (3.03) |
| Amide 🡪 acidic | 1 (1.01) |
| Amide 🡪 hydroxyl | 4 (4.04) |
| Aromatic 🡪 aliphatic | 4 (4.04) |
| Aromatic 🡪 basic | 3 (3.03) |
| Aromatic 🡪 hydroxyl | 1 (1.01) |
| Aromatic 🡪 sulfur | 2 (2.02) |
| Basic 🡪 aromatic | 2 (2.02) |
| Hydroxyl 🡪 aliphatic | 18 (18.18) |
| Hydroxyl 🡪 amide | 1 (1.01) |
| Hydroxyl 🡪 aromatic | 1 (1.01) |
| Hydroxyl 🡪 hydroxyl | 2 (2.02) |
| Hydroxyl 🡪 sulfur | 1 (1.01) |
| Sulfur 🡪 aromatic | 1 (1.01) |
| Sulfur 🡪 hydroxyl | 3 (3.03) |
| **Charge** |  |
| Negative 🡪 uncharged | 2 (2.02) |
| Positive 🡪 uncharged | 2 (2.02) |
| Uncharged 🡪 negative | 1 (1.01) |
| Uncharged 🡪 positive | 3 (3.03) |
| Uncharged 🡪 uncharged | 91 (91.92) |
| **Hydrogen donor or acceptor atoms** |  |
| Acceptor 🡪 donor and acceptor | 2 (2.02) |
| Donor and acceptor 🡪 acceptor | 1 (1.01) |
| Donor and acceptor 🡪 donor and acceptor | 12 (12.12) |
| Donor and acceptor 🡪 neither donor nor acceptor | 22 (22.22) |
| Neither donor nor acceptor 🡪 donor and acceptor | 29 (29.29) |
| Neither donor nor acceptor 🡪 neither donor nor acceptor | 33 (33.33) |
| **Polarity** |  |
| (Continue) **Supplementary Table 4b**. Physicochemical characteristics of the amino acid changes arose from 460 types of definite non-synonymous substitutions per sub-population. | |
| Category of change | N (%) |
| Nonpolar 🡪 nonpolar | 33 (33.33) |
| Nonpolar 🡪 polar | 29 (29.29) |
| Polar 🡪 nonpolar | 22 (22.22) |
| Polar 🡪 polar | 15 (15.15) |
| *Class4: Male, Han ethnic, Age ≥60, CD4 <200 (N=37)* | |
| **Hydropathy** |  |
| Hydrophilic 🡪 hydrophilic | 2 (2.99) |
| Hydrophilic 🡪 neutral | 1 (1.49) |
| Hydrophobic 🡪 hydrophobic | 21 (31.34) |
| Hydrophobic 🡪 neutral | 17 (25.37) |
| Neutral 🡪 hydrophilic | 2 (2.99) |
| Neutral 🡪 hydrophobic | 16 (23.88) |
| Neutral 🡪 neutral | 8 (11.94) |
| **Volume** |  |
| Large 🡪 large | 1 (1.49) |
| Large 🡪 medium | 6 (8.96) |
| Large 🡪 small | 6 (8.96) |
| Large 🡪 very large | 2 (2.99) |
| Medium 🡪 large | 5 (7.46) |
| Medium 🡪 very large | 1 (1.49) |
| Medium 🡪 very small | 1 (1.49) |
| Small 🡪 large | 5 (7.46) |
| Small 🡪 small | 2 (2.99) |
| Small 🡪 very large | 1 (1.49) |
| Small 🡪 very small | 12 (17.91) |
| Very large 🡪 large | 2 (2.99) |
| Very large 🡪 medium | 3 (4.48) |
| Very large 🡪 small | 1 (1.49) |
| Very small 🡪 medium | 4 (5.97) |
| Very small 🡪 small | 14 (20.90) |
| Very small 🡪 very large | 1 (1.49) |
| **Chemical** |  |
| Acidic 🡪 amide | 2 (2.99) |
| Aliphatic 🡪 aliphatic | 16 (23.88) |
| Aliphatic 🡪 aromatic | 2 (2.99) |
| Aliphatic 🡪 hydroxyl | 15 (22.39) |
| Aliphatic 🡪 sulfur | 1 (1.49) |
| Amide 🡪 hydroxyl | 1 (1.49) |
| Aromatic 🡪 aliphatic | 2 (2.99) |
| Aromatic 🡪 basic | 3 (4.48) |
| (Continue) **Supplementary Table 4b**. Physicochemical characteristics of the amino acid changes arose from 460 types of definite non-synonymous substitutions per sub-population. | |
| Category of change | N (%) |
| Aromatic 🡪 sulfur | 1 (1.49) |
| Basic 🡪 aromatic | 1 (1.49) |
| Hydroxyl 🡪 aliphatic | 13 (19.40) |
| Hydroxyl 🡪 amide | 2 (2.99) |
| Hydroxyl 🡪 aromatic | 1 (1.49) |
| Hydroxyl 🡪 hydroxyl | 2 (2.99) |
| Hydroxyl 🡪 sulfur | 2 (2.99) |
| Sulfur 🡪 aromatic | 1 (1.49) |
| Sulfur 🡪 hydroxyl | 2 (2.99) |
| **Charge** |  |
| Negative 🡪 uncharged | 2 (2.99) |
| Positive 🡪 uncharged | 1 (1.49) |
| Uncharged 🡪 positive | 3 (4.48) |
| Uncharged 🡪 uncharged | 61 (91.04) |
| **Hydrogen donor or acceptor atoms** |  |
| Acceptor 🡪 donor and acceptor | 2 (2.99) |
| Donor and acceptor 🡪 donor and acceptor | 9 (13.43) |
| Donor and acceptor 🡪 neither donor nor acceptor | 17 (25.37) |
| Neither donor nor acceptor 🡪 donor and acceptor | 18 (26.87) |
| Neither donor nor acceptor 🡪 neither donor nor acceptor | 21 (31.34) |
| **Polarity** |  |
| Nonpolar 🡪 nonpolar | 21 (31.34) |
| Nonpolar 🡪 polar | 18 (26.87) |
| Polar 🡪 nonpolar | 17 (25.37) |
| Polar 🡪 polar | 11 (16.42) |
| *Class5: Male, Han ethnic, Age 17-29, CD4 ≥200 (N=97)* | |

| **Hydropathy** |  |
| --- | --- |
| Hydrophilic 🡪 hydrophilic | 6 (4.38) |
| Hydrophilic 🡪 neutral | 9 (6.57) |
| Hydrophobic 🡪 hydrophobic | 38 (27.74) |
| Hydrophobic 🡪 neutral | 37 (27.01) |
| Hydrophobic 🡪 none (arose from the amino acid change to STOP) | 1 (0.73) |
| Neutral 🡪 hydrophilic | 2 (1.46) |
| Neutral 🡪 hydrophobic | 26 (18.98) |
| Neutral 🡪 neutral | 16 (11.68) |
| None (arose from the original amino acid of STOP) 🡪 hydrophilic | 1 (0.73) |
| None (arose from the original amino acid of STOP) 🡪 none (arose from the amino acid change to STOP) | 1 (0.73) |
| **Volume** |  |
| Large 🡪 large | 5 (3.65) |

| (Continue) **Supplementary Table 4b**. Physicochemical characteristics of the amino acid changes arose from 460 types of definite non-synonymous substitutions per sub-population. | |
| --- | --- |
| Category of change | N (%) |

| Large 🡪 medium | 13 (9.49) |
| --- | --- |
| Large 🡪 small | 18 (13.14) |
| Large 🡪 very large | 3 (2.19) |
| Large 🡪 very small | 1 (0.73) |
| Large 🡪 none (arose from the amino acid change to STOP) | 1 (0.73) |
| Medium 🡪 large | 8 (5.84) |
| Medium 🡪 very large | 1 (0.73) |
| Medium 🡪 very small | 3 (2.19) |
| Small 🡪 large | 8 (5.84) |
| Small 🡪 small | 4 (2.92) |
| Small 🡪 very small | 30 (21.90) |
| Very large 🡪 large | 7 (5.11) |
| Very large 🡪 medium | 4 (2.92) |
| Very large 🡪 small | 1 (0.73) |
| Very large 🡪 very large | 1 (0.73) |
| Very large 🡪 very small | 2 (1.46) |
| Very small 🡪 large | 1 (0.73) |
| Very small 🡪 medium | 1 (0.73) |
| Very small 🡪 small | 20 (14.60) |
| Very small 🡪 very small | 3 (2.19) |
| None (arose from the original amino acid of STOP) 🡪 large | 1 (0.73) |
| None (arose from the original amino acid of STOP) 🡪 none (arose from the amino acid change to STOP) | 1 (0.73) |
| **Chemical** |  |
| Acidic 🡪 amide | 2 (1.46) |
| Aliphatic 🡪 aliphatic | 24 (17.52) |
| Aliphatic 🡪 aromatic | 3 (2.19) |
| Aliphatic 🡪 hydroxyl | 31 (22.63) |
| Aliphatic 🡪 sulfur | 4 (2.92) |
| Amide 🡪 acidic | 1 (0.73) |
| Amide 🡪 basic | 3 (2.19) |
| Amide 🡪 hydroxyl | 8 (5.84) |
| Aromatic 🡪 aliphatic | 7 (5.11) |
| Aromatic 🡪 aromatic | 1 (0.73) |
| Aromatic 🡪 basic | 4 (2.92) |
| Aromatic 🡪 hydroxyl | 2 (1.46) |
| Aromatic 🡪 sulfur | 1 (0.73) |
| Basic 🡪 aromatic | 1 (0.73) |
| Basic 🡪 basic | 1 (0.73) |
| Hydroxyl 🡪 aliphatic | 27 (19.71) |

| (Continue) **Supplementary Table 4b**. Physicochemical characteristics of the amino acid changes arose from 460 types of definite non-synonymous substitutions per sub-population. | |
| --- | --- |
| Category of change | N (%) |

| Hydroxyl 🡪 amide | 2 (1.46) |
| --- | --- |
| Hydroxyl 🡪 hydroxyl | 2 (1.46) |
| Hydroxyl 🡪 sulfur | 3 (2.19) |
| Sulfur 🡪 aliphatic | 2 (1.46) |
| Sulfur 🡪 hydroxyl | 5 (3.65) |
| Sulfur 🡪 none (arose from the amino acid change to STOP) | 1 (0.73) |
| None (arose from the original amino acid of STOP) 🡪 basic | 1 (0.73) |
| None (arose from the original amino acid of STOP) 🡪 None (arose from the amino acid change to STOP) | 1 (0.73) |
| **Charge** |  |
| Negative 🡪 uncharged | 2 (1.46) |
| Positive 🡪 positive | 1 (0.73) |
| Positive 🡪 uncharged | 1 (0.73) |
| Uncharged 🡪 negative | 1 (0.73) |
| Uncharged 🡪 positive | 7 (5.11) |
| Uncharged 🡪 uncharged | 122 (89.05) |
| Uncharged 🡪 none (arose from the amino acid change to STOP) | 1 (0.73) |
| None (arose from the original amino acid of STOP) 🡪 positive | 1 (0.73) |
| None (arose from the original amino acid of STOP) 🡪 none (arose from the amino acid change to STOP) | 1 (0.73) |
| **Hydrogen donor or acceptor atoms** |  |
| Acceptor 🡪 donor and acceptor | 2 (1.46) |
| Donor 🡪 donor | 1 (0.73) |
| Donor 🡪 donor and acceptor | 1 (0.73) |
| Donor and acceptor 🡪 acceptor | 1 (0.73) |
| Donor and acceptor 🡪 donor | 3 (2.19) |
| Donor and acceptor 🡪 donor and acceptor | 17 (12.41) |
| Donor and acceptor 🡪 none (arose from the amino acid change to STOP) | 31 (22.63) |
| Neither donor nor acceptor 🡪 donor and acceptor | 38 (27.74) |
| Neither donor nor acceptor 🡪 neither donor nor acceptor | 40 (29.20) |
| Neither donor nor acceptor 🡪 none (arose from the amino acid change to STOP) | 1 (0.73) |
| None (arose from the original amino acid of STOP) 🡪 donor | 1 (0.73) |
| None (arose from the original amino acid of STOP) 🡪 none (arose from the amino acid change to STOP) | 1 (0.73) |
| **Polarity** |  |
| Nonpolar 🡪 nonpolar | 41 (29.93) |
| Nonpolar 🡪 polar | 38 (27.74) |
| Nonpolar 🡪 none (arose from the amino acid change to STOP) | 1 (0.73) |

| (Continue) **Supplementary Table 4b**. Physicochemical characteristics of the amino acid changes arose from 460 types of definite non-synonymous substitutions per sub-population. | |
| --- | --- |
| Category of change | N (%) |

| Polar 🡪 nonpolar | 31 (22.63) |
| --- | --- |
| Polar 🡪 polar | 24 (17.52) |
| None (arose from the original amino acid of STOP) 🡪 polar | 1 (0.73) |
| None (arose from the original amino acid of STOP) 🡪 none (arose from the amino acid change to STOP) | 1 (0.73) |
| *Class6: Male, Han ethnic, Age 30-44, CD4 ≥200 (N=162)* | |
| **Hydropathy** |  |
| Hydrophilic 🡪 hydrophilic | 8 (4.76) |
| Hydrophilic 🡪 neutral | 9 (5.36) |
| Hydrophilic 🡪 none (arose from the amino acid change to STOP) | 1 (0.60) |
| Hydrophobic 🡪 hydrophobic | 56 (33.33) |
| Hydrophobic 🡪 neutral | 37 (22.02) |
| Neutral 🡪 hydrophilic | 5 (2.98) |
| Neutral 🡪 hydrophobic | 39 (23.21) |
| Neutral 🡪 neutral | 12 (7.14) |
| None (arose from the original amino acid of STOP) 🡪 hydrophilic | 1 (0.60) |
| **Volume** |  |
| Large 🡪 large | 6 (3.57) |
| Large 🡪 medium | 20 (11.90) |
| Large 🡪 small | 12 (7.14) |
| Large 🡪 very large | 3 (1.79) |
| Large 🡪 very small | 1 (0.60) |
| Medium 🡪 large | 12 (7.14) |
| Medium 🡪 medium | 1 (0.60) |
| Medium 🡪 very large | 1 (0.60) |
| Medium 🡪 very small | 3 (1.79) |
| Medium 🡪 none (arose from the amino acid change to STOP) | 1 (0.60) |
| Small 🡪 large | 6 (3.57) |
| Small 🡪 small | 8 (4.76) |
| Small 🡪 very large | 1 (0.60) |
| Small 🡪 very small | 37 (22.02) |
| Very large 🡪 large | 9 (5.36) |
| Very large 🡪 medium | 5 (2.98) |
| Very large 🡪 small | 2 (1.19) |
| Very large 🡪 very small | 5 (2.98) |
| Very small 🡪 medium | 6 (3.57) |
| Very small 🡪 small | 24 (14.29) |
| Very small 🡪 very large | 2 (1.19) |
| Very small 🡪 very small | 2 (1.19) |
| None (arose from the original amino acid of STOP) 🡪 large | 1 (0.60) |

| (Continue) **Supplementary Table 4b**. Physicochemical characteristics of the amino acid changes arose from 460 types of definite non-synonymous substitutions per sub-population. | |
| --- | --- |
| Category of change | N (%) |

| **Chemical** |  |
| --- | --- |
| Acidic 🡪 amide | 6 (3.57) |
| Acidic 🡪 basic | 1 (0.60) |
| Acidic 🡪 none (arose from the amino acid change to STOP) | 1 (0.60) |
| Aliphatic 🡪 acidic | 1 (0.60) |
| Aliphatic 🡪 aliphatic | 36 (21.43) |
| Aliphatic 🡪 aromatic | 4 (2.38) |
| Aliphatic 🡪 hydroxyl | 25 (14.88) |
| Aliphatic 🡪 sulfur | 6 (3.57) |
| Amide 🡪 acidic | 1 (0.60) |
| Amide 🡪 hydroxyl | 9 (5.36) |
| Aromatic 🡪 aliphatic | 9 (5.36) |
| Aromatic 🡪 basic | 5 (2.98) |
| Aromatic 🡪 hydroxyl | 5 (2.98) |
| Aromatic 🡪 sulfur | 2 (1.19) |
| Basic 🡪 amide | 1 (0.60) |
| Basic 🡪 aromatic | 1 (0.60) |
| Basic 🡪 basic | 1 (0.60) |
| Hydroxyl 🡪 aliphatic | 34 (20.24) |
| Hydroxyl 🡪 amide | 2 (1.19) |
| Hydroxyl 🡪 aromatic | 1 (0.60) |
| Hydroxyl 🡪 hydroxyl | 3 (1.79) |
| Hydroxyl 🡪 sulfur | 2 (1.19) |
| Sulfur 🡪 aliphatic | 4 (2.38) |
| Sulfur 🡪 aromatic | 1 (0.60) |
| Sulfur 🡪 hydroxyl | 6 (3.57) |
| None (arose from the original amino acid of STOP) 🡪 basic | 1 (0.60) |
| **Charge** |  |
| Negative 🡪 positive | 1 (0.60) |
| Negative 🡪 uncharged | 6 (3.57) |
| Negative 🡪 none (arose from the amino acid change to STOP) | 1 (0.60) |
| Positive 🡪 positive | 1 (0.60) |
| Positive 🡪 uncharged | 2 (1.19) |
| Uncharged 🡪 negative | 2 (1.19) |
| Uncharged 🡪 positive | 5 (2.98) |
| Uncharged 🡪 uncharged | 149 (88.69) |
| None (arose from the original amino acid of STOP) 🡪 positive | 1 (0.60) |
| **Hydrogen donor or acceptor atoms** |  |
| Acceptor 🡪 donor | 1 (0.60) |
| Acceptor 🡪 donor and acceptor | 6 (3.57) |

| (Continue) **Supplementary Table 4b**. Physicochemical characteristics of the amino acid changes arose from 460 types of definite non-synonymous substitutions per sub-population. | |
| --- | --- |
| Category of change | N (%) |

| Acceptor 🡪 none (arose from the amino acid change to STOP) | 1 (0.60) |
| --- | --- |
| Donor 🡪 donor and acceptor | 1 (0.60) |
| Donor and acceptor 🡪 acceptor | 1 (0.60) |
| Donor and acceptor 🡪 donor | 1 (0.60) |
| Donor and acceptor 🡪 donor and acceptor | 21 (12.50) |
| Donor and acceptor 🡪 neither donor nor acceptor | 39 (23.21) |
| Neither donor nor acceptor 🡪 acceptor | 1 (0.60) |
| Neither donor nor acceptor 🡪 donor | 1 (0.60) |
| Neither donor nor acceptor 🡪 donor and acceptor | 36 (21.43) |
| Neither donor nor acceptor 🡪 neither donor nor acceptor | 58 (34.52) |
| None (arose from the original amino acid of STOP) 🡪 donor | 1 (0.60) |
| **Polarity** |  |
| Nonpolar 🡪 nonpolar | 59 (35.12) |
| Nonpolar 🡪 polar | 38 (22.62) |
| Polar 🡪 nonpolar | 39 (23.21) |
| Polar 🡪 polar | 30 (17.86) |
| Polar 🡪 none (arose from the amino acid change to STOP) | 1 (0.60) |
| None (arose from the original amino acid of STOP) 🡪 polar | 1 (0.60) |
| *Class7: Male, Han ethnic, Age 45-59, CD4 ≥200 (N=61)* | |
| **Hydropathy** |  |
| Hydrophilic 🡪 hydrophilic | 4 (4.12) |
| Hydrophilic 🡪 hydrophobic | 1 (1.03) |
| Hydrophilic 🡪 neutral | 3 (3.09) |
| Hydrophobic 🡪 hydrophobic | 25 (25.77) |
| Hydrophobic 🡪 neutral | 20 (20.62) |
| Neutral 🡪 hydrophilic | 3 (3.09) |
| Neutral 🡪 hydrophobic | 27 (27.84) |
| Neutral 🡪 neutral | 13 (13.40) |
| None (arose from the original amino acid of STOP) 🡪 hydrophilic | 1 (1.03) |
| **Volume** |  |
| Large 🡪 large | 1 (1.03) |
| Large 🡪 medium | 9 (9.28) |
| Large 🡪 small | 7 (7.22) |
| Large 🡪 very large | 3 (3.09) |
| Medium 🡪 large | 7 (7.22) |
| Medium 🡪 medium | 1 (1.03) |
| Medium 🡪 small | 1 (1.03) |
| Medium 🡪 very large | 1 (1.03) |
| Small 🡪 large | 5 (5.15) |
| Small 🡪 small | 2 (2.06) |

| (Continue) **Supplementary Table 4b**. Physicochemical characteristics of the amino acid changes arose from 460 types of definite non-synonymous substitutions per sub-population. | |
| --- | --- |
| Category of change | N (%) |

| Small 🡪 very small | 24 (24.74) |
| --- | --- |
| Very large 🡪 large | 2 (2.06) |
| Very large 🡪 medium | 4 (4.12) |
| Very large 🡪 small | 1 (1.03) |
| Very large 🡪 very small | 2 (2.06) |
| Very small 🡪 large | 1 (1.03) |
| Very small 🡪 medium | 3 (3.09) |
| Very small 🡪 small | 17 (17.53) |
| Very small 🡪 very large | 1 (1.03) |
| Very small 🡪 very small | 4 (4.12) |
| None (arose from the original amino acid of STOP) 🡪 large | 1 (1.03) |
| **Chemical** |  |
| Acidic 🡪 acidic | 1 (1.03) |
| Acidic 🡪 aliphatic | 1 (1.03) |
| Acidic 🡪 amide | 1 (1.03) |
| Aliphatic 🡪 aliphatic | 18 (18.56) |
| Aliphatic 🡪 aromatic | 3 (3.09) |
| Aliphatic 🡪 hydroxyl | 16 (16.49) |
| Aliphatic 🡪 sulfur | 2 (2.06) |
| Amide 🡪 acidic | 1 (1.03) |
| Amide 🡪 hydroxyl | 3 (3.09) |
| Aromatic 🡪 aliphatic | 2 (2.06) |
| Aromatic 🡪 basic | 4 (4.12) |
| Aromatic 🡪 hydroxyl | 2 (2.06) |
| Aromatic 🡪 sulfur | 1 (1.03) |
| Basic 🡪 amide | 1 (1.03) |
| Basic 🡪 aromatic | 1 (1.03) |
| Hydroxyl 🡪 aliphatic | 25 (25.77) |
| Hydroxyl 🡪 amide | 3 (3.09) |
| Hydroxyl 🡪 aromatic | 1 (1.03) |
| Hydroxyl 🡪 hydroxyl | 4 (4.12) |
| Hydroxyl 🡪 sulfur | 2 (2.06) |
| Sulfur 🡪 aliphatic | 1 (1.03) |
| Sulfur 🡪 hydroxyl | 3 (3.09) |
| None (arose from the original amino acid of STOP) 🡪 basic | 1 (1.03) |
| **Charge** |  |
| Negative 🡪 negative | 1 (1.03) |
| Negative 🡪 uncharged | 2 (2.06) |
| Positive 🡪 uncharged | 2 (2.06) |
| Uncharged 🡪 negative | 1 (1.03) |

| (Continue) **Supplementary Table 4b**. Physicochemical characteristics of the amino acid changes arose from 460 types of definite non-synonymous substitutions per sub-population. | |
| --- | --- |
| Category of change | N (%) |

| Uncharged 🡪 positive | 4 (4.12) |
| --- | --- |
| Uncharged 🡪 uncharged | 86 (88.66) |
| None (arose from the original amino acid of STOP) 🡪 positive | 1 (1.03) |
| **Hydrogen donor or acceptor atoms** |  |
| Acceptor 🡪 acceptor | 1 (1.03) |
| Acceptor 🡪 donor and acceptor | 1 (1.03) |
| Acceptor 🡪 neither donor nor acceptor | 1 (1.03) |
| Donor 🡪 donor and acceptor | 1 (1.03) |
| Donor and acceptor 🡪 acceptor | 1 (1.03) |
| Donor and acceptor 🡪 donor and acceptor | 16 (16.49) |
| Donor and acceptor 🡪 neither donor nor acceptor | 28 (28.87) |
| Neither donor nor acceptor 🡪 donor and acceptor | 21 (21.65) |
| Neither donor nor acceptor 🡪 neither donor nor acceptor | 26 (26.80) |
| None (arose from the original amino acid of STOP) 🡪 donor | 1 (1.03) |
| **Polarity** |  |
| Nonpolar 🡪 nonpolar | 26 (26.80) |
| Nonpolar 🡪 polar | 21 (21.65) |
| Polar 🡪 nonpolar | 29 (29.90) |
| Polar 🡪 polar | 20 (20.62) |
| None (arose from the original amino acid of STOP) 🡪 polar | 1 (1.03) |
| *Class8: Male, Han ethnic, Age ≥60, CD4 ≥200 (N=37)* | |
| **Hydropathy** |  |
| Hydrophilic 🡪 hydrophilic | 1 (1.47) |
| Hydrophilic 🡪 neutral | 2 (2.94) |
| Hydrophobic 🡪 hydrophobic | 16 (23.53) |
| Hydrophobic 🡪 neutral | 17 (25.00) |
| Neutral 🡪 hydrophilic | 1 (1.47) |
| Neutral 🡪 hydrophobic | 24 (35.29) |
| Neutral 🡪 neutral | 7 (10.29) |
| **Volume** |  |
| Large 🡪 large | 2 (2.94) |
| Large 🡪 medium | 5 (7.35) |
| Large 🡪 small | 7 (10.29) |
| Large 🡪 very large | 2 (2.94) |
| Medium 🡪 large | 4 (5.88) |
| Medium 🡪 very large | 1 (1.47) |
| Small 🡪 large | 5 (7.35) |
| Small 🡪 small | 2 (2.94) |
| Small 🡪 very large | 1 (1.47) |
| Small 🡪 very small | 19 (27.94) |

| (Continue) **Supplementary Table 4b**. Physicochemical characteristics of the amino acid changes arose from 460 types of definite non-synonymous substitutions per sub-population. | |
| --- | --- |
| Category of change | N (%) |

| Very large 🡪 large | 2 (2.94) |
| --- | --- |
| Very large 🡪 medium | 3 (4.41) |
| Very large 🡪 small | 1 (1.47) |
| Very large 🡪 very small | 2 (2.94) |
| Very small 🡪 large | 1 (1.47) |
| Very small 🡪 small | 10 (14.71) |
| Very small 🡪 very large | 1 (1.47) |
| **Chemical** |  |
| Acidic 🡪 amide | 1 (1.47) |
| Aliphatic 🡪 aliphatic | 10 (14.71) |
| Aliphatic 🡪 aromatic | 2 (2.94) |
| Aliphatic 🡪 hydroxyl | 9 (13.24) |
| Aliphatic 🡪 sulfur | 1 (1.47) |
| Amide 🡪 hydroxyl | 2 (2.94) |
| Aromatic 🡪 aliphatic | 2 (2.94) |
| Aromatic 🡪 basic | 3 (4.41) |
| Aromatic 🡪 hydroxyl | 2 (2.94) |
| Aromatic 🡪 sulfur | 1 (1.47) |
| Basic 🡪 aromatic | 1 (1.47) |
| Hydroxyl 🡪 aliphatic | 22 (32.35) |
| Hydroxyl 🡪 amide | 1 (1.47) |
| Hydroxyl 🡪 aromatic | 1 (1.47) |
| Hydroxyl 🡪 hydroxyl | 2 (2.94) |
| Hydroxyl 🡪 sulfur | 1 (1.47) |
| Sulfur 🡪 aromatic | 1 (1.47) |
| Sulfur 🡪 hydroxyl | 5 (7.35) |
| Sulfur 🡪 sulfur | 1 (1.47) |
| **Charge** |  |
| Negative 🡪 uncharged | 1 (1.47) |
| Positive 🡪 uncharged | 1 (1.47) |
| Uncharged 🡪 positive | 3 (4.41) |
| Uncharged 🡪 uncharged | 63 (92.65) |
| **Hydrogen donor or acceptor atoms** |  |
| Acceptor 🡪 donor and acceptor | 1 (1.47) |
| Donor 🡪 donor and acceptor | 1 (1.47) |
| Donor and acceptor 🡪 donor and acceptor | 9 (13.24) |
| Donor and acceptor 🡪 neither donor nor acceptor | 25 (36.76) |
| Neither donor nor acceptor 🡪 donor and acceptor | 16 (23.53) |
| Neither donor nor acceptor 🡪 neither donor nor acceptor | 16 (23.53) |
| **Polarity** |  |

| (Continue) **Supplementary Table 4b**. Physicochemical characteristics of the amino acid changes arose from 460 types of definite non-synonymous substitutions per sub-population. | |
| --- | --- |
| Category of change | N (%) |

| Nonpolar 🡪 nonpolar | 16 (23.53) |
| --- | --- |
| Nonpolar 🡪 polar | 17 (25.00) |
| Polar 🡪 nonpolar | 25 (36.76) |
| Polar 🡪 polar | 10 (14.71) |
| *Class9: Female, Han ethnic, Age 17-29, CD4 <200 (N=28)* | |
| **Hydropathy** |  |
| Hydrophilic 🡪 hydrophilic | 4 (6.67) |
| Hydrophilic 🡪 neutral | 3 (5.00) |
| Hydrophobic 🡪 hydrophobic | 15 (25.00) |
| Hydrophobic 🡪 neutral | 13 (21.67) |
| Neutral 🡪 hydrophilic | 1 (1.67) |
| Neutral 🡪 hydrophobic | 16 (26.67) |
| Neutral 🡪 neutral | 7 (11.67) |
| None (arose from the original amino acid of STOP) 🡪 neutral | 1 (1.67) |
| **Volume** |  |
| Large 🡪 large | 1 (1.67) |
| Large 🡪 medium | 5 (8.33) |
| Large 🡪 small | 6 (10.00) |
| Large 🡪 very large | 1 (1.67) |
| Medium 🡪 large | 6 (10.00) |
| Medium 🡪 very large | 1 (1.67) |
| Small 🡪 large | 3 (5.00) |
| Small 🡪 small | 4 (6.67) |
| Small 🡪 very large | 1 (1.67) |
| Small 🡪 very small | 16 (26.67) |
| Very large 🡪 large | 1 (1.67) |
| Very large 🡪 medium | 2 (3.33) |
| Very small 🡪 medium | 1 (1.67) |
| Very small 🡪 small | 9 (15.00) |
| Very small 🡪 very large | 1 (1.67) |
| Very small 🡪 very small | 1 (1.67) |
| None (arose from the original amino acid of STOP) 🡪 very small | 1 (1.67) |
| **Chemical** |  |
| Acidic 🡪 amide | 2 (3.33) |
| Aliphatic 🡪 aliphatic | 12 (20.00) |
| Aliphatic 🡪 aromatic | 1 (1.67) |
| Aliphatic 🡪 hydroxyl | 10 (16.67) |
| Aliphatic 🡪 sulfur | 1 (1.67) |
| Amide 🡪 acidic | 2 (3.33) |
| Amide 🡪 hydroxyl | 3 (5.00) |

| (Continue) **Supplementary Table 4b**. Physicochemical characteristics of the amino acid changes arose from 460 types of definite non-synonymous substitutions per sub-population. | |
| --- | --- |
| Category of change | N (%) |

| Aromatic 🡪 aliphatic | 1 (1.67) |
| --- | --- |
| Aromatic 🡪 basic | 2 (3.33) |
| Basic 🡪 aromatic | 1 (1.67) |
| Hydroxyl 🡪 aliphatic | 16 (26.67) |
| Hydroxyl 🡪 amide | 1 (1.67) |
| Hydroxyl 🡪 aromatic | 1 (1.67) |
| Hydroxyl 🡪 hydroxyl | 2 (3.33) |
| Sulfur 🡪 aromatic | 1 (1.67) |
| Sulfur 🡪 hydroxyl | 3 (5.00) |
| None (arose from the original amino acid of STOP) 🡪 hydroxyl | 1 (1.67) |
| **Charge** |  |
| Negative 🡪 uncharged | 2 (3.33) |
| Positive 🡪 uncharged | 1 (1.67) |
| Uncharged 🡪 negative | 2 (3.33) |
| Uncharged 🡪 positive | 2 (3.33) |
| Uncharged 🡪 uncharged | 52 (86.67) |
| None (arose from the original amino acid of STOP) 🡪 uncharged | 1 (1.67) |
| **Hydrogen donor or acceptor atoms** |  |
| Acceptor 🡪 donor and acceptor | 2 (3.33) |
| Donor and acceptor 🡪 acceptor | 2 (3.33) |
| Donor and acceptor 🡪 donor and acceptor | 9 (15.00) |
| Donor and acceptor 🡪 neither donor nor acceptor | 17 (28.33) |
| Neither donor nor acceptor 🡪 donor and acceptor | 14 (23.33) |
| Neither donor nor acceptor 🡪 neither donor nor acceptor | 15 (25.00) |
| None (arose from the original amino acid of STOP) 🡪 donor and acceptor | 1 (1.67) |
| **Polarity** |  |
| Nonpolar 🡪 nonpolar | 15 (25.00) |
| Nonpolar 🡪 polar | 14 (23.33) |
| Polar 🡪 nonpolar | 17 (28.33) |
| Polar 🡪 polar | 13 (21.67) |
| None (arose from the original amino acid of STOP) 🡪 polar | 1 (1.67) |
| *Class10: Female, Han ethnic, Age 30-44, CD4 <200 (N=34)* | |
| **Hydropathy** |  |
| Hydrophilic 🡪 hydrophilic | 2 (3.45) |
| Hydrophobic 🡪 hydrophobic | 16 (27.59) |
| Hydrophobic 🡪 neutral | 19 (32.76) |
| Neutral 🡪 hydrophilic | 1 (1.72) |
| Neutral 🡪 hydrophobic | 13 (22.41) |
| Neutral 🡪 neutral | 7 (12.07) |

| (Continue) **Supplementary Table 4b**. Physicochemical characteristics of the amino acid changes arose from 460 types of definite non-synonymous substitutions per sub-population. | |
| --- | --- |
| Category of change | N (%) |

| **Volume** |  |
| --- | --- |
| Large 🡪 large | 2 (3.45) |
| Large 🡪 medium | 6 (10.34) |
| Large 🡪 small | 9 (15.52) |
| Large 🡪 very large | 1 (1.72) |
| Medium 🡪 large | 4 (6.90) |
| Small 🡪 large | 3 (5.17) |
| Small 🡪 small | 1 (1.72) |
| Small 🡪 very large | 1 (1.72) |
| Small 🡪 very small | 13 (22.41) |
| Very large 🡪 large | 2 (3.45) |
| Very large 🡪 medium | 2 (3.45) |
| Very small 🡪 medium | 2 (3.45) |
| Very small 🡪 small | 11 (18.97) |
| Very small 🡪 very small | 1 (1.72) |
| **Chemical** |  |
| Acidic 🡪 amide | 1 (1.72) |
| Acidic 🡪 basic | 1 (1.72) |
| Aliphatic 🡪 aliphatic | 10 (17.24) |
| Aliphatic 🡪 aromatic | 1 (1.72) |
| Aliphatic 🡪 hydroxyl | 14 (24.14) |
| Aliphatic 🡪 sulfur | 1 (1.72) |
| Aromatic 🡪 aliphatic | 2 (3.45) |
| Aromatic 🡪 basic | 2 (3.45) |
| Hydroxyl 🡪 aliphatic | 14 (24.14) |
| Hydroxyl 🡪 amide | 1 (1.72) |
| Hydroxyl 🡪 hydroxyl | 3 (5.17) |
| Sulfur 🡪 aliphatic | 2 (3.45) |
| Sulfur 🡪 aromatic | 1 (1.72) |
| Sulfur 🡪 hydroxyl | 5 (8.62) |
| **Charge** |  |
| Negative 🡪 positive | 1 (1.72) |
| Negative 🡪 uncharged | 1 (1.72) |
| Uncharged 🡪 positive | 2 (3.45) |
| Uncharged 🡪 uncharged | 54 (93.10) |
| **Hydrogen donor or acceptor atoms** |  |
| Acceptor 🡪 donor | 1 (1.72) |
| Acceptor 🡪 donor and acceptor | 1 (1.72) |
| Donor 🡪 donor and acceptor | 6 (10.34) |
| Donor 🡪 neither donor nor acceptor | 14 (24.14) |

| (Continue) **Supplementary Table 4b**. Physicochemical characteristics of the amino acid changes arose from 460 types of definite non-synonymous substitutions per sub-population. | |
| --- | --- |
| Category of change | N (%) |

| Neither donor nor acceptor 🡪 donor and acceptor | 20 (34.48) |
| --- | --- |
| Neither donor nor acceptor 🡪 neither donor nor acceptor | 16 (27.59) |
| **Polarity** |  |
| Nonpolar 🡪 nonpolar | 16 (27.59) |
| Nonpolar 🡪 polar | 20 (34.48) |
| Polar 🡪 nonpolar | 14 (24.14) |
| Polar 🡪 polar | 8 (13.79) |
| *Class11: Female, Han ethnic, Age 45-59, CD4 <200 (N=12)* | |
| **Hydropathy** |  |
| Hydrophilic 🡪 neutral | 1 (3.23) |
| Hydrophobic 🡪 hydrophobic | 8 (25.81) |
| Hydrophobic 🡪 neutral | 8 (25.81) |
| Neutral 🡪 hydrophobic | 9 (29.03) |
| Neutral 🡪 neutral | 5 (16.13) |
| **Volume** |  |
| Large 🡪 large | 1 (3.23) |
| Large 🡪 medium | 4 (12.90) |
| Large 🡪 small | 4 (12.90) |
| Medium 🡪 large | 2 (6.45) |
| Medium 🡪 very large | 1 (3.23) |
| Small 🡪 large | 1 (3.23) |
| Small 🡪 very small | 10 (32.26) |
| Very large 🡪 large | 1 (3.23) |
| Very large 🡪 medium | 1 (3.23) |
| Very large 🡪 small | 1 (3.23) |
| Very small 🡪 small | 5 (16.13) |
| **Chemical** |  |
| Aliphatic 🡪 aliphatic | 5 (16.13) |
| Aliphatic 🡪 hydroxyl | 5 (16.13) |
| Aliphatic 🡪 sulfur | 1 (3.23) |
| Amide 🡪 hydroxyl | 1 (3.23) |
| Aromatic 🡪 aliphatic | 1 (3.23) |
| Aromatic 🡪 basic | 1 (3.23) |
| Aromatic 🡪 sulfur | 1 (3.23) |
| Basic 🡪 aromatic | 1 (3.23) |
| Hydroxyl 🡪 aliphatic | 8 (25.81) |
| Hydroxyl 🡪 hydroxyl | 3 (9.68) |
| Sulfur 🡪 aliphatic | 1 (3.23) |
| Sulfur 🡪 hydroxyl | 3 (9.68) |
| **Charge** |  |

| (Continue) **Supplementary Table 4b**. Physicochemical characteristics of the amino acid changes arose from 460 types of definite non-synonymous substitutions per sub-population. | |
| --- | --- |
| Category of change | N (%) |

| Positive 🡪 uncharged | 1 (3.23) |
| --- | --- |
| Uncharged 🡪 positive | 1 (3.23) |
| Uncharged 🡪 uncharged | 29 (93.55) |
| **Hydrogen donor or acceptor atoms** |  |
| Donor and acceptor 🡪 donor and acceptor | 6 (19.35) |
| Donor and acceptor 🡪 neither donor nor acceptor | 9 (29.03) |
| Neither donor nor acceptor 🡪 donor and acceptor | 8 (25.81) |
| Neither donor nor acceptor 🡪 neither donor nor acceptor | 8 (25.81) |
| **Polarity** |  |
| Nonpolar 🡪 nonpolar | 8 (25.81) |
| Nonpolar 🡪 polar | 8 (25.81) |
| Polar 🡪 nonpolar | 9 (29.03) |
| Polar 🡪 polar | 6 (19.35) |
| *Class12: Female, Han ethnic, Age ≥60, CD4 <200 (N=6)* | |
| **Hydropathy** |  |
| Hydrophilic 🡪 neutral | 2 (8.33) |
| Hydrophobic 🡪 hydrophobic | 5 (20.83) |
| Hydrophobic 🡪 neutral | 5 (20.83) |
| Neutral 🡪 hydrophilic | 1 (4.17) |
| Neutral 🡪 hydrophobic | 9 (37.50) |
| Neutral 🡪 neutral | 2 (8.33) |
| **Volume** |  |
| Large 🡪 large | 1 (4.17) |
| Large 🡪 medium | 2 (8.33) |
| Large 🡪 small | 2 (8.33) |
| Medium 🡪 large | 1 (4.17) |
| Small 🡪 large | 1 (4.17) |
| Small 🡪 very small | 8 (33.33) |
| Very small 🡪 large | 1 (4.17) |
| Very small 🡪 medium | 1 (4.17) |
| Very small 🡪 small | 5 (20.83) |
| Very small 🡪 very large | 1 (4.17) |
| Very small 🡪 very small | 1 (4.17) |
| **Chemical** |  |
| Aliphatic 🡪 aliphatic | 4 (16.67) |
| Aliphatic 🡪 hydroxyl | 3 (12.50) |
| Aliphatic 🡪 sulfur | 1 (4.17) |
| Amide 🡪 hydroxyl | 2 (8.33) |
| Hydroxyl 🡪 aliphatic | 9 (37.50) |
| Hydroxyl 🡪 amide | 1 (4.17) |

| (Continue) **Supplementary Table 4b**. Physicochemical characteristics of the amino acid changes arose from 460 types of definite non-synonymous substitutions per sub-population. | |
| --- | --- |
| Category of change | N (%) |

| Hydroxyl 🡪 aromatic | 1 (4.17) |
| --- | --- |
| Hydroxyl 🡪 hydroxyl | 1 (4.17) |
| Sulfur 🡪 hydroxyl | 2 (8.33) |
| **Charge** |  |
| Uncharged 🡪 uncharged | 24 (100.00) |
| **Hydrogen donor or acceptor atoms** |  |
| Donor and acceptor 🡪 donor and acceptor | 4 (16.67) |
| Donor and acceptor 🡪 neither donor nor acceptor | 10 (41.67) |
| Neither donor nor acceptor 🡪 donor and acceptor | 5 (20.83) |
| Neither donor nor acceptor 🡪 neither donor nor acceptor | 5 (20.83) |
| **Polarity** |  |
| Nonpolar 🡪 nonpolar | 5 (20.83) |
| Nonpolar 🡪 polar | 5 (20.83) |
| Polar 🡪 nonpolar | 10 (41.67) |
| Polar 🡪 polar | 4 (16.67) |
| *Class13: Female, Han ethnic, Age 17-29, CD4 ≥200 (N=57)* | |
| **Hydropathy** |  |
| Hydrophilic 🡪 hydrophilic | 6 (5.61) |
| Hydrophilic 🡪 neutral | 4 (3.74) |
| Hydrophobic 🡪 hydrophilic | 1 (0.93) |
| Hydrophobic 🡪 hydrophobic | 30 (28.04) |
| Hydrophobic 🡪 neutral | 31 (28.97) |
| Neutral 🡪 hydrophilic | 2 (1.87) |
| Neutral 🡪 hydrophobic | 23 (21.50) |
| Neutral 🡪 neutral | 9 (8.41) |
| None (arose from the original amino acid of STOP) 🡪 hydrophilic | 1 (0.93) |
| **Volume** |  |
| Large 🡪 large | 2 (1.87) |
| Large 🡪 medium | 16 (14.95) |
| Large 🡪 small | 12 (11.21) |
| Large 🡪 very large | 1 (0.93) |
| Large 🡪 very small | 1 (0.93) |
| Medium 🡪 large | 6 (5.61) |
| Medium 🡪 medium | 1 (0.93) |
| Medium 🡪 very large | 1 (0.93) |
| Medium 🡪 very small | 2 (1.87) |
| Small 🡪 large | 2 (1.87) |
| Small 🡪 small | 6 (5.61) |
| Small 🡪 very large | 1 (0.93) |
| Small 🡪 very small | 20 (18.69) |

| (Continue) **Supplementary Table 4b**. Physicochemical characteristics of the amino acid changes arose from 460 types of definite non-synonymous substitutions per sub-population. | |
| --- | --- |
| Category of change | N (%) |

| Very large 🡪 large | 2 (1.87) |
| --- | --- |
| Very large 🡪 medium | 3 (2.80) |
| Very large 🡪 small | 3 (2.80) |
| Very large 🡪 very small | 3 (2.80) |
| Very small 🡪 medium | 2 (1.87) |
| Very small 🡪 small | 19 (17.76) |
| Very small 🡪 very large | 1 (0.93) |
| Very small 🡪 very small | 2 (1.87) |
| None (arose from the original amino acid of STOP) 🡪 large | 1 (0.93) |
| **Chemical** |  |
| Acidic 🡪 amide | 3 (2.80) |
| Aliphatic 🡪 aliphatic | 24 (22.43) |
| Aliphatic 🡪 aromatic | 1 (0.93) |
| Aliphatic 🡪 basic | 1 (0.93) |
| Aliphatic 🡪 hydroxyl | 20 (18.69) |
| Aliphatic 🡪 sulfur | 3 (2.80) |
| Amide 🡪 acidic | 3 (2.80) |
| Amide 🡪 basic | 1 (0.93) |
| Amide 🡪 hydroxyl | 3 (2.80) |
| Aromatic 🡪 aliphatic | 2 (1.87) |
| Aromatic 🡪 basic | 3 (2.80) |
| Aromatic 🡪 hydroxyl | 3 (2.80) |
| Aromatic 🡪 sulfur | 3 (2.80) |
| Basic 🡪 aromatic | 1 (0.93) |
| Hydroxyl 🡪 aliphatic | 19 (17.76) |
| Hydroxyl 🡪 amide | 2 (1.87) |
| Hydroxyl 🡪 aromatic | 1 (0.93) |
| Hydroxyl 🡪 hydroxyl | 3 (2.80) |
| Sulfur 🡪 aliphatic | 1 (0.93) |
| Sulfur 🡪 aromatic | 1 (0.93) |
| Sulfur 🡪 hydroxyl | 8 (7.48) |
| None (arose from the original amino acid of STOP) 🡪 basic | 1 (0.93) |
| **Charge** |  |
| Negative 🡪 uncharged | 3 (2.80) |
| Positive 🡪 uncharged | 1 (0.93) |
| Uncharged 🡪 negative | 3 (2.80) |
| Uncharged 🡪 positive | 5 (4.67) |
| Uncharged 🡪 uncharged | 94 (87.85) |
| None (arose from the original amino acid of STOP) 🡪 positive | 1 (0.93) |
| **Hydrogen donor or acceptor atoms** |  |

| (Continue) **Supplementary Table 4b**. Physicochemical characteristics of the amino acid changes arose from 460 types of definite non-synonymous substitutions per sub-population. | |
| --- | --- |
| Category of change | N (%) |

| Acceptor 🡪 donor and acceptor | 3 (2.80) |
| --- | --- |
| Donor and acceptor 🡪 acceptor | 3 (2.80) |
| Donor and acceptor 🡪 donor and acceptor | 13 (12.15) |
| Donor and acceptor 🡪 neither donor nor acceptor | 23 (21.50) |
| Neither donor nor acceptor 🡪 donor | 1 (0.93) |
| Neither donor nor acceptor 🡪 donor and acceptor | 32 (29.91) |
| Neither donor nor acceptor 🡪 neither donor nor acceptor | 31 (28.97) |
| None (arose from the original amino acid of STOP) 🡪 donor | 1 (0.93) |
| **Polarity** |  |
| Nonpolar 🡪 nonpolar | 31 (28.97) |
| Nonpolar 🡪 polar | 33 (30.84) |
| Polar 🡪 nonpolar | 23 (21.50) |
| Polar 🡪 polar | 19 (17.76) |
| None (arose from the original amino acid of STOP) 🡪 polar | 1 (0.93) |
| *Class14: Female, Han ethnic, Age 30-44, CD4 ≥200 (N=26)* | |
| **Hydropathy** |  |
| Hydrophilic 🡪 hydrophilic | 1 (1.85) |
| Hydrophilic 🡪 neutral | 1 (1.85) |
| Hydrophobic 🡪 hydrophobic | 25 (46.30) |
| Hydrophobic 🡪 neutral | 11 (20.37) |
| Neutral 🡪 hydrophilic | 1 (1.85) |
| Neutral 🡪 hydrophobic | 7 (12.96) |
| Neutral 🡪 neutral | 8 (14.81) |
| **Volume** |  |
| Large 🡪 large | 2 (3.70) |
| Large 🡪 medium | 7 (12.96) |
| Large 🡪 small | 2 (3.70) |
| Large 🡪 very large | 2 (3.70) |
| Medium 🡪 large | 7 (12.96) |
| Medium 🡪 very large | 1 (1.85) |
| Medium 🡪 very small | 1 (1.85) |
| Small 🡪 large | 1 (1.85) |
| Small 🡪 small | 1 (1.85) |
| Small 🡪 very small | 9 (16.67) |
| Very large 🡪 large | 3 (5.56) |
| Very large 🡪 medium | 3 (5.56) |
| Very large 🡪 small | 1 (1.85) |
| Very small 🡪 medium | 2 (3.70) |
| Very small 🡪 small | 11 (20.37) |
| Very small 🡪 very small | 1 (1.85) |

| (Continue) **Supplementary Table 4b**. Physicochemical characteristics of the amino acid changes arose from 460 types of definite non-synonymous substitutions per sub-population. | |
| --- | --- |
| Category of change | N (%) |

| **Chemical** |  |
| --- | --- |
| Acidic 🡪 amide | 1 (1.85） |
| Aliphatic 🡪 aliphatic | 16 (29.63） |
| Aliphatic 🡪 aromatic | 2 (3.70） |
| Aliphatic 🡪 hydroxyl | 9 (16.67） |
| Aliphatic 🡪 sulfur | 2 (3.70） |
| Amide 🡪 hydroxyl | 1 (1.85） |
| Aromatic 🡪 aliphatic | 3 (5.56） |
| Aromatic 🡪 basic | 3 (5.56） |
| Aromatic 🡪 sulfur | 1 (1.85） |
| Basic 🡪 aromatic | 1 (1.85） |
| Hydroxyl 🡪 aliphatic | 8 (14.81） |
| Hydroxyl 🡪 amide | 1 (1.85） |
| Hydroxyl 🡪 hydroxyl | 3 (5.56） |
| Sulfur 🡪 aliphatic | 1 (1.85） |
| Sulfur 🡪 hydroxyl | 2 (3.70） |
| **Charge** |  |
| Negative 🡪 uncharged | 1 (1.85) |
| Positive 🡪 uncharged | 1 (1.85) |
| Uncharged 🡪 positive | 3 (5.56) |
| Uncharged 🡪 uncharged | 49 (90.74) |
| **Hydrogen donor or acceptor atoms** |  |
| Acceptor 🡪 donor and acceptor | 1 (1.85) |
| Donor 🡪 neither donor nor acceptor | 1 (1.85) |
| Donor and acceptor 🡪 donor and acceptor | 9 (16.67) |
| Donor and acceptor 🡪 neither donor nor acceptor | 8 (14.81) |
| Neither donor nor acceptor 🡪 donor and acceptor | 11 (20.37) |
| Neither donor nor acceptor 🡪 neither donor nor acceptor | 24 (44.44) |
| **Polarity** |  |
| Nonpolar 🡪 nonpolar | 25 (46.30) |
| Nonpolar 🡪 polar | 11 (20.37) |
| Polar 🡪 nonpolar | 8 (14.81) |
| Polar 🡪 polar | 10 (18.52) |
| *Class15: Female, Han ethnic, Age 45-59, CD4 ≥200 (N=22)* | |
| **Hydropathy** |  |
| Hydrophilic 🡪 hydrophilic | 3 (5.45) |
| Hydrophilic 🡪 neutral | 2 (3.64) |
| Hydrophobic 🡪 hydrophobic | 16 (29.09) |
| Hydrophobic 🡪 neutral | 14 (25.45) |
| Neutral 🡪 hydrophilic | 1 (1.82) |

| (Continue) **Supplementary Table 4b**. Physicochemical characteristics of the amino acid changes arose from 460 types of definite non-synonymous substitutions per sub-population. | |
| --- | --- |
| Category of change | N (%) |

| Neutral 🡪 hydrophobic | 11 (20.00) |
| --- | --- |
| Neutral 🡪 neutral | 8 (14.55) |
| **Volume** |  |
| Large 🡪 large | 1 (1.82) |
| Large 🡪 medium | 6 (10.91) |
| Large 🡪 small | 6 (10.91) |
| Large 🡪 very large | 2 (3.64) |
| Medium 🡪 large | 5 (9.09) |
| Medium 🡪 very large | 1 (1.82) |
| Medium 🡪 very small | 1 (1.82) |
| Small 🡪 large | 2 (3.64) |
| Small 🡪 small | 3 (5.45) |
| Small 🡪 very small | 11 (20.00) |
| Very large 🡪 medium | 3 (5.45) |
| Very large 🡪 small | 1 (1.82) |
| Very small 🡪 medium | 1 (1.82) |
| Very small 🡪 small | 11 (20.00) |
| Very small 🡪 very small | 1 (1.82) |
| **Chemical** |  |
| Acidic 🡪 amide | 2 (3.64) |
| Aliphatic 🡪 aliphatic | 12 (21.82) |
| Aliphatic 🡪 aromatic | 2 (3.64) |
| Aliphatic 🡪 hydroxyl | 11 (20.00) |
| Aliphatic 🡪 sulfur | 2 (3.64) |
| Amide 🡪 acidic | 1 (1.82) |
| Amide 🡪 hydroxyl | 2 (3.64) |
| Aromatic 🡪 basic | 3 (5.45) |
| Aromatic 🡪 sulfur | 1 (1.82) |
| Basic 🡪 aromatic | 1 (1.82) |
| Hydroxyl 🡪 aliphatic | 10 (18.18) |
| Hydroxyl 🡪 amide | 1 (1.82) |
| Hydroxyl 🡪 hydroxyl | 1 (1.82) |
| Sulfur 🡪 aliphatic | 1 (1.82) |
| Sulfur 🡪 hydroxyl | 5 (9.09) |
| **Charge** |  |
| Negative 🡪 uncharged | 2 (3.64) |
| Positive 🡪 uncharged | 1 (1.82) |
| Uncharged 🡪 negative | 1 (1.82) |
| Uncharged 🡪 positive | 3 (5.45) |
| Uncharged 🡪 uncharged | 48 (87.27) |

| (Continue) **Supplementary Table 4b**. Physicochemical characteristics of the amino acid changes arose from 460 types of definite non-synonymous substitutions per sub-population. | |
| --- | --- |
| Category of change | N (%) |

| **Hydrogen donor or acceptor atoms** |  |
| --- | --- |
| Acceptor 🡪 donor and acceptor | 2 (3.64) |
| Donor and acceptor 🡪 acceptor | 1 (1.82) |
| Donor and acceptor 🡪 donor and acceptor | 8 (14.55) |
| Donor and acceptor 🡪 neither donor nor acceptor | 11 (20.00) |
| Neither donor nor acceptor 🡪 donor and acceptor | 16 (29.09) |
| Neither donor nor acceptor 🡪 neither donor nor acceptor | 17 (30.91) |
| **Polarity** |  |
| Nonpolar 🡪 nonpolar | 17 (30.91) |
| Nonpolar 🡪 polar | 16 (29.09) |
| Polar 🡪 nonpolar | 11 (20.00) |
| Polar 🡪 polar | 11 (20.00) |
| *Class16: Female, Han ethnic, Age ≥60, CD4 ≥200 (N=8)* | |
| **Hydropathy** |  |
| Hydrophilic 🡪 hydrophilic | 1 (4.00) |
| Hydrophilic 🡪 neutral | 3 (12.00) |
| Hydrophilic 🡪 none (arose from the amino acid change to STOP) | 1 (4.00) |
| Hydrophobic 🡪 hydrophobic | 6 (24.00) |
| Hydrophobic 🡪 neutral | 4 (16.00) |
| Neutral 🡪 hydrophobic | 8 (32.00) |
| Neutral 🡪 neutral | 2 (8.00) |
| **Volume** |  |
| Large 🡪 large | 1 (4.00) |
| Large 🡪 medium | 2 (8.00) |
| Large 🡪 small | 1 (4.00) |
| Large 🡪 very large | 1 (4.00) |
| Large 🡪 none (arose from the amino acid change to STOP) | 1 (4.00) |
| Medium 🡪 large | 1 (4.00) |
| Medium 🡪 very large | 1 (4.00) |
| Small 🡪 large | 1 (4.00) |
| Small 🡪 small | 1 (4.00) |
| Small 🡪 very small | 10 (40.00) |
| Very large 🡪 large | 1 (4.00) |
| Very small 🡪 small | 4 (16.00) |
| **Chemical** |  |
| Acidic 🡪 amide | 1 (4.00) |
| Aliphatic 🡪 aliphatic | 3 (12.00) |
| Aliphatic 🡪 aromatic | 1 (4.00) |
| Aliphatic 🡪 hydroxyl | 4 (16.00) |
| Aliphatic 🡪 sulfur | 1 (4.00) |

| (Continue) **Supplementary Table 4b**. Physicochemical characteristics of the amino acid changes arose from 460 types of definite non-synonymous substitutions per sub-population. | |
| --- | --- |
| Category of change | N (%) |

| Amide 🡪 hydroxyl | 3 (12.00) |
| --- | --- |
| Aromatic 🡪 aliphatic | 1 (4.00) |
| Basic 🡪 aromatic | 1 (4.00) |
| Basic 🡪 none (arose from the amino acid change to STOP) | 1 (4.00) |
| Hydroxyl 🡪 aliphatic | 9 (36.00) |
| **Charge** |  |
| Negative 🡪 uncharged | 1 (4.00) |
| Positive 🡪 uncharged | 1 (4.00) |
| Positive 🡪 none (arose from the amino acid change to STOP) | 1 (4.00) |
| Uncharged 🡪 uncharged | 22 (88.00) |
| **Hydrogen donor or acceptor atoms** |  |
| Acceptor 🡪 donor and acceptor | 1 (4.00) |
| Donor 🡪 none (arose from the amino acid change to STOP) | 1 (4.00) |
| Donor and acceptor 🡪 donor and acceptor | 4 (16.00) |
| Donor and acceptor 🡪 neither donor nor acceptor | 9 (36.00) |
| Neither donor nor acceptor 🡪 donor and acceptor | 4 (16.00) |
| Neither donor nor acceptor 🡪 neither donor nor acceptor | 6 (24.00) |
| **Polarity** |  |
| Nonpolar 🡪 nonpolar | 6 (24.00) |
| Nonpolar 🡪 polar | 4 (16.00) |
| Polar 🡪 nonpolar | 9 (36.00) |
| Polar 🡪 polar | 5 (20.00) |
| Polar 🡪 none (arose from the amino acid change to STOP) | 1 (4.00) |
