# Supplementary material for: Baseline Characteristics of Mitochondrial DNA and Mutations Associated With Short-Term Posttreatment CD4+T-Cell Recovery in Chinese People With HIV
Source: Front Immunol. 2021 Dec 14;12:793375. doi: 10.3389/fimmu.2021.793375 (PMC8712318; doi:10.3389/fimmu.2021.793375)
Supplement: Supplementary file 1 [file DataSheet_1.zip › SupplementaryMaterial/Supplementary Table5.docx]

| **Supplementary Table 5**. Linear slopes of relative diversity density of synonymous and non-synonymous substitutions per sub-population. | | |
| --- | --- | --- |
| Sub-population | Synonymous substitutions | Non-synonymous substitutions |
| Class1: Male, Han ethnic, Age 17-29, CD4 <200 | 1766.84 | 4869.45 |
| Class2: Male, Han ethnic, Age 30-44, CD4 <200 | 2653.52 | 9433.87 |
| Class3: Male, Han ethnic, Age 45-59, CD4 <200 | 2033.65 | 5128.39 |
| Class4: Male, Han ethnic, Age ≥60, CD4 <200 | 1911.60 | 5632.96 |
| Class5: Male, Han ethnic, Age 17-29, CD4 ≥200 | 2618.58 | 7493.28 |
| Class6: Male, Han ethnic, Age 30-44, CD4 ≥200 | 3403.71 | 13281.14 |
| Class7: Male, Han ethnic, Age 45-59, CD4 ≥200 | 2140.70 | 6184.00 |
| Class8: Male, Han ethnic, Age ≥60, CD4 ≥200 | 1855.37 | 4710.94 |
| Class9: Female, Han ethnic, Age 17-29, CD4 <200 | 1526.64 | 4353.46 |
| Class10: Female, Han ethnic, Age 30-44, CD4 <200 | 1643.90 | 5833.72 |
| Class11: Female, Han ethnic, Age 45-59, CD4 <200 | 961.54 | 3533.10 |
| Class12: Female, Han ethnic, Age ≥60, CD4 <200 | 876.69 | 1460.50 |
| Class13: Female, Han ethnic, Age 17-29, CD4 ≥200 | 1972.48 | 5990.21 |
| Class14: Female, Han ethnic, Age 30-44, CD4 ≥200 | 1394.95 | 4452.15 |
| Class15: Female, Han ethnic, Age 45-59, CD4 ≥200 | 1158.25 | 3578.49 |
| Class16: Female, Han ethnic, Age ≥60, CD4 ≥200 | 852.21 | 1877.69 |
