# Supplementary material for: Baseline Characteristics of Mitochondrial DNA and Mutations Associated With Short-Term Posttreatment CD4+T-Cell Recovery in Chinese People With HIV
Source: Front Immunol. 2021 Dec 14;12:793375. doi: 10.3389/fimmu.2021.793375 (PMC8712318; doi:10.3389/fimmu.2021.793375)
Supplement: Supplementary file 1 [file DataSheet_1.zip › SupplementaryMaterial/Supplementary Table6.docx]

| **Supplementary Table 6**. Associations of mtDNA substitutions with pre-ART CD4+T cell counts in mtDNA genome-wide association analyses. | | | | | |
| --- | --- | --- | --- | --- | --- |
| Substitution | β | P | 95% CI | SE | t |

| m.93A>G | -21.83 | 0.746 | (-154.37, 110.70) | 67.51 | -0.32 |
| --- | --- | --- | --- | --- | --- |
| m.103G>A | -31.05 | 0.625 | (-155.69, 93.58) | 63.48 | -0.49 |
| m.143G>A | -56.46 | 0.450 | (-203.07, 90.15) | 74.67 | -0.76 |
| m.146T>C | 2.10 | 0.860 | (-21.16, 25.35) | 11.85 | 0.18 |
| m.150C>T | 5.29 | 0.602 | (-14.65, 25.24) | 10.16 | 0.52 |
| m.151C>T | 20.97 | 0.623 | (-62.76, 104.71) | 42.65 | 0.49 |
| m.152T>C | 8.93 | 0.404 | (-12.07, 29.94) | 10.70 | 0.83 |
| m.153A>G | 1.72 | 0.962 | (-68.15, 71.58) | 35.59 | 0.05 |
| m.185G>A | 3.49 | 0.910 | (-56.84, 63.81) | 30.73 | 0.11 |
| m.189A>G | 3.10 | 0.934 | (-69.95, 76.16) | 37.21 | 0.08 |
| m.194C>T | -23.44 | 0.396 | (-77.65, 30.76) | 27.61 | -0.85 |
| m.195T>C | 29.05 | 0.141 | (-9.61, 67.71) | 19.69 | 1.48 |
| m.199T>C | 1.76 | 0.890 | (-23.05, 26.57) | 12.64 | 0.14 |
| m.200A>G | -22.11 | 0.452 | (-79.82, 35.60) | 29.39 | -0.75 |
| m.204T>C | 0.15 | 0.994 | (-36.53, 36.83) | 18.68 | 0.01 |
| m.207G>A | -0.49 | 0.981 | (-39.86, 38.89) | 20.06 | -0.02 |
| m.210A>G | -21.83 | 0.528 | (-89.68, 46.01) | 34.56 | -0.63 |
| m.215A>G | -3.62 | 0.955 | (-128.06, 120.83) | 63.39 | -0.06 |
| m.217T>C | 86.18 | 0.203 | (-46.53, 218.88) | 67.59 | 1.27 |
| m.235A>G | 13.31 | 0.431 | (-19.86, 46.47) | 16.89 | 0.79 |
| m.248A>G | 34.12 | 0.273 | (-26.89, 95.13) | 31.08 | 1.10 |
| m.263A>G | -66.00 | 0.064 | (-135.94, 3.94) | 35.62 | -1.85 |
| m.298C>T | 50.09 | 0.252 | (-35.74, 135.92) | 43.72 | 1.15 |
| m.310T>C | -4.23 | 0.630 | (-21.49, 13.03) | 8.79 | -0.48 |
| m.318T>C | -2.49 | 0.937 | (-64.52, 59.55) | 31.60 | -0.08 |
| m.456C>T | -10.59 | 0.853 | (-122.71, 101.53) | 57.11 | -0.19 |
| m.489T>C | -8.23 | 0.359 | (-25.84, 9.39) | 8.97 | -0.92 |
| m.499G>A | -6.83 | 0.812 | (-63.33, 49.67) | 28.78 | -0.24 |
| m.663A>G | 13.31 | 0.440 | (-20.51, 47.13) | 17.23 | 0.77 |
| m.681T>C | -10.83 | 0.844 | (-119.03, 97.36) | 55.11 | -0.20 |
| m.709G>A | -3.74 | 0.713 | (-23.73, 16.25) | 10.18 | -0.37 |
| m.735A>G | -26.88 | 0.597 | (-126.75, 73.00) | 50.87 | -0.53 |
| m.752C>T | -18.76 | 0.546 | (-79.73, 42.20) | 31.05 | -0.60 |
| m.827A>G | -6.83 | 0.781 | (-54.98, 41.31) | 24.52 | -0.28 |
| m.961T>C | 17.07 | 0.409 | (-23.50, 57.65) | 20.67 | 0.83 |
| m.1005T>C | 22.46 | 0.409 | (-30.96, 75.88) | 27.21 | 0.83 |
| m.1048C>T | -10.59 | 0.845 | (-117.14, 95.96) | 54.27 | -0.20 |
| m.1107T>C | -18.11 | 0.543 | (-76.54, 40.32) | 29.76 | -0.61 |
| m.1119T>C | -0.82 | 0.966 | (-38.89, 37.25) | 19.39 | -0.04 |
| m.1382A>C | 5.46 | 0.855 | (-52.99, 63.91) | 29.77 | 0.18 |

| (Continue) **Supplementary Table 6**. Associations of mtDNA substitutions with pre-ART CD4+T cell counts in mtDNA genome-wide association analyses. | | | | | |
| --- | --- | --- | --- | --- | --- |
| Substitution | β | P | 95% CI | SE | t |

| m.1438A>G | 18.88 | 0.532 | (-40.47, 78.23) | 30.23 | 0.62 |
| --- | --- | --- | --- | --- | --- |
| m.1598G>A | 7.38 | 0.865 | (-77.85, 92.62) | 43.41 | 0.17 |
| m.1709G>A | 40.00 | 0.220 | (-23.93, 103.93) | 32.56 | 1.23 |
| m.1719G>A | -6.89 | 0.865 | (-86.26, 72.49) | 40.43 | -0.17 |
| m.1736A>G | 16.73 | 0.321 | (-16.36, 49.82) | 16.85 | 0.99 |
| m.1824T>C | 24.43 | 0.355 | (-27.44, 76.30) | 26.42 | 0.92 |
| m.2835C>T | -50.79 | 0.166 | (-122.74, 21.16) | 36.65 | -1.39 |
| m.3010G>A | 0.42 | 0.977 | (-27.51, 28.34) | 14.22 | 0.03 |
| m.3206C>T | 14.18 | 0.783 | (-86.78, 115.14) | 51.43 | 0.28 |
| m.3316G>A | -18.78 | 0.391 | (-61.71, 24.15) | 21.87 | -0.86 |
| m.3394T>C | -24.39 | 0.451 | (-87.84, 39.06) | 32.32 | -0.75 |
| m.3398T>C | 6.95 | 0.918 | (-124.73, 138.63) | 67.07 | 0.10 |
| m.3483G>A | 8.36 | 0.879 | (-99.24, 115.96) | 54.81 | 0.15 |
| m.3497C>T | 6.85 | 0.712 | (-29.53, 43.22) | 18.53 | 0.37 |
| m.3537A>G | -21.83 | 0.549 | (-93.39, 49.72) | 36.45 | -0.60 |
| m.3552T>A | 3.37 | 0.854 | (-32.64, 39.39) | 18.34 | 0.18 |
| m.3571C>T | 20.15 | 0.333 | (-20.68, 60.98) | 20.80 | 0.97 |
| m.3606A>G | -17.31 | 0.720 | (-112.06, 77.45) | 48.26 | -0.36 |
| m.3834G>A | -0.31 | 0.994 | (-82.82, 82.19) | 42.02 | -0.01 |
| m.3882G>A | -12.41 | 0.690 | (-73.41, 48.59) | 31.07 | -0.40 |
| m.3970C>T | 13.00 | 0.345 | (-14.03, 40.03) | 13.77 | 0.94 |
| m.4048G>A | 7.78 | 0.666 | (-27.57, 43.12) | 18.00 | 0.43 |
| m.4071C>T | 1.76 | 0.899 | (-25.48, 28.99) | 13.87 | 0.13 |
| m.4086C>T | 0.46 | 0.980 | (-36.08, 37.00) | 18.61 | 0.02 |
| m.4140C>T | 58.46 | 0.167 | (-24.44, 141.36) | 42.23 | 1.38 |
| m.4164A>G | 7.13 | 0.696 | (-28.66, 42.92) | 18.23 | 0.39 |
| m.4216T>C | 86.15 | 0.109 | (-19.21, 191.52) | 53.67 | 1.61 |
| m.4248T>C | 13.31 | 0.440 | (-20.51, 47.13) | 17.23 | 0.77 |
| m.4386T>C | 11.31 | 0.563 | (-27.07, 49.68) | 19.55 | 0.58 |
| m.4491G>A | -38.86 | 0.342 | (-119.11, 41.38) | 40.87 | -0.95 |
| m.4715A>G | -3.97 | 0.756 | (-29.06, 21.12) | 12.78 | -0.31 |
| m.4820G>A | -6.83 | 0.812 | (-63.33, 49.67) | 28.78 | -0.24 |
| m.4824A>G | 13.31 | 0.392 | (-17.22, 43.84) | 15.55 | 0.86 |
| m.4833A>G | -34.52 | 0.084 | (-73.69, 4.65) | 19.95 | -1.73 |
| m.4850C>T | -12.41 | 0.582 | (-56.61, 31.79) | 22.51 | -0.55 |
| m.4883C>T | -12.00 | 0.374 | (-38.51, 14.51) | 13.50 | -0.89 |
| m.5108T>C | -40.42 | 0.065 | (-83.34, 2.50) | 21.86 | -1.85 |
| m.5147G>A | -23.54 | 0.486 | (-89.85, 42.77) | 33.77 | -0.70 |
| m.5153A>G | -10.83 | 0.844 | (-119.03, 97.36) | 55.11 | -0.20 |
| m.5178C>A | -11.86 | 0.385 | (-38.64, 14.92) | 13.64 | -0.87 |

| (Continue) **Supplementary Table 6**. Associations of mtDNA substitutions with pre-ART CD4+T cell counts in mtDNA genome-wide association analyses. | | | | | |
| --- | --- | --- | --- | --- | --- |
| Substitution | β | P | 95% CI | SE | t |

| m.5231G>A | 11.52 | 0.558 | (-27.11, 50.14) | 19.67 | 0.59 |
| --- | --- | --- | --- | --- | --- |
| m.5301A>G | -18.11 | 0.564 | (-79.69, 43.47) | 31.37 | -0.58 |
| m.5351A>G | 7.13 | 0.696 | (-28.66, 42.92) | 18.23 | 0.39 |
| m.5417G>A | -0.31 | 0.986 | (-35.65, 35.02) | 18.00 | -0.02 |
| m.5442T>C | -12.57 | 0.563 | (-55.23, 30.08) | 21.73 | -0.58 |
| m.5460G>A | 10.05 | 0.559 | (-23.67, 43.78) | 17.18 | 0.59 |
| m.5465T>C | 21.05 | 0.557 | (-49.27, 91.37) | 35.82 | 0.59 |
| m.5585G>A | 19.39 | 0.627 | (-58.81, 97.60) | 39.83 | 0.49 |
| m.5587T>C | -35.38 | 0.640 | (-183.93, 113.16) | 75.66 | -0.47 |
| m.5601C>T | -20.28 | 0.287 | (-57.66, 17.10) | 19.04 | -1.07 |
| m.5628T>C | 41.30 | 0.399 | (-54.79, 137.39) | 48.94 | 0.84 |
| m.5821G>A | 9.92 | 0.701 | (-40.82, 60.67) | 25.85 | 0.38 |
| m.6023G>A | -6.89 | 0.842 | (-74.80, 61.02) | 34.59 | -0.20 |
| m.6179G>A | -50.94 | 0.110 | (-113.49, 11.62) | 31.86 | -1.60 |
| m.6216T>C | -14.37 | 0.710 | (-90.14, 61.40) | 38.59 | -0.37 |
| m.6253T>C | -10.83 | 0.852 | (-125.16, 103.49) | 58.23 | -0.19 |
| m.6392T>C | 20.93 | 0.131 | (-6.24, 48.11) | 13.84 | 1.51 |
| m.6413T>C | -10.06 | 0.795 | (-86.02, 65.90) | 38.69 | -0.26 |
| m.6455C>T | 1.76 | 0.899 | (-25.48, 28.99) | 13.87 | 0.13 |
| m.6599A>G | 23.92 | 0.645 | (-77.96, 125.81) | 51.89 | 0.46 |
| m.6680T>C | 4.30 | 0.816 | (-31.98, 40.59) | 18.48 | 0.23 |
| m.6752A>G | 15.56 | 0.642 | (-50.05, 81.16) | 33.41 | 0.47 |
| m.6960C>T | -2.87 | 0.940 | (-77.34, 71.59) | 37.93 | -0.08 |
| m.6962G>A | 9.62 | 0.581 | (-24.61, 43.84) | 17.43 | 0.55 |
| m.7196C>A | -6.07 | 0.651 | (-32.44, 20.29) | 13.43 | -0.45 |
| m.7250A>G | 58.46 | 0.167 | (-24.44, 141.36) | 42.23 | 1.38 |
| m.7336C>Y | 6.64 | 0.873 | (-74.76, 88.04) | 41.46 | 0.16 |
| m.7444G>A | 13.59 | 0.818 | (-102.51, 129.70) | 59.14 | 0.23 |
| m.7600G>A | -20.28 | 0.279 | (-57.05, 16.49) | 18.73 | -1.08 |
| m.7684T>C | 10.05 | 0.563 | (-24.03, 44.13) | 17.36 | 0.58 |
| m.7828A>G | 24.43 | 0.355 | (-27.44, 76.30) | 26.42 | 0.92 |
| m.7853G>A | 7.78 | 0.593 | (-20.77, 36.33) | 14.54 | 0.53 |
| m.7861T>C | 68.38 | 0.382 | (-85.19, 221.96) | 78.22 | 0.87 |
| m.8020G>A | 10.26 | 0.679 | (-38.34, 58.85) | 24.75 | 0.41 |
| m.8149A>G | -23.46 | 0.452 | (-84.68, 37.77) | 31.19 | -0.75 |
| m.8414C>T | 5.13 | 0.732 | (-24.33, 34.60) | 15.01 | 0.34 |
| m.8473T>C | -22.10 | 0.709 | (-138.50, 94.29) | 59.29 | -0.37 |
| m.8563A>G | 40.00 | 0.171 | (-17.34, 97.34) | 29.21 | 1.37 |
| m.8584G>A | -4.00 | 0.725 | (-26.33, 18.33) | 11.37 | -0.35 |
| m.8684C>T | -50.94 | 0.110 | (-113.49, 11.62) | 31.86 | -1.60 |

| (Continue) **Supplementary Table 6**. Associations of mtDNA substitutions with pre-ART CD4+T cell counts in mtDNA genome-wide association analyses. | | | | | |
| --- | --- | --- | --- | --- | --- |
| Substitution | β | P | 95% CI | SE | t |

| m.8701A>G | -7.79 | 0.390 | (-25.57, 9.98) | 9.06 | -0.86 |
| --- | --- | --- | --- | --- | --- |
| m.8784A>G | 6.95 | 0.912 | (-116.37, 130.26) | 62.81 | 0.11 |
| m.8793T>C | 30.33 | 0.510 | (-60.07, 120.74) | 46.05 | 0.66 |
| m.8794C>T | 13.31 | 0.440 | (-20.51, 47.13) | 17.23 | 0.77 |
| m.8829C>T | 8.85 | 0.838 | (-76.26, 93.95) | 43.35 | 0.20 |
| m.8856G>A | 58.46 | 0.167 | (-24.44, 141.36) | 42.23 | 1.38 |
| m.8964C>T | 5.46 | 0.855 | (-52.99, 63.91) | 29.77 | 0.18 |
| m.9053G>A | 0.46 | 0.979 | (-33.85, 34.77) | 17.48 | 0.03 |
| m.9090T>C | 15.56 | 0.668 | (-55.53, 86.64) | 36.21 | 0.43 |
| m.9123G>A | 21.05 | 0.557 | (-49.27, 91.37) | 35.82 | 0.59 |
| m.9128T>C | -25.92 | 0.422 | (-89.33, 37.48) | 32.29 | -0.80 |
| m.9180A>G | -18.78 | 0.482 | (-71.24, 33.68) | 26.72 | -0.70 |
| m.9296C>T | -24.64 | 0.466 | (-91.03, 41.75) | 33.82 | -0.73 |
| m.9377A>G | -20.28 | 0.329 | (-61.03, 20.47) | 20.76 | -0.98 |
| m.9536C>T | -30.21 | 0.502 | (-118.54, 58.13) | 44.99 | -0.67 |
| m.9540T>C | -7.89 | 0.378 | (-25.46, 9.69) | 8.95 | -0.88 |
| m.9545A>G | 3.51 | 0.864 | (-36.57, 43.59) | 20.42 | 0.17 |
| m.9548G>A | -4.10 | 0.779 | (-32.76, 24.56) | 14.60 | -0.28 |
| m.9575G>A | -20.60 | 0.258 | (-56.33, 15.13) | 18.20 | -1.13 |
| m.9773C>Y | -21.51 | 0.515 | (-86.43, 43.40) | 33.06 | -0.65 |
| m.9814T>C | 13.83 | 0.664 | (-48.66, 76.32) | 31.83 | 0.43 |
| m.9824T>C | 10.24 | 0.714 | (-44.48, 64.95) | 27.87 | 0.37 |
| m.9950T>C | -3.86 | 0.877 | (-52.82, 45.10) | 24.94 | -0.15 |
| m.10208T>C | 15.00 | 0.733 | (-71.35, 101.35) | 43.98 | 0.34 |
| m.10238T>C | -73.43 | 0.230 | (-193.30, 46.44) | 61.05 | -1.20 |
| m.10310G>A | 6.20 | 0.680 | (-23.35, 35.75) | 15.05 | 0.41 |
| m.10325G>A | 29.10 | 0.536 | (-63.21, 121.42) | 47.02 | 0.62 |
| m.10397A>G | -17.56 | 0.473 | (-65.61, 30.50) | 24.48 | -0.72 |
| m.10398A>G | -8.16 | 0.345 | (-25.09, 8.78) | 8.63 | -0.95 |
| m.10399C>G | 9.76 | 0.481 | (-17.42, 36.94) | 13.84 | 0.71 |
| m.10400C>T | -7.55 | 0.411 | (-25.56, 10.46) | 9.17 | -0.82 |
| m.10454T>C | 23.92 | 0.647 | (-78.48, 126.33) | 52.16 | 0.46 |
| m.10535T>C | 23.12 | 0.495 | (-43.34, 89.57) | 33.85 | 0.68 |
| m.10586G>A | 24.43 | 0.355 | (-27.44, 76.30) | 26.42 | 0.92 |
| m.10609T>C | 9.62 | 0.581 | (-24.61, 43.84) | 17.43 | 0.55 |
| m.10646G>A | 30.33 | 0.458 | (-49.84, 110.50) | 40.84 | 0.74 |
| m.10873T>C | -7.89 | 0.386 | (-25.74, 9.97) | 9.09 | -0.87 |
| m.11215C>T | -35.54 | 0.336 | (-108.03, 36.96) | 36.93 | -0.96 |
| m.11440G>A | 20.00 | 0.456 | (-32.67, 72.67) | 26.83 | 0.75 |
| m.11536C>T | 40.36 | 0.175 | (-17.97, 98.69) | 29.71 | 1.36 |

| (Continue) **Supplementary Table 6**. Associations of mtDNA substitutions with pre-ART CD4+T cell counts in mtDNA genome-wide association analyses. | | | | | |
| --- | --- | --- | --- | --- | --- |
| Substitution | β | P | 95% CI | SE | t |

| m.11665C>T | -12.41 | 0.582 | (-56.61, 31.79) | 22.51 | -0.55 |
| --- | --- | --- | --- | --- | --- |
| m.11696G>A | -31.18 | 0.311 | (-91.52, 29.16) | 30.73 | -1.01 |
| m.11914G>A | 1.00 | 0.946 | (-27.79, 29.79) | 14.66 | 0.07 |
| m.11944T>C | -18.76 | 0.546 | (-79.73, 42.20) | 31.05 | -0.60 |
| m.11969G>A | -4.00 | 0.853 | (-46.49, 38.49) | 21.64 | -0.18 |
| m.12007G>A | -13.79 | 0.581 | (-62.87, 35.28) | 25.00 | -0.55 |
| m.12026A>G | -18.76 | 0.546 | (-79.73, 42.20) | 31.05 | -0.60 |
| m.12091T>C | -12.41 | 0.582 | (-56.61, 31.79) | 22.51 | -0.55 |
| m.12338T>C | 24.43 | 0.355 | (-27.44, 76.30) | 26.42 | 0.92 |
| m.12358A>G | 10.31 | 0.631 | (-31.77, 52.38) | 21.43 | 0.48 |
| m.12361A>G | 7.38 | 0.872 | (-82.66, 97.43) | 45.87 | 0.16 |
| m.12372G>A | -3.00 | 0.876 | (-40.60, 34.60) | 19.15 | -0.16 |
| m.12405C>T | 7.13 | 0.696 | (-28.66, 42.92) | 18.23 | 0.39 |
| m.12406G>A | 6.00 | 0.730 | (-28.14, 40.14) | 17.39 | 0.35 |
| m.12549C>T | 58.46 | 0.167 | (-24.44, 141.36) | 42.23 | 1.38 |
| m.12630G>A | 60.00 | 0.081 | (-7.46, 127.46) | 34.36 | 1.75 |
| m.12705C>T | 1.83 | 0.839 | (-15.91, 19.58) | 9.04 | 0.20 |
| m.12771G>A | 47.00 | 0.458 | (-77.33, 171.33) | 63.33 | 0.74 |
| m.12811T>C | 7.13 | 0.689 | (-27.83, 42.09) | 17.81 | 0.40 |
| m.12882C>T | 6.00 | 0.733 | (-28.55, 40.55) | 17.60 | 0.34 |
| m.12957T>C | 3.82 | 0.914 | (-65.62, 73.26) | 35.37 | 0.11 |
| m.13104A>G | 50.09 | 0.252 | (-35.74, 135.92) | 43.72 | 1.15 |
| m.13105A>G | 8.70 | 0.856 | (-85.24, 102.63) | 47.85 | 0.18 |
| m.13135G>A | 0.28 | 0.992 | (-56.14, 56.70) | 28.74 | 0.01 |
| m.13152A>G | 58.46 | 0.167 | (-24.44, 141.36) | 42.23 | 1.38 |
| m.13263A>G | 3.37 | 0.860 | (-34.24, 40.98) | 19.16 | 0.18 |
| m.13269A>G | -2.90 | 0.931 | (-68.91, 63.12) | 33.62 | -0.09 |
| m.13563A>G | -11.94 | 0.551 | (-51.22, 27.33) | 20.00 | -0.60 |
| m.13590G>A | -10.17 | 0.723 | (-66.38, 46.05) | 28.63 | -0.36 |
| m.13681A>G | -3.44 | 0.932 | (-82.13, 75.26) | 40.08 | -0.09 |
| m.13708G>A | 24.69 | 0.400 | (-32.86, 82.24) | 29.31 | 0.84 |
| m.13759G>A | 9.38 | 0.579 | (-23.78, 42.55) | 16.89 | 0.56 |
| m.13928G>C | 9.53 | 0.491 | (-17.61, 36.68) | 13.83 | 0.69 |
| m.14200T>C | -38.42 | 0.281 | (-108.28, 31.44) | -1.08 | 35.58 |
| m.14308T>C | -7.72 | 0.839 | (-82.16, 66.72) | -0.20 | 37.92 |
| m.14318T>C | 3.53 | 0.858 | (-35.30, 42.36) | 0.18 | 19.78 |
| m.14470T>C | -73.67 | 0.019 | (-135.40, -11.94) | -2.34 | 31.44 |
| m.14502T>C | 70.26 | 0.059 | (-2.68, 143.19) | 1.89 | 37.15 |
| m.14560G>A | 21.51 | 0.692 | (-84.91, 127.93) | 0.40 | 54.21 |
| m.14569G>A | -25.60 | 0.217 | (-66.27, 15.07) | -1.24 | 20.72 |

| (Continue) **Supplementary Table 6**. Associations of mtDNA substitutions with pre-ART CD4+T cell counts in mtDNA genome-wide association analyses. | | | | | |
| --- | --- | --- | --- | --- | --- |
| Substitution | β | P | 95% CI | SE | t |

| m.14587A>G | -70.33 | 0.390 | (-230.91, 90.25) | -0.86 | 81.79 |
| --- | --- | --- | --- | --- | --- |
| m.14668C>T | 5.13 | 0.732 | (-24.33, 34.60) | 0.34 | 15.01 |
| m.14783T>C | -7.79 | 0.381 | (-25.24, 9.65) | -0.88 | 8.89 |
| m.14861G>A | -33.43 | 0.398 | (-111.03, 44.17) | -0.85 | 39.52 |
| m.14978A>G | 53.15 | 0.099 | (-9.99, 116.29) | 1.65 | 32.16 |
| m.14979T>C | 14.18 | 0.783 | (-86.78, 115.14) | 0.28 | 51.43 |
| m.15024G>A | -35.38 | 0.640 | (-183.93, 113.16) | -0.47 | 75.66 |
| m.15040C>T | 58.46 | 0.167 | (-24.44, 141.36) | 1.38 | 42.23 |
| m.15043G>A | -8.23 | 0.356 | (-25.72, 9.26) | -0.92 | 8.91 |
| m.15071T>C | 58.46 | 0.167 | (-24.44, 141.36) | 1.38 | 42.23 |
| m.15218A>G | 70.26 | 0.057 | (-2.13, 142.64) | 1.91 | 36.87 |
| m.15223C>T | 7.38 | 0.865 | (-77.85, 92.62) | 0.17 | 43.41 |
| m.15235A>G | -21.83 | 0.549 | (-93.39, 49.72) | -0.60 | 36.45 |
| m.15236A>G | -17.64 | 0.556 | (-76.37, 41.09) | -0.59 | 29.91 |
| m.15301G>A | -8.21 | 0.353 | (-25.53, 9.11) | -0.93 | 8.82 |
| m.15323G>A | -64.33 | 0.063 | (-132.17, 3.50) | -1.86 | 34.55 |
| m.15326A>G | -16.62 | 0.551 | (-71.33, 38.10) | -0.60 | 27.87 |
| m.15346G>A | -0.82 | 0.966 | (-38.89, 37.25) | -0.04 | 19.39 |
| m.15487A>T | -3.97 | 0.765 | (-30.08, 22.14) | -0.30 | 13.30 |
| m.15508C>T | 7.38 | 0.865 | (-77.85, 92.62) | 0.17 | 43.41 |
| m.15535C>T | -10.00 | 0.694 | (-59.94, 39.94) | -0.39 | 25.43 |
| m.15662A>G | 7.38 | 0.865 | (-77.85, 92.62) | 0.17 | 43.41 |
| m.15724A>G | -26.74 | 0.591 | (-124.34, 70.85) | -0.54 | 49.71 |
| m.15784T>C | 47.65 | 0.201 | (-25.37, 120.68) | 1.28 | 37.19 |
| m.15851A>G | 6.95 | 0.861 | (-70.66, 84.55) | 0.18 | 39.53 |
| m.15924A>G | -1.54 | 0.976 | (-101.77, 98.69) | -0.03 | 51.05 |
| m.15927G>A | -3.10 | 0.941 | (-85.57, 79.36) | -0.07 | 42.00 |
| m.15930G>A | 1.54 | 0.977 | (-102.33, 105.40) | 0.03 | 52.90 |
| m.16051A>G | 16.62 | 0.771 | (-95.34, 128.57) | 0.29 | 57.03 |
| m.16086T>C | 64.54 | 0.124 | (-17.77, 146.84) | 1.54 | 41.92 |
| m.16092T>C | -25.93 | 0.413 | (-88.13, 36.27) | -0.82 | 31.68 |
| m.16093T>C | 3.37 | 0.833 | (-28.03, 34.78) | 0.21 | 16.00 |
| m.16111C>T | 8.38 | 0.650 | (-27.83, 44.60) | 0.45 | 18.45 |
| m.16126T>C | 40.00 | 0.215 | (-23.25, 103.25) | 1.24 | 32.22 |
| m.16129G>A | 6.21 | 0.504 | (-12.04, 24.46) | 0.67 | 9.30 |
| m.16136T>C | -10.17 | 0.705 | (-62.84, 42.50) | -0.38 | 26.83 |
| m.16140T>C | 4.93 | 0.755 | (-26.08, 35.94) | 0.31 | 15.79 |
| m.16162A>G | 0.46 | 0.980 | (-36.55, 37.48) | 0.02 | 18.85 |
| m.16164A>G | 1.36 | 0.978 | (-96.75, 99.47) | 0.03 | 49.97 |
| m.16172T>C | 16.13 | 0.347 | (-17.55, 49.81) | 0.94 | 17.15 |

| (Continue) **Supplementary Table 6**. Associations of mtDNA substitutions with pre-ART CD4+T cell counts in mtDNA genome-wide association analyses. | | | | | |
| --- | --- | --- | --- | --- | --- |
| Substitution | β | P | 95% CI | SE | t |

| m.16182A>C | 7.68 | 0.593 | (-20.50, 35.87) | 0.54 | 14.36 |
| --- | --- | --- | --- | --- | --- |
| m.16183A>C | -3.71 | 0.687 | (-21.80, 14.37) | -0.40 | 9.21 |
| m.16184C>T | -49.67 | 0.078 | (-104.92, 5.58) | -1.76 | 28.14 |
| m.16185C>T | 22.78 | 0.510 | (-45.02, 90.58) | 0.66 | 34.53 |
| m.16189T>C | -3.79 | 0.683 | (-22.02, 14.44) | -0.41 | 9.29 |
| m.16192C>T | -19.78 | 0.372 | (-63.25, 23.70) | -0.89 | 22.14 |
| m.16213G>A | -7.51 | 0.907 | (-134.38, 119.36) | -0.12 | 64.62 |
| m.16217T>C | -7.83 | 0.486 | (-29.87, 14.21) | -0.70 | 11.23 |
| m.16218C>T | -101.50 | 0.150 | (-239.66, 36.66) | -1.44 | 70.37 |
| m.16223C>T | 0.31 | 0.971 | (-16.88, 17.51) | 0.04 | 8.76 |
| m.16227A>G | -11.94 | 0.662 | (-65.51, 41.63) | -0.44 | 27.29 |
| m.16231T>C | -3.14 | 0.914 | (-60.57, 54.29) | -0.11 | 29.25 |
| m.16234C>T | -3.14 | 0.858 | (-37.65, 31.37) | -0.18 | 17.58 |
| m.16243T>C | 11.43 | 0.748 | (-58.33, 81.19) | 0.32 | 35.53 |
| m.16248C>T | 66.77 | 0.408 | (-91.59, 225.13) | 0.83 | 80.66 |
| m.16249T>C | 37.28 | 0.378 | (-45.75, 120.32) | 0.88 | 42.29 |
| m.16256C>T | -21.51 | 0.664 | (-118.63, 75.60) | -0.43 | 49.47 |
| m.16257C>A | 2.28 | 0.912 | (-38.28, 42.85) | 0.11 | 20.66 |
| m.16260C>T | -3.59 | 0.857 | (-42.60, 35.42) | -0.18 | 19.87 |
| m.16261C>T | 10.50 | 0.415 | (-14.75, 35.75) | 0.82 | 12.86 |
| m.16266C>A | -2.87 | 0.943 | (-82.04, 76.30) | -0.07 | 40.32 |
| m.16266C>T | -17.56 | 0.443 | (-62.48, 27.37) | -0.77 | 22.88 |
| m.16274G>A | 6.85 | 0.700 | (-28.02, 41.71) | 0.39 | 17.76 |
| m.16278C>T | -26.21 | 0.213 | (-67.47, 15.05) | -1.25 | 21.02 |
| m.16284A>G | 9.08 | 0.734 | (-43.37, 61.53) | 0.34 | 26.72 |
| m.16290C>T | 12.85 | 0.463 | (-21.49, 47.19) | 0.73 | 17.49 |
| m.16291C>T | -25.44 | 0.419 | (-87.26, 36.39) | -0.81 | 31.49 |
| m.16295C>T | -24.00 | 0.192 | (-60.11, 12.11) | -1.30 | 18.39 |
| m.16297T>C | 7.13 | 0.702 | (-29.38, 43.64) | 0.38 | 18.60 |
| m.16298T>C | 1.00 | 0.935 | (-22.92, 24.92) | 0.08 | 12.18 |
| m.16299A>G | 21.51 | 0.734 | (-102.73, 145.75) | 0.34 | 63.28 |
| m.16300A>G | 12.59 | 0.765 | (-70.14, 95.32) | 0.30 | 42.14 |
| m.16302A>C | 7.30 | 0.680 | (-27.42, 42.02) | 0.41 | 17.69 |
| m.16304T>C | 13.00 | 0.433 | (-19.53, 45.53) | 0.78 | 16.57 |
| m.16311T>C | 10.53 | 0.432 | (-15.77, 36.83) | 0.79 | 13.40 |
| m.16316A>G | -21.39 | 0.339 | (-65.24, 22.46) | -0.96 | 22.34 |
| m.16319G>A | 1.33 | 0.920 | (-24.66, 27.32) | 0.10 | 13.24 |
| m.16325T>C | 18.97 | 0.861 | (-193.30, 231.25) | 0.18 | 108.12 |
| m.16327C>T | 3.37 | 0.856 | (-33.17, 39.91) | 0.18 | 18.61 |
| m.16335A>G | 25.15 | 0.350 | (-27.63, 77.94) | 0.94 | 26.89 |

| (Continue) **Supplementary Table 6**. Associations of mtDNA substitutions with pre-ART CD4+T cell counts in mtDNA genome-wide association analyses. | | | | | |
| --- | --- | --- | --- | --- | --- |
| Substitution | β | P | 95% CI | SE | t |

| m.16355C>T | 72.72 | 0.186 | (-35.25, 180.68) | 1.32 | 54.99 |
| --- | --- | --- | --- | --- | --- |
| m.16357T>C | -3.28 | 0.904 | (-56.76, 50.19) | -0.12 | 27.24 |
| m.16360C>T | -33.63 | 0.525 | (-137.45, 70.19) | -0.64 | 52.88 |
| m.16362T>C | -24.29 | 0.016 | (-43.94, -4.63) | -2.43 | 10.01 |
| m.16390G>A | -4.56 | 0.874 | (-61.15, 52.02) | -0.16 | 28.82 |
| m.16399A>G | -1.79 | 0.971 | (-99.29, 95.70) | -0.04 | 49.66 |
| m.16519T>C | 8.92 | 0.307 | (-8.22, 26.06) | 1.02 | 8.73 |

β, slope; 95% CI, 95% confidence interval; SE, standard error
