# Supplementary material for: Baseline Characteristics of Mitochondrial DNA and Mutations Associated With Short-Term Posttreatment CD4+T-Cell Recovery in Chinese People With HIV
Source: Front Immunol. 2021 Dec 14;12:793375. doi: 10.3389/fimmu.2021.793375 (PMC8712318; doi:10.3389/fimmu.2021.793375)
Supplement: Supplementary file 1 [file DataSheet_1.zip › SupplementaryMaterial/Supplementary Table7.docx]

| **Supplementary Table 7**. Associations of mtDNA substitutions with changes from pre-ART to post-ART in the CD4+T cell count in mtDNA genome-wide association analyses. | | | | | |
| --- | --- | --- | --- | --- | --- |
| Substitution | β | P | 95% CI | SE | t |

| m.93A>G | 49.56 | 0.003 | (17.08, 82.05) | 16.55 | 3.00 |
| --- | --- | --- | --- | --- | --- |
| m.103G>A | -16.76 | 0.577 | (-75.70, 42.18) | 30.02 | -0.56 |
| m.143G>A | 1.23 | 0.977 | (-80.90, 83.36) | 41.83 | 0.03 |
| m.146T>C | 10.80 | 0.362 | (-12.43, 34.02) | 11.83 | 0.91 |
| m.150C>T | -2.53 | 0.811 | (-23.26, 18.20) | 10.56 | -0.24 |
| m.151C>T | 36.88 | 0.320 | (-35.94, 109.70) | 37.09 | 0.99 |
| m.152T>C | -4.26 | 0.663 | (-23.45, 14.92) | 9.77 | -0.44 |
| m.153A>G | -17.09 | 0.427 | (-59.25, 25.08) | 21.48 | -0.80 |
| m.185G>A | 7.41 | 0.907 | (-117.41, 132.23) | 63.58 | 0.12 |
| m.189A>G | 30.66 | 0.623 | (-91.76, 153.07) | 62.35 | 0.49 |
| m.194C>T | 42.40 | 0.397 | (-55.79, 140.59) | 50.01 | 0.85 |
| m.195T>C | 11.63 | 0.380 | (-14.33, 37.58) | 13.22 | 0.88 |
| m.199T>C | 6.83 | 0.703 | (-28.28, 41.94) | 17.88 | 0.38 |
| m.200A>G | -4.01 | 0.887 | (-59.58, 51.55) | 28.30 | -0.14 |
| m.204T>C | -18.13 | 0.379 | (-58.53, 22.28) | 20.58 | -0.88 |
| m.207G>A | 0.80 | 0.972 | (-43.22, 44.82) | 22.42 | 0.04 |
| m.210A>G | -2.42 | 0.915 | (-46.74, 41.90) | 22.57 | -0.11 |
| m.215A>G | 49.87 | 0.172 | (-21.81, 121.54) | 36.51 | 1.37 |
| m.217T>C | -10.28 | 0.871 | (-134.44, 113.88) | 63.24 | -0.16 |
| m.235A>G | 13.08 | 0.430 | (-19.46, 45.61) | 16.57 | 0.79 |
| m.248A>G | -40.27 | 0.265 | (-111.17, 30.62) | 36.11 | -1.12 |
| m.263A>G | -40.95 | 0.262 | (-112.51, 30.60) | 36.45 | -1.12 |
| m.298C>T | -43.31 | 0.304 | (-125.99, 39.36) | 42.11 | -1.03 |
| m.310T>C | -4.85 | 0.618 | (-23.93, 14.24) | 9.72 | -0.50 |
| m.318T>C | 70.46 | 0.164 | (-28.81, 169.73) | 50.56 | 1.39 |
| m.456C>T | 8.42 | 0.837 | (-71.80, 88.64) | 40.86 | 0.21 |
| m.489T>C | 0.57 | 0.948 | (-16.65, 17.78) | 8.77 | 0.06 |
| m.499G>A | 24.95 | 0.332 | (-25.47, 75.36) | 25.68 | 0.97 |
| m.663A>G | 10.17 | 0.570 | (-24.93, 45.28) | 17.88 | 0.57 |
| m.681T>C | 41.09 | 0.473 | (-71.35, 153.54) | 57.27 | 0.72 |
| m.709G>A | -2.08 | 0.848 | (-23.38, 19.21) | 10.85 | -0.19 |
| m.735A>G | 64.29 | 0.175 | (-28.61, 157.19) | 47.32 | 1.36 |
| m.752C>T | -12.37 | 0.616 | (-60.77, 36.04) | 24.65 | -0.50 |
| m.827A>G | 25.66 | 0.239 | (-17.05, 68.38) | 21.76 | 1.18 |
| m.961T>C | -41.69 | 0.302 | (-120.92, 37.55) | 40.36 | -1.03 |
| m.1005T>C | -50.97 | 0.005 | (-86.79, -15.14) | 18.25 | -2.79 |
| m.1048C>T | -20.44 | 0.699 | (-124.27, 83.40) | 52.89 | -0.39 |
| m.1107T>C | 2.51 | 0.912 | (-42.03, 47.04) | 22.68 | 0.11 |
| m.1119T>C | 7.22 | 0.700 | (-29.59, 44.03) | 18.75 | 0.39 |

| (Continue) **Supplementary Table 7**. Associations of mtDNA substitutions with changes from pre-ART to post-ART in the CD4+T cell count in mtDNA genome-wide association analyses. | | | | | |
| --- | --- | --- | --- | --- | --- |
| Substitution | β | P | 95% CI | SE | t |

| m.1382A>C | 9.37 | 0.832 | (-77.22, 95.96) | 44.10 | 0.21 |
| --- | --- | --- | --- | --- | --- |
| m.1438A>G | 11.27 | 0.707 | (-47.63, 70.17) | 30.00 | 0.38 |
| m.1598G>A | 8.65 | 0.793 | (-56.22, 73.53) | 33.05 | 0.26 |
| m.1709G>A | -9.34 | 0.839 | (-99.64, 80.97) | 46.00 | -0.20 |
| m.1719G>A | 41.82 | 0.291 | (-35.87, 119.52) | 39.57 | 1.06 |
| m.1736A>G | 14.84 | 0.441 | (-22.94, 52.63) | 19.25 | 0.77 |
| m.1824T>C | -44.14 | 0.025 | (-82.59, -5.69) | 19.59 | -2.25 |
| m.2835C>T | 14.83 | 0.566 | (-35.93, 65.59) | 25.85 | 0.57 |
| m.3010G>A | 2.14 | 0.859 | (-21.45, 25.73) | 12.02 | 0.18 |
| m.3206C>T | 11.79 | 0.771 | (-67.88, 91.46) | 40.58 | 0.29 |
| m.3316G>A | 69.14 | 0.153 | (-25.69, 163.97) | 48.30 | 1.43 |
| m.3394T>C | -41.05 | 0.006 | (-70.32, -11.78) | 14.91 | -2.75 |
| m.3398T>C | -71.88 | 0.082 | (-152.83, 9.08) | 41.23 | -1.74 |
| m.3483G>A | 4.01 | 0.907 | (-63.42, 71.45) | 34.35 | 0.12 |
| m.3497C>T | 7.56 | 0.729 | (-35.24, 50.36) | 21.80 | 0.35 |
| m.3537A>G | -4.60 | 0.825 | (-45.50, 36.29) | 20.83 | -0.22 |
| m.3552T>A | 26.99 | 0.323 | (-26.55, 80.52) | 27.27 | 0.99 |
| m.3571C>T | -3.52 | 0.888 | (-52.55, 45.50) | 24.97 | -0.14 |
| m.3606A>G | -68.19 | 0.160 | (-163.40, 27.03) | 48.50 | -1.41 |
| m.3834G>A | 42.63 | 0.167 | (-17.81, 103.08) | 30.79 | 1.38 |
| m.3882G>A | 5.96 | 0.886 | (-75.57, 87.48) | 41.52 | 0.14 |
| m.3970C>T | -11.36 | 0.315 | (-33.55, 10.82) | 11.30 | -1.01 |
| m.4048G>A | -3.25 | 0.884 | (-47.16, 40.65) | 22.36 | -0.15 |
| m.4071C>T | 3.50 | 0.854 | (-33.93, 40.92) | 19.06 | 0.18 |
| m.4086C>T | 2.05 | 0.895 | (-28.45, 32.55) | 15.53 | 0.13 |
| m.4140C>T | 92.11 | 0.113 | (-21.78, 205.99) | 58.01 | 1.59 |
| m.4164A>G | 3.50 | 0.874 | (-39.83, 46.83) | 22.07 | 0.16 |
| m.4216T>C | 23.78 | 0.562 | (-56.66, 104.23) | 40.97 | 0.58 |
| m.4248T>C | 10.17 | 0.570 | (-24.93, 45.28) | 17.88 | 0.57 |
| m.4386T>C | 14.18 | 0.634 | (-44.35, 72.72) | 29.81 | 0.48 |
| m.4491G>A | -47.81 | 0.013 | (-85.56, -10.07) | 19.22 | -2.49 |
| m.4715A>G | -1.22 | 0.939 | (-32.66, 30.23) | 16.02 | -0.08 |
| m.4820G>A | 24.95 | 0.332 | (-25.47, 75.36) | 25.68 | 0.97 |
| m.4824A>G | 5.31 | 0.743 | (-26.48, 37.10) | 16.19 | 0.33 |
| m.4833A>G | -30.66 | 0.080 | (-64.95, 3.63) | 17.47 | -1.76 |
| m.4850C>T | 5.96 | 0.888 | (-76.86, 88.77) | 42.18 | 0.14 |
| m.4883C>T | 2.66 | 0.798 | (-17.78, 23.11) | 10.41 | 0.26 |
| m.5108T>C | -30.28 | 0.055 | (-61.26, 0.69) | 15.78 | -1.92 |
| m.5147G>A | -5.73 | 0.868 | (-73.21, 61.75) | 34.37 | -0.17 |

| (Continue) **Supplementary Table 7**. Associations of mtDNA substitutions with changes from pre-ART to post-ART in the CD4+T cell count in mtDNA genome-wide association analyses. | | | | | |
| --- | --- | --- | --- | --- | --- |
| Substitution | β | P | 95% CI | SE | t |

| m.5153A>G | 41.09 | 0.473 | (-71.35, 153.54) | 57.27 | 0.72 |
| --- | --- | --- | --- | --- | --- |
| m.5178C>A | 2.25 | 0.830 | (-18.32, 22.83) | 10.48 | 0.21 |
| m.5231G>A | -2.82 | 0.865 | (-35.53, 29.88) | 16.66 | -0.17 |
| m.5301A>G | -11.20 | 0.617 | (-55.09, 32.70) | 22.36 | -0.50 |
| m.5351A>G | 3.50 | 0.874 | (-39.83, 46.83) | 22.07 | 0.16 |
| m.5417G>A | -2.82 | 0.871 | (-36.99, 31.34) | 17.40 | -0.16 |
| m.5442T>C | 5.15 | 0.903 | (-77.65, 87.95) | 42.17 | 0.12 |
| m.5460G>A | -2.03 | 0.909 | (-36.74, 32.69) | 17.68 | -0.11 |
| m.5465T>C | 0.87 | 0.983 | (-79.52, 81.26) | 40.94 | 0.02 |
| m.5585G>A | 22.92 | 0.750 | (-118.30, 164.15) | 71.93 | 0.32 |
| m.5587T>C | 17.77 | 0.731 | (-83.83, 119.38) | 51.75 | 0.34 |
| m.5601C>T | -37.29 | 0.195 | (-93.76, 19.17) | 28.76 | -1.30 |
| m.5628T>C | 50.83 | 0.364 | (-59.04, 160.70) | 55.96 | 0.91 |
| m.5821G>A | 26.99 | 0.393 | (-35.06, 89.04) | 31.60 | 0.85 |
| m.6023G>A | 25.47 | 0.502 | (-49.03, 99.97) | 37.95 | 0.67 |
| m.6179G>A | 14.83 | 0.397 | (-19.50, 49.16) | 17.49 | 0.85 |
| m.6216T>C | 24.37 | 0.515 | (-49.03, 97.77) | 37.38 | 0.65 |
| m.6253T>C | 45.37 | 0.541 | (-100.18, 190.92) | 74.14 | 0.61 |
| m.6392T>C | -9.35 | 0.436 | (-32.91, 14.22) | 12.00 | -0.78 |
| m.6413T>C | 33.23 | 0.390 | (-42.64, 109.09) | 38.64 | 0.86 |
| m.6455C>T | 3.50 | 0.854 | (-33.93, 40.92) | 19.06 | 0.18 |
| m.6599A>G | -43.59 | 0.110 | (-97.05, 9.87) | 27.23 | -1.60 |
| m.6680T>C | 1.32 | 0.949 | (-39.26, 41.90) | 20.67 | 0.06 |
| m.6752A>G | -40.41 | 0.237 | (-107.39, 26.57) | 34.12 | -1.18 |
| m.6960C>T | -24.42 | 0.249 | (-66.02, 17.17) | 21.19 | -1.15 |
| m.6962G>A | -1.69 | 0.882 | (-24.04, 20.66) | 11.38 | -0.15 |
| m.7196C>A | -1.22 | 0.939 | (-32.68, 30.25) | 16.03 | -0.08 |
| m.7250A>G | 92.11 | 0.113 | (-21.78, 205.99) | 58.01 | 1.59 |
| m.7336C>Y | -43.18 | 0.149 | (-101.87, 15.51) | 29.90 | -1.44 |
| m.7444G>A | -29.93 | 0.687 | (-175.73, 115.87) | 74.26 | -0.40 |
| m.7600G>A | -37.93 | 0.291 | (-108.33, 32.48) | 35.86 | -1.06 |
| m.7684T>C | 5.77 | 0.771 | (-33.19, 44.73) | 19.85 | 0.29 |
| m.7828A>G | -44.14 | 0.025 | (-82.59, -5.69) | 19.59 | -2.25 |
| m.7853G>A | 3.59 | 0.861 | (-36.62, 43.79) | 20.48 | 0.18 |
| m.7861T>C | 9.37 | 0.806 | （-65.39, 84.13） | 38.08 | 0.25 |
| m.8020G>A | -3.76 | 0.935 | (-94.41, 86.69) | 46.17 | -0.08 |
| m.8149A>G | -0.50 | 0.967 | (-24.64, 23.69) | 12.29 | -0.04 |
| m.8414C>T | -9.18 | 0.850 | (-104.32, 85.69) | 48.46 | -0.19 |
| m.8473T>C | -41.69 | 0.382 | (-135.23, 51.69) | 47.65 | -0.87 |

| (Continue) **Supplementary Table 7**. Associations of mtDNA substitutions with changes from pre-ART to post-ART in the CD4+T cell count in mtDNA genome-wide association analyses. | | | | | |
| --- | --- | --- | --- | --- | --- |
| Substitution | β | P | 95% CI | SE | t |

| m.8563A>G | 0.74 | 0.954 | （-24.46, 25.95） | 12.84 | 0.06 |
| --- | --- | --- | --- | --- | --- |
| m.8584G>A | 14.83 | 0.397 | （-19.50, 49.16） | 17.49 | 0.85 |
| m.8684C>T | 2.22 | 0.800 | （-14.99, 19.43） | 8.77 | 0.25 |
| m.8701A>G | 8.65 | 0.899 | （-125.65, 142.96） | 68.41 | 0.13 |
| m.8784A>G | 14.03 | 0.758 | （-75.46, 103.52） | 45.58 | 0.31 |
| m.8793T>C | 10.17 | 0.570 | （-24.93, 45.28） | 17.88 | 0.57 |
| m.8794C>T | 8.33 | 0.785 | （-51.52, 68.18） | 30.48 | 0.27 |
| m.8829C>T | 9.37 | 0.806 | （-65.39, 84.13） | 38.08 | 0.25 |
| m.8856G>A | 92.11 | 0.113 | （-21.78, 205.99） | 58.01 | 1.59 |
| m.8964C>T | 9.37 | 0.832 | （-77.22, 95.96） | 44.10 | 0.21 |
| m.9053G>A | -2.16 | 0.842 | (-23.53, 19.69) | 10.88 | -0.20 |
| m.9090T>C | -40.48 | 0.311 | (-118.82, 37.69) | 39.90 | -1.01 |
| m.9123G>A | 0.87 | 0.983 | （-79.52, 81.26） | 40.94 | 0.02 |
| m.9128T>C | -9.07 | 0.815 | (-85.21, 67.69) | 38.78 | -0.23 |
| m.9180A>G | -12.37 | 0.599 | (-58.53, 33.69) | 23.51 | -0.53 |
| m.9296C>T | -36.23 | 0.405 | (-121.66, 49.69) | 43.51 | -0.83 |
| m.9377A>G | -29.20 | 0.476 | (-109.59, 51.69) | 40.95 | -0.71 |
| m.9536C>T | 31.30 | 0.589 | （-82.42, 145.03） | 57.93 | 0.54 |
| m.9540T>C | 2.22 | 0.800 | （-14.96, 19.40） | 8.75 | 0.25 |
| m.9545A>G | 3.68 | 0.901 | （-54.21, 61.56） | 29.48 | 0.12 |
| m.9548G>A | 2.30 | 0.894 | （-31.65, 36.26） | 17.30 | 0.13 |
| m.9575G>A | -37.50 | 0.346 | (-115.58, 40.69) | 39.77 | -0.94 |
| m.9773C>Y | -35.68 | 0.061 | (-72.98, 1.69) | 19.00 | -1.88 |
| m.9814T>C | -43.49 | 0.008 | (-75.43, -11.69) | 16.27 | -2.67 |
| m.9824T>C | -10.31 | 0.811 | (-94.74, 74.69) | 43.01 | -0.24 |
| m.9950T>C | 0.84 | 0.963 | （-34.31, 35.99） | 17.90 | 0.05 |
| m.10208T>C | 28.07 | 0.651 | （-93.80, 149.94） | 62.07 | 0.45 |
| m.10238T>C | 65.72 | 0.389 | （-83.90, 215.34） | 76.21 | 0.86 |
| m.10310G>A | -11.36 | 0.327 | (-34.12, 11.69) | 11.59 | -0.98 |
| m.10325G>A | -22.55 | 0.426 | (-78.20, 33.69) | 28.34 | -0.80 |
| m.10397A>G | 2.51 | 0.912 | （-41.79, 46.80） | 22.56 | 0.11 |
| m.10398A>G | 0.59 | 0.946 | （-16.63, 17.81） | 8.77 | 0.07 |
| m.10399C>G | 14.65 | 0.455 | （-23.81, 53.10） | 19.59 | 0.75 |
| m.10400C>T | 2.22 | 0.799 | （-14.91, 19.35） | 8.72 | 0.25 |
| m.10454T>C | -6.87 | 0.824 | (-67.63, 53.69) | 30.94 | -0.22 |
| m.10535T>C | -37.65 | 0.128 | (-86.14, 10.69) | 24.70 | -1.52 |
| m.10586G>A | -44.14 | 0.025 | (-82.59, -5.69) | 19.59 | -2.25 |
| m.10609T>C | -1.69 | 0.882 | (-24.04, 20.69) | 11.38 | -0.15 |
| m.10646G>A | 30.30 | 0.561 | （-72.00, 132.59） | 52.10 | 0.58 |

| (Continue) **Supplementary Table 7**. Associations of mtDNA substitutions with changes from pre-ART to post-ART in the CD4+T cell count in mtDNA genome-wide association analyses. | | | | | |
| --- | --- | --- | --- | --- | --- |
| Substitution | β | P | 95% CI | SE | t |

| m.10873T>C | -0.49 | 0.955 | (-17.63, 16.69) | 8.73 | -0.06 |
| --- | --- | --- | --- | --- | --- |
| m.11215C>T | -6.30 | 0.910 | (-115.06, 102.69) | 55.40 | -0.11 |
| m.11440G>A | -15.31 | 0.702 | (-93.82, 63.69) | 39.99 | -0.38 |
| m.11536C>T | -12.78 | 0.790 | (-106.93, 81.69) | 47.96 | -0.27 |
| m.11665C>T | 5.96 | 0.888 | （-76.86, 88.77） | 42.18 | 0.14 |
| m.11696G>A | -0.50 | 0.985 | (-52.52, 51.69) | 26.50 | -0.02 |
| m.11914G>A | 12.59 | 0.525 | （-26.30, 51.47） | 19.81 | 0.64 |
| m.11944T>C | -12.37 | 0.616 | (-60.77, 36.69) | 24.65 | -0.50 |
| m.11969G>A | 46.74 | 0.117 | （-11.72, 105.21） | 29.78 | 1.57 |
| m.12007G>A | 19.47 | 0.554 | （-45.18, 84.13） | 32.93 | 0.59 |
| m.12026A>G | -12.37 | 0.616 | (-60.77, 36.69) | 24.65 | -0.50 |
| m.12091T>C | 5.96 | 0.888 | （-76.86, 88.77） | 42.18 | 0.14 |
| m.12338T>C | -44.14 | 0.025 | (-82.59, -5.69) | 19.59 | -2.25 |
| m.12358A>G | -3.44 | 0.842 | (-37.30, 30.69) | 17.25 | -0.20 |
| m.12361A>G | 8.65 | 0.774 | （-50.54, 67.84） | 30.15 | 0.29 |
| m.12372G>A | -2.82 | 0.881 | (-39.74, 34.69) | 18.80 | -0.15 |
| m.12405C>T | 3.50 | 0.874 | （-39.83, 46.83） | 22.07 | 0.16 |
| m.12406G>A | -1.64 | 0.884 | (-23.70, 20.69) | 11.23 | -0.15 |
| m.12549C>T | 92.11 | 0.113 | （-21.78, 205.99） | 58.01 | 1.59 |
| m.12630G>A | 3.95 | 0.931 | （-85.10, 93.00） | 45.36 | 0.09 |
| m.12705C>T | 2.32 | 0.807 | （-16.30, 20.93） | 9.48 | 0.24 |
| m.12771G>A | -16.59 | 0.589 | (-76.89, 43.69) | 30.71 | -0.54 |
| m.12811T>C | -3.25 | 0.881 | (-45.91, 39.69) | 21.72 | -0.15 |
| m.12882C>T | -2.16 | 0.850 | (-24.66, 20.69) | 11.46 | -0.19 |
| m.12957T>C | 52.52 | 0.402 | （-70.42, 175.45） | 62.62 | 0.84 |
| m.13104A>G | -43.31 | 0.304 | (-125.99, 39.69) | 42.11 | -1.03 |
| m.13105A>G | 7.56 | 0.764 | （-41.84, 56.96） | 25.16 | 0.30 |
| m.13135G>A | 50.45 | 0.189 | （-24.88, 125.77） | 38.36 | 1.31 |
| m.13152A>G | 92.11 | 0.113 | （-21.78, 205.99） | 58.01 | 1.59 |
| m.13263A>G | 3.68 | 0.896 | （-51.70, 59.05） | 28.21 | 0.13 |
| m.13269A>G | 26.19 | 0.776 | （-154.70, 207.08） | 92.14 | 0.28 |
| m.13563A>G | -36.65 | 0.259 | (-100.34, 27.69) | 32.44 | -1.13 |
| m.13590G>A | 24.95 | 0.323 | （-24.52, 74.42） | 25.20 | 0.99 |
| m.13681A>G | 48.88 | 0.350 | （-53.79, 151.55） | 52.30 | 0.93 |
| m.13708G>A | -38.73 | 0.035 | (-74.72, -2.69) | 18.33 | -2.11 |
| m.13759G>A | -5.07 | 0.648 | (-26.87, 16.69) | 11.10 | -0.46 |
| m.13928G>C | -17.69 | 0.133 | (-40.80, 5.69) | 11.77 | -1.50 |
| m.14200T>C | -43.46 | 0.218 | (-112.61, 25.69) | -1.23 | 35.22 |
| m.14308T>C | -56.47 | 8.22E-05 | (-84.46, -28.48) | -3.96 | 14.26 |

| (Continue) **Supplementary Table 7**. Associations of mtDNA substitutions with changes from pre-ART to post-ART in the CD4+T cell count in mtDNA genome-wide association analyses. | | | | | |
| --- | --- | --- | --- | --- | --- |
| Substitution | β | P | 95% CI | SE | t |

| m.14318T>C | 3.68 | 0.903 | (-55.72, 63.08) | 0.12 | 30.26 |
| --- | --- | --- | --- | --- | --- |
| m.14470T>C | 7.00 | 0.717 | (-30.86, 44.86) | 0.36 | 19.28 |
| m.14502T>C | 92.72 | 0.111 | (-21.22, 206.67) | 1.60 | 58.04 |
| m.14560G>A | 47.22 | 0.327 | (-47.27, 141.71) | 0.98 | 48.13 |
| m.14569G>A | -28.91 | 0.093 | (-62.64, 4.81) | -1.68 | 17.18 |
| m.14587A>G | 16.24 | 0.781 | (-98.28, 130.76) | 0.28 | 58.33 |
| m.14668C>T | -0.50 | 0.967 | (-24.68, 23.68) | -0.04 | 12.32 |
| m.14783T>C | -0.49 | 0.956 | (-17.71, 16.73) | -0.06 | 8.77 |
| m.14861G>A | -11.86 | 0.775 | (-93.28, 69.56) | -0.29 | 41.47 |
| m.14978A>G | 24.14 | 0.540 | (-53.18, 101.46) | 0.61 | 39.38 |
| m.14979T>C | 11.79 | 0.771 | (-67.72, 91.31) | 0.29 | 40.50 |
| m.15024G>A | 17.77 | 0.733 | (-84.37, 119.92) | 0.34 | 52.03 |
| m.15040C>T | 92.11 | 0.113 | (-21.78, 205.99) | 1.59 | 58.01 |
| m.15043G>A | -1.11 | 0.899 | (-18.21, 15.99) | -0.13 | 8.71 |
| m.15071T>C | 92.11 | 0.113 | (-21.78, 205.99) | 1.59 | 58.01 |
| m.15218A>G | 92.88 | 0.056 | (-2.24, 188.00) | 1.92 | 48.45 |
| m.15223C>T | 8.65 | 0.793 | (-56.07, 73.38) | 0.26 | 32.97 |
| m.15235A>G | -4.60 | 0.826 | (-45.59, 36.38) | -0.22 | 20.88 |
| m.15236A>G | -5.01 | 0.906 | (-88.16, 78.14) | -0.12 | 42.35 |
| m.15301G>A | -4.85 | 0.575 | (-21.82, 12.11) | -0.56 | 8.64 |
| m.15323G>A | -13.53 | 0.600 | (-64.10, 37.04) | -0.53 | 25.76 |
| m.15326A>G | -30.16 | 0.396 | (-99.91, 39.59) | -0.85 | 35.53 |
| m.15346G>A | 7.22 | 0.701 | (-29.66, 44.10) | 0.38 | 18.78 |
| m.15487A>T | -1.22 | 0.940 | (-32.71, 30.27) | -0.08 | 16.04 |
| m.15508C>T | 8.65 | 0.793 | (-56.07, 73.38) | 0.26 | 32.97 |
| m.15535C>T | 27.65 | 0.173 | (-12.16, 67.46) | 1.36 | 20.28 |
| m.15662A>G | 8.65 | 0.793 | (-56.07, 73.38) | 0.26 | 32.97 |
| m.15724A>G | 46.10 | 0.447 | (-72.97, 165.17) | 0.76 | 60.65 |
| m.15784T>C | -35.16 | 0.332 | (-106.26, 35.93) | -0.97 | 36.21 |
| m.15851A>G | 8.33 | 0.763 | (-45.84, 62.51) | 0.30 | 27.59 |
| m.15924A>G | 70.49 | 0.030 | (6.86, 134.11) | 2.17 | 32.41 |
| m.15927G>A | 13.00 | 0.549 | (-29.57, 55.57) | 0.60 | 21.68 |
| m.15930G>A | 19.77 | 0.628 | (-60.37, 99.91) | 0.48 | 40.82 |
| m.16051A>G | 24.68 | 0.216 | (-14.41, 63.78) | 1.24 | 19.91 |
| m.16086T>C | 19.71 | 0.669 | (-70.77, 110.19) | 0.43 | 46.08 |
| m.16092T>C | -3.41 | 0.899 | (-55.85, 49.04) | -0.13 | 26.71 |
| m.16093T>C | 13.95 | 0.453 | (-22.55, 50.44) | 0.75 | 18.59 |
| m.16111C>T | -2.14 | 0.916 | (-41.93, 37.66) | -0.11 | 20.27 |
| m.16126T>C | -8.79 | 0.778 | (-69.89, 52.32) | -0.28 | 31.12 |

| (Continue) **Supplementary Table 7**. Associations of mtDNA substitutions with changes from pre-ART to post-ART in the CD4+T cell count in mtDNA genome-wide association analyses. | | | | | |
| --- | --- | --- | --- | --- | --- |
| Substitution | β | P | 95% CI | SE | t |

| m.16129G>A | 5.85 | 0.518 | (-11.91, 23.61) | 0.65 | 9.04 |
| --- | --- | --- | --- | --- | --- |
| m.16136T>C | 28.39 | 0.187 | (-13.84, 70.61) | 1.32 | 21.51 |
| m.16140T>C | -3.49 | 0.811 | (-32.06, 25.08) | -0.24 | 14.55 |
| m.16162A>G | 2.30 | 0.901 | (-33.93, 38.53) | 0.12 | 18.45 |
| m.16164A>G | 10.51 | 0.821 | (-80.87, 101.88) | 0.23 | 46.54 |
| m.16172T>C | -2.53 | 0.842 | (-27.36, 22.30) | -0.20 | 12.65 |
| m.16182A>C | -1.38 | 0.914 | (-26.62, 23.85) | -0.11 | 12.85 |
| m.16183A>C | 2.39 | 0.814 | (-17.54, 22.33) | 0.24 | 10.15 |
| m.16184C>T | 10.49 | 0.513 | (-20.96, 41.94) | 0.65 | 16.02 |
| m.16185C>T | -41.59 | 0.146 | (-97.66, 14.48) | -1.46 | 28.56 |
| m.16189T>C | 5.55 | 0.526 | (-11.62, 22.72) | 0.63 | 8.75 |
| m.16192C>T | 21.46 | 0.341 | (-22.76, 65.68) | 0.95 | 22.52 |
| m.16213G>A | 24.88 | 0.592 | (-66.26, 116.02) | 0.54 | 46.42 |
| m.16217T>C | 10.67 | 0.380 | (-13.18, 34.52) | 0.88 | 12.15 |
| m.16218C>T | -26.18 | 0.478 | (-98.52, 46.17) | -0.71 | 36.85 |
| m.16223C>T | 3.19 | 0.748 | (-16.28, 22.66) | 0.32 | 9.92 |
| m.16227A>G | 43.50 | 0.448 | (-69.05, 156.06) | 0.76 | 57.33 |
| m.16231T>C | 46.05 | 0.276 | (-36.80, 128.91) | 1.09 | 42.20 |
| m.16234C>T | -22.98 | 0.144 | (-53.83, 7.87) | -1.46 | 15.71 |
| m.16243T>C | 8.31 | 0.760 | (-45.05, 61.68) | 0.31 | 27.18 |
| m.16248C>T | -7.08 | 0.812 | (-65.53, 51.38) | -0.24 | 29.78 |
| m.16249T>C | -13.24 | 0.688 | (-77.94, 51.47) | -0.40 | 32.96 |
| m.16256C>T | 20.41 | 0.534 | (-43.98, 84.80) | 0.62 | 32.80 |
| m.16257C>A | -2.82 | 0.879 | (-39.22, 33.57) | -0.15 | 18.54 |
| m.16260C>T | -3.23 | 0.930 | (-74.91, 68.46) | -0.09 | 36.51 |
| m.16261C>T | -14.24 | 0.360 | (-44.78, 16.31) | -0.92 | 15.56 |
| m.16266C>A | -28.94 | 0.143 | (-67.69, 9.80) | -1.47 | 19.74 |
| m.16266C>T | -11.98 | 0.544 | (-50.72, 26.75) | -0.61 | 19.73 |
| m.16274G>A | -0.57 | 0.967 | (-27.15, 26.01) | -0.04 | 13.54 |
| m.16278C>T | -43.37 | 0.065 | (-89.52, 2.77) | -1.85 | 23.50 |
| m.16284A>G | 102.12 | 0.146 | (-35.67, 239.91) | 1.46 | 70.18 |
| m.16290C>T | 10.17 | 0.545 | (-22.79, 43.13) | 0.61 | 16.79 |
| m.16291C>T | -25.14 | 0.446 | (-89.91, 39.63) | -0.76 | 32.99 |
| m.16295C>T | 6.54 | 0.857 | (-64.55, 77.63) | 0.18 | 36.21 |
| m.16297T>C | -3.25 | 0.868 | (-41.57, 35.06) | -0.17 | 19.52 |
| m.16298T>C | 3.68 | 0.811 | (-26.46, 33.81) | 0.24 | 15.35 |
| m.16299A>G | -7.79 | 0.879 | (-108.59, 93.01) | -0.15 | 51.34 |
| m.16300A>G | 19.39 | 0.536 | (-42.02, 80.79) | 0.62 | 31.28 |
| m.16302A>C | 21.76 | 0.395 | (-28.40, 71.91) | 0.85 | 25.55 |

| (Continue) **Supplementary Table 7**. Associations of mtDNA substitutions with changes from pre-ART to post-ART in the CD4+T cell count in mtDNA genome-wide association analyses. | | | | | |
| --- | --- | --- | --- | --- | --- |
| Substitution | β | P | 95% CI | SE | t |

| m.16304T>C | -17.70 | 0.107 | (-39.22, 3.82) | -1.61 | 10.96 |
| --- | --- | --- | --- | --- | --- |
| m.16311T>C | 4.25 | 0.690 | (-16.62, 25.11) | 0.40 | 10.63 |
| m.16316A>G | -1.77 | 0.953 | (-61.20, 57.66) | -0.06 | 30.27 |
| m.16319G>A | 15.87 | 0.197 | (-8.27, 40.01) | 1.29 | 12.30 |
| m.16325T>C | -15.28 | 0.877 | (-209.19, 178.63) | -0.15 | 98.77 |
| m.16327C>T | 3.68 | 0.893 | (-49.85, 57.20) | 0.13 | 27.26 |
| m.16335A>G | -14.86 | 0.533 | (-61.60, 31.88) | -0.62 | 23.81 |
| m.16355C>T | 26.83 | 0.727 | (-123.80, 177.47) | 0.35 | 76.72 |
| m.16357T>C | 60.74 | 0.493 | (-112.95, 234.42) | 0.69 | 88.47 |
| m.16360C>T | 2.92 | 0.955 | (-98.21, 104.06) | 0.06 | 51.51 |
| m.16362T>C | -17.41 | 0.099 | (-38.08, 3.27) | -1.65 | 10.53 |
| m.16390G>A | 24.49 | 0.502 | (-47.05, 96.03) | 0.67 | 36.44 |
| m.16399A>G | 145.12 | 0.010 | (35.20, 255.05) | 2.59 | 55.99 |
| m.16519T>C | 0.53 | 0.952 | (-16.62, 17.68) | 0.06 | 8.73 |

β, slope; 95% CI, 95% confidence interval; SE, standard error
