# Supplementary material for: Baseline Characteristics of Mitochondrial DNA and Mutations Associated With Short-Term Posttreatment CD4+T-Cell Recovery in Chinese People With HIV
Source: Front Immunol. 2021 Dec 14;12:793375. doi: 10.3389/fimmu.2021.793375 (PMC8712318; doi:10.3389/fimmu.2021.793375)
Supplement: Supplementary file 1 [file DataSheet_1.zip › SupplementaryMaterial/Supplementary Table8.docx]

| **Supplementary Table 8**. Distributions of relative diversity density^a^ of synonymous and non-synonymous substitutions across 13 protein-coding genes in each sub-population. | | |
| --- | --- | --- |
| Sub-population | Relative diversity density of synonymous substitutions | Relative diversity density of non-synonymous substitutions |
| Class 1: Male, Han ethnic, Age 17-29, CD4 <200 | 0.000508766 | 0.000069514 |
| Class 2: Male, Han ethnic, Age 30-44, CD4 <200 | 0.000371585 | 0.000055393 |
| Class 3: Male, Han ethnic, Age 45-59, CD4 <200 | 0.000437917 | 0.000061196 |
| Class 4: Male, Han ethnic, Age ≥60, CD4 <200 | 0.000443333 | 0.000074107 |
| Class 5: Male, Han ethnic, Age 17-29, CD4 ≥200 | 0.000334483 | 0.000056535 |
| Class 6: Male, Han ethnic, Age 30-44, CD4 ≥200 | 0.000305999 | 0.000040526 |
| Class 7: Male, Han ethnic, Age 45-59, CD4 ≥200 | 0.000436974 | 0.000062044 |
| Class 8: Male, Han ethnic, Age ≥60, CD4 ≥200 | 0.000423774 | 0.000073063 |
| Class 9: Female, Han ethnic, Age 17-29, CD4 <200 | 0.000577218 | 0.000084134 |
| Class 10: Female, Han ethnic, Age 30-44, CD4 <200 | 0.000553400 | 0.000065879 |
| Class 11: Female, Han ethnic, Age 45-59, CD4 <200 | 0.000773932 | 0.000099766 |
| Class 12: Female, Han ethnic, Age ≥60, CD4 <200 | 0.000763881 | 0.000154476 |
| Class 13: Female, Han ethnic, Age 17-29, CD4 ≥200 | 0.000459175 | 0.000071818 |
| Class 14: Female, Han ethnic, Age 30-44, CD4 ≥200 | 0.000584508 | 0.000087635 |
| Class 15: Female, Han ethnic, Age 45-59, CD4 ≥200 | 0.000608546 | 0.000096547 |
| Class 16: Female, Han ethnic, Age ≥60, CD4 ≥200 | 0.000723676 | 0.000115857 |

^a^ Relative diversity density is assessed by the proportion of the observed number of types of synonymous or non-synonymous substitutions to all possible synonymous (8291) or non-synonymous (25894) substitutions, further divided by the sample size of each sub-population.
