# Supplementary material for: Baseline Characteristics of Mitochondrial DNA and Mutations Associated With Short-Term Posttreatment CD4+T-Cell Recovery in Chinese People With HIV
Source: Front Immunol. 2021 Dec 14;12:793375. doi: 10.3389/fimmu.2021.793375 (PMC8712318; doi:10.3389/fimmu.2021.793375)
Supplement: Supplementary file 1 [file DataSheet_1.zip › SupplementaryMaterial/Supplementary Table9.docx]

| **Supplementary Table 9**. Distribution of changes in acidity and polarity properties of amino acids. | | | | | | | | | | |
| --- | --- | --- | --- | --- | --- | --- | --- | --- | --- | --- |
| Sub-population | Plot | P^a^ | P^b^ | P^c^ | P^d^ | P^e^ | P^f^ | P^g^ | P^h^ | P^i^ |
| Class 1:  Male, Han ethnic,  Age 17-29, CD4 <200 | 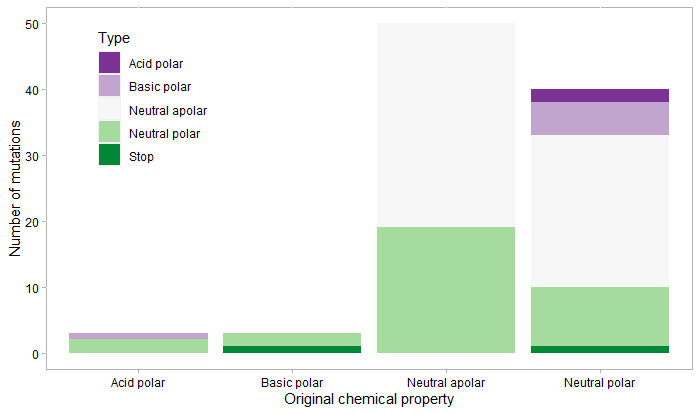 | 0.38 | 1.00 | 1.00 | 0.78 | - | 0.58 | 0.05 | 0.13 | 0.03 |
| Class 2:  Male, Han ethnic,  Age 30-44, CD4 <200 | 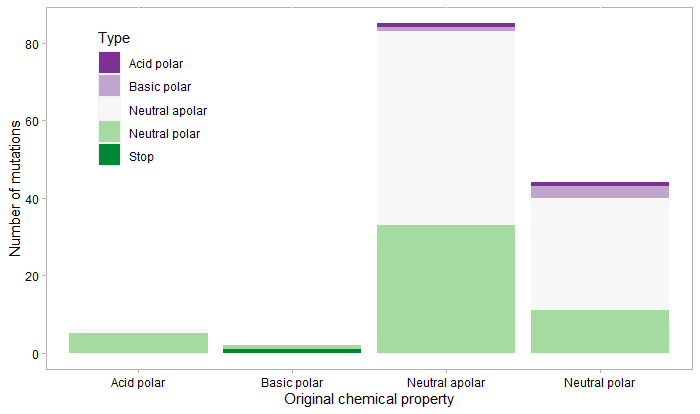 | 0.41 | 1.00 | 1.00 | 0.75 | - | 0.66 | 0.02 | 0.07 | 0.00 |
| Class 3:  Male, Han ethnic,  Age 45-59, CD4 <200 | 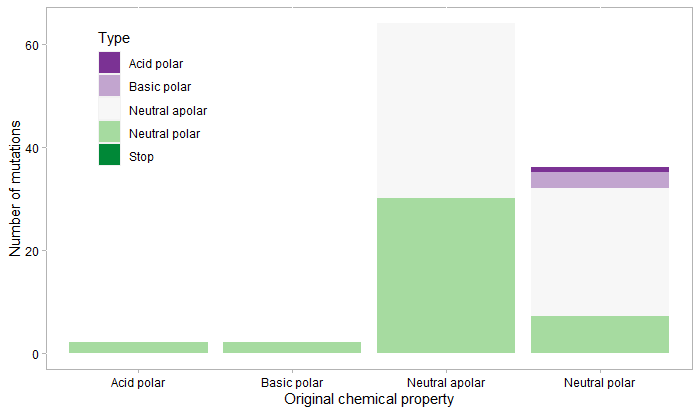 | 0.47 | 1.00 | 1.00 | 0.81 | - | 0.69 | 0.03 | 0.08 | 0.00 |
|  | | | | | | | | | | |
| (Continue) **Supplementary Table 9**. Distribution of changes in acidity and polarity properties of amino acids. | | | | | | | | | | |
| Sub-population | Plot | P^a^ | P^b^ | P^c^ | P^d^ | P^e^ | P^f^ | P^g^ | P^h^ | P^i^ |
| Class 4:  Male, Han ethnic,  Age ≥60, CD4 <200 | 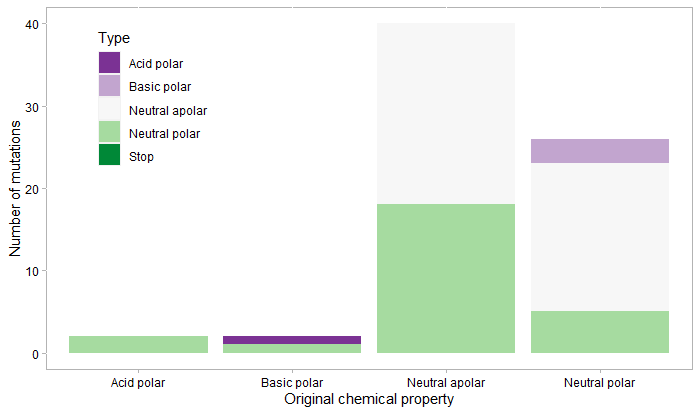 | 0.45 | 1.00 | 1.00 | 0.81 | - | 0.69 | 0.00 | 0.12 | 0.00 |
| Class 5:  Male, Han ethnic,  Age 17-29, CD4 ≥200 | 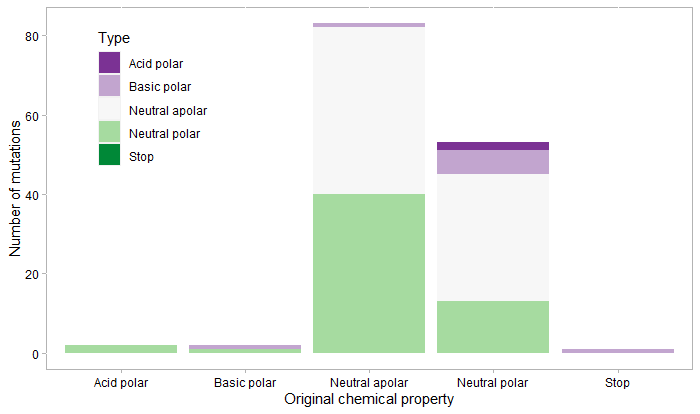 | 0.49 | 1.00 | 0.50 | 0.75 | 1.00 | 0.60 | 0.04 | 0.11 | 0.00 |
| Class 6:  Male, Han ethnic,  Age 30-44, CD4 ≥200 | 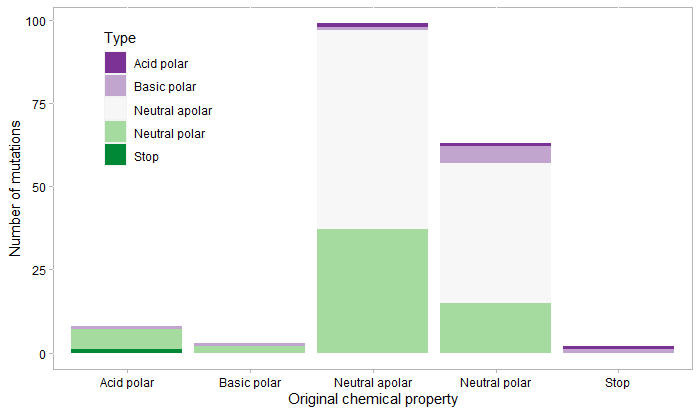 | 0.39 | 1.00 | 0.67 | 0.76 | 1.00 | 0.67 | 0.02 | 0.08 | 0.00 |
|  | | | | | | | | | | |
| (Continue) **Supplementary Table 9**. Distribution of changes in acidity and polarity properties of amino acids. | | | | | | | | | | |
| Sub-population | Plot | P^a^ | P^b^ | P^c^ | P^d^ | P^e^ | P^f^ | P^g^ | P^h^ | P^i^ |
| Class 7:  Male, Han ethnic,  Age 45-59, CD4 ≥200 | 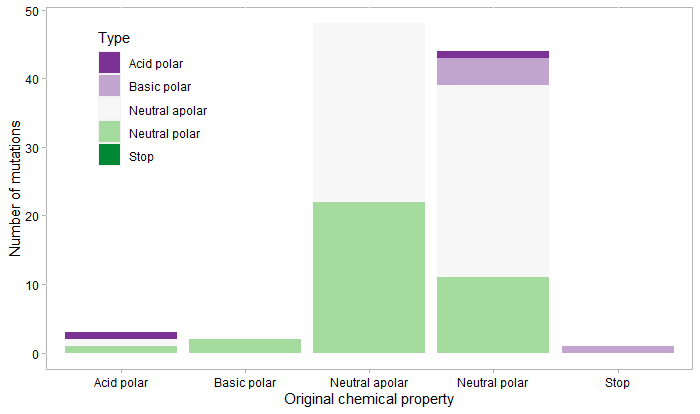 | 0.46 | 0.67 | 1.00 | 0.75 | 1.00 | 0.64 | 0.02 | 0.09 | 0.00 |
| Class 8:  Male, Han ethnic,  Age ≥60, CD4 ≥200 | 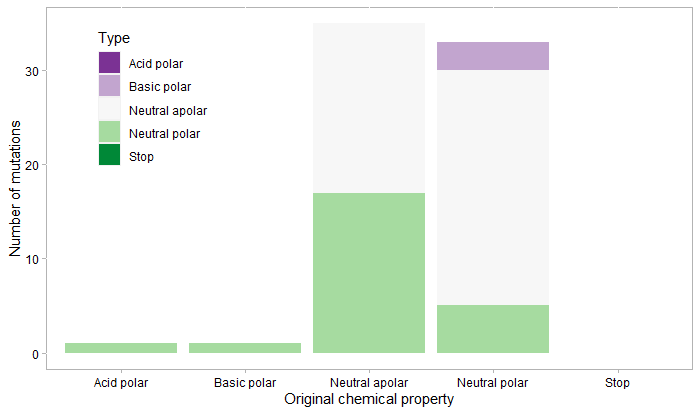 | 0.49 | 1.00 | 1.00 | 0.85 | - | 0.76 | 0.00 | 0.09 | 0.00 |
| Class 9:  Female, Han ethnic,  Age 17-29, CD4 <200 | 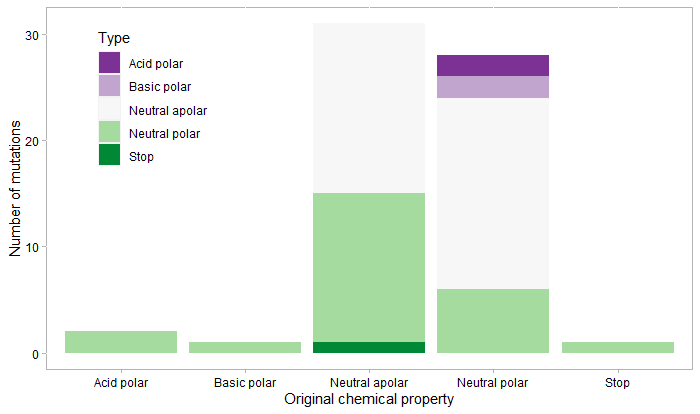 | 0.48 | 1.00 | 1.00 | 0.79 | 1.00 | 0.64 | 0.07 | 0.07 | 0.00 |
|  | | | | | | | | | | |
|  | | | | | | | | | | |
| (Continue) **Supplementary Table 9**. Distribution of changes in acidity and polarity properties of amino acids. | | | | | | | | | | |
| Sub-population | Plot | P^a^ | P^b^ | P^c^ | P^d^ | P^e^ | P^f^ | P^g^ | P^h^ | P^i^ |
| Class 10:  Female, Han ethnic,  Age 30-44, CD4 <200 | 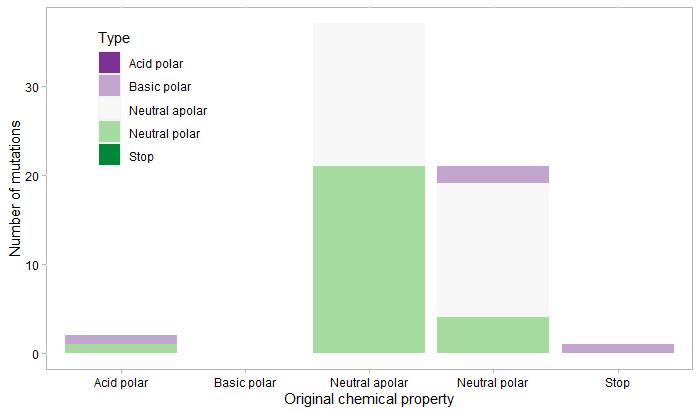 | 0.57 | 1.00 | - | 0.81 | 1.00 | 0.71 | 0.00 | 0.10 | 0.00 |
| Class 11:  Female, Han ethnic,  Age 45-59, CD4 <200 | 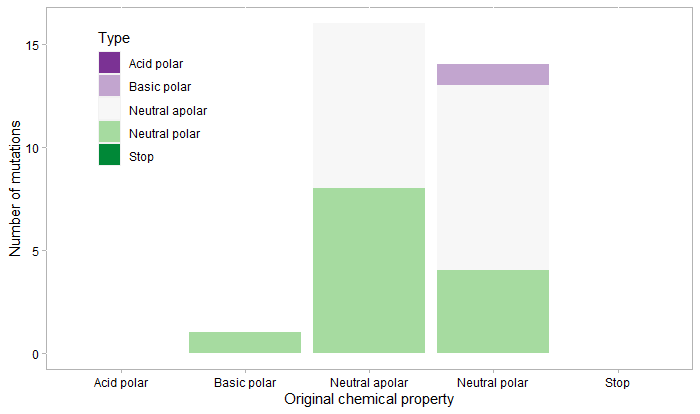 | 0.50 | - | 1.00 | 0.71 | - | 0.64 | 0.00 | 0.07 | 0.00 |
| Class 12:  Female, Han ethnic,  Age ≥60, CD4 <200 | 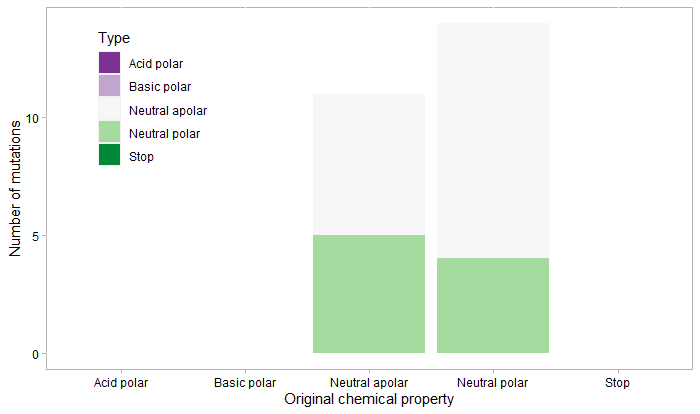 | 0.45 | - | - | 0.71 | - | 0.71 | 0.00 | 0.00 | 0.00 |
|  | | | | | | | | | | |
| (Continue) **Supplementary Table 9**. Distribution of changes in acidity and polarity properties of amino acids. | | | | | | | | | | |
| Sub-population | Plot | P^a^ | P^b^ | P^c^ | P^d^ | P^e^ | P^f^ | P^g^ | P^h^ | P^i^ |
| Class 13:  Female, Han ethnic,  Age 17-29, CD4 ≥200 | 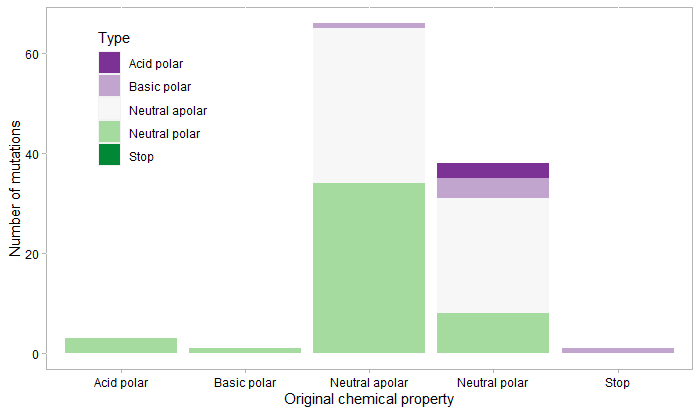 | 0.53 | 1.00 | 1.00 | 0.79 | 1.000 | 0.1 | 0.08 | 0.11 | 0.00 |
| Class 14:  Female, Han ethnic,  Age 30-44, CD4 ≥200 | 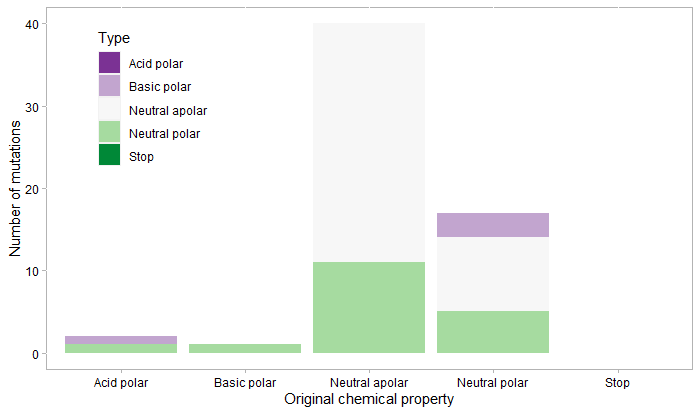 | 0.28 | 1.00 | 1.00 | 0.71 | - | 0.53 | 0.00 | 0.18 | 0.00 |
| Class 15:  Female, Han ethnic,  Age 45-59, CD4 ≥200 | 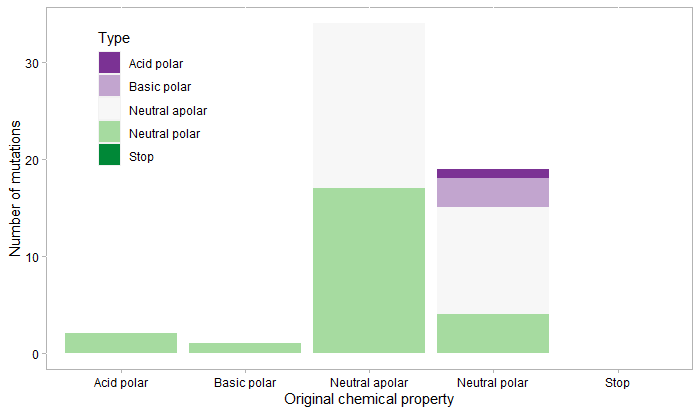 | 0.50 | 1.00 | 1.00 | 0.79 | - | 0.58 | 0.05 | 0.16 | 0.00 |
|  | | | | | | | | | | |
| (Continue) **Supplementary Table 9**. Distribution of changes in acidity and polarity properties of amino acids. | | | | | | | | | | |
| Sub-population | Plot | P^a^ | P^b^ | P^c^ | P^d^ | P^e^ | P^f^ | P^g^ | P^h^ | P^i^ |
| Class 16:  Female, Han ethnic,  Age ≥60, CD4 ≥200 | 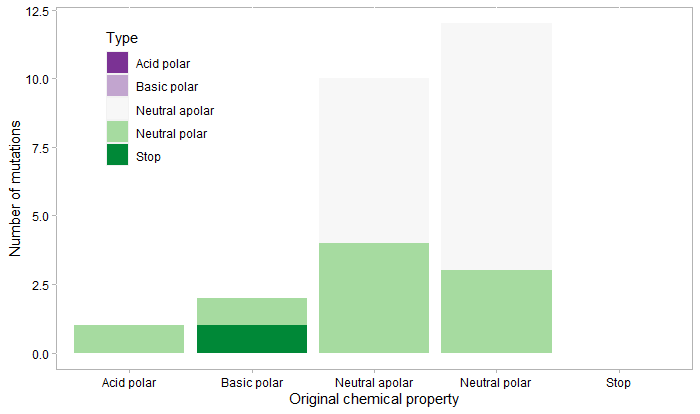 | 0.400 | 1.00 | 1.00 | 0.75 | - | 0.75 | 0.00 | 0.00 | 0.00 |

^a^ The proportion of non-synonymous substitutions mutating neutral apolar codons to codons with other different chemical properties (i.e. acid polar, basic polar, neutral polar, and stop) relative to all non-synonymous substitutions mutating neutral apolar codons

^b^ The proportion of non-synonymous substitutions mutating acid polar codons to codons with other different chemical properties (i.e. neutral apolar, basic polar, neutral polar, and stop) relative to all non-synonymous substitutions mutating acid polar codons

^c^ The proportion of non-synonymous substitutions mutating basic polar codons to codons with other different chemical properties (i.e. neutral apolar, acid polar, neutral polar, and stop) relative to all non-synonymous substitutions mutating basic polar codons

^d^ The proportion of non-synonymous substitutions mutating neutral polar codons to codons with other different chemical properties (i.e. neutral apolar, acid polar, basic polar, and stop) relative to all non-synonymous substitutions mutating neutral polar codons

^e^ The proportion of non-synonymous substitutions mutating stop polar codons to codons with other different chemical properties (i.e. neutral apolar, acid polar, basic polar, and neutral polar) relative to all non-synonymous substitutions mutating stop codons

^f^ The proportion of non-synonymous substitutions mutating neutral polar codons to neutral apolar codons relative to all non-synonymous substitutions mutating neutral polar codons

^g^ The proportion of non-synonymous substitutions mutating neutral polar codons to acid polar codons relative to all non-synonymous substitutions mutating neutral polar codons

^h^ The proportion of non-synonymous substitutions mutating neutral polar codons to basic polar codons relative to all non-synonymous substitutions mutating neutral polar codons

^i^ The proportion of non-synonymous substitutions mutating neutral polar codons to stop codons relative to all non-synonymous substitutions mutating neutral polar codons
