# Supplementary material for: Baseline Characteristics of Mitochondrial DNA and Mutations Associated With Short-Term Posttreatment CD4+T-Cell Recovery in Chinese People With HIV
Source: Front Immunol. 2021 Dec 14;12:793375. doi: 10.3389/fimmu.2021.793375 (PMC8712318; doi:10.3389/fimmu.2021.793375)
Supplement: Supplementary file 1 [file DataSheet_1.zip › SupplementaryMaterial/Supplementary Text1.docx]

**Supplementary Text 1. Other mutational features across 16 sub-populations**

### **Peak mutational diversity and volume**

The mutational diversity and volume of base variants, substitutions, indels, synonymous substitutions, and non-synonymous substitutions varied across classes of populations (Supplementary Table 1). Class 6 (male; aged 30-44; CD4 ≥200) recorded the richest diversity (N_d_=324) and the largest volume (N_v_=1881) of base variants in the division NDs and D-loop, respectively. By contrast, when diversity and volume were expressed relative to sample size per class and kbp size per macro division, class 12 (female; aged ≥60; CD4 <200) recorded the highest diversity density (D_d_=5.50) of base variants in D-loop whereas class 4 (male; aged ≥60; CD4 <200) recorded the highest volume density (D_v_=10.60) of base variants in D-loop. Likewise, class 6 recorded the richest diversity (N_d_=319) and the largest volume (N_v_=1680) of substitutions in the division NDs, however, class 12 recorded the highest diversity density (D_d_=4.47) of substitutions in D-loop and class 4 recorded the highest volume density of substitutions in D-loop (D_v_=9.18). When dividing substitutions into synonymous and non-synonymous ones, class 6 recorded the richest diversity of both synonymous (N_d_=232) and non-synonymous (N_d_=87) substitutions in the division NDs, respectively. This class also recorded the largest volume of both synonymous (N_d_=1244) and non-synonymous (N_d_=436) substitutions in the division NDs. By contrast, class 11 (female; aged 45-59; CD4 <200) and class 12 harbored the top diversity density of synonymous (D_d_=0.80) and non-synonymous (D_d_=0.94) substitutions in divisions CYB and ATPs, respectively. Class 13 (female; aged 17-29; CD4 ≥200) harbored the top volume density of both synonymous (D_v_=2.17) and non-synonymous (D_v_=2.69) substitutions in CYB and ATPs.

### **Trends in the volume with ages**

We assessed the trend in the volume density of substitutions pe macro division with ages across sub-populations (Supplementary Figure 1). Female participants with severer immunodeficiency, compared to counterparts with milder immunodeficiency, trended more steeply toward the higher volume density of substitutions in the divisions D-loop and tRNAs with ages. Male participants with severer immunodeficiency, compared to counterparts with milder immunodeficiency, trended more steeply with ages toward the higher volume density of substitutions in ATPs. However, unlike the diversity density, we did not observe the marked uptrend in the volume density of substitutions, synonymous substitutions, or non-synonymous substitutions with ages in many divisions.

### **Diversity of synonymous and non-synonymous substitutions**

We evaluated the relative diversity density of definite synonymous and non-synonymous substitutions within 13 protein-coding genes across 16 classes of sub-populations (Supplementary Table 8). The relative diversity density of synonymous and non-synonymous substitutions diverged across sub-populations with maximum 0.08% in class 11 (female, Han ethnic, aged 45-49, and CD4 <200) and maximum 0.02% in class 12 (female, Han ethnic, aged ≥60, and CD4 <200), respectively. Female participants exhibited an average ~1.6-fold richer relative diversity density in terms of both synonymous and non-synonymous substitutions, compared to male counterparts of identical levels of ages and pre-ART immune states. When fixing the gender and age, the severely immunocompromised participants displayed richer relative diversity density in terms of both synonymous substitutions (for male: class 1 VS class 5, class 2 VS class 6, class 3 VS class 7; for female: class 9 VS class 13, class 11 VS class 15, class 12 VS class 16) and non-synonymous substitutions (for male: class 1 VS class 5, class 2 VS class 6, class 4 VS class 8; for female: class 9 VS class 13, class 11 VS class 15, class 12 VS class 16).

When the analysis targeted each of 13 protein-coding genes, nearly all sub-populations of male participants obtained a prominent linear correlation for the observed number of synonymous substitutions relative to the maximum number of synonymous substitutions (R^2^ >80). But this did not apply for non-synonymous substitutions (R^2^ <50). Similarly, many sub-populations of female participants obtained a linear correlation for synonymous substitutions (50 < R^2^ <80) but a weaker linear correlation for non-synonymous substitutions (R^2^ <50) (Supplementary Figures 2 and 3).

### **Amino acid changes deduced from non-synonymous substitutions**

Most of non-synonymous substitutions originated from neutral apolar- and neutral polar-coding codons within each sub-population, following the pattern observed in the whole population of 856 participants. Only female participants with pre-ART CD4 ≥200 exhibited a monotonous increase with ages in the proportion of the non-synonymous substitutions mutating neutral polar-coding codons to neutral apolar-coding codons (Supplementary Table 9).

### **Transitions and transversions**

Like all 856 participants, the sub-population of each class demonstrated the remarkable dominance of transition types across the global mitochondrial genome, with the class 12 (female, Han ethnic, aged ≥60, and CD4 <200) recording the largest transition:transversion ratio (20.6:1) and the class 6 (male, Han ethnic, aged 33-44, and CD4 ≥200) the smallest one (10.7:1). When the scale was narrowed into 13 protein-coding genes, in general, the transition:transversion ratio increased steeply, with the class 4 recording the largest transition: transversion ratio (34.3:1) and the class 11 the smallest one (12.8:1) (Supplementary Table 10).

We next broke transitions in protein-coding genes into 4 types (~A;~C;~T;~G). In general, most sub-populations showed the slight deviation from unity in the ratio of prevalence of each type, and the strong mutational bias towards C and against T. For instance, the male, aged 17-29, Han ethnic, and CD4 <200 population presented this ratio of 1.13:1.48:1:1.36. Likewise, the male, aged 30-44, Han ethnic, and CD4 <200 population showed the ratio of 1.18:1.35:1:1.33 (Supplementary Table 10). Several sub-populations (classes 2, 4-6, 9-10) showed a good linear correlation (R^2^ ≥0.80) between the observed number of types of transitions per person and the maximum number of types of transitions in each of protein-coding genes, whilst the remaining 10 classes showed a moderate linear correlation (0.50< R^2^ <0.80) (Supplementary Table 11a). Female participants (2236.90) showed a ~1.7-fold lower average of the linear slope than male participants (1314.85), indicating women bore more rise in mutational variety of transitions per unit increase in the maximum transitions. Class 12 (female, Han ethnic, age ≥60, and CD4 <200) showed the highest unit increase in diversity for transitions with the smallest linear slope (668.69), conversely, class 6 (Male, Han ethnic, Age 30-44, CD4 ≥200) showed the smallest unit increase in diversity for transitions with the largest linear slope (3366.39) (Supplementary Table 11a).

When transversions in 13 protein-coding genes were broken into 4 types (~A;~C;~T;~G), most sub-populations exhibited the pronounced divergence in the ratio of four transversion types, accompanied by remarkable deviation from unity, and they also exhibited the strong mutational bias against G. For instance, the male, aged 45-59, Han ethnic, and CD4 <200 population presented this value of 6:3:4:1, whereas the ratio changed into 2:1:2:1 in the aged ≥60 population with identical gender, ethnicity, and pre-ART immune states (Supplementary Table 10). When comparing the observed number of transversions per person and maximum number of types of transversions in each of protein-coding genes, 5 classes (male: classes 5, 7, and 8; female: classes 13 and 14) presented a moderate correlation (0.50< R^2^ <0.80) whilst the remaining 11 classes showed a weak correlation (R^2^ <0.50) (Supplementary Table 11b). Female participants (17653.08) showed a ~2.3-fold lower average of the linear slope than male participants (7707.94), indicating women bore more rise in mutational variety of transversions per unit increase in the maximum transversions. Class 10 (female, Han ethnic, age 30-44, and CD4 <200) showed the highest unit increase in diversity for transitions with the smallest linear slope (2399.13), conversely, class 6 (Male, Han ethnic, Age 30-44, CD4 ≥200) showed the smallest unit increase in diversity for transitions with the largest linear slope (32055.66) (Supplementary Table 11b).

### **Bias of amino acid changes and physiochemical properties of amino acid changes deduced from non-synonymous substitutions**

Although no statistically significant differences in overall distributions of bias densities of amino acid changes were observed between each sub-population of CD4 <200 and its age- and gender-matched counterpart of CD4 ≥200, female populations of CD4 <200 showed higher bias densities of amino acid changes to a number of amino acids (age 17-29: A, D, F, H, I, L, N, P, S, STOP, Y; age 30-44: K, P, S, T; age 45-59: A, C, I, S, T, V, Y; age ≥60: A, F, I, L, M, N, P, S, T, V), compared to their age- and gender-matched counterparts of CD4 ≥200. Such difference dropped from age 17-29 but increased from age 30-44 to age ≥60. Specially, female populations of CD4 <200 showed a higher bias density of amino acid changes to S than age-matched female populations of CD4 ≥200, whereas male populations of CD4 <200 showed a higher bias density of amino acid changes to T than age-matched male populations of CD4 ≥200 (Supplementary Tables 12 and 13).

We next sought the patterns of the deduced changes of hydropathy, volume, chemical, charge, hydrogen donor or acceptor atoms, and polarity of amino acid replacements from non-synonymous mutations at the sub-population level (Supplementary Table 4). Male participants with CD4 <200 exhibited a monotonous increase with ages in the volumetric changes of amino acids from small to very small, or very small to small per person. This population also exhibited a monotonous increase with ages. Similarly, female participants with CD4 <200 exhibited a monotonous increase with ages in from very small to small. This population additionally experienced a monotonous increase in from aliphatic to hydroxyl per person, from sulfur to hydroxyl per person, and from nonpolar to polar per person. Conversely, these phenomena were not detected in neither male nor female participants with CD4 ≥200. Compared to their counterparts with CD4 ≥200 and identical ages, female participants with CD4 <200 showed more hydropathy changes from neutral to hydrophobic, more volumetric changes from large to small and from small to very mall, more chemical changes from hydroxyl to aliphatic, and more polarity changes from polar to nonpolar per person. However, male participants with CD4 <200 rarely followed this pattern.
